# Unified Approach to Diverse Fused Fragments via Catalytic Dehydrative Cyclization

**DOI:** 10.1002/chem.202201107

**Published:** 2022-07-04

**Authors:** Ashley J. Basson, Nathan R. Halcovitch, Mark G. McLaughlin

**Affiliations:** ^1^ Department of Chemistry Lancaster University Bailrigg Lancaster LA1 4YB UK

**Keywords:** calcium, cyclization, dehydration, fragments, polycyclic

## Abstract

A range of highly functionalized polycyclic fragments have been synthesized, employing a catalytic dehydrative cyclization. A range of nucleophiles are shown to be successful, with the reaction producing numerous high value motifs.

## Graphical abstract

**Access to diverse** polycyclic scaffolds via an efficient calcium catalyzed dehydration‐cyclization strategy is described. This approach provides a range of small molecule fragments bearing a range of medicinally relevant functional groups. Both Lewis and Bronsted acid pathways are in use, with the ability to fine tune the reactivity depending on the choice of solvent.

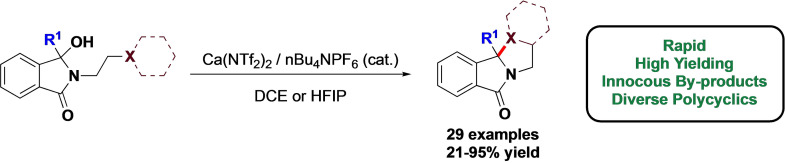

## Introduction

Fragment‐based drug discovery (FBDD) has undoubtedly made a large and sustained positive impact on medicinal chemistry campaigns since its inception.^[1]^ The ability to screen large collections of small molecules, coupled with advances in structural biology, has resulted in more structurally diverse lead compounds. Advances in synthetic chemistry, and most notably, the ability to synthesize a wide range of fragments bearing functional handles that facilitates rapid diversification underpin this success.

Fragment libraries have traditionally focused on flat, aromatic scaffolds with notable successes, however, these libraries have struggled to identify hits for new and complex biological targets.^[2]^ This has led to more focused libraries, consisting of 3D, sp^3^ rich small molecules. This increased concentration towards more complex scaffolds is not unfounded, with several elegant studies showing that shape is arguably one of the more important factors affecting biological activity. Furthermore, 3D scaffolds have improved physicochemical properties including solubility as well as improving specific ADMET properties.^[3]^

As mentioned, fragment diversity is intrinsically linked to advances in synthetic methodology, and access to novel fragments has remained a focus of the synthetic chemistry community. In particular, the development of methodology to access novel or less explored scaffolds continues to attract considerable effort.

**Figure 1:**
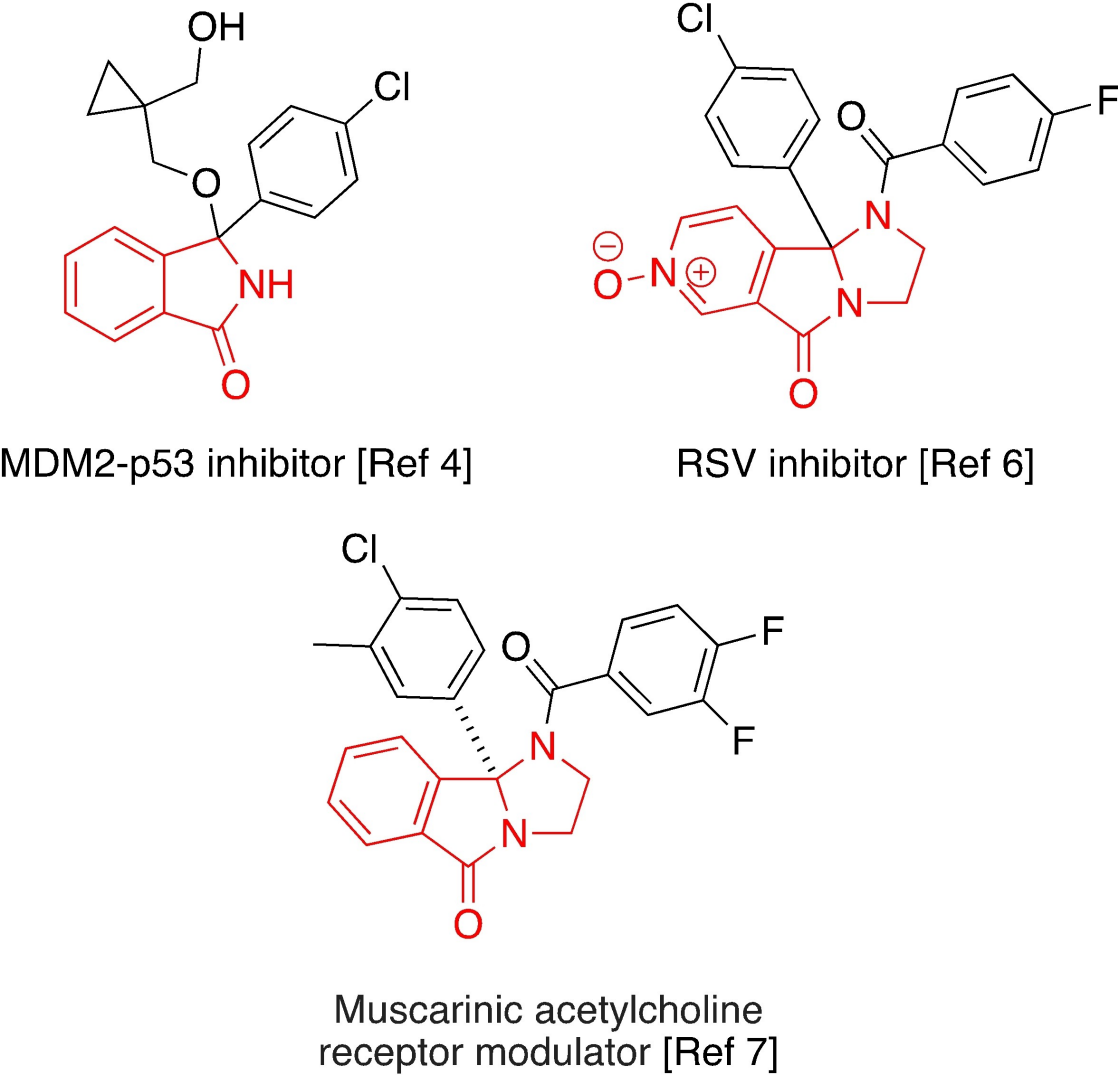
Selected biologically active isoindolinones.

Isoindolinones represent an important class of biologically active small molecules,^[4]^ and have seen increasing use in medicinal chemistry.^[5]^ Of particular interest to our group are tricyclic isoindolinones, which have shown to have anti‐inflammatory,^[6]^ anti‐addictive^[7]^ and CNS modulating effects (Figure 1).^[8]^

**Figure 2:**
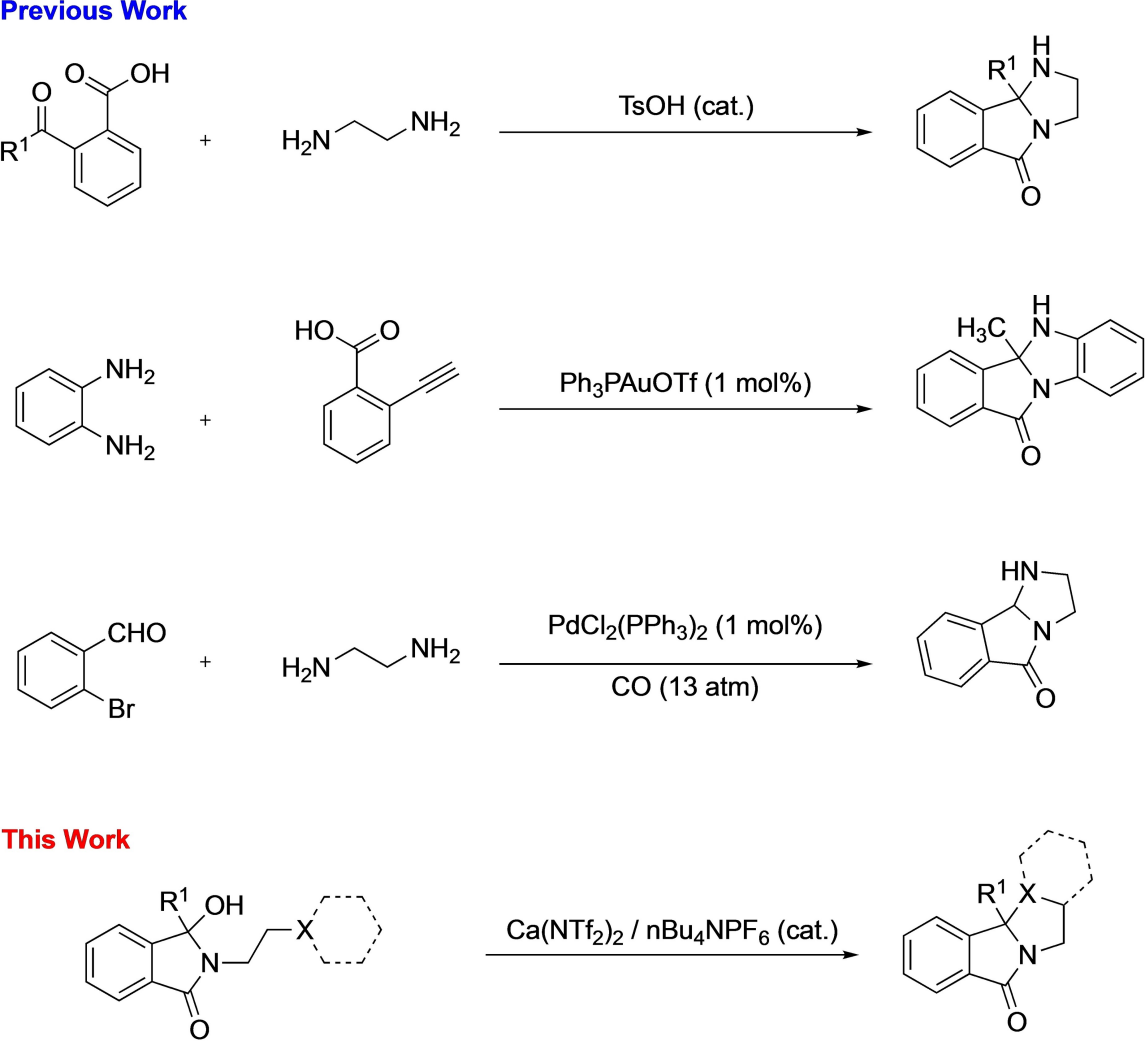
Example approaches to polycyclic fragments.

Their synthesis often relies upon a linear strategy to build the tricyclic core, which somewhat impedes its use in modular synthesis, with low yielding condensation reactions routinely employed.^[8b]^ Other approaches employing Brønsted acids,^[9]^ one‐pot cascades^[10]^ and palladium catalyzed carbonylations^[11]^ have all been successful (Figure 2). Although these methods all produce the desired scaffold, their utility is somewhat diminished by the accessibility of the starting materials as well as the inherent reactivity of the starting materials producing unwanted or isomeric mixtures.

Calcium catalysis, once thought to be a curiosity, has seen swift growth over the last decade.^[12]^ In particular, work by Niggemann,^[13]^ Harder„^[14]^ Lebœuf^[15]^ and others^[16]^ have shown that calcium is a functional group tolerant Lewis acid catalyst with wide ranging applications. Due to the success of our work on the derivatization of isoindolinones,^[17]^ we reasoned that we could access these important fragments through a rapid and facile dehydrative cyclisation procedure employing catalytic calcium bis(trifluoromethanesulfonimide) (Ca(NTf_2_)_2_).

## Results and Discussion

We began our investigation focusing on the dehydration‐cyclisation of sulfur nucleophiles, given the ready accessibility of the starting materials, as well as the fact that these thioazoloindoline motifs have a range of important biological activity. Optimization of the process focused on varying catalyst loading, temperature and solvent, and we quickly found that the reaction was amenable to low catalyst loadings in a variety of solvents (Table 1). In particular, running the reaction in hexafluoroisopropanol (HFIP) at 40 °C in the presence of 1 mol% catalyst provided the desired product 2a in 94 % after 15 minutes. Lowering the temperature has little effect on yield, however the reaction times are prolonged. A survey of solvents was also unsuccessful, with no reaction taking place in 1,2‐DCE and EtOAc We decided that given many functional groups are tolerant to elevated temperatures, running the reaction over a shorter period of time would prove more useful to the synthetic community.

**Table 1:** Selected optimization of sulfur‐based nucleophiles.

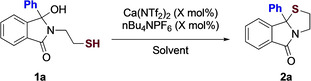					
Entry	Loading [mol %]	Temperature [°C]	Solvent	Time [h]	Yield^[a]^ [%]
1	10	80	1,2‐DCE	0.25	94
2	1	80	1,2‐DCE	0.25	90
3	1	80	EtOAc	0.25	96
4	10	40	HFIP	0.25	96
5	1	40	HFIP	0.25	94
6	1	r.t.	HFIP	12	94

[a] Isolated yield.

**Figure 3:**
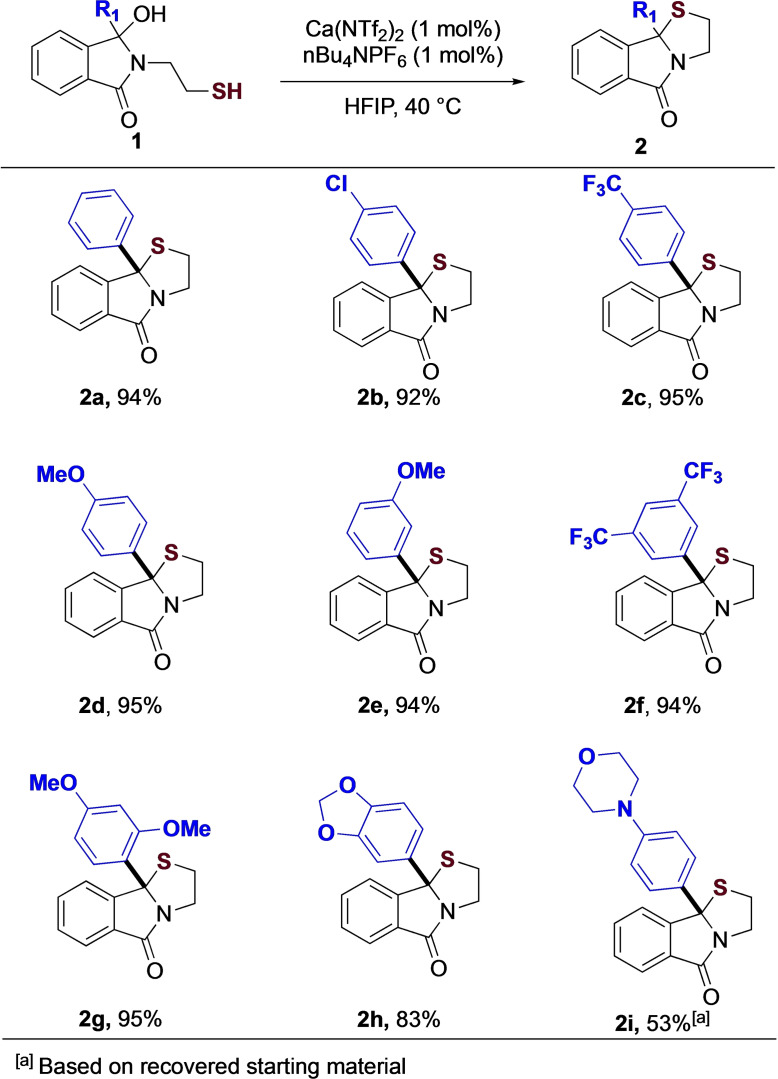
Thiol substrate scope.

With these optimized conditions in hand, we probed the substrate scope of the reaction. We were particularly interested in ensuring that the 6/5/5 ring system was substituted at the thiazole junction, given the lack of general methods to access these in the literature. As shown (Figure 3), the reaction is tolerant to a wide range of functionality, including electron withdrawing (**2 b, 2 c**) and electron donating (**2 d**) groups, providing the fused tricyclic in excellent yield. Differing (**2 e**) and multiple (**2 f, 2 g**) substitution is also well tolerated, as are oxygen (**2 h**) and nitrogen (**2 i**) based heterocyclic systems, providing the thiazoles in moderate to excellent yield.

Finally, attempts at employing alkylated species in the reaction was unsuccessful, instead undergoing complex fragmentation reaction in all cases.

Given our success in accessing 6/5/5 ring systems, we next turned our attention towards the formation of 6/5/6 scaffolds. We also decided to expand the range of nucleophiles within the study, and therefore moved onto the use of indole as reactive partner. This not only allows for the synthesis of complex motifs from readily available starting materials, but provides novel fused diazapolycyclics for inclusion in in‐house fragment libraries.

Subjecting **3 a** to our previously optimized conditions afforded **4 a** in excellent yields, albeit with slightly longer reaction times (Table 2) Efforts to reduce this, including alternative solvents and temperature was not fruitful. Nevertheless, given that the reaction is complete after 1.5 h, as well as the mild reaction conditions, we were happy to proceed.

**Table 2:** Indole optimization.

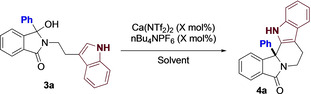					
Entry	Loading [mol %]	Temperature [°C]	Solvent	Time [h]	Yield^[a]^ [%]
1	5	80	1,2‐DCE	1	84
2	5	40	HFIP	1	80
3	1	40	HFIP	1	89

[a] Isolated yield.

**Figure 4:**
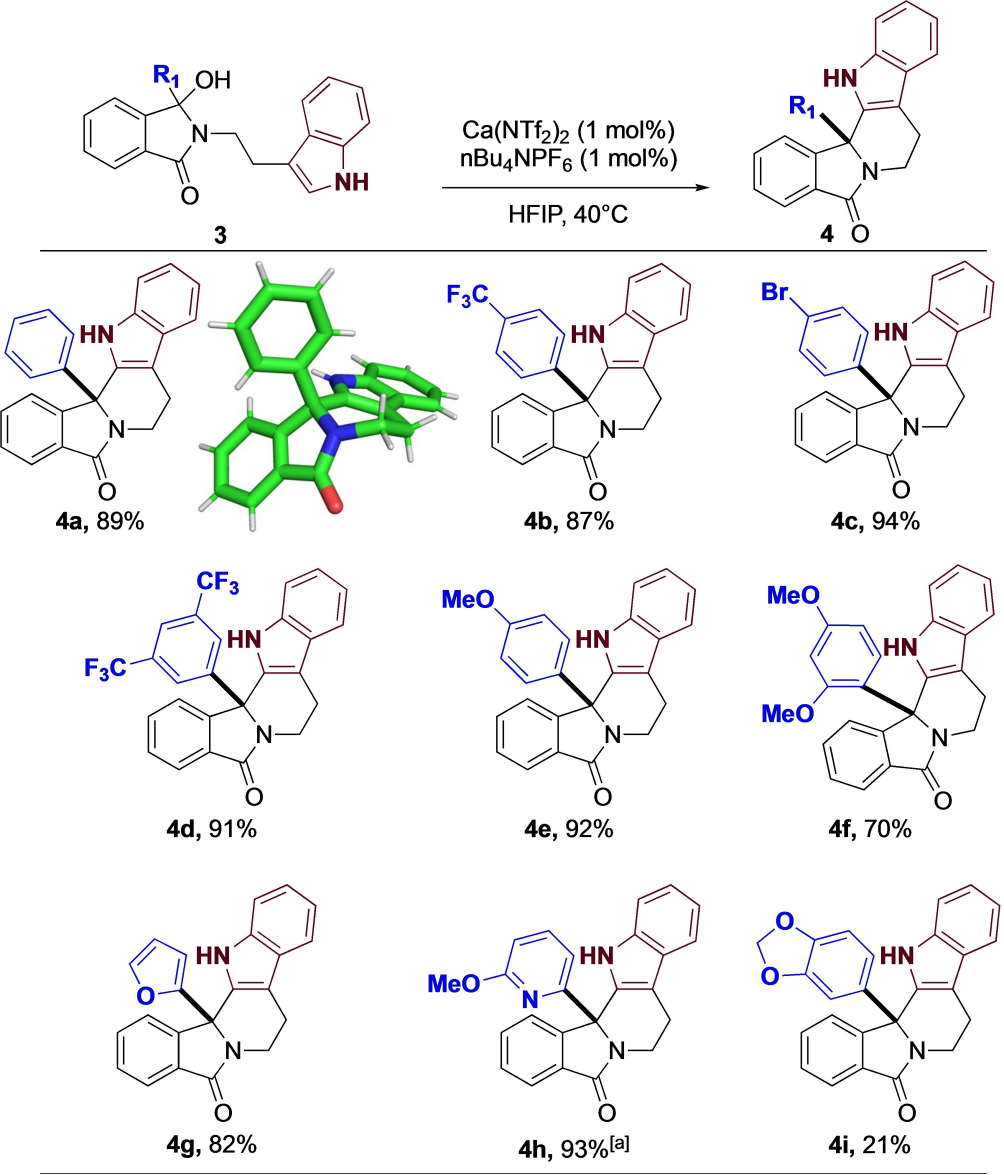
Indole substrates. [a] 1.2‐DCE at 80 °C with 1 mol% catalyst.

With this simple optimization complete, we explored the differing functionality at R^1^, and in particular, on groups of importance in medicinal chemistry. As shown (Figure 4), the reaction worked exceptionally well, with both electron withdrawing (**4 b‐d**) and donating groups (**4 e, 4 f**) having moderate to no effect on efficiency the reaction. Furthermore, aromatic heterocycles were well tolerated, with the reaction providing both furan (**4 g**) and pyridine (**4 h**) analogues in high yield. Subjecting the acid sensitive acetal to our reaction conditions, only a small amount of the described product (**4 i**) was isolated, with the mass balance being unreacted starting material.

**Figure 5:**
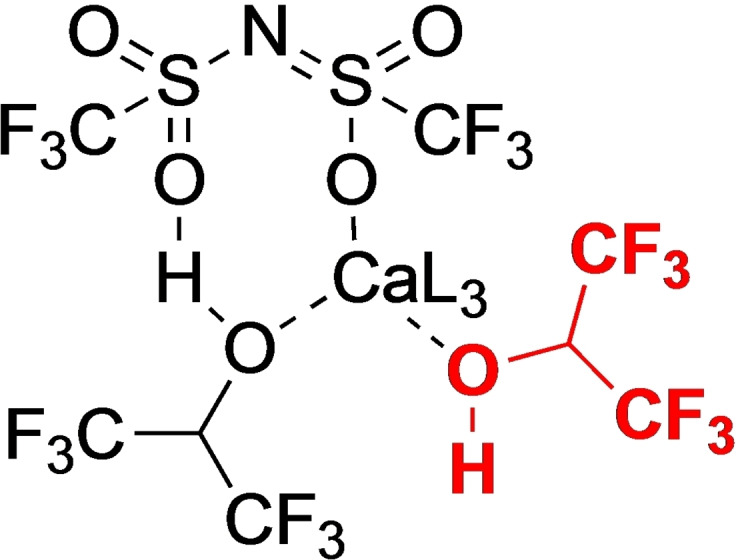
Postulated active Brønsted acid catalyst.

Although we envisaged the reaction to proceed via Lewis acid catalysis, with hidden Brønsted acid catalysis previously ruled out, the fact that the reaction works with improved reproducibility in HFIP suggests Brønsted acidity is playing a key role. Elegant computational studies by Lebœuf and co‐workers^[18]^ has shown that the addition of HFIP into the catalyst system employed here results in well‐defined Brønsted acid (Figure 5), which is capable of performing the same dehydration envisaged for the Lewis acid pathway.

**Figure 6:**
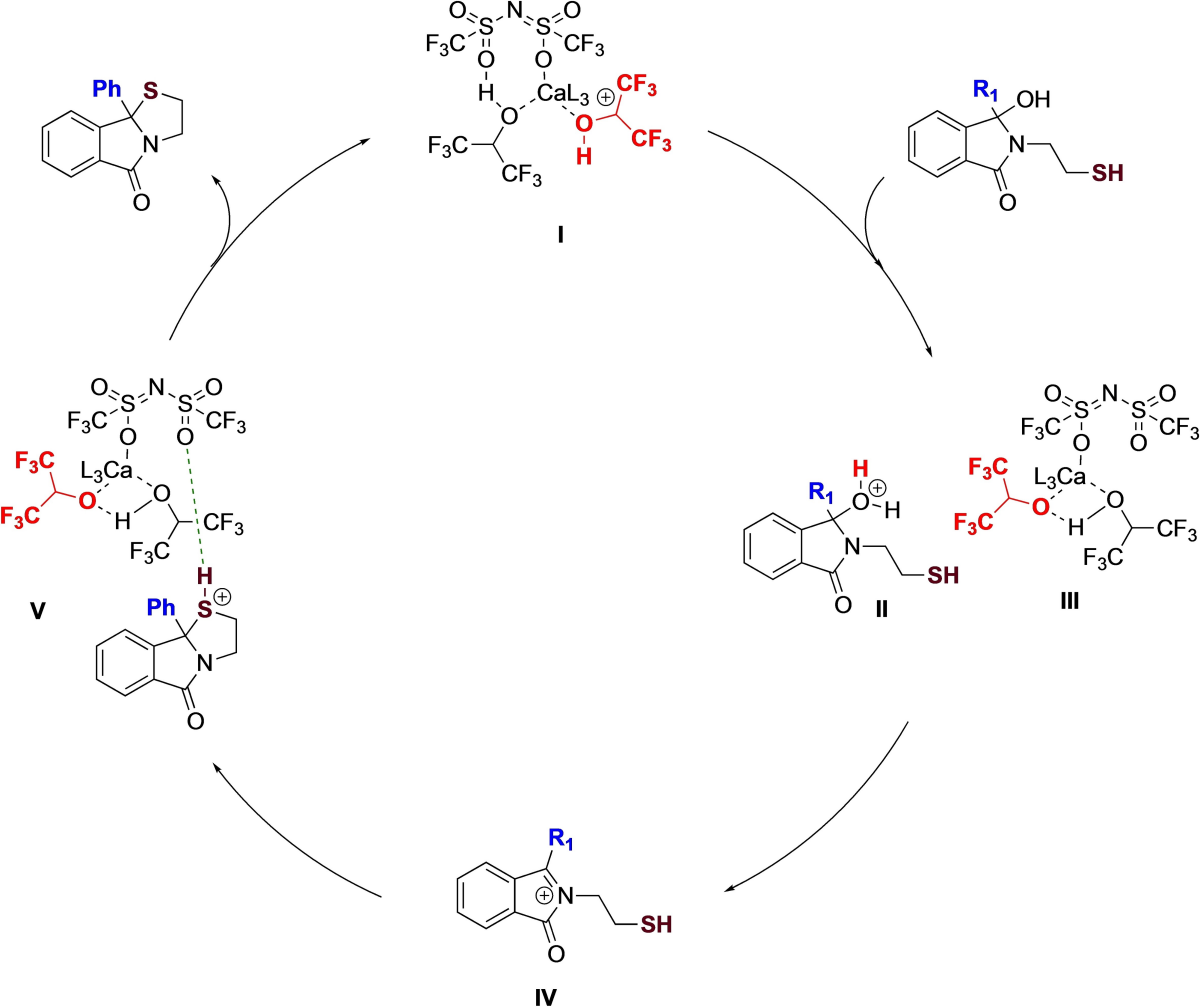
Proposed catalytic cycle.

Given that the reaction with the Lewis acid salt proceeds more slowly, it is reasonable to assume that the Brønsted acid type pathway is the favored one. We therefore propose the following mechanism based on previous studies (Figure 6). The Brønsted acid calcium complex **I** preferentially protonates the hydroxyl functionality of the hydrozylisoindolinone, to afford protonated **II** and complex **III**. Loss of water affords the desired N‐acyliminium ion **IV**, which undergoes intramolecular trapping with the tethered nucleophile and subsequent re‐complexation with **III** to provide **V**. Protonation of the NTf_2_ ligand and internal proton transfer gives the thiazolidine product and regenerates the catalyst.

We next turned our sights towards amine nucleophiles, given their importance in target synthesis and their ubiquity in medicinal chemistry. From our previous experience, we knew that in all likelihood, simple primary and secondary amines would not be amenable as reaction partners, and this turned out to be the case. However, switching to the more easily accessible, and modular, amide, proved to be much more successful. Optimization of this process was also relatively smooth, with a summary provided in Table 3. Treating **5 a**, readily available in two steps, with our previously described conditions resulted in decomposition, regardless of the temperature used. Careful reaction monitoring did not provide useful data, with decomposition occurring rapidly in all cases. A survey of previously successful solvents showed that the reaction could indeed progress (Entry 3). Increasing the temperature provided the desired product in high yield, and further investigation showed that increasing the catalyst loading gave a more reproducible yield and cleaner product.

**Table 3:** Amide optimization.

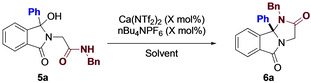					
Entry	Loading [mol %]	Temperature [°C]	Solvent	Time [h]	Yield^[a]^ [%]
1	10	40	HFIP	12	decomp.
2	10	80	EtOAc	12	decomp
3	10	80	1,2‐DCE	12	29
4	10	100	1,2‐DCE	4	88
5	20	100	1,2‐DCE	0.25	85

**Figure 7:**
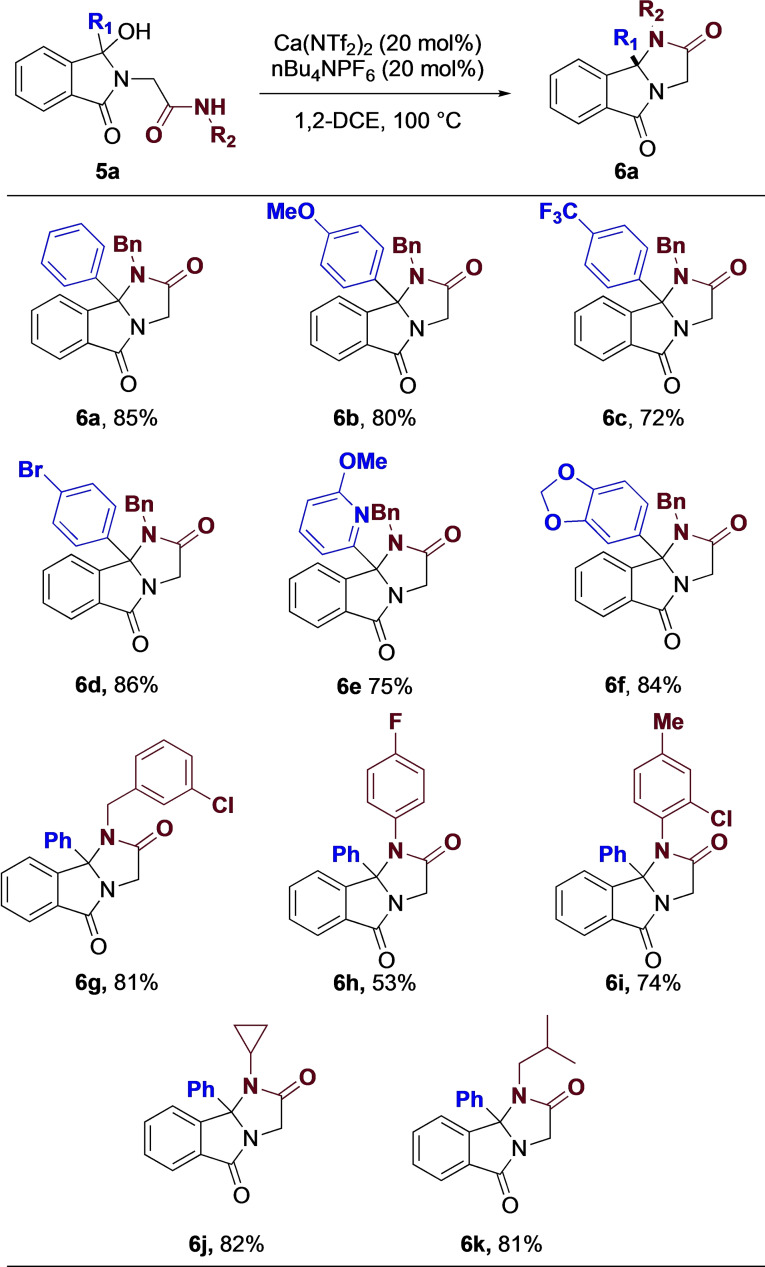
Amide substrate scope.

Once again, we wanted to probe the tolerance of the reaction, and in particular, wanted to vary both R^1^ and R^2^ (Figure 7). As expected, the reaction was tolerant to both electron donating and withdrawing groups, providing each in good yield. Nitrogen and oxygen heterocycles were also well tolerated, affording the complex fused 6/5/5 scaffold in high yield. Varying the substitution on the amide proved more variable. Substituted benzyl (**6 g**) produced the desired product in high yield, while a noticeable drop was observed in electron withdrawing aniline derivatives (**6 h**). Mixed electronics however worked well providing the fused ring system in high yields (**6 i**). Finally, alkyl substitution also worked well, with cyclic (**6 j**) and acyclic (**6 k**) being produced in high yield.

## Conclusion

We have described a facile, high yielding and green methodology to access highly functionalized polycyclic fragments. The reaction is tolerant to a wide range of useful functional groups, providing fused scaffolds rapidly. We envisage this methodology to be of importance to both natural product and medicinal chemists alike, and have been included in our in‐house fragment libraries.

## Experimental Section

All experimental details are given in the Supporting Information.

Deposition Number 2154226 (for **4 a**) contains the supplementary crystallographic data for this paper. These data are provided free of charge by the joint Cambridge Crystallographic Data Centre and Fachinformationszentrum Karlsruhe Access Structures service.

## Conflict of interest

The authors declare no conflict of interest.

1

## Supporting information

As a service to our authors and readers, this journal provides supporting information supplied by the authors. Such materials are peer reviewed and may be re‐organized for online delivery, but are not copy‐edited or typeset. Technical support issues arising from supporting information (other than missing files) should be addressed to the authors.

Supporting Information

## Data Availability

The data that support the findings of this study are available in the supplementary material of this article.
