# Supplementary material for: Unified Approach to Diverse Fused Fragments via Catalytic Dehydrative Cyclization
Source: Chemistry. 2022 Jul 4;28(48):e202201107. doi: 10.1002/chem.202201107 (PMC9542485; doi:10.1002/chem.202201107)

Chemistry–A European Journal

Supporting Information

Unified Approach to Diverse Fused Fragments via Catalytic Dehydrative Cyclization

Ashley J. Basson, Nathan R. Halcovitch, and Mark G. McLaughlin*

Unified Approach to Diverse Fused Fragments via Catalytic Dehydrative Cyclization

Ashley J. Basson, Nathan R. Halcovitch and Mark G. McLaughlin*

Department of Chemistry, Lancaster University, Bailrigg, Lancaster, LA1 4YB, UK

Contents

General Information 2

Synthetic Approach Summary 3

General Procedures for the Synthesis of 3-hydroxyisoindolinones.....................5

Sulfur Nucleophiles – Thiols 5

Carbon Nucleophiles - Indole...17

Nitrogen Nucleophiles - Amides...30

Crystallography Data 48

References 49

Copies of Spectra 50

General Information

Solvents & reagents

Reagents were purchased in the highest purity available from Acros Organics, Alfa Aesar, Fluorochem, TCI, Fisher Scientific or Sigma Aldrich. All solvents were purchased from commercial sources and used without purification (reagent grade). Metal salts and ligands were stored in a desiccator when not in use. Anhydrous solvent was prepared by storing solvent over activated 4Å MS for 72 hours. Standard vacuum line techniques were used and glassware was oven dried prior to use. Organic solvents were dried during workup using anhydrous Na_2SO_4. All reactions were performed using DrySyn heating mantles and pressure regulated vials or round bottom flasks.

Purification and chromatography

Thin Layer Chromatography (TLC) was carried out using aluminium plates coated with 60 F254 silica gel. Plates were visualised using UV light (254 or 365 nm) and developed with iodine and basic permanganate solution. Flash chromatography was performed on VWR Silica gel 60, 40–63 microns RE as the stationary phase and the solvents employed were of reagent grade.

Characterisation

^{1}H NMR spectroscopic data were obtained at 400 MHz (Bruker Ultrashield 400 Plus) and 13C{1H} NMR data were obtained at 100 MHz (Bruker Ultrashield 400 Plus) at 298 K. The chemical shifts are reported in parts per million (δ) relative to residual $CHCl_3$ (δH = 7.26 ppm) and $CDCl_3$ (δC = 77.16 ppm, central line.) The assignment of the signals in the 1 H and 13C NMR spectra was achieved through 2D-NMR techniques: COSY, HSQC and HMBC. Coupling constants (J) are quoted in Hertz. Infrared spectra were recorded on an Agilent Technologies Cary 630 FTIR spectrometer. High resolution mass spectrometry data were recorded using electron spray ionization (ESI) or atmospheric pressure chemical ionization (ESI) on a Shimadzu LCMS-IT-TOF mass spectrometer.

Synthetic Approach Summary

Access to thioazoloindoline scaffolds

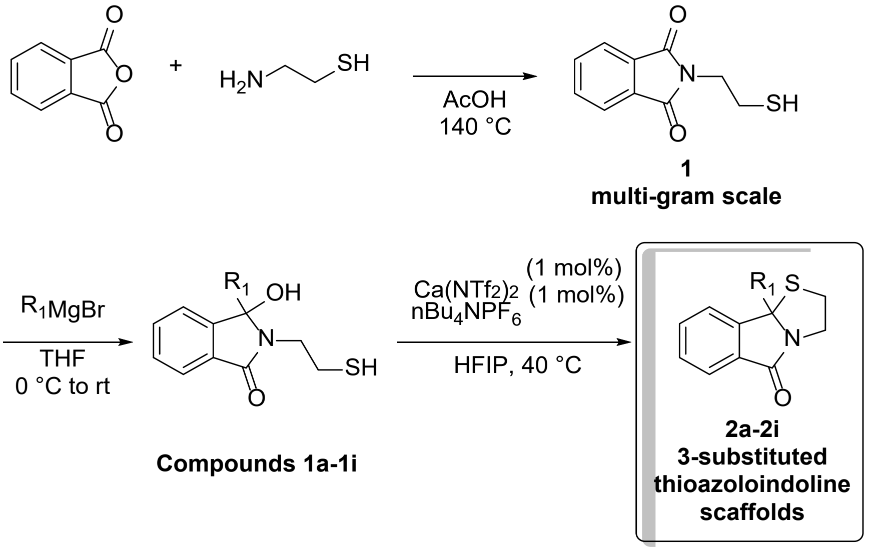

Access to diazapolycyclic scaffolds

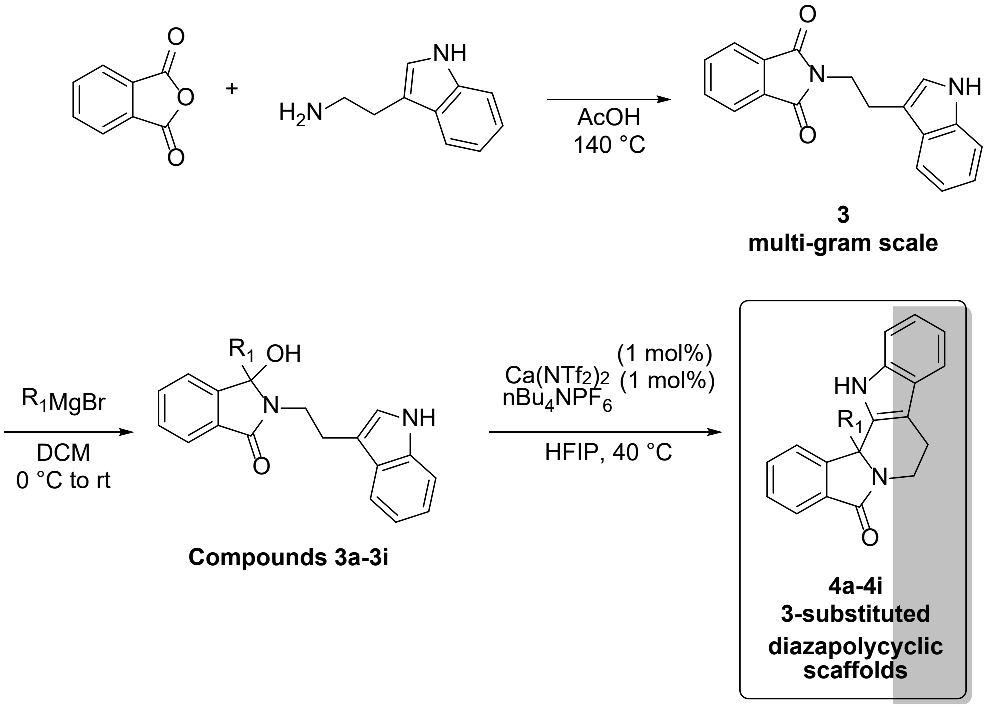

Access to 3-substituted 6/5/5 scaffolds scaffolds

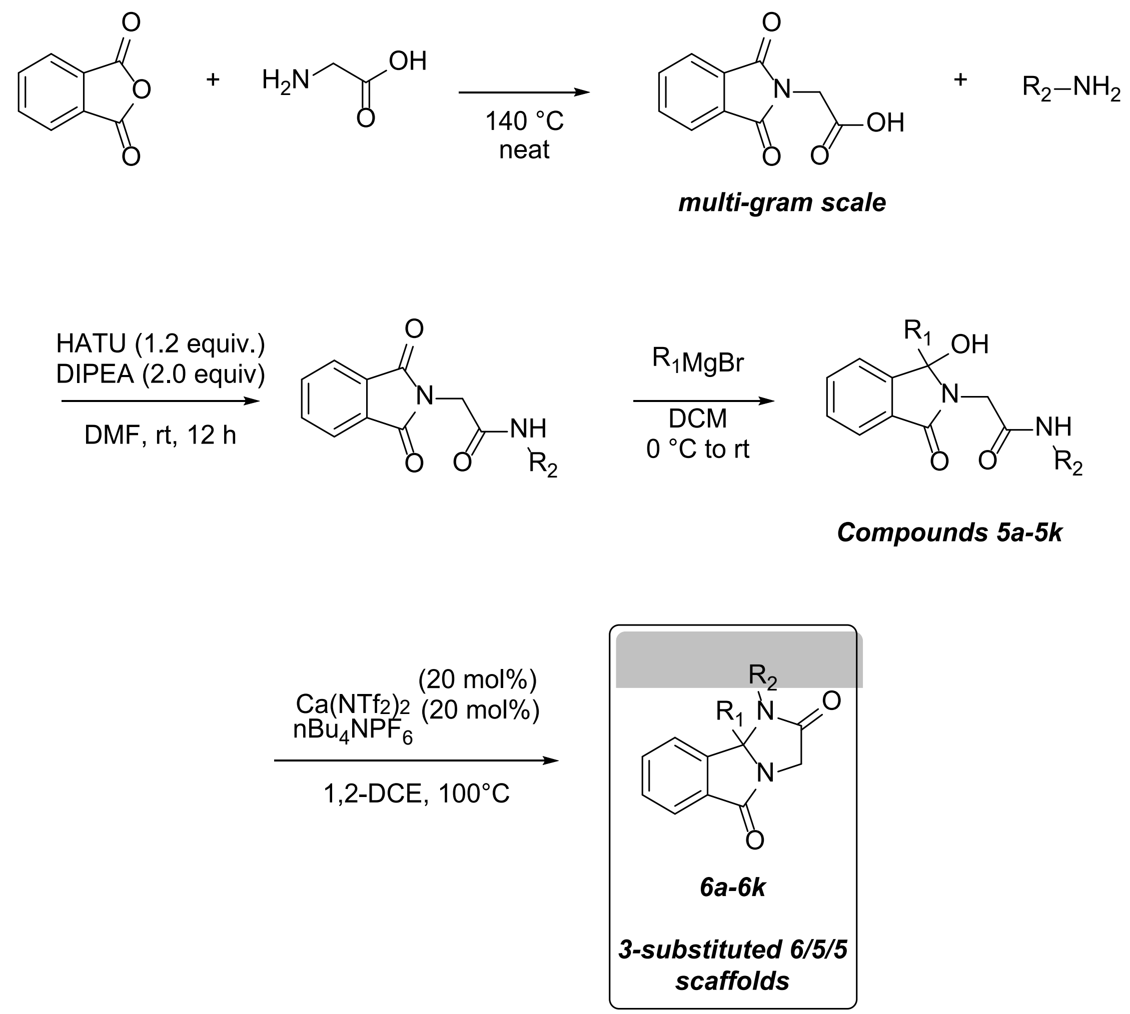

General Procedures for the Synthesis of 3-hydroxyisoindolinones

General Procedure A – Synthesis of 3-hydroxyisoindolinones by Grignard Addition

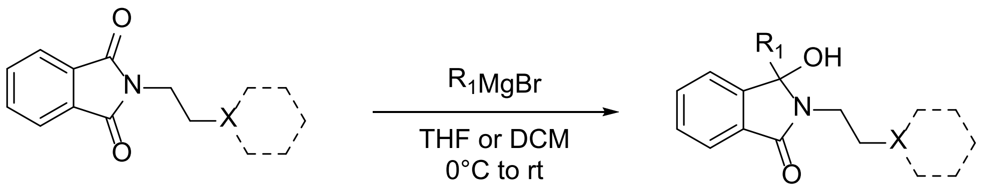

N-(2-mercaptoethyl)-phthalimide (1.0 equiv.) was added to a flame dried RBF and purged with argon. Dry THF or DCM (0.25 M) was added, and the solution was cooled to 0°C. The Grignard reagent* (3.0 equiv.) was then added dropwise, and the reaction was warmed to room temperature. Upon completion of the reaction (30 mins) which was indicated by the TLC, the reaction was quenched with NH_4Cl, and extracted into DCM (3 x 5 mL). The organic layers were combined, dried over Na_2SO_4, filtered and concentrated. The product was then purified by FCC (EtOAc:Hex) to afford the pure compound.

*Grignard reagents were either purchased or freshly prepared by suspending magnesium turnings (3.10 equiv.) in dry THF (1.0 M) under argon with 1,2-dibromoethane (0.1 equiv.) as an initiator. Dropwise addition of aryl halide (3.0 equiv.) and stirring for 2 h afforded the Grignard reagent which was then diluted to 0.5 M before being added to the electrophile.

General Procedure B – Synthesis of 3-hydroxyisoindolinones by lithium halogen exchange

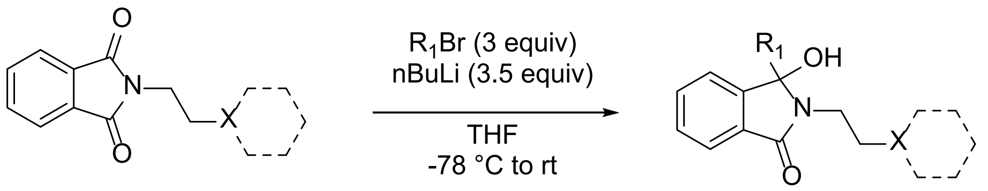

To a flame dried round bottomed flask purged with argon was added the corresponding aryl bromide (3.5 equiv.) and anhydrous THF (0.25 M). The solution was cooled to -78 °C and nBuLi (3 equiv. 2.5 M in hexanes) was added dropwise. The resulting solution was stirred at -78 °C for 1 hour. Phthalimide was then added in one portion and the reaction was stirred warmed to room temperature. Following completion of the reaction, indicated by TLC, the reaction was quenched with sat. aq. NH_4Cl and extracted with DCM (3 x 25 mL). The combined organic layers then dried over Na_2SO_4, filtered and concentrated. The product was then purified by FCC (EtOAc:Hex) to afford the pure compound.

Sulfur Nucleophiles – Thiols

Synthesis of N-(2-mercaptoethyl)-phthalimide (1)

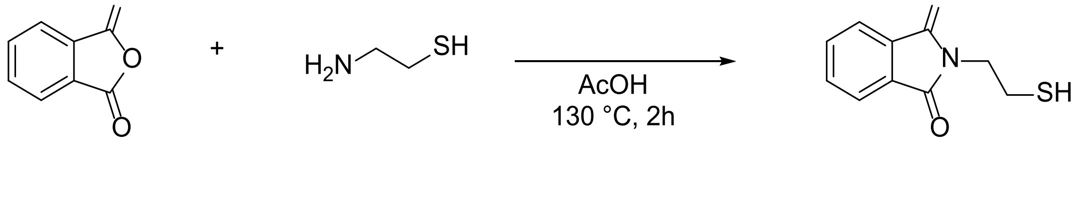

Phthalic anhydride (3.0 g, 20 mmol) and cysteamine (1.7 g, 22 mmol) was dissolved in acetic acid (30 mL) and stirred at 130°C for 2h. Upon completion of the reaction, indicated by TLC the reaction was cooled, and concentrated under reduced pressure by azeotropic removal of acetic acid with cyclohexane. The product was then purified by flash column chromatography (0 to 10% EtOAc:Hex) to afford the pure product as a white solid (1.7 g, 40%).

RF (1:1 EtOAc:Hex): 0.71

^{1}H NMR (400 MHz, $CDCl_3$): δ 7.88 – 7.84 (m, 2H), 7.75 – 7.72 (m, 2H), 3.89 (t, J = 7.3 Hz, 2H), 2.89 – 2.79 (m, 2H), 1.43 (t, J = 8.5 Hz, 1H).

^{13}C NMR (101 MHz, $CDCl_3$): δ 168.3, 134.3, 132.1, 123.6, 40.88, 23.1.

Data in accordance with literature[1]

3-hydroxy-3-phenyl-2-(2-sulfanylethyl)isoindolin-1-one (1a)

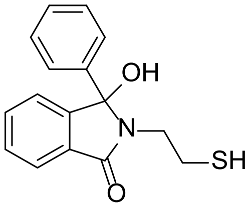

The title compound was prepared according to general procedure **A** from N-(2-mercaptoethyl)-phthalimide (450 mg, 2.20 mmol), phenylmagnesium bromide (3.0 M in Et_2O, 2.20 mL, 6.50 mmol) in THF (9 mL). Following completion of the reaction (2 h), purification by FCC (1:5 EtOAc:Hex, 1% NEt_3) afforded the pure product as a white solid (450 mg, 73%).

RF (1:1 EtOAc:Hex): 0.47

IR ν_{max} (cm^{-1}): 3306, 2957, 1678, 1608, 1425, 1071, 764

HRMS (APCI)m/z: $[M - H_2O]^+$ Calcd for $C_{16}H_{14}NOS$ 268.0796; Found 268.0806

^{1}H NMR (400 MHz, DMSO-d_6): δ 7.73 (d, J = 6.7 Hz, 1H), 7.56 (td, J = 7.4, 1.4 Hz, 1H), 7.51 (td, J = 7.4, 1.2 Hz, 1H), 7.39 – 7.30 (m, 5H), 7.26 (d, J = 6.9 Hz, 1H), 7.18 (s, 1H), 3.60 – 3.49 (m, 1H), 3.11 – 2.99 (m, 1H), 2.69 – 2.52 (m, 3H).

^{13}C NMR (101 MHz, DMSO-d_6): δ 166.6, 149.5, 139.9, 132.7, 130.2, 129.3, 128.6, 128.2, 125.8, 122.8, 122.6, 90.5, 42.5, 22.4.

3-(4-chlorophenyl)-3-hydroxy-2-(2-sulfanylethyl)isoindolin-1-one (1b)

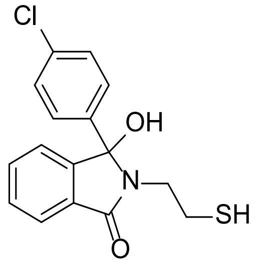

The title compound was prepared according to general procedure **A** from N-(2-mercaptoethyl)-phthalimide (200 mg, 0.97 mmol), 4-chlorophenylmagnesium bromide (1.0 M in Et_2O, 2.90 mL, 2.90 mmol) in THF (5 mL). Following completion of the reaction (2 h), purification by FCC (1:5 EtOAc:Hex, 1% NEt_3) afforded the pure product as an off-white solid (218 mg, 71%).

RF (1:1 EtOAc:Hex): 0.49

IR ν_{max} (cm^{-1}): 3316, 2860, 2821, 1673, 1608, 1478, 1146, 763

HRMS (APCI)m/z: $[M – H_2O]^+$ Calcd for $C_{16}H_{13}ClNOS$ 302.0406; Found 302.0413

^{1}H NMR (400 MHz, $CDCl_3$): δ 7.82 – 7.78 (m, 1H), 7.56 – 7.45 (m, 2H), 7.37 – 7.29 (m, 4H), 7.29 – 7.26 (m, 1H), 3.85 – 3.70 (m, 1H), 3.40 (br s, 1H), 3.15 – 3.03 (m, 1H), 2.90 – 2.76 (m, 1H), 2.70 – 2.55 (m, 1H), 1.40 (t, *J* = 8.5 Hz, 1H).

^{13}C NMR (101 MHz, $CDCl_3$): δ 167.9, 148.6, 137.1, 134.9, 133.3, 130.1, 129.1, 129.0, 127.75, 123.7, 122. 8, 90.8, 42.9, 23.3.

3-hydroxy-2-(2-sulfanylethyl)-3-[4-(trifluoromethyl)phenyl]isoindolin-1-one (1c)

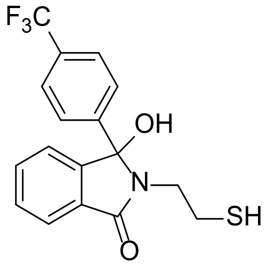

The title compound was prepared according to general procedure **A** from N-(2-mercaptoethyl)-phthalimide (200 mg, 0.97 mmol), (4-(trifluoromethyl)phenyl)magnesium bromide (0.5 M in THF, 6.0 mL, 2.90 mmol) in THF (4 mL). The Grignard reagent was freshly prepared from magnesium turnings (73 mg, 3.00 mmol), 4-bromobenzotrifluoride (650 mg, 0.41 mL, 2.90 mmol) and 1,2-dibromoethane (8 µL, 0.0098 mmol) in THF (3 mL). Following completion of the reaction (1 h), purification by FCC (1:1 EtOAc:Hex) afforded the pure product as a white solid (277 mg, 81%).

RF (1:1 EtOAc:Hex): 0.55

IR ν_{max} (cm^{-1}): 3200, 1671, 1614, 1325, 1070, 768

HRMS (APCI)m/z: $[M – H_2O]^+$ Calcd for $C_{17}H_{13}F_3NOS$ 336.0670; Found 336.0661

^{1}H NMR (400 MHz, $CDCl_3$): δ 7.86 – 7.80 (m, 1H), 7.62 (d, *J* = 8.4 Hz, 2H), 7.57 – 7.47 (m, 4H), 7.30 – 7.26 (m, 1H), 3.86 – 3.74 (m, 1H), 3.48 (br s, 1H), 3.12 – 3.00 (m, 1H), 2.93 – 2.79 (m, 1H), 2.72 – 2.60 (m, 1H), 1.42 (t, *J* = 8.5 Hz, 1H).

^{13}C NMR (101 MHz, $CDCl_3$): δ 168.0, 148.4, 142.7, 133.4, 130.9 (q, *J* = 32.7 Hz), 130.2, 130.1, 126.8, 125.9 (q, *J* = 3.8 Hz), 124.0 (q, *J* = 272.3 Hz), 123.8, 122.8, 90.7, 43.0, 23.3.

^{19}F NMR (376 MHz, $CDCl_3$): -62.6

3-hydroxy-3-(4-methoxyphenyl)-2-(2-sulfanylethyl)isoindolin-1-one (1d)

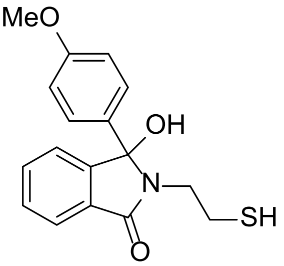

The title compound was prepared according to general procedure **A** from N-(2-mercaptoethyl)-phthalimide (150 mg, 0.72 mmol), 4-methoxyphenylmagnesium bromide (0.5 M in THF, 4.2 mL, 2.20 mmol) in THF (3 mL). The Grignard reagent was freshly prepared from magnesium turnings (55 mg, 2.20 mmol), 4-bromoanisole (406 mg, 0.28 mL, 2.20 mmol) and 1,2-dibromoethane (6 µL, 0.0072 mmol) in THF (2 mL). Following completion of the reaction (1 h), purification by FCC (1:1 EtOAc:Hex) afforded the pure product as a white solid (182 mg, 80%).

RF (1:1 EtOAc:Hex): 0.46

IR ν_{max} (cm^{-1}): 3191, 1670, 1610, 1467, 1092, 770

HRMS (APCI)m/z: $[M + H]^+$ Calcd for $C_{17}H_{16}NO_2S$ 298.0902; Found 298.0894

^{1}H NMR (400 MHz, $CDCl_3$): δ 7.81 – 7.76 (m, 1H), 7.56 – 7.43 (m, 2H), 7.34 – 7.26 (m, 3H), 6.86 (d, *J* = 9.0 Hz, 2H), 3.80 (s, 3H), 3.78 – 3.68 (m, 1H), 3.34 (s, 1H), 3.18 – 3.08 (m, 1H), 2.86 – 2.73 (m, 1H), 2.63 – 2.47 (m, 1H), 1.38 (t, *J* = 8.5 Hz, 1H).

^{13}C NMR (101 MHz, $CDCl_3$): δ 168.0, 148.7, 137.2, 134.9, 133.2, 130.0, 130.0, 129.1, 127.8, 123.6, 122.8, 90.8, 42.9, 23.2. (one resonance missing)

3-hydroxy-3-(3-methoxyphenyl)-2-(2-sulfanylethyl)isoindolin-1-one (1e)

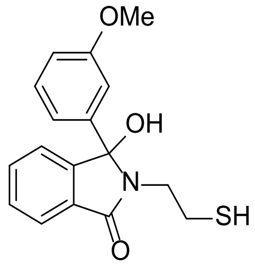

The title compound was prepared according to general procedure **A** from N-(2-mercaptoethyl)-phthalimide (200 mg, 0.97 mmol), 3-methoxyphenylmagnesium bromide (0.5 M in THF, 6.0 mL, 2.90 mmol) in THF (4 mL). The Grignard reagent was freshly prepared from magnesium turnings (73 mg, 3.00 mmol), 3-bromoanisole (541 mg, 0.37 mL, 2.90 mmol) and 1,2-dibromoethane (8 µL, 0.0098 mmol) in THF (3 mL). Following completion of the reaction (30 mins), purification by FCC (1:1 EtOAc:Hex) afforded the pure product as a white solid (216 mg, 71%).

RF (1:1 EtOAc:Hex): 0.46

IR ν_{max} (cm^{-1}): 3315, 2960, 2835, 1675, 1586, 1398, 1146, 762

HRMS (APCI)m/z: $[M – H_2O]^+$ Calcd for $C_{17}H_{16}NO_2S$ 298.0902; Found 298.0892

^{1}H NMR (400 MHz, $CDCl_3$): δ 7.71 (d, *J* = 7.2 Hz, 1H), 7.57 – 7.40 (m, 2H), 7.37 – 7.18 (m, 2H), 7.08 – 6.99 (m, 1H), 6.96 – 6.81 (m, 2H), 4.09 (s, 1H), 3.80 (s, 3H), 3.76 – 3.64 (m, 1H), 3.22 – 3.06 (m, 1H), 2.84 – 2.67 (m, 1H), 2.61 – 2.45 (m, 1H), 1.39 (t, *J* = 8.6 Hz, 1H).

^{13}C NMR (101 MHz, $CDCl_3$): δ 168.1, 160.0, 149.0, 140.2, 133.1, 130.1, 129.9, 129.7, 123.5, 122.8, 118.5, 114.0, 112.2, 91.1, 55.5, 43.0, 23.2.

3-[3,5-bis(trifluoromethyl)phenyl]-3-hydroxy-2-(2-sulfanylethyl)isoindolin-1-one (1f)

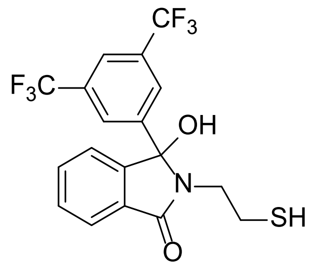

The title compound was prepared according to general procedure **A** from N-(2-mercaptoethyl)-phthalimide (200 mg, 0.97 mmol), 3,5-bis(trifluoromethyl)-phenylmagnesium bromide (0.5 M in THF, 6.0 mL, 2.90 mmol) in THF (4 mL). The Grignard reagent was freshly prepared from magnesium turnings (73 mg, 3.00 mmol), 1,3-bis(trifluoromethyl)-5-bromobenzene (848 mg, 0.49 mL, 2.90 mmol) and 1,2-dibromoethane (8 µL, 0.0098 mmol) in THF (3 mL). Following completion of the reaction (30 mins), purification by FCC (1:3 EtOAc:Hex, 1% NEt_3) afforded the pure product as a white solid (361 mg, 89%).

RF (1:1 EtOAc:Hex): 0.66

IR ν_{max} (cm^{-1}): 3161, 2898, 1681, 1282, 1120, 762

HRMS (APCI)m/z: $[M – H_2O]^+$ Calcd for $C_{18}H_{12}F_6NOS$ 404.0544; Found 404.0536

^{1}H NMR (400 MHz, $CDCl_3$): δ 7.93 – 7.82 (m, 4H), 7.61 – 7.53 (m, 2H), 7.30 – 7.27 (m, 1H), 3.86 (ddd, *J* = 14.1, 7.6, 5.2 Hz, 1H), 3.50 (s, 1H), 3.12 – 3.00 (m, 1H), 2.99 – 2.86 (m, 1H), 2.80 – 2.66 (m, 1H), 1.44 (dd, *J* = 9.0, 8.1 Hz, 1H).

^{13}C NMR (101 MHz, $CDCl_3$): δ 168.0, 147.7, 142.0, 133.7, 130.7, 130.0, 126.7, 124.1, 132.5 (q, *J* = 33.5 Hz), 123.1 (d, *J* = 272.6 Hz), 123.1 (d, *J* = 3.8 Hz), 122.8, 90.2, 42.9, 23.1.

^{19}F NMR (376 MHz, $CDCl_3$): -62.7

3-(2,4-dimethoxyphenyl)-3-hydroxy-2-(2-sulfanylethyl)isoindolin-1-one (1g)

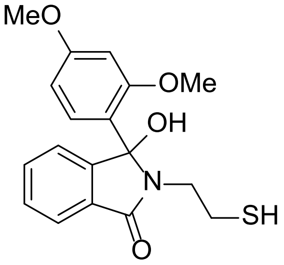

The title compound was prepared according to general procedure **A** from N-(2-mercaptoethyl)-phthalimide (200 mg, 0.97 mmol), 2,4-dimethoxyphenylmagnesium bromide (0.5 M in THF, 6.0 mL, 2.90 mmol) in THF (4 mL). The Grignard reagent was freshly prepared from magnesium turnings (73 mg, 3.00 mmol), 1-bromo-2,4-dimethoxybenzene (628 mg, 0.37 mL, 2.90 mmol) and 1,2-dibromoethane (8 μL, 0.0098 mmol) in THF (3 mL). Following completion of the reaction (30 mins), purification by FCC (1:1 EtOAc:Hex, 1% NEt_3) afforded the pure product as a yellow solid (152 mg, 46%).

RF (1:1 EtOAc:Hex): 0.31

IR ν_{max} (cm^{-1}): 3283, 2995, 2933, 1661, 1582, 1403, 1185, 1033, 837

HRMS (APCI)m/z: $[M - H_2O]^+$ Calcd for $C_{18}H_{19}NO_3S$ 328.1007; Found 328.0894

^1H NMR (400 MHz, DMSO-d_6): 7.88 (d, J = 8.7 Hz, 1H), 7.72 – 7.62 (m, 1H), 7.53 – 7.40 (m, 2H), 7.12 (dd, J = 6.1, 1.5 Hz, 1H), 6.84 (s, 1H), 6.63 (dd, J = 8.7, 2.5 Hz, 1H), 6.41 (d, J = 2.4 Hz, 1H), 3.76 (s, 3H), 3.50 – 3.36 (m, 1H), 3.24 (s, 3H), 3.04 – 2.89 (m, 1H), 2.57-2.52 (m, 1H), 2.48 - 2.43 (m, 1H), 2.42 – 2.29 (m, 1H).

^{13}C NMR (101 MHz, $CDCl_3$): δ 168.4, 161.7, 157.7, 149.1, 132.4, 131.2, 129.5, 129.2, 123.0, 121.9, 118.3, 104.7, 99.5, 55.7, 55.5, 43.2, 23.2. (one resonance missing)

3-(1,3-benzodioxol-5-yl)-3-hydroxy-2-(2-sulfanylethyl)isoindolin-1-one (1h)

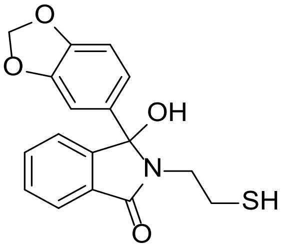

The title compound was prepared according to general procedure **B** from N-(2-mercaptoethyl)-phthalimide (200 mg, 0.97 mmol), 1-bromo-3,4-(methylenedioxy)benzene (776 mg, 0.47 mL, 3.86 mmol) and n-butyllithium (2.5 M in Hexane, 1.20 mL, 2.90 mmol) in THF (3 mL). Following completion of the reaction (1 h), purification by FCC (1:1 EtOAc:Hex) afforded the pure product as a dark yellow solid (100 mg, 31%).

RF (1:1 EtOAc:Hex): 0.37

IR ν_{max} (cm^{-1}): 3073, 2939, 2889, 1698, 1608, 1485, 1239, 1034, 807

HRMS (APCI)m/z: $[M - H_2O]^+$ Calcd for $C_{17}H_{14}NO_3S$ 312.0694; Found 312.0691

^1H NMR (400 MHz, $CDCl_3$): δ 7.51 (d, *J* = 7.4 Hz, 1H), 7.45 (td, *J* = 7.5, 1.2 Hz, 1H), 7.36 (td, *J* = 7.4, 1.0 Hz, 1H), 7.26 – 7.23 (m, 1H), 6.88 (dd, *J* = 8.3, 1.7 Hz, 1H), 6.76 – 6.70 (m, 2H), 5.92 (dd, *J* = 7.4, 1.4 Hz, 2H), 5.03 (s, 1H), 3.51 (ddd, *J* = 14.2, 9.7, 5.4 Hz, 1H), 3.04 (ddd, *J* = 14.1, 9.7, 5.9 Hz, 1H), 2.67 – 2.53 (m, 1H), 2.45 – 2.34 (m, 1H).

^{13}C NMR (101 MHz, $CDCl_3$): δ 171.0, 149.6, 148.2, 147.9, 134.2, 133.1, 129.1, 128.8, 124.5, 122.9, 119.8, 108.2, 106.9, 101.6, 82.1, 43.1, 38.3.

3-hydroxy-3-(4-morpholinophenyl)-2-(2-sulfanylethyl)isoindolin-1-one (1i)

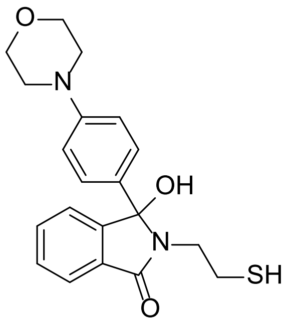

The title compound was prepared according to general procedure **B** from N-(2-mercaptoethyl)-phthalimide (100 mg, 0.48 mmol), 4-(4-bromophenyl)morpholine (409 mg, 1.70 mmol) and n-butyllithium (2.5 M in Hexane, 0.58 mL, 1.45 mmol) in THF (2 mL). Following completion of the reaction (30 mins), purification by FCC (1:3 EtOAc:Hex) afforded the pure product as a white solid (35 mg, 20%).

RF (1:1 EtOAc:Hex): 0.31

IR ν_{max} (cm^{-1}): 3211, 2952, 2917, 2846, 1672, 1608, 1405, 1122, 930

HRMS (APCI)m/z: $[M - H_2O]^+$ Calcd for $C_{20}H_{21}N_2O_2S$ 353.1324; Found 353.1311

^1H NMR (400 MHz, $CDCl_3$): δ 7.84 – 7.80 (m, 1H), 7.54 – 7.43 (m, 2H), 7.32 – 7.26 (m, 3H), 6.85 (d, *J* = 9.0 Hz, 2H), 3.86 – 3.82 (m, 4H), 3.81 – 3.73 (m, 1H), 3.23 – 3.10 (m, 5H), 3.08 (s, 1H), 2.90 – 2.78 (m, 1H), 2.70 – 2.57 (m, 1H), 1.40 (t, *J* = 8.5 Hz, 1H).

^{13}C NMR (101 MHz, $CDCl_3$): δ 167.9, 149.2, 134.8, 133.0, 131.8, 129.7, 128.9, 127.2, 123.6, 122.8, 115.3, 91.2, 67.0, 48.9, 42.9, 23.4.

Thiols – Products

General procedure C

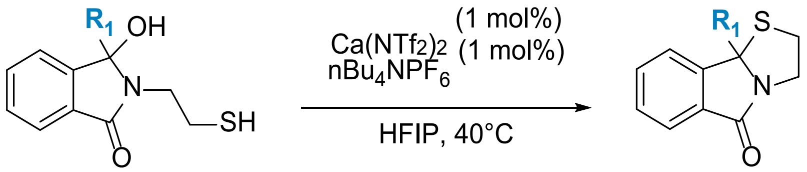

To a 4 mL vial capped with a capped with teflon cap was added $Ca(NTf_2)_2$ (1 mol%) and nBu_4NPF_6 (1 mol%) in HFIP (0.2 M). 3-hydroxyisoindolinone (1 equiv.) was added and the reaction was stirred at 40 °C until TLC analysis indicated full conversion to the product. The solution was then concentrated and purified by FCC (EtOAc:Hex) to afford the pure compound.

9b-phenyl-2,3-dihydrothiazolo[2,3-a]isoindol-5-one (2a)

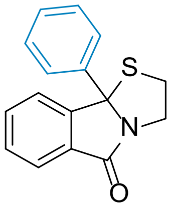

The title compound was prepared according to general procedure **C** from 3-hydroxy-3-phenyl-2-(2-sulfanylethyl)isoindolin-1-one (50 mg, 0.175 mmol), $Ca(NTf_2)_2$ (1.1 mg, 0.00175 mmol) and nBu_4NPF_6 (0.7 mg, 0.00175 mmol) in HFIP (0.9 mL). Following completion of the reaction (30 mins), purification by FCC (1:5 EtOAc:Hex) afforded the pure compound as a yellow oil (44 mg, 94%).

RF (1:1 EtOAc:Hex): 0.65

IR ν_{max} (cm^{-1}):): 3054, 3006, 2939, 1702, 1608, 1467, 1323, 1092, 744

HRMS (APCI)m/z: $[M + H]^+$ Calcd for $C_{16}H_{14}NOS$ 268.0796; Found 268.0801

^{1}H NMR (400 MHz, $CDCl_3$): δ 7.82 – 7.78 (m, 1H), 7.66 – 7.61 (m, 2H), 7.48 (td, *J* = 7.4, 1.4 Hz, 1H), 7.43 (td, *J* = 7.4, 1.3 Hz, 1H), 7.39 – 7.28 (m, 4H), 4.58 – 4.46 (m, 1H), 3.57 – 3.48 (m, 1H), 3.42 – 3.26 (m, 2H).

^{13}C NMR (101 MHz, $CDCl_3$): δ 171.1, 149.6, 140.5, 133.1, 129.1, 129.0, 128.8, 128.5, 126.1, 124.5, 123.00, 82.1, 43.1, 38.2.

9b-(4-chlorophenyl)-2,3-dihydrothiazolo[2,3-a]isoindol-5-one (2b)

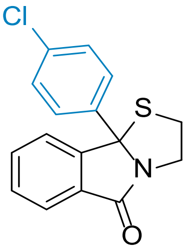

The title compound was prepared according to general procedure **C** from 3-(4-chlorophenyl)-3-hydroxy-2-(2-sulfanylethyl)isoindolin-1-one (100 mg, 0.313 mmol), $Ca(NTf_2)_2$ (1.9 mg, 0.00313 mmol) and nBu_4NPF_6 (1.2 mg, 0.00313 mmol) in HFIP (1.6 mL). Following completion of the reaction (30 mins), purification by FCC (1:5 EtOAc:Hex) afforded the pure compound as a colourless oil (94 mg, 92%).

RF (1:1 EtOAc:Hex): 0.71

IR ν_{max} (cm^{-1}): 3055, 2941, 1703, 1465, 1321, 1090, 818

HRMS (APCI)m/z: $[M + H]^+$ Calcd for $C_{16}H_{13}ClNOS$ 302.0406; Found 302.0416

^1H NMR (400 MHz, $CDCl_3$): δ 7.81 (d, *J* = 6.7 Hz, 1H), 7.58 (d, *J* = 8.6 Hz, 2H), 7.50 (td, *J* = 7.5, 1.5 Hz, 1H), 7.46 (td, *J* = 7.4, 1.2 Hz, 1H), 7.33 (d, *J* = 8.6 Hz, 2H), 7.28 – 7.23 (m, 2H), 4.56 – 4.44 (m, 1H), 3.60 – 3.49 (m, 1H), 3.36 – 3.27 (m, 2H).

^{13}C NMR (101 MHz, $CDCl_3$): δ 171.1, 149.3, 139. 2, 134.6 133.3, 129.3, 129.0, 129.0, 127.7, 124.7, 122.9, 81.7, 43.2, 38.4.

9b-[4-(trifluoromethyl)phenyl]-2,3-dihydrothiazolo[2,3-a]isoindol-5-one (2c)

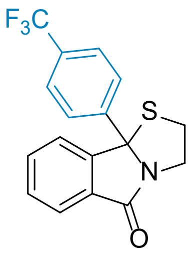

The title compound was prepared according to general procedure **C** from 3-hydroxy-2-(2-sulfanylethyl)-3-[4-(trifluoromethyl)phenyl]isoindolin-1-one (100 mg, 0.283 mmol), $Ca(NTf_2)_2$ (1.7 mg, 0.00283 mmol) and nBu_4NPF_6 (1.1 mg, 0.00283 mmol) in HFIP (1.4 mL). Following completion of the reaction (1 h), purification by FCC (1:4 EtOAc:Hex) afforded the pure compound as a colourless oil (95 mg, 95%).

RF (1:4 EtOAc:Hex): 0.42

IR ν_{max} (cm^{-1}): 3051, 2941, 1707, 1608, 1467, 1320, 1111, 740

HRMS (APCI)m/z: $[M + H]^+$ Calcd for $C_{17}H_{13}F_3NOS$ 336.0670; Found 336.0664

^1H NMR (400 MHz, $CDCl_3$): δ 7.82 (dd, *J* = 6.7, 1.5 Hz, 1H), 7.78 (d, *J* = 8.3 Hz, 2H), 7.63 (d, *J* = 8.3 Hz, 2H), 7.56 – 7.43 (m, 2H), 7.28 (dd, *J* = 6.7, 1.1 Hz, 1H), 4.59 – 4.48 (m, 1H), 3.60 – 3.51 (m, 1H), 3.37 – 3.26 (m, 2H).

^{13}C NMR (101 MHz, $CDCl_3$): δ 171.1, 148.9, 144.9, 133.4, 130.8 (q, *J* = 32.6 Hz), 129.5, 129.0, 126.7, 125.9 (q, *J* = 3.5 Hz), 124.9, 124.0 (q, *J* = 272.6 Hz), 123.0, 81.6, 43.4, 38.3.

^{19}F NMR (376 MHz, CDCl3): δ 62.54

9b-(4-methoxyphenyl)-2,3-dihydrothiazolo[2,3-a]isoindol-5-one (2d)

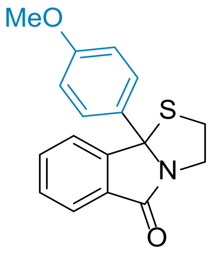

The title compound was prepared according to general procedure **C** from 3-hydroxy-3-(4-methoxyphenyl)-2-(2-sulfanylethyl)isoindolin-1-one (80 mg, 0.254 mmol), $Ca(NTf_2)_2$ (1.5 mg, 0.00254 mmol) and nBu_4NPF_6 (1.0 mg, 0.00254 mmol) in HFIP (1.3 mL). Following completion of the reaction (30 mins), purification by FCC (1:5 EtOAc:Hex) afforded the pure compound as a colourless oil (72 mg, 95%).

RF (1:5 EtOAc:Hex): 0.62

IR v_{max} (cm^{-1}): 3051, 2935, 2837, 1700, 1608, 1508, 1247, 1169, 1031

HRMS (APCI)m/z: $[M + H]^+$ Calcd for $C_{17}H_{16}NO_2S$ 298.0902; Found 298.0894

^1H NMR (400 MHz, $CDCl_3$): δ 7.79 (d, *J* = 7.3 Hz, 1H), 7.55 (d, *J* = 8.8 Hz, 2H), 7.52 – 7.40 (m, 2H), 7.28 (d, *J* = 7.4 Hz, 1H), 6.87 (d, *J* = 8.8 Hz, 2H), 4.55 – 4.43 (m, 1H), 3.80 (s, 3H), 3.60 – 3.47 (m, 1H), 3.39 – 3.28 (m, 2H).

^{13}C NMR (101 MHz, $CDCl_3$): δ 171.1, 159.8, 149.9, 133.1, 132.3, 129.0, 128.9, 127.6, 124.5, 123.0, 114.1, 81.9, 55.5, 43.1, 38.3.

9b-(3-methoxyphenyl)-2,3-dihydrothiazolo[2,3-a]isoindol-5-one (2e)

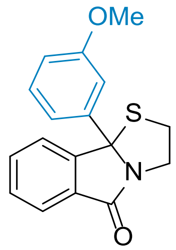

The title compound was prepared according to general procedure **C** from 3-hydroxy-3-(3-methoxyphenyl)-2-(2-sulfanylethyl)isoindolin-1-one (80 mg, 0.254 mmol), $Ca(NTf_2)_2$ (1.5 mg, 0.00254 mmol) and nBu_4NPF_6 (1.0 mg, 0.00254 mmol) in HFIP (1.3 mL). Following completion of the reaction (15 mins), purification by FCC (1:1 EtOAc:Hex) afforded the pure compound as white solid (71 mg, 94%).

RF (1:1 EtOAc:Hex): 0.63

IR v_{max} (cm^{-1}): 3068, 2954, 1698, 1579, 1347, 1247, 1049, 874

HRMS (APCI)m/z: $[M + H]^+$ Calcd for $C_{17}H_{16}NO_2S$ 298.0902; Found 298.0883

^1H NMR (400 MHz, $CDCl_3$): δ 7.80 (ddd, *J* = 7.2, 1.4, 0.8 Hz, 1H), 7.49 (td, *J* = 7.4, 1.4 Hz, 1H), 7.44 (td, *J* = 7.4, 1.3 Hz, 1H), 7.34 – 7.31 (m, 1H), 7.31 – 7.26 (m, 1H), 7.23 (ddd, *J* = 7.8, 1.7, 1.2 Hz, 1H), 7.19 – 7.15 (m, 1H), 6.84 (ddd, *J* = 8.0, 2.6, 1.1 Hz, 1H), 4.54 – 4.45 (m, 1H), 3.80 (s, 3H), 3.58 – 3.47 (m, 1H), 3.42 – 3.28 (m, 1H).

^{13}C NMR (101 MHz, $CDCl_3$): δ 171.1, 160.00, 149.5, 142.3, 133.1, 130.0, 129.2, 129.0, 124.6, 123.0, 118.6, 113.7, 112.0, 82.1, 55.5, 43.3, 38.3.

9b-[3,5-bis(trifluoromethyl)phenyl]-2,3-dihydrothiazolo[2,3-a]isoindol-5-one (2f)

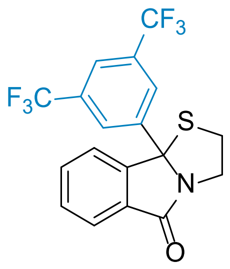

The title compound was prepared according to general procedure **C** from 3-[3,5-bis(trifluoromethyl)phenyl]-3-hydroxy-2-(2-sulfanylethyl)isoindolin-1-one (100 mg, 0.237 mmol), $Ca(NTf_2)_2$ (1.4 mg, 0.00237 mmol) and nBu_4NPF_6 (1.0 mg, 0.00237 mmol) in HFIP (1.2 mL). Following completion of the reaction (20 mins), purification by FCC (3:10 EtOAc:Hex) afforded the pure compound as a white solid (90 mg, 94%).

RF (1:1 EtOAc:Hex): 0.77

IR ν_{max} (cm^{-1}): 3040, 2950, 1716, 1608, 1279, 1122, 742

HRMS (APCI)m/z: $[M + H]^+$ Calcd for $C_{18}H_{12}F_6NOS$ 404.0544; Found 404.0557

^1H NMR (400 MHz, $CDCl_3$): δ 8.10 (s, 2H), 7.90 – 7.79 (m, 2H), 7.60 – 7.48 (m, 2H), 7.25 – 7.22 (m, 1H), 4.64 – 4.51 (m, 1H), 3.65 – 3.51 (m, 1H), 3.40 – 3.25 (m, 2H).

^{13}C NMR (101 MHz, $CDCl_3$): δ 171.1, 148.3, 144.1, 133.7, 132.4 (q, *J* = 33.6 Hz), 129.9, 128.8, 126.5, 125.2, 123.2 (q, *J* = 273.0 Hz), 122.8, 122.7 (d, *J* = 3.7 Hz), 81.3, 43.6, 38.5.

^{19}F NMR (376 MHz, $CDCl_3$): δ -62.6

9b-(2,4-dimethoxyphenyl)-2,3-dihydrothiazolo[2,3-a]isoindol-5-one (2g)

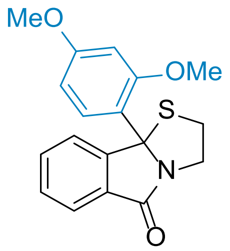

The title compound was prepared according to general procedure **C** from 3-(2,4-dimethoxyphenyl)-3-hydroxy-2-(2-sulfanylethyl)isoindolin-1-one (100 mg, 0.290 mmol), $Ca(NTf_2)_2$ (1.7 mg, 0.00290 mmol) and nBu_4NPF_6 (1.1 mg, 0.00290 mmol) in HFIP (1.5 mL). Following completion of the reaction (20 mins), purification by FCC (1:1 EtOAc:Hex) afforded the pure compound as white solid (90 mg, 95%).

RF (1:1 EtOAc:Hex): 0.60

IR ν_{max} (cm^{-1}): 3070, 2933, 2835, 1698, 1343, 1267, 1023

HRMS (APCI)m/z: $[M + H]^+$ Calcd for $C_{18}H_{18}NO_3S$ 328.1007; Found 328.1003

^1H NMR (400 MHz, $CDCl_3$): δ 7.87 (d, *J* = 7.7 Hz, 1H), 7.74 (d, *J* = 7.7 Hz, 1H), 7.47 (td, *J* = 7.6, 1.3 Hz, 1H), 7.40 (td, *J* = 7.5, 0.9 Hz, 1H), 7.35 (d, *J* = 8.6 Hz, 1H), 6.59 (d, *J* = 2.4 Hz, 1H), 6.39 (dd, *J* = 8.6, 2.4 Hz, 1H), 4.75 (ddd, *J* = 11.6, 6.4, 1.6 Hz, 1H), 4.00 (s, 3H), 3.79 (s, 3H), 3.38 – 3.30 (m, 1H), 3.28 – 3.20 (m, 1H), 3.07 (ddd, *J* = 10.0, 5.9, 1.6 Hz, 1H).

^{13}C NMR (101 MHz, $CDCl_3$): δ 171.6, 161.1, 158.1, 152.0, 133.4, 128.9, 128.6, 124.9, 124.1, 123.6, 123.00, 104.2, 100.8, 79.00, 55.9, 55.6, 44.4, 36.4.

9b-(1,3-benzodioxol-5-yl)-2,3-dihydrothiazolo[2,3-a]isoindol-5-one (2h)

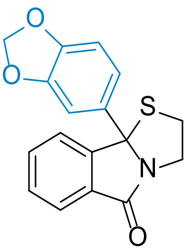

The title compound was prepared according to general procedure **C** from 3-(1,3-benzodioxol-5-yl)-3-hydroxy-2-(2-sulfanylethyl)isoindolin-1-one (50 mg, 0.152 mmol), $Ca(NTf_2)_2$ (1.0 mg, 0.00152 mmol) and nBu_4NPF_6 (0.6 mg, 0.00152 mmol) in HFIP (0.8 mL). Following completion of the reaction (15 mins), purification by FCC (1:1 EtOAc:Hex) afforded the pure compound as white solid (39 mg, 83%).

RF (1:1 EtOAc:Hex): 0.52

IR ν_{max} (cm^{-1}): 3060, 2939, 1702, 1608, 1485, 1243, 1029, 746

HRMS (APCI)m/z: $[M + H]^+$ Calcd for $C_{17}H_{14}NO_3S$ 312.0694; Found 312.0703

^1H NMR (400 MHz, $CDCl_3$): δ 7.81 – 7.77 (m, 1H), 7.50 (td, *J* = 7.5, 1.4 Hz, 1H), 7.44 (td, *J* = 7.4, 1.2 Hz, 1H), 7.31 – 7.27 (m, 1H), 7.17 (dd, *J* = 8.2, 1.9 Hz, 1H), 7.07 (d, *J* = 1.9 Hz, 1H), 6.77 (d, *J* = 8.1 Hz, 1H), 5.96 (dd, *J* = 6.7, 1.4 Hz, 2H), 4.56 – 4.40 (m, 1H), 3.61 – 3.47 (m, 1H), 3.42 – 3.29 (m, 2H).

^{13}C NMR (101 MHz, $CDCl_3$): δ 171.0, 149.7, 148.2, 147.9, 134.3, 133.1, 129.1, 128.9, 124.6, 122.9, 119.9, 108.2, 107.0, 101.6, 82.1, 43.1, 38.4.

9b-(4-morpholinophenyl)-2,3-dihydrothiazolo[2,3-a]isoindol-5-one (2i)

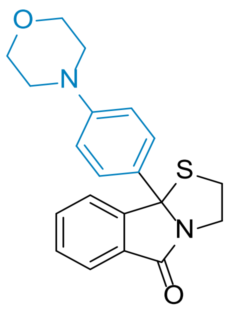

The title compound was prepared according to general procedure **C** from 3-hydroxy-3-(4-morpholinophenyl)-2-(2-sulfanylethyl)isoindolin-1-one (30 mg, 0.081 mmol), $Ca(NTf_2)_2$ (0.5

mg, 0.00081 mmol) and nBu_4NPF_6 (0.3 mg, 0.00081 mmol) in HFIP (0.4 mL). The reaction was stirred overnight and purification by FCC (1:5 EtOAc:DCM) afforded the pure compound as a colourless oil (8 mg, 28%).

RF (1:1 EtOAc:Hex): 0.43

IR ν_{max} (cm^{-1}): 3062, 2934, 2843, 1698, 1247, 1032

HRMS (APCI)m/z: $[M + H]^+$ Calcd for $C_{20}H_{21}N_2O_2S$ 353.1324; Found 353.1325

^1H NMR (400 MHz, $CDCl_3$): δ 7.81 (d, *J* = 6.9 Hz, 1H), 7.61 – 7.37 (m, 4H), 7.31 (d, *J* = 7.4 Hz, 1H), 6.89 (d, *J* = 8.9 Hz, 2H), 4.58 – 4.42 (m, 1H), 3.91 – 3.79 (m, 4H), 3.63 – 3.48 (m, 1H), 3.43 – 3.28 (m, 2H), 3.19 (dd, *J* = 5.6, 4.1 Hz, 4H).

^{13}C NMR (101 MHz, $CDCl_3$): δ 171.1, 151.3, 150.0, 133.1, 132.2, 129.0, 128.7, 127.3, 124.5, 123.0, 115.3, 82.0, 67.0, 49.0, 43.1, 38.3.

Carbon Nucleophiles - Indole

Synthesis of 2-[2-(1H-indol-3-yl)ethyl]isoindoline-1,3-dione (3)

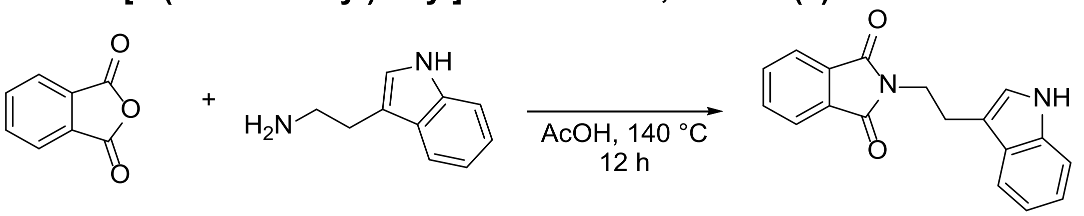

Phthalic anhydride (1.0 g, 6.75 mmol) and tryptamine (1.3 g, 8.10 mmol) was dissolved in acetic acid (14 mL) and stirred at 140 °C overnight. Upon completion of the reaction, indicated by TLC, the mixture was cooled, diluted with water (50 mL) and quenched slowly with sat. aq. $NaHCO_3$. The solution was transferred to a separating funnel and extracted into DCM (3 x 50 mL). The combined organic layers were dried over Na_2SO_4 and concentrated. Purification by FCC (1:3 EtOAc:Hex) afforded the title compound as a yellow solid (1.6g, 81%).

RF (1:1 EtOAc:Hex): 0.71

^1H NMR (400 MHz, $CDCl_3$): δ 7.99 (s, 1H), 7.84 (dd, *J* = 5.4, 3.0 Hz, 2H), 7.77 – 7.68 (m, 3H), 7.35 (d, *J* = 8.0 Hz, 1H), 7.22 – 7.16 (m, 1H), 7.16 – 7.09 (m, 2H), 4.07 – 3.96 (m, 2H), 3.21 – 3.12 (m, 2H).

^{13}C NMR (101 MHz, $CDCl_3$): δ 168.5, 136.4, 134.0, 132.4, 127.6, 123.3, 122.3, 122.1, 119.7, 119.0, 112.6, 111.2, 38.7, 24.6.

*Data in accordance with literature[2]

Starting Materials – Indole

3-hydroxy-2-[2-(1H-indol-3-yl)ethyl]-3-phenyl-isoindolin-1-one (3a)

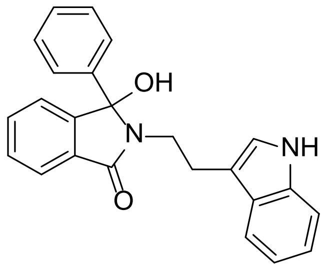

The title compound was prepared according to general procedure **A** from 2-[2-(1H-indol-3-yl)ethyl]isoindoline-1,3-dione (300 mg, 1.03 mmol), Phenylmagnesium bromide (3.0 M in Et_2O, 1.03 mL, 3.10 mmol) in DCM (4 mL). Following completion of the reaction (1 h), purification by FCC (1:1 EtOAc:Hex) afforded the pure product as a white solid (250 mg, 66%).

RF (1:1 EtOAc:Hex): 0.31

IR ν_{max} (cm^{-1}): 3368, 3230, 1657, 1614, 1414, 1198, 1055, 850

HRMS (APCI)m/z: $[M – H_2O]^+$ Calcd for $C_{24}H_{19}N_2O$ 351.1497; Found 351.1509

^1H NMR (400 MHz, DMSO-d_6): δ 10.78 (s, 1H), 7.79 – 7.74 (m, 1H), 7.55 (pd, *J* = 7.4, 1.4 Hz, 2H), 7.44 – 7.34 (m, 5H), 7.34 – 7.26 (m, 3H), 7.20 (s, 1H), 7.10 (d, *J* = 2.3 Hz, 1H), 7.07 – 7.01 (m, 1H), 6.95 (ddd, *J* = 7.9, 7.1, 1.0 Hz, 1H), 3.61 (ddd, *J* = 13.5, 12.0, 5.1 Hz, 1H), 3.19 (ddd, *J* = 13.6, 12.0, 5.0 Hz, 1H), 3.04 – 2.92 (m, 1H), 2.80 – 2.66 (m, 1H).

^{13}C NMR (101 MHz, $CDCl_3$): δ 168.1, 149.2, 138.7, 136.3, 132.8, 130.7, 129.6, 128.7, 128.6, 127.4, 126.4, 123.3, 122.8, 122.1, 122.1, 119.4, 119.1, 113.5, 111.2, 91.5, 40.5, 24.5.

3-hydroxy-2-[2-(1H-indol-3-yl)ethyl]-3-[4-(trifluoromethyl)phenyl]isoindolin-1-one (3b)

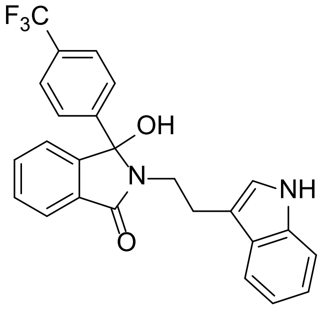

The title compound was prepared according to general procedure **A** from 2-[2-(1H-indol-3-yl)ethyl]isoindoline-1,3-dione (250 mg, 0.86 mmol), (4-(trifluoromethyl)phenyl)magnesium bromide (0.5 M in THF, 6.2 mL, 2.60 mmol) in DCM (4 mL). The Grignard reagent was freshly prepared from magnesium turnings (65 mg, 2.68 mmol), 4-bromobenzotrifluoride

(581 mg, 0.39 mL, 2.60 mmol) and 1,2-dibromoethane (7 µL, 0.0086 mmol) in THF (3 mL). Following completion of the reaction (30 mins), purification by FCC (1:1 EtOAc:Hex) afforded the pure product as a pale-yellow solid (250 mg, 67%).

RF (1:1 EtOAc:Hex): 0.27

IR ν_{max} (cm^{-1}): 3342, 3056, 2939, 1659, 1612, 1321, 1066, 822

HRMS (APCI)m/z: $[M – H_2O]^+$ Calcd for $C_{25}H_{18}F_3N_2O$ 419.1371; Found 419.1382

^1H NMR (400 MHz, DMSO-d_6): δ 10.78 (s, 1H), 7.81 – 7.77 (m, 1H), 7.73 (d, *J* = 8.2 Hz, 2H), 7.62 (d, *J* = 8.1 Hz, 2H), 7.60 – 7.55 (m, 2H), 7.44 (s, 1H), 7.35 – 7.26 (m, 3H), 7.12 (d, *J* = 2.3 Hz, 1H), 7.07 – 7.00 (m, 1H), 6.96 – 6.89 (m, 1H), 3.60 (ddd, *J* = 13.6, 11.8, 5.2 Hz, 1H), 3.19 (ddd, *J* = 13.7, 11.8, 5.1 Hz, 1H), 3.06 – 2.91 (m, 1H), 2.81 – 2.65 (m, 1H).

^{13}C NMR (101 MHz, $CDCl_3$): δ 166.7, 149.0, 145.0, 136.2, 132.7, 130.6, 129.6, 128.7 (q, *J* = 32.1 Hz), 127.0, 126.9, 125.5 (q, *J* = 3.4 Hz), 122.8, 122.6, 120.9, 118.2, 117.9, 111.4, 111.3, 90.2, 24.5. (one resonance missing)

^{19}F NMR (376 MHz, CDCl3): δ -61.1

3-(4-bromophenyl)-3-hydroxy-2-[2-(1H-indol-3-yl)ethyl]isoindolin-1-one (3c)

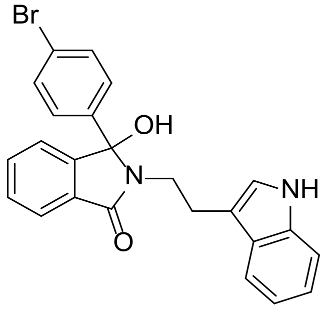

The title compound was prepared according to general procedure **A** from 2-[2-(1H-indol-3-yl)ethyl]isoindoline-1,3-dione (250 mg, 0.86 mmol), 4-bromophenylmagnesium bromide (0.5 M in THF, 6.2 mL, 2.60 mmol) in DCM (4 mL). The Grignard reagent was freshly prepared from magnesium turnings (65 mg, 2.68 mmol), 1,4-dibromobenzene (610 mg, 2.60 mmol) and 1,2-dibromoethane (7 µL, 0.0086 mmol) in THF (3 mL). Following completion of the reaction (30 mins), purification by FCC (1:3 EtOAc:Hex) afforded the pure product as a pale-yellow solid (248 mg, 64%).

RF (1:1 EtOAc:Hex): 0.33

IR ν_{max} (cm^{-1}): 3330, 3068, 1657, 1610, 1403, 1068, 805

HRMS (APCI)m/z: $[M – H_2O]^+$ Calcd for $C_{24}H_{18}BrN_2O$ 429.0603; Found 429.0611

^1H NMR (400 MHz, DMSO-d_6): δ 10.78 (s, 1H), 7.80 – 7.73 (m, 1H), 7.64 – 7.48 (m, 4H), 7.45 – 7.21 (m, 6H), 7.11 (d, *J* = 2.3 Hz, 1H), 7.08 – 7.01 (m, 1H), 6.99 – 6.92 (m, 1H), 3.58 (ddd, *J* = 13.6, 11.9, 5.2 Hz, 1H), 3.18 (ddd, *J* = 13.7, 11.8, 5.0 Hz, 1H), 3.03 – 2.88 (m, 1H), 2.81 – 2.69 (m, 1H).

^{13}C NMR (101 MHz, DMSO-d_6): δ 166.6, 149.2, 139.8, 136.2, 132.6, 131.4, 130.6, 129.4, 128.5, 128.3, 126.9, 122.8, 122.6, 122.5, 121.5, 121.0, 118.3, 118.0, 111.4, 90.2, 24.6. (one resonance missing)

3-[3,5-bis(trifluoromethyl)phenyl]-3-hydroxy-2-[2-(1H-indol-3-yl)ethyl]isoindolin-1-one (3d)

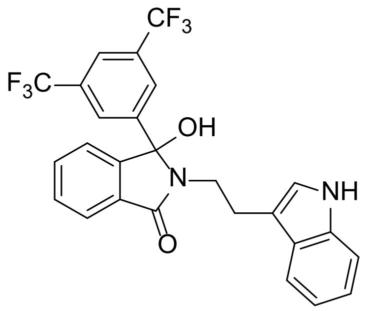

The title compound was prepared according to general procedure **A** from 2-[2-(1H-indol-3-yl)ethyl]isoindoline-1,3-dione (250 mg, 0.86 mmol), 3,5-bis(trifluoromethyl)-phenylmagnesium bromide (0.5 M in THF, 6.2 mL, 2.60 mmol) in DCM (4 mL). The Grignard reagent was freshly prepared from magnesium turnings (65 mg, 2.68 mmol), 1,3-bis(trifluoromethyl)-5-bromobenzene (757 mg, 0.45 mL, 2.60 mmol) and 1,2-dibromoethane (7 µL, 0.0086 mmol) in THF (3 mL). Following completion of the reaction (1 h), purification by FCC (1:3 EtOAc:Hex) afforded the pure product as an off-white solid (122 mg, 28%).

RF (1:1 EtOAc:Hex): 0.19

IR ν_{max} (cm^{-1}): 3465, 3263, 1668, 1619, 1364, 1277, 1131, 900

HRMS (APCI)m/z: $[M – H_2O]^+$ Calcd for $C_{26}H_{17}F_6N_2O$ 487.1245; Found 487.1258

^1H NMR (400 MHz, $CDCl_3$): δ 7.94 (s, 1H), 7.89 – 7.79 (m, 4H), 7.57 – 7.50 (m, 2H), 7.46 (d, *J* = 8.1 Hz, 1H), 7.34 (d, *J* = 8.1 Hz, 1H), 7.26 – 7.21 (m, 1H), 7.21 – 7.16 (m, 1H), 7.12 – 7.05 (m, 1H), 6.99 (d, *J* = 2.3 Hz, 1H), 3.96 – 3.80 (m, 1H), 3.27 (ddd, *J* = 13.8, 9.2, 6.4 Hz, 1H), 3.22 – 3.12 (m, 1H), 3.11 (s, 1H), 2.93 (ddd, *J* = 14.4, 9.1, 5.7 Hz, 1H).

^{13}C NMR (101 MHz, DMSO-d_6): δ 166.8, 148.1, 144.1, 136.2, 133.0, 130.7 (q, *J* = 33.1 Hz), 130.6, 130.0, 126.8, 126.7, 126.7, 123.1 (q, *J* = 273.1 Hz), 122.9, 122.9, 122.7, 122.5 (q, *J* = 3.6 Hz), 121.0, 118.2, 117.7, 111.5, 111.0, 89.6, 24.5.

^{19}F NMR (376 MHz, $CDCl_3$): δ -62.8

3-hydroxy-2-[2-(1H-indol-3-yl)ethyl]-3-(4-methoxyphenyl)isoindolin-1-one (3e)

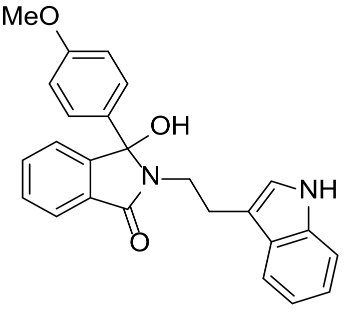

The title compound was prepared according to general procedure **A** from 2-[2-(1H-indol-3-yl)ethyl]isoindoline-1,3-dione (250 mg, 0.86 mmol), 4-methoxyphenylmagnesium bromide (0.5 M in THF, 6.2 mL, 2.60 mmol) in DCM (4 mL). The Grignard reagent was freshly

prepared from magnesium turnings (65 mg, 2.68 mmol), 4-bromoanisole (483 mg, 0.32 mL, 2.60 mmol) and 1,2-dibromoethane (7 µL, 0.0086 mmol) in THF (3 mL). Following completion of the reaction (30 mins), purification by FCC (1:1 EtOAc:Hex) afforded the pure product as a white solid (150 mg, 44%).

RF (1:1 EtOAc:Hex): 0.20

IR ν_{max} (cm^{-1}): 3364, 3010, 2919, 1677, 1605, 1407, 1019, 828

HRMS (APCI)m/z: $[M – H_2O]^+$ Calcd for $C_{25}H_{22}N_2O_2$ 381.1603; Found 381.1590

^{1}H NMR (400 MHz, DMSO-d_6): δ 10.78 (s, 1H), 7.79 – 7.72 (m, 1H), 7.54 (dqd, *J* = 14.4, 7.4, 1.2 Hz, 2H), 7.40 (d, *J* = 7.8 Hz, 1H), 7.36 – 7.24 (m, 4H), 7.15 – 7.09 (m, 2H), 7.09 – 7.02 (m, 1H), 6.98 – 6.88 (m, 3H), 3.72 (s, 3H), 3.68 – 3.53 (m, 1H), 3.30 – 3.12 (m, 1H), 3.07 – 2.92 (m, 1H), 2.75 (td, *J* = 13.4, 5.0 Hz, 1H).

^{13}C NMR (101 MHz, DMSO-d_6): δ 166.6, 159.1, 149.9, 136.3, 132.5, 132.0, 130.7, 129.1, 127.3, 127.0, 122.8, 122.6, 122.4, 121.0, 118.3, 118.2, 113.8, 111.6, 111.4, 90.6, 55.1, 24.6. (one resonance missing)

3-(2,4-dimethoxyphenyl)-3-hydroxy-2-[2-(1H-indol-3-yl)ethyl]isoindolin-1-one (3f)

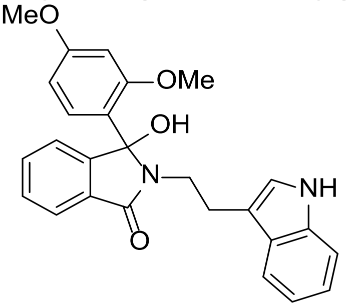

The title compound was prepared according to general procedure **A** from 2-[2-(1H-indol-3-yl)ethyl]isoindoline-1,3-dione (250 mg, 0.86 mmol), 2,4-dimethoxyphenylmagnesium bromide (0.5 M in THF, 6.2 mL, 2.60 mmol) in DCM (4 mL). The Grignard reagent was freshly prepared from magnesium turnings (65 mg, 2.68 mmol), 1-bromo-2,4-dimethoxybenzene (561 mg, 0.37 mL, 2.60 mmol) and 1,2-dibromoethane (7 µL, 0.0086 mmol) in THF (3 mL). Following completion of the reaction (1 h), purification by FCC (1:1 EtOAc:Hex) afforded the pure product as an off-white solid (350 mg, 95%).

RF (1:1 EtOAc:Hex): 0.21

IR ν_{max} (cm^{-1}): 3293, 2935, 2835, 1655, 1435, 1029, 759

HRMS (APCI)m/z: $[M – H_2O]^+$ Calcd for $C_{26}H_{23}N_2O_3$ 411.1709; Found 411.1698

^{1}H NMR (400 MHz, DMSO-d_6): δ 10.74 (s, 1H), 7.99 (d, *J* = 8.7 Hz, 1H), 7.71 – 7.65 (m, 1H), 7.51 – 7.42 (m, 2H), 7.29 (d, *J* = 8.1 Hz, 1H), 7.20 (d, *J* = 7.9 Hz, 1H), 7.19 – 7.12 (m, 1H), 7.07 (d, *J* = 2.3 Hz, 1H), 7.06 – 6.99 (m, 1H), 6.95 – 6.88 (m, 1H), 6.81 (s, 1H), 6.69 (dd, *J* = 8.7, 2.4 Hz, 1H), 6.41 (d, *J* = 2.4 Hz, 1H), 3.76 (s, 3H), 3.55 – 3.37 (m, 1H), 3.24 (s, 3H), 3.21 – 3.05 (m, 1H), 3.02 – 2.86 (m, 1H), 2.61 – 2.52 (m, 1H).

^{13}C NMR (101 MHz, DMSO-d_6): δ 167.0, 161.0, 157.3, 149.6, 136.2, 132.3, 131.5, 129.4, 128.4, 126.9, 122.5, 121.7, 121.6, 120.9, 119.4, 118.1, 118.1, 111.7, 111.4, 104.5, 99.2, 88.3, 79.2, 55.4, 55.2, 24.0.

3-(2-furyl)-3-hydroxy-2-[2-(1H-indol-3-yl)ethyl]isoindolin-1-one (3g)

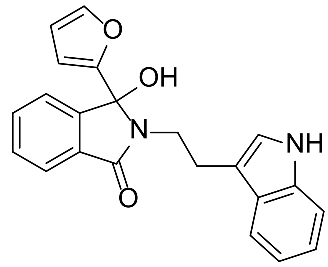

Furan (0.23 mL, 3.10 mmol) was dissolved in dry THF (7 mL). n-buthyllithium (2.5 M in Hexane, 1.03 mL, 2.60 mmol) was added dropwise at -78 °C and the mixture was stirred at room temperature for 2h. The solution was then cooled to -78 ºC and 2-[2-(1H-indol-3-yl)ethyl]isoindoline-1,3-dione (250 mg, 0.86 mmol) was added in a single porition. The reaction mixture was allowed to warm to room temperature and stirred for 2h. Upon completion of the reaction, indicated by TLC, the reaction mixture was quenched with sat. NH_4Cl and extracted with DCM (3 x 25 mL). The combined organic layers were dried over Na_2SO_4 and concentrated. Purification by FCC (1:1 EtOAc:Hex) afforded the pure product as a yellow solid (167 mg, 54 %).

RF (1:1 EtOAc:Hex): 0.17

IR ν_{max} (cm^{-1}): 3412, 3293, 1661, 1410, 1152, 818, 742

HRMS (APCI)m/z: $[M – H_2O]^+$ Calcd for $C_{22}H_{17}N_2O_2$ 341.1290; Found 341.1301

^1H NMR (400 MHz, $CDCl_3$): δ 7.95 (s, 1H), 7.85 – 7.79 (m, 1H), 7.61 (d, *J* = 7.8 Hz, 1H), 7.56 – 7.46 (m, 2H), 7.43 – 7.38 (m, 1H), 7.36 – 7.32 (m, 1H), 7.32 (dd, *J* = 1.8, 0.9 Hz, 1H), 7.20 – 7.15 (m, 1H), 7.13 – 7.07 (m, 1H), 7.04 (d, *J* = 2.3 Hz, 1H), 6.60 (dd, *J* = 3.3, 0.9 Hz, 1H), 6.39 (dd, *J* = 3.3, 1.8 Hz, 1H), 3.87 (ddd, *J* = 14.0, 10.1, 5.3 Hz, 1H), 3.49 (ddd, *J* = 14.0, 10.0, 6.3 Hz, 1H), 3.14 (ddd, *J* = 14.2, 9.8, 6.2 Hz, 1H), 2.86 – 2.77 (m, 1H), 2.77 (s, 1H).

^{13}C NMR (101 MHz, $CDCl_3$): δ 167.6, 150.8, 146.2, 143.2, 136.3, 132.7, 131.0, 130.2, 127.5, 123.5, 122.7, 122.2, 122.2, 119.5, 119.2, 113.6, 111.2, 110.8, 109.4, 88.1, 40.4, 24.4.

3-hydroxy-2-[2-(1H-indol-3-yl)ethyl]-3-(6-methoxy-2-pyridyl)isoindolin-1-one (3h)

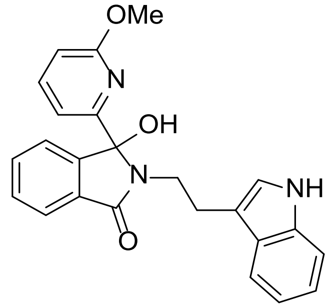

The title compound was prepared according to general procedure **B** from 2-[2-(1H-indol-3-yl)ethyl]isoindoline-1,3-dione (250 mg, 0.86 mmol), 2-bromo-6-methoxypyridine (650 mg, 0.47 mL, 3.44 mmol) and n-butyllithium (2.5 M in Hexane, 1.21 mL, 3.01 mmol) in THF (6 mL). Following completion of the reaction (1 h), purification by FCC (1:1 EtOAc:Hex) afforded the pure product as a pale-yellow solid (186 mg, 54%).

RF (1:1 EtOAc:Hex): 0.23

IR ν_{max} (cm^{-1}): 3300, 2932, 2861, 1675, 1575, 1269, 800

HRMS (APCI)m/z: $[M - H_2O]^+$ Calcd for $C_{24}H_{20}N_3O_2$ 382.1556; Found 382.1548

^1H NMR (400 MHz, $CDCl_3$): δ δ 8.02 – 7.77 (m, 2H), 7.55 – 7.40 (m, 4H), 7.32 (d, *J* = 8.1 Hz, 1H), 7.25 – 7.22 (m, 1H), 7.20 – 7.13 (m, 1H), 7.11 – 7.05 (m, 1H), 7.02 (d, *J* = 2.3 Hz, 1H), 6.76 – 6.69 (m, 1H), 6.52 (dd, *J* = 7.4, 0.6 Hz, 1H), 6.47 (s, 1H), 4.07 (s, 3H), 3.74 (ddd, *J* = 13.9, 11.0, 5.6 Hz, 1H), 3.39 (ddd, *J* = 14.0, 11.0, 5.4 Hz, 1H), 3.18 – 3.02 (m, 1H), 2.89 (ddd, *J* = 14.2, 10.9, 5.2 Hz, 1H).

^{13}C NMR (101 MHz, $CDCl_3$): δ 168.2, 163.1, 154.8, 147.9, 140.6, 136.3, 132.6, 131.8, 129.8, 127.5, 123.4, 122.5, 122.1, 122.0, 119.3, 118.9, 113.6, 113.5, 111.2, 111.1, 89.8, 54.0, 40.4, 24.8.

3-(1,3-benzodioxol-5-yl)-3-hydroxy-2-[2-(1H-indol-3-yl)ethyl]isoindolin-1-one (3i)

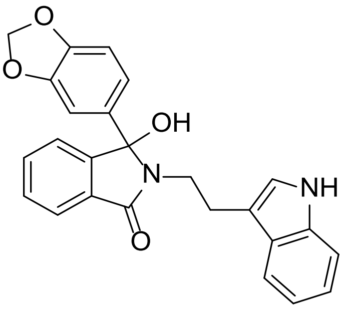

The title compound was prepared according to general procedure **B** from 2-[2-(1H-indol-3-yl)ethyl]isoindoline-1,3-dione (250 mg, 0.86 mmol), 1-bromo-3,4-(methylenedioxy)benzene (692 mg, 0.42 mL, 3.44 mmol) and n-butyllithium (2.5 M in Hexane, 1.21 mL, 3.01 mmol) in THF (7 mL). Following completion of the reaction (1 h), purification by FCC (1:3 EtOAc:DCM) afforded the pure product as a light-brown solid (271 mg, 76%).

RF (1:1 EtOAc:Hex): 0.23

IR ν_{max} (cm^{-1}): 3314, 3056, 2898, 1661, 1405, 1239, 1036, 766

HRMS (APCI)m/z: $[M - H_2O]^+$ Calcd for $C_{25}H_{19}N_2O_3$ 395.1396; Found 395.1408

^1H NMR (400 MHz, DMSO-d_6): δ 10.78 (s, 1H), 7.79 – 7.70 (m, 1H), 7.57 (td, *J* = 7.4, 1.4 Hz, 1H), 7.52 (td, *J* = 7.4, 1.2 Hz, 1H), 7.42 (d, *J* = 7.8 Hz, 1H), 7.35 – 7.27 (m, 2H), 7.15 (s, 1H), 7.12 (s, 1H), 7.09 – 7.01 (m, 1H), 7.00 – 6.92 (m, 1H), 6.92 – 6.83 (m, 3H), 5.99 (dd, *J* = 4.7, 0.9 Hz, 2H), 3.60 (ddd, *J* = 13.5, 11.9, 5.2 Hz, 1H), 3.26 – 3.13 (m, 1H), 3.08 – 2.88 (m, 1H), 2.88 – 2.68 (m, 1H).

^{13}C NMR (101 MHz, DMSO-d_6): δ 166.5, 149.7, 147.4, 147.1, 136.3, 134.1, 132.4, 130.6, 129.2, 127.0, 122.7, 122.6, 122.4, 121.0, 119.5, 118.2, 118.1, 111.6, 111.4, 108.1, 106.4, 101.2, 90.5, 39.9, 24.6.

Indole Products

2-phenyl-10,20-diazapentacyclo[11.7.0.02,10.03,8.014,19]icosa-1(13),3,5,7,14(19),15,17-heptaen-9-one (4a)

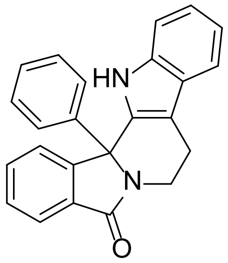

The title compound was prepared according to general procedure **C** from 3-hydroxy-2-[2-(1H-indol-3-yl)ethyl]-3-phenyl-isoindolin-1-one (80 mg, 0.22 mmol), $Ca(NTf_2)_2$ (1.3 mg, 0.0022 mmol) and nBu_4NPF_6 (0.8 mg, 0.0022 mmol) in HFIP (1.1 mL). Following completion of the reaction (1 h), purification by FCC (1:1 EtOAc:Hex) afforded the pure compound as a yellow solid (68 mg, 89%).

RF (1:1 EtOAc:Hex): 0.47

IR ν_{max} (cm^{-1}): 3261, 3058, 2935, 1659, 1394, 1448, 932

HRMS (APCI)m/z: $[M + H]^+$ Calcd for $C_{24}H_{19}N_2O$ 351.1497; Found 351.1510

^{1}H NMR (400 MHz, $CDCl_3$): δ δ 8.20 (s, 1H), 7.98 – 7.89 (m, 1H), 7.64 – 7.56 (m, 1H), 7.55 – 7.49 (m, 3H), 7.38 (dt, *J* = 8.1, 0.9 Hz, 1H), 7.32 – 7.20 (m, 4H), 7.17 – 7.11 (m, 1H), 7.03 – 6.97 (m, 2H), 4.68 – 4.56 (m, 1H), 3.24 – 3.03 (m, 2H), 2.99 – 2.79 (m, 1H).

^{13}C NMR (101 MHz, $CDCl_3$): δ 168.1, 148.7, 140.3, 136.5, 132.5, 132.2, 132.0, 129.2, 128.8, 128.7, 127.9, 126.8, 124.7, 123.1, 122.7, 120.3, 119.1, 111.3, 111.2, 68.0, 35.1, 21.7.

2-[4-(trifluoromethyl)phenyl]-10,20-diazapentacyclo[11.7.0.02,10.03,8.014,19]icosa-1(13),3,5,7,14(19),15,17-heptaen-9-one (4b)

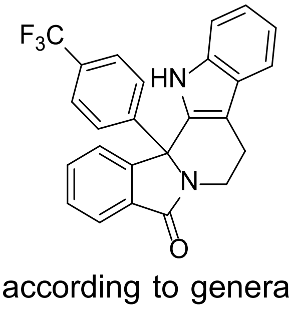

The title compound was prepared according to general procedure **C** from 3-hydroxy-2-[2-(1H-indol-3-yl)ethyl]-3-[4-(trifluoromethyl)phenyl]isoindolin-1-one (120 mg, 0.28 mmol), $Ca(NTf_2)_2$ (1.7 mg, 0.0028 mmol) and nBu_4NPF_6 (1.1 mg, 0.0028 mmol) in HFIP (1.4 mL). Following completion of the reaction (1 h), purification by FCC (1:1 EtOAc:Hex) afforded the pure compound as a white solid (100 mg, 87%).

RF (1:1 EtOAc:Hex): 0.60

IR ν_{max} (cm^{-1}): 3258, 3014, 2939, 1664, 1320, 1113, 742

HRMS (APCI)m/z: $[M + H]^+$ Calcd for $C_{25}H_{18}F_3N_2O$ 419.1371; Found 419.1380

^{1}H NMR (400 MHz, $CDCl_3$): δ 8.15 (s, 1H), 7.96 (d, *J* = 7.3 Hz, 1H), 7.62 (td, *J* = 7.5, 1.2 Hz, 1H), 7.58 – 7.46 (m, 4H), 7.39 (d, *J* = 8.1 Hz, 1H), 7.24 (dd, *J* = 8.1, 1.1 Hz, 2H), 7.20 – 7.12 (m, 3H), 4.71 – 4.59 (m, 1H), 3.18 – 3.02 (m, 2H), 2.97 – 2.81 (m, 1H).

^{13}C NMR (101 MHz, DMSO-d_6): δ 167.1, 148.0, 145.2, 136.6, 132.8, 131.2, 130.8, 129.3, 128.9 (q, *J* = 32.0 Hz), 128.1, 125.8 – 125.6 (m), 124.0, 124.0 (q, *J* = 272.3 Hz), 123.5, 122.2, 119.2, 118.6, 111.5, 109.3, 79.2, 67.0, 35.0, 21.1.

^{19}F NMR (376 MHz, $CDCl_3$): δ -62.8

2-(4-bromophenyl)-10,20-diazapentacyclo[11.7.0.02,10.03,8.014,19]icosa-1(13),3,5,7,14(19),15,17-heptaen-9-one (4c)

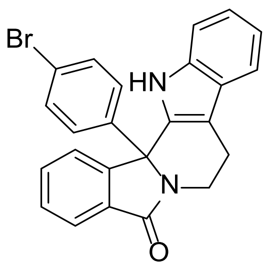

The title compound was prepared according to general procedure **C** from 3-(4-bromophenyl)-3-hydroxy-2-[2-(1H-indol-3-yl)ethyl]isoindolin-1-one (100 mg, 0.22 mmol), $Ca(NTf_2)_2$ (1.3 mg, 0.0022 mmol) and nBu_4NPF_6 (0.9 mg, 0.0022 mmol) in HFIP (1.1 mL). Following completion of the reaction (1 h), purification by FCC (1:1 EtOAc:Hex) afforded the pure compound as an off-white solid (90 mg, 94%).

RF (1:1 EtOAc:Hex): 0.57

IR ν_{max} (cm^{-1}): 3263, 3058, 2932, 1661, 1487, 1340, 941

HRMS (APCI)m/z: $[M + H]^+$ Calcd for $C_{24}H_{18}BrN_2O$ 429.0603; Found 429.0590

^{1}H NMR (400 MHz, $CDCl_3$): δ 8.23 (s, 1H), 7.98 – 7.87 (m, 1H), 7.63 – 7.57 (m, 1H), 7.56 – 7.49 (m, 3H), 7.43 – 7.33 (m, 3H), 7.25 – 7.20 (m, 1H), 7.19 – 7.11 (m, 1H), 6.87 (d, *J* = 8.7 Hz, 2H), 4.72 – 4.53 (m, 1H), 3.17 – 2.99 (m, 2H), 2.97 – 2.74 (m, 1H).

^{13}C NMR (101 MHz, $CDCl_3$): δ 168.0, 148.2, 139.5, 136.6, 132.7, 132.0, 131.9, 131.5, 129.7, 129.4, 126.7, 124.8, 123.2, 123.1, 122.6, 120.5, 119.2, 111.4, 111.3, 67.5, 35.1, 21.6.

2-[3,5-bis(trifluoromethyl)phenyl]-10,20-diazapentacyclo[11.7.0.02,10.03,8.014,19]icosa-1(13),3,5,7,14(19),15,17-heptaen-9-one (4d)

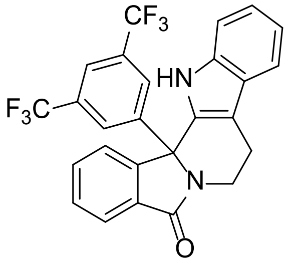

The title compound was prepared according to general procedure **C** from 3-[3,5-bis(trifluoromethyl)phenyl]-3-hydroxy-2-[2-(1H-indol-3-yl)ethyl]isoindolin-1-one (57 mg, 0.11 mmol), $Ca(NTf_2)_2$ (0.7 mg, 0.0011 mmol) and nBu_4NPF_6 (0.4 mg, 0.0011 mmol) in HFIP (0.6 mL). Following completion of the reaction (1 h), purification by FCC (1:1 EtOAc:Hex) afforded the pure compound as a white solid (50 mg, 91%).

RF (1:1 EtOAc:Hex): 0.57

IR ν_{max} (cm^{-1}): 3263, 3056, 2950, 1675, 1370, 1277, 1172, 902

HRMS (APCI)m/z: $[M + H]^+$ Calcd for $C_{26}H_{17}F_6N_2O$ 487.1245; Found 487.1256

^1H NMR (400 MHz, DMSO-d_6): δ ^1H NMR (400 MHz, DMSO) δ 11.61 (s, 1H), 8.19 (s, 1H), 8.08 (d, *J* = 7.7 Hz, 1H), 7.86 (d, *J* = 7.3 Hz, 1H), 7.75 (td, *J* = 7.6, 1.2 Hz, 1H), 7.63 (td, *J* = 7.5, 0.8 Hz, 1H), 7.57 (s, 2H), 7.50 (d, *J* = 7.9 Hz, 1H), 7.45 (d, *J* = 8.1 Hz, 1H), 7.21 – 7.14 (m, 1H), 7.09 – 7.02 (m, 1H), 4.55 – 4.43 (m, 1H), 3.10 – 2.97 (m, 1H), 2.97 – 2.78 (m, 2H).

^{13}C NMR (101 MHz, DMSO-d_6): δ 167.4, 147.4, 144.0, 136.6, 133.2, 130.8 (q, *J* = 33.0 Hz), 130.5, 130.2, 129.7, 127.3, 127.3, 125.6, 123.8, 122.9 (q, *J* = 273.1 Hz), 122.9 (q, *J* = 3.6 Hz), 122.6, 119.4, 118.9, 111.6, 109.8, 66.7, 35.2, 21.0.

^{19}F NMR (376 MHz, $CDCl_3$): δ -62.7

2-(4-methoxyphenyl)-10,20-diazapentacyclo[11.7.0.02,10.03,8.014,19]icosa-1(13),3,5,7,14(19),15,17-heptaen-9-one (4e)

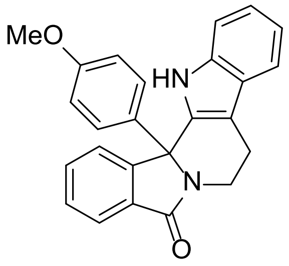

The title compound was prepared according to general procedure **C** from 3-hydroxy-2-[2-(1H-indol-3-yl)ethyl]-3-(4-methoxyphenyl)isoindolin-1-one (40 mg, 0.1 mmol), $Ca(NTf_2)_2$ (0.6 mg, 0.001 mmol) and nBu_4NPF_6 (0.4 mg, 0.001 mmol) in HFIP (0.5 mL). Following completion of the reaction (1 h), purification by FCC (1:1 EtOAc:Hex) afforded the pure compound as a pale yellow solid (35 mg, 92%).

RF (1:1 EtOAc:Hex): 0.45

IR ν_{max} (cm^{-1}): 3230, 2935, 2840, 1643, 1662, 1508, 1394, 1252, 740

HRMS (APCI)m/z: $[M + H]^+$ Calcd for $C_{25}H_{21}N_2O_2$ 381.1603; Found 381.1592

^1H NMR (400 MHz, $CDCl_3$): δ ^1H NMR (400 MHz, $CDCl_3$) δ 8.09 (s, 1H), 7.96 – 7.91 (m, 1H), 7.64 – 7.56 (m, 1H), 7.56 – 7.49 (m, 3H), 7.38 – 7.34 (m, 1H), 7.25 – 7.19 (m, 1H), 7.18 – 7.11 (m, 1H), 6.90 (d, *J* = 9.0 Hz, 2H), 6.76 (d, *J* = 9.0 Hz, 2H), 4.68 – 4.53 (m, 1H), 3.76 (s, 3H), 3.22 – 2.99 (m, 2H), 2.92 – 2.80 (m, 1H).

^{13}C NMR (101 MHz, $CDCl_3$): δ 167.9, 159.9, 148.8, 136.5, 132.4, 132.4, 132.2, 132.1, 129.3, 129.1, 126.8, 124.6, 123.0, 122.7, 120.3, 119.1, 114.0, 111.3, 111.1, 67.6, 55.5, 35.0, 21.7.

2-(2,4-dimethoxyphenyl)-10,20-diazapentacyclo[11.7.0.02,10.03,8.014,19]icosa-1(13),3,5,7,14(19),15,17-heptaen-9-one (4f)

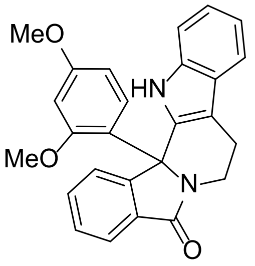

The title compound was prepared according to general procedure **C** from 3-(2,4-dimethoxyphenyl)-3-hydroxy-2-[2-(1H-indol-3-yl)ethyl]isoindolin-1-one (100 mg, 0.23 mmol), $Ca(NTf_2)_2$ (1.4 mg, 0.0023 mmol) and nBu_4NPF_6 (0.9 mg, 0.0023 mmol) in HFIP (1.2 mL). Following completion of the reaction (1 h), purification by FCC (1:1 EtOAc:Hex) afforded the pure compound as a white solid (67 mg, 70%).

RF (1:1 EtOAc:Hex): 0.35

IR ν_{max} (cm^{-1}): 3194, 3055, 2842, 1657, 1576, 1265, 939

HRMS (APCI)m/z: $[M + H]^+$ Calcd for $C_{26}H_{23}N_2O_3$ 411.1709; Found 2411.1715

^1H NMR (400 MHz, $CDCl_3$): δ 8.10 (s, 1H), 7.89 (dt, *J* = 7.5, 0.9 Hz, 1H), 7.56 – 7.47 (m, 3H), 7.47 – 7.40 (m, 1H), 7.35 (d, *J* = 8.1 Hz, 1H), 7.23 – 7.17 (m, 1H), 7.15 – 7.09 (m, 1H), 6.86 (d, *J* = 8.6 Hz, 1H), 6.38 – 6.29 (m, 2H), 4.70 – 4.59 (m, 1H), 3.76 (s, 3H), 3.30 (s, 3H), 3.24 – 3.02 (m, 2H), 2.91 – 2.74 (m, 1H).

^{13}C NMR (101 MHz, $CDCl_3$): δ 168.6, 161.7, 159.1, 149.6, 136.4, 133.0, 132.5, 132.0, 131.7, 128.1, 126.8, 124.1, 122.8, 121.1, 120.0, 119.9, 119.0, 111.3, 110.8, 103.8, 100.1, 66.9, 55.5, 55.4, 35.3, 21.7.

2-(2-furyl)-10,20-diazapentacyclo[11.7.0.02,10.03,8.014,19]icosa-1(13),3,5,7,14(19),15,17-heptaen-9-one (4g)

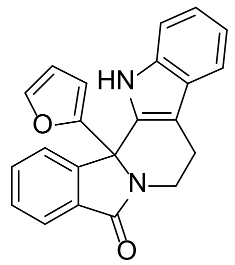

The title compound was prepared according to general procedure **C** from 3-(2-furyl)-3-hydroxy-2-[2-(1H-indol-3-yl)ethyl]isoindolin-1-one (100 mg, 0.28 mmol), $Ca(NTf_2)_2$ (1.7 mg, 0.0028 mmol) and nBu_4NPF_6 (1.1 mg, 0.0028 mmol) in HFIP (0.7 mL). Following completion of the reaction (1.5 h), purification by FCC (1:1 EtOAc:Hex) afforded the pure compound as an off-white solid (78 mg, 82%).

RF (1:1 EtOAc:Hex): 0.43

IR ν_{max} (cm^{-1}): 3256, 2920, 2846, 1655, 1398, 1146, 1014

HRMS (APCI)m/z: $[M + H]^+$ Calcd for $C_{22}H_{17}N_2O_2$ 341.1290; Found 341.1301

^1H NMR (400 MHz, $CDCl_3$): δ 8.40 (s, 1H), 7.91 (d, *J* = 7.5 Hz, 1H), 7.77 (d, *J* = 7.6 Hz, 1H), 7.61 (td, *J* = 7.5, 1.2 Hz, 1H), 7.51 (td, *J* = 7.5, 0.9 Hz, 2H), 7.38 (dt, *J* = 8.2, 0.8 Hz, 1H), 7.36 (dd, *J* = 1.8, 0.9 Hz, 1H), 7.21 (ddd, *J* = 8.2, 7.1, 1.2 Hz, 1H), 7.12 (ddd, *J* = 8.0, 7.1, 1.0 Hz, 1H), 6.29 (dd, *J* = 3.3, 1.9 Hz, 1H), 6.20 (dd, *J* = 3.3, 0.9 Hz, 1H), 4.80 – 4.71 (m, 1H), 3.41 (ddd, *J* = 13.4, 11.6, 4.9 Hz, 1H), 3.05 (ddd, *J* = 15.7, 11.6, 6.2 Hz, 1H), 2.93 – 2.81 (m, 1H).

^{13}C NMR (101 MHz, $CDCl_3$): δ 168.4, 152.2, 145.9, 143.7, 136.6, 132.4, 131.5, 131.0, 129.4, 126.6, 124.7, 123.1, 122.6, 120.2, 119.2, 111.4, 110.6, 110.5, 109.2, 63.7, 36.4, 21.8.

2-(6-methoxy-2-pyridyl)-10,20-diazapentacyclo[11.7.0.02,10.03,8.014,19]icosa-1(13),3,5,7,14(19),15,17-heptaen-9-one (4h)

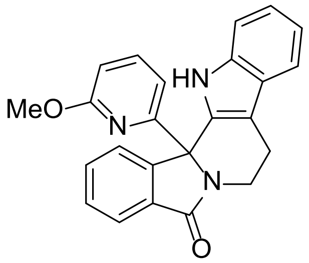

The title compound was prepared according to general procedure **C** from 3-hydroxy-2-[2-(1H-indol-3-yl)ethyl]-3-(6-methoxy-2-pyridyl)isoindolin-1-one (55 mg, 0.14 mmol), $Ca(NTf_2)_2$ (0.8 mg, 0.0014 mmol) and nBu_4NPF_6 (0.5 mg, 0.0014 mmol) in HFIP (0.7 mL). Following completion of the reaction (2 h), purification by FCC (1:1 EtOAc:Hex) afforded the pure compound as an off-white solid (49 mg, 93%).

RF (1:1 EtOAc:Hex): 0.49

IR $ν_{max}$ (cm^{-1}): 3324, 2946, 2892, 1655, 1573, 1415, 1267, 1033

HRMS (APCI)m/z: $[M + H]^+$ Calcd for $C_{24}H_{20}N_3O_2$ 382.1556; Found 382.1548

^1H NMR (400 MHz, $CDCl_3$): δ 9.47 (s, 1H), 8.13 (d, *J* = 7.7 Hz, 1H), 7.87 (d, *J* = 7.4 Hz, 1H), 7.59 – 7.36 (m, 5H), 7.23 – 7.15 (m, 1H), 7.14 – 7.05 (m, 1H), 6.99 (dd, *J* = 7.4, 0.6 Hz, 1H), 6.67 (dd, *J* = 8.3, 0.6 Hz, 1H), 4.97 (dd, *J* = 13.3, 5.3 Hz, 1H), 4.18 (s, 3H), 3.42 (ddd, *J* = 13.1, 11.7, 4.6 Hz, 1H), 3.08 (ddd, *J* = 15.6, 11.6, 5.9 Hz, 1H), 2.85 (dd, *J* = 15.5, 4.2 Hz, 1H).

^{13}C NMR (101 MHz, $CDCl_3$): δ 169.9, 164.3, 156.9, 147.7, 140.3, 136.8, 133.3, 132.3, 130.6, 128.9, 126.6, 124.4, 123.3, 122.7, 120.0, 119.0, 111.3, 110.7, 110.3, 109.8, 67.9, 53.7, 37.7, 22.0.

2-(1,3-benzodioxol-5-yl)-10,20-diazapentacyclo[11.7.0.02,10.03,8.014,19]icosa-1(13),3,5,7,14(19),15,17-heptaen-9-one (4i)

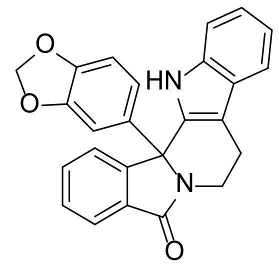

The title compound was prepared according to general procedure **C** from 3-(1,3-benzodioxol-5-yl)-3-hydroxy-2-[2-(1H-indol-3-yl)ethyl]isoindolin-1-one (100 mg, 0.24 mmol), $Ca(NTf_2)_2$ (1.5 mg, 0.0024 mmol) and nBu_4NPF_6 (0.9 mg, 0.0024 mmol) in HFIP (1.2 mL). The reaction was stirred overnight. Purification by FCC (1:1 EtOAc:Hex) afforded the pure compound as a pale yellow solid (20 mg, 21%).

RF (1:1 EtOAc:Hex): 0.43

IR $ν_{max}$ (cm^{-1}): 3252, 3056, 2920, 1659, 1485, 1398, 1236, 1111, 930

HRMS (APCI)m/z: $[M + H]^+$ Calcd for $C_{25}H_{19}N_2O_3$ 395.1396; Found 395.1405

^1H NMR (400 MHz, $CDCl_3$): δ 11.44 (s, 1H), 8.06 (d, *J* = 7.7 Hz, 1H), 7.80 (d, *J* = 7.3 Hz, 1H), 7.71 (td, *J* = 7.5, 1.2 Hz, 1H), 7.58 (td, *J* = 7.4, 0.9 Hz, 1H), 7.46 (d, *J* = 7.8 Hz, 1H), 7.40 (d, *J* = 8.1 Hz, 1H), 7.17 – 7.09 (m, 1H), 7.05 – 6.98 (m, 1H), 6.86 (d, *J* = 8.2 Hz, 1H), 6.46 (dd, *J* = 8.2, 1.9 Hz, 1H), 6.34 (d, *J* = 1.8 Hz, 1H), 5.99 (d, *J* = 0.7 Hz, 2H), 4.49 – 4.35 (m, 1H), 3.04 (ddd, *J* = 13.1, 10.4, 6.0 Hz, 2H), 2.92 – 2.75 (m, 1H).

^{13}C NMR (101 MHz, $CDCl_3$): δ 166.8, 148.6, 147.6, 147.3, 136.4, 134.3, 132.5, 132.1, 130.8, 129.0, 125.7, 124.0, 123.3, 122.1, 121.1, 119.0, 118.5, 111.4, 108.7, 108.0, 107.1, 101.4, 67.3, 34.7, 21.2.

Nitrogen Nucleophiles - Amides

(1,3-dioxo-1,3-dihydro-2*H*-isoindol-2-yl)acetic acid (Phthalimidoglycine)

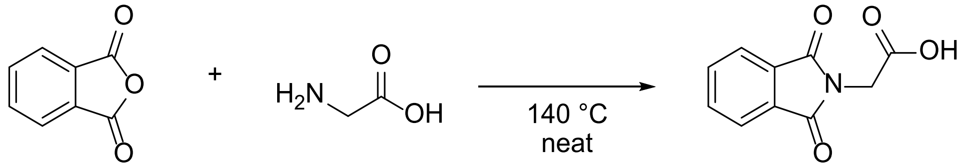

Glycine (1.05 g, 14.0 mmol, 1.0 equiv.) was added in one portion to phthalic anhydride (2.27 g, 15.4 mmol), 1.1 equiv.) at room temperature and heated at 140 °C for 30 minutes. The reaction mixture was cooled to room temperature and the resulting solid was purified by recrystallization (dissolved in hot IMS, followed by addition of H_2O) to give phthalimidoglycine (2.55 g, 12.4 mmol, 89%) as a colourless solid.

RF (9:1 DCM:MeOH): 0.05

^{1}H NMR (400 MHz, DMSO-d_6): δ 7.95 – 7.87 (m, 4H), 4.32 (s, 2H).

^{13}C NMR (101 MHz, DMSO-d_6): δ 168.9, 167.2, 134.8, 131.4, 123.4.

*Data in accordance with literature[3]

General Procedure D for Amide Coupling

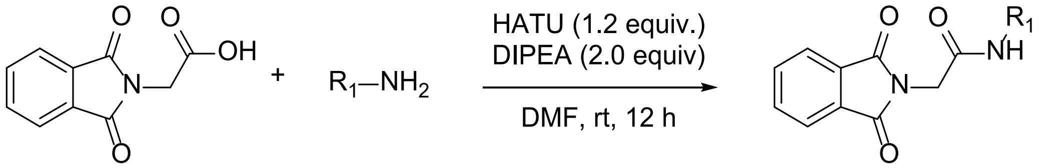

To an oven-dried vial, with a magnetic stirrer bar was added phthalimidoglycine (1.0 equiv.) and HATU (1.2 equiv.) which was sealed with a septum and then purged with N_2 and dissolved in dry DMF (0.2 M). DIPEA (2.0 equiv.) was then added and the reaction was stirred at room temperature for 5 mins after which the amine (1.1 equiv.) was added and the reaction was stirred at room temperature overnight. Following completion of the reaction, indicated by TLC (DCM:MeOH), the reaction was quenched with $NaHCO_3$ and transferred to a separating funnel which was then extracted into DCM (3 x 20 mL). The combined organic layers were then washed with water (3 x 50 mL) followed by 5% aq. LiCl (1 x 50 mL), dried over Na_2SO_4, filtered and concentrated. The product was then purified by flash column chromatography to afford the pure amide.

N-benzyl-2-(1,3-dioxoisoindolin-2-yl)acetamide

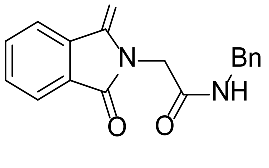

The title compound was prepared according the General Procedure **D** from phthalimidoglycine (1.0 g, 4.90 mmol), HATU (2.2 g, 5.90 mmol), DIPEA (1.70 mL, 9.80 mmol), and benzylamine (0.60 mL, 5.40 mmol) in DMF (25 mL). Following completion of the

reaction and work-up, purification by FCC (DCM:MeOH, 95:5) afforded the pure compound as an off-white solid (1.43 g, 68%).

RF (95:5 DCM:MeOH): 0.51

IR ν_{max} (cm^{-1}): 3480, 3286, 3065, 2920, 1776, 1720, 1418, 1115

HRMS (APCI)m/z: $[M + H]^+$ Calcd for $C_{17}H_{15}N_2O_3$ 295.1083; Found 295.1092

^1H NMR (400 MHz, $CDCl_3$): δ 7.89 – 7.81 (m, 2H), 7.75 – 7.69 (m, 2H), 7.35 – 7.27 (m, 2H), 7.26 – 7.21 (m, 3H), 6.24 (s, 1H), 4.43 (d, *J* = 5.7 Hz, 2H), 4.35 (s, 2H).

^{13}C NMR (101 MHz, $CDCl_3$): δ 167.9, 166.1, 137.7, 134.4, 132.1, 128.9, 127.9, 127.8, 123.8, 43.9, 40.9.

N-cyclopropyl-2-(1,3-dioxoisoindolin-2-yl)acetamide

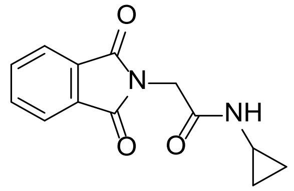

The title compound was prepared according the General Procedure **D** from phthalimidoglycine (200 mg, 0.98 mmol), HATU (440 mg, 1.20 mmol), DIPEA (0.34 mL, 1.95 mmol), and cyclopropylamine (75 µL, 1.10 mmol) in DMF (5 mL). Following completion of the reaction and work-up, purification by FCC (DCM:MeOH, 95:5) afforded the pure compound as an off-white solid (170 mg, 71%).

RF (95:5 DCM:MeOH): 0.30

IR ν_{max} (cm^{-1}): 3282, 2920, 1772, 1715, 1318, 1115, 949

HRMS (APCI)m/z: $[M + H]^+$ Calcd for $C_{13}H_{13}N_2O_3$ 245.0926; Found 245.0927

^1H NMR (400 MHz, DMSO-d_6): δ 8.27 (d, *J* = 3.7 Hz, 1H), 7.93 – 7.83 (m, 4H), 4.13 (s, 2H), 2.66 – 2.58 (m, 1H), 0.61 (td, *J* = 7.0, 4.8 Hz, 2H), 0.44 – 0.37 (m, 2H).

^{13}C NMR (101 MHz, DMSO-d_6): δ 167.5, 167.0, 134.6, 131.8, 123.2, 38.2, 22.3, 5.5.

2-(1,3-dioxoisoindolin-2-yl)-N-isobutyl-acetamide

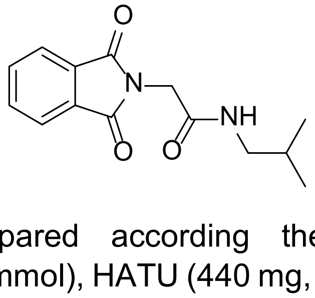

The title compound was prepared according the General Procedure **D** from phthalimidoglycine (200 mg, 0.98 mmol), HATU (440 mg, 1.20 mmol), DIPEA (0.34 mL, 1.95 mmol), and isobutylamine (91 µL, 0.91 mmol) in DMF (5 mL). Following completion of the reaction and work-up, purification by FCC (DCM:MeOH, 95:5) afforded the pure compound a white solid (170 mg, 67%).

RF (95:5 DCM:MeOH): 0.50

IR ν_{max} (cm^{-1}): 3297, 2958, 1771, 1718, 1416, 1254, 951

HRMS (APCI)m/z: $[M + H]^+$ Calcd for $C_{14}H_{17}N_2O_3$ 261.1239; Found 261.1230

^1H NMR (400 MHz, $CDCl_3$): δ 7.90 – 7.86 (m, 2H), 7.77 – 7.72 (m, 2H), 5.83 (s, 1H), 4.33 (s, 2H), 3.11 (dd, *J* = 6.6, 6.2 Hz, 2H), 1.78 (hept, *J* = 6.7 Hz, 1H), 0.90 (d, *J* = 6.7 Hz, 6H).

^{13}C NMR (101 MHz, $CDCl_3$): δ 168.0, 166.1, 134.4, 132.2, 123.8, 47.3, 41.1, 28.6, 20.2.

N-[(3-chlorophenyl)methyl]-2-(1,3-dioxoisoindolin-2-yl)acetamide

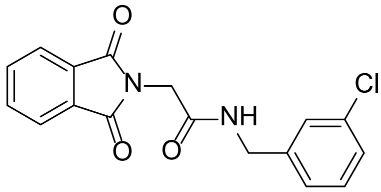

The title compound was prepared according the General Procedure **D** from phthalimidoglycine (200 mg, 0.98 mmol), HATU (440 mg, 1.20 mmol), DIPEA (0.34 mL, 1.95 mmol), and 3-chlorobenzylamine (0.16 mL, 1.10 mmol) in DMF (5 mL). Following completion of the reaction and work-up, purification by FCC (DCM:MeOH, 98:2) afforded the pure compound as a white solid (190 mg, 59%).

RF (95:5 DCM:MeOH): 0.57

IR ν_{max} (cm^{-1}): 3283, 3062, 1774, 1724, 1653, 1551, 1418, 952

HRMS (APCI)m/z: $[M + H]^+$ Calcd for $C_{17}H_{14}ClN_2O_3$ 329.0693; Found 329.0682

^1H NMR (400 MHz, $CDCl_3$): δ 7.91 – 7.87 (m, 2H), 7.81 – 7.72 (m, 2H), 7.28 – 7.24 (m, 3H), 7.19 – 7.14 (m, 1H), 6.07 (s, 1H), 4.45 (d, *J* = 5.9 Hz, 2H), 4.39 (s, 2H).

^{13}C NMR (101 MHz, $CDCl_3$): δ 167.9, 166.2, 139.7, 134.7, 134.5, 132.1, 130.2, 128.0, 128.0, 126.0, 123.9, 43.4, 41.1.

2-(1,3-dioxoisoindolin-2-yl)-N-(4-fluorophenyl)acetamide

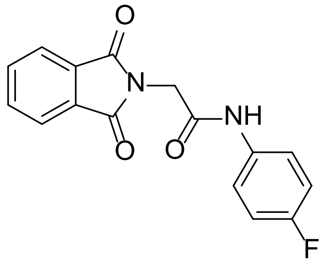

The title compound was prepared according the General Procedure **D** from phthalimidoglycine (200 mg, 0.98 mmol), HATU (440 mg, 1.20 mmol), DIPEA (0.34 mL, 1.95 mmol), and 4-fluoroaniline (0.10 mL, 1.10 mmol) in DMF (5 mL). Following completion of the

reaction and work-up, purification by FCC (DCM:EtOAc, 3:1) afforded the pure compound as a white solid (147 mg, 51%).

RF (95:5 DCM:MeOH): 0.53

IR ν_{max} (cm^{-1}): 3263, 3075, 1776, 1716, 1511, 1221, 952

HRMS (APCI)m/z: $[M + H]^+$ Calcd for $C_{16}H_{12}FN_2O_3$ 299.0832; Found 299.0827

^1H NMR (400 MHz, $CDCl_3$): δ 7.95 – 7.86 (m, 2H), 7.81 – 7.71 (m, 2H), 7.60 (s, 1H), 7.50 – 7.39 (m, 2H), 6.99 (t, *J* = 8.6 Hz, 2H), 4.50 (s, 2H).

^{13}C NMR (101 MHz, DMSO-d_6): δ 167.5, 164.8, 158.2 (d, *J* = 240.1 Hz), 134.9 (d, *J* = 2.5 Hz), 134.7, 131.6, 123.3, 121.0 (d, *J* = 7.9 Hz), 115.4 (d, *J* = 22.3 Hz), 40.7.

^{19}F NMR (376 MHz, CDCl3): δ -117.3

N-(2-chloro-4-methyl-phenyl)-2-(1,3-dioxoisoindolin-2-yl)acetamide

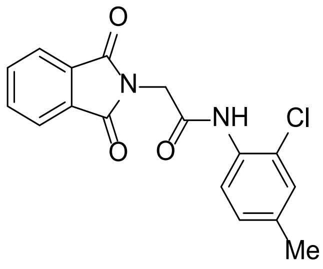

The title compound was prepared according the General Procedure **D** from phthalimidoglycine (200 mg, 0.98 mmol), HATU (440 mg, 1.20 mmol), DIPEA (0.34 mL, 1.95 mmol), and 2-chloro-4-methylaniline (0.13 mL, 1.10 mmol) in DMF (5 mL). Following completion of the reaction and work-up, purification by FCC (DCM:EtOAc, 3:1) afforded the pure compound as a white solid (148 mg, 46%).

RF (95:5 DCM:MeOH): 0.77

IR ν_{max} (cm^{-1}): 3248, 3047, 1769, 1722, 1668, 1538, 1412, 949

HRMS (APCI)m/z: $[M + H]^+$ Calcd for $C_{17}H_{14}ClN_2O_3$ 329.0693; Found 329.0685

^1H NMR (400 MHz, $CDCl_3$): δ 8.17 (d, *J* = 8.1 Hz, 1H), 7.94 – 7.90 (m, 2H), 7.86 (s, 1H), 7.79 – 7.75 (m, 2H), 7.18 (d, *J* = 1.0 Hz, 1H), 7.05 (dd, *J* = 8.2, 1.4 Hz, 1H), 4.56 (s, 2H), 2.29 (s, 3H).

^{13}C NMR (101 MHz, $CDCl_3$): δ 167.8, 164.1, 135.4, 134.5, 132.1, 131.5, 129.5, 128.52, 123.9, 122.7, 121.7, 41.8, 20.8.

N-benzyl-2-(1-hydroxy-3-oxo-1-phenyl-isoindolin-2-yl)acetamide (5a)

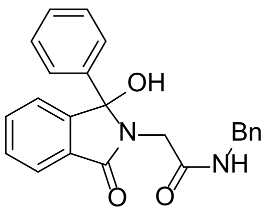

The title compound was prepared according to general procedure **A** from N-benzyl-2-(1,3-dioxoisoindolin-2-yl)acetamide (500 mg, 1.70 mmol), phenylmagnesium bromide (3.0 M in Et_2O, 1.70 mL, 5.10 mmol) in THF (9 mL). Following completion of the reaction (1 h), purification by FCC (1:1 EtOAc:Hex) afforded the pure product as a yellow solid (414 mg, 65%).

RF (1:1 EtOAc:Hex): 0.30

IR ν_{max} (cm^{-1}): 3289, 3062, 2922, 1638, 1541, 1370, 1055, 934

HRMS (APCI)m/z: $[M – H_2O]^+$ Calcd for $C_{23}H_{19}N_2O_2$ 355.1477; Found 355.1487

^1H NMR (400 MHz, $CDCl_3$): δ 7.68 – 7.61 (m, 1H), 7.51 (td, *J* = 7.5, 1.2 Hz, 1H), 7.46 – 7.40 (m, 1H), 7.40 – 7.30 (m, 6H), 7.28 – 7.18 (m, 5H), 6.69 (s, 1H), 4.55 (d, *J* = 16.5 Hz, 1H), 4.38 (ddd, *J* = 41.3, 14.7, 5.6 Hz, 2H), 3.57 (d, *J* = 16.4 Hz, 1H), 0.94 – 0.85 (m, 1H).

^{13}C NMR (101 MHz, $CDCl_3$): δ 170.0, 169.0, 150.1, 139.0, 137.3, 133.4, 129.3, 129.1, 128.8, 128.8, 128.0, 127.7, 126.4, 123.5, 123.0, 91.2, 77.4, 44.2, 43.5.

N-benzyl-2-[1-hydroxy-1-(4-methoxyphenyl)-3-oxo-isoindolin-2-yl]acetamide (5b)

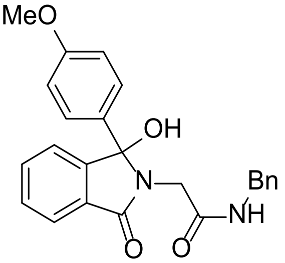

The title compound was prepared according to general procedure **A** from N-benzyl-2-(1,3-dioxoisoindolin-2-yl)acetamide (200 mg, 0.68 mmol), 4-methoxyphenylmagnesium bromide (0.5 M in THF, 4.0 mL, 2.04 mmol), in DCM (3 mL). The Grignard reagent was freshly prepared from magnesium turnings (51 mg, 2.11 mmol), 4-bromoanisole (381 mg, 0.26 mL, 2.04 mmol) and 1,2-dibromoethane (6 µL, 0.0068 mmol) in THF (2 mL). Following completion of the reaction (2 h), purification by FCC (1:3 EtOAc:DCM, 1% NEt_3) afforded the pure product as a white solid (165 mg, 60%).

RF (1:1 EtOAc:Hex): 0.27

IR ν_{max} (cm^{-1}): 3286, 3062, 2932, 2837, 1638, 1608, 1249, 1170

HRMS (APCI)m/z: $[M – H_2O]^+$ Calcd for $C_{24}H_{21}N_2O_3$ 385.1552; Found 385.1555

^1H NMR (400 MHz, $CDCl_3$): δ 7.63 (d, *J* = 7.4 Hz, 1H), 7.49 (td, *J* = 7.5, 1.2 Hz, 1H), 7.41 (td, *J* = 7.5, 1.0 Hz, 1H), 7.31 – 7.26 (m, 4H), 7.25 – 7.18 (m, 4H), 7.10 (t, *J* = 5.5 Hz, 1H), 6.84 (d, *J* = 9.0 Hz, 2H), 6.51 (s, 1H), 4.49 (d, *J* = 16.4 Hz, 1H), 4.37 (ddd, *J* = 38.8, 14.7, 5.6 Hz, 2H), 3.79 (s, 3H), 3.56 (d, *J* = 16.4 Hz, 1H).

^{13}C NMR (101 MHz, $CDCl_3$): δ 170.0, 168.9, 159.9, 150.3, 137.3, 133.3, 130.8, 129.2, 129.1, 128.8, 128.0, 127.8, 127.7, 123.5, 123.0, 114.2, 91.1, 55.4, 44.2, 43.4.

N-benzyl-2-[1-hydroxy-3-oxo-1-[4-(trifluoromethyl)phenyl]isoindolin-2-yl]acetamide (5c)

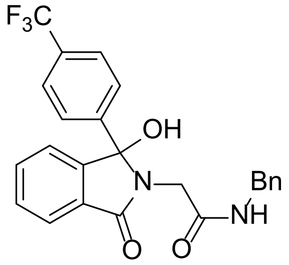

The title compound was prepared according to general procedure **A** from N-benzyl-2-(1,3-dioxoisoindolin-2-yl)acetamide (200 mg, 0.68 mmol), (4-(trifluoromethyl)phenyl)magnesium bromide (0.5 M in THF, 4.0 mL, 2.04 mmol), in DCM (3 mL). The Grignard reagent was freshly prepared from magnesium turnings (51 mg, 2.11 mmol), 4-bromobenzotrifluoride (460 mg, 2.04 mmol) and 1,2-dibromoethane (6 µL, 0.0068 mmol) in THF (2 mL). Following completion of the reaction (2 h), purification by FCC (1:1 EtOAc:Hex) afforded the pure product as a white solid (193 mg, 64%).

RF (1:1 EtOAc:Hex): 0.36

IR ν_{max} (cm^{-1}): 3297, 3055, 2889, 2822, 1690, 1644, 1323, 1109, 1068

HRMS (APCI)m/z: $[M – H_2O]^+$ Calcd for $C_{24}H_{18}F_3N_2O_2$ 423.1320; Found 423.1310

^1H NMR (400 MHz, $CDCl_3$): δ 7.83 – 7.75 (m, 1H), 7.63 – 7.55 (m, 4H), 7.55 – 7.45 (m, 2H), 7.36 – 7.26 (m, 5H), 6.41 (s, 1H), 6.37 (s, 1H), 4.52 (d, *J* = 16.3 Hz, 1H), 4.49 – 4.39 (m, 2H), 3.53 (d, *J* = 16.3 Hz, 1H).

^{13}C NMR (101 MHz, $CDCl_3$): δ 169.9, 168.9, 149.6, 143.4, 137.0, 133.6, 131.0 (q, *J* = 32.4 Hz), 129.6, 129.0, 128.9, 128.0, 127.9, 127.0, 125.9 (q, *J* = 3.5 Hz), 124.0 (q, *J* = 272.3 Hz), 123.7, 123.0, 90.6, 44.4, 43.5.

^{19}F NMR (376 MHz, $CDCl_3$): δ -62.7

N-benzyl-2-[1-(4-bromophenyl)-1-hydroxy-3-oxo-isoindolin-2-yl]acetamide (5d)

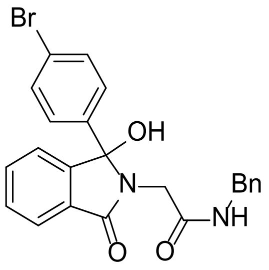

The title compound was prepared according to general procedure **A** from N-benzyl-2-(1,3-dioxoisoindolin-2-yl)acetamide (400 mg, 1.36 mmol), 4-bromophenylmagnesium bromide (0.5 M in THF, 8.0 mL, 4.08 mmol), in DCM (6 mL). The Grignard reagent was freshly prepared from magnesium turnings (102 mg, 4.21 mmol), 1,4-dibromobenzene (960 mg, 4.08 mmol) and 1,2-dibromoethane (12 µL, 0.0136 mmol) in THF (4 mL). Following completion of the reaction (30 mins), purification by FCC (1:3 EtOAc:DCM, 1% NEt_3) afforded the pure product as a white solid (270 mg, 44%).

RF (1:1 EtOAc:Hex): 0.33

IR ν_{max} (cm^{-1}): 3297, 3066, 2982, 1690, 1644, 1379, 1060, 937

HRMS (APCI)m/z: $[M - H_2O]^+$ Calcd for $C_{23}H_{18}BrN_2O_2$ 433.0552; Found 433.0564

^1H NMR (400 MHz, $CDCl_3$): δ 7.65 (d, *J* = 7.4 Hz, 1H), 7.53 (td, *J* = 7.5, 1.2 Hz, 1H), 7.50 – 7.42 (m, 3H), 7.32 – 7.21 (m, 8H), 7.15 (t, *J* = 5.6 Hz, 1H), 6.78 (s, 1H), 4.55 (d, *J* = 16.4 Hz, 1H), 4.39 (ddd, *J* = 41.0, 14.7, 5.6 Hz, 1H), 3.55 (d, *J* = 16.4 Hz, 1H).

^{13}C NMR (101 MHz, $CDCl_3$): δ 169.9, 168.9, 149.7, 138.3, 137.1, 133.5, 132.0, 129.5, 128.9, 128.9, 128.3, 128.0, 127.8, 126.4, 123.6, 123.0, 90.8, 44.3, 43.4.

N-benzyl-2-[1-hydroxy-1-(6-methoxy-2-pyridyl)-3-oxo-isoindolin-2-yl]acetamide (5e)

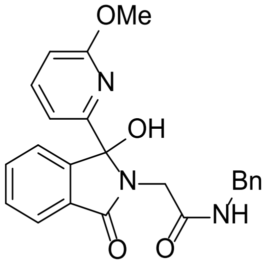

The title compound was prepared according to general procedure **B** from N-benzyl-2-(1,3-dioxoisoindolin-2-yl)acetamide (300 mg, 1.02 mmol), 2-bromo-6-methoxypyridine (770 mg, 0.50 mL, 4.09 mmol) and n-butyllithium (2.5 M in Hexane, 1.43 mL, 3.57 mmol) in THF (8 mL). Following completion of the reaction (1 h), purification by FCC (1:3 EtOAc:DCM, 1% NEt_3) afforded the pure product as a yellow solid (63 mg, 15%).

RF (1:1 EtOAc:Hex): 0.15

IR ν_{max} (cm^{-1}): 3286, 2928, 1707, 1638, 1467, 1267, 1027, 803

HRMS (APCI)m/z: $[M - H_2O]^+$ Calcd for $C_{23}H_{20}N_3O_3$ 386.1505; Found 386.1492

^{1}H NMR (400 MHz, $CDCl_3$): δ 7.94 – 7.86 (m, 1H), 7.62 – 7.49 (m, 3H), 7.36 – 7.29 (m, 3H), 7.28 – 7.18 (m, 3H), 6.93 (s, 1H), 6.78 (dd, *J* = 8.3, 0.6 Hz, 1H), 6.68 (s, 1H), 6.61 (dd, *J* = 7.4, 0.6 Hz, 1H), 4.42 (dd, *J* = 5.9, 1.6 Hz, 2H), 4.38 (d, *J* = 16.8 Hz, 1H), 4.01 (s, 3H), 3.61 (d, *J* = 16.8 Hz, 1H).

^{13}C NMR (101 MHz, $CDCl_3$): δ 168.9, 168.6, 163.5, 153.5, 147.8, 140.8, 138.1, 133.3, 130.4, 130.1, 128.8, 127.7, 127.5, 123. 9, 122.8, 113.3, 111.7, 90.0, 54.0, 43.7, 43.6.

2-[1-(1,3-benzodioxol-5-yl)-1-hydroxy-3-oxo-isoindolin-2-yl]-N-benzyl-acetamide (5f)

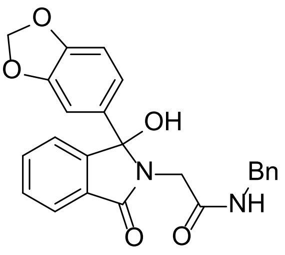

The title compound was prepared according to general procedure **B** from N-benzyl-2-(1,3-dioxoisoindolin-2-yl)acetamide (500 mg, 1.70 mmol), 1-bromo-3,4-(methylenedioxy)benzene (478 mg, 0.29 mL, 2.40 mmol) and n-butyllithium (2.5 M in Hexane, 0.82 mL, 2.04 mmol) in THF (6 mL). Following completion of the reaction (1 h), purification by FCC (1:3 EtOAc:DCM, 1% NEt_3) afforded the pure product as a yellow solid (108 mg, 38%).

RF (1:1 EtOAc:Hex): 0.21

IR ν_{max} (cm^{-1}): 3286, 3081, 2900, 1687, 1638, 1374, 1239, 1034

HRMS (APCI)m/z: $[M – H_2O]^+$ Calcd for $C_{24}H_{19}N_2O_4$ 399.1345; Found 399.1352

^{1}H NMR (400 MHz, $CDCl_3$): δ 7.69 (d, *J* = 7.4 Hz, 1H), 7.52 (td, *J* = 7.5, 1.2 Hz, 1H), 7.43 (td, *J* = 7.5, 1.0 Hz, 1H), 7.34 – 7.26 (m, 3H), 7.26 – 7.21 (m, 3H), 6.97 (dd, *J* = 8.1, 1.8 Hz, 1H), 6.81 – 6.73 (m, 3H), 6.38 (s, 1H), 5.95 (dd, *J* = 6.6, 1.4 Hz, 2H), 4.50 (d, *J* = 16.4 Hz, 1H), 4.47 – 4.32 (m, 2H), 3.60 (d, *J* = 16.3 Hz, 1H).

^{13}C NMR (101 MHz, $CDCl_3$): δ 170.0, 168.7, 150.1, 148.2, 148.0, 137.2, 133.4, 132.9, 129.3, 129.1, 128.9, 128.0, 127.9, 123.6, 122.9, 120.2, 108.4, 107.0, 101.5, 91.0, 44.3, 43.4.

N-[(3-chlorophenyl)methyl]-2-(1-hydroxy-3-oxo-1-phenyl-isoindolin-2-yl)acetamide (5g)

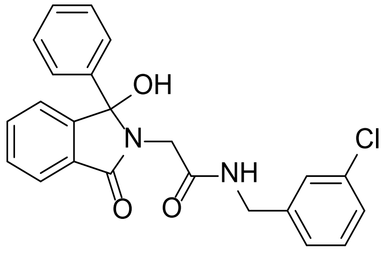

The title compound was prepared according to general procedure **A** from N-[(3-chlorophenyl)methyl]-2-(1,3-dioxoisoindolin-2-yl)acetamide (150 mg, 0.46 mmol), phenylmagnesium bromide (3.0 M in Et_2O, 0.46 mL, 1.37 mmol) in DCM (2 mL). Following

completion of the reaction (1 h), purification by FCC (1:5 EtOAc:DCM, 1% NEt_3) afforded the pure product as a white solid (104 mg, 56%).

RF (1:1 EtOAc:Hex): 0.27

IR ν_{max} (cm^{-1}): 3289, 2920, 1702, 1638, 1541, 1420, 1055, 768

HRMS (APCI)m/z: $[M - H_2O]^+$ Calcd for $C_{23}H_{18}ClN_2O_2$ 389.1057; Found 389.1045

^1H NMR (400 MHz, $CDCl_3$): δ 7.75 (d, *J* = 7.4 Hz, 1H), 7.52 (td, *J* = 7.5, 1.3 Hz, 1H), 7.46 (td, *J* = 7.4, 1.1 Hz, 1H), 7.43 – 7.38 (m, 2H), 7.38 – 7.29 (m, 4H), 7.25 – 7.20 (m, 3H), 7.16 – 7.09 (m, 1H), 6.68 (s, 1H), 4.50 (d, *J* = 16.4 Hz, 1H), 4.39 (ddd, *J* = 38.3, 15.0, 5.8 Hz, 2H), 3.60 (d, *J* = 16.3 Hz, 1H).

^{13}C NMR (101 MHz, $CDCl_3$): δ 172.9, 172.4, 145.4, 138.1, 136.2, 134.3, 132.9, 132.2, 130.9, 130.0, 129.7, 129.5, 127.6, 127.2, 126.9, 125.2, 125.0, 125.0, 86.6, 46.3, 44.8.

N-(4-fluorophenyl)-2-(1-hydroxy-3-oxo-1-phenyl-isoindolin-2-yl)acetamide (5h)

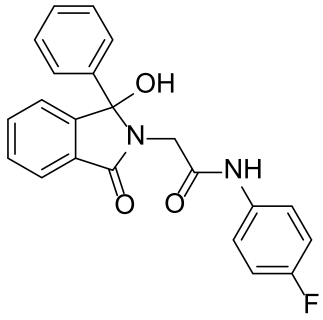

The title compound was prepared according to general procedure **A** from 2-(1,3-dioxoisoindolin-2-yl)-N-(4-fluorophenyl)acetamide (100 mg, 0.34 mmol), phenylmagnesium bromide (3.0 M in Et_2O, 0.34 mL, 1.01 mmol) in DCM (2 mL). Following completion of the reaction (30 mins), purification by FCC (1:5 EtOAc:DCM, 1% NEt_3) afforded the pure product as a colourless oil (80 mg, 63%).

RF (1:5 EtOAc:DCM): 0.40

IR ν_{max} (cm^{-1}): 3360, 3159, 3090, 2926, 1681, 1507, 1215, 1057

HRMS (APCI)m/z: $[M - H_2O]^+$ Calcd for $C_{22}H_{16}FN_2O_2$ 359.1196; Found 359.1209

^1H NMR (400 MHz, DMSO-d_6): δ δ 9.28 (s, 1H), 7.76 (d, *J* = 7.4 Hz, 1H), 7.54 (td, *J* = 7.5, 1.1 Hz, 1H), 7.47 (td, *J* = 7.5, 0.9 Hz, 1H), 7.41 – 7.27 (m, 8H), 6.83 (t, *J* = 8.7 Hz, 2H), 6.40 (s, 1H), 4.68 (d, *J* = 16.6 Hz, 1H), 3.74 (d, *J* = 16.6 Hz, 1H).

^{13}C NMR (101 MHz, DMSO-d_6): δ 169.6, 168.3, 159.6 (d, *J* = 244.0 Hz), 150.3, 138.5, 133.7, 133.5, 133.4, 129.6, 129.1, 129.0, 128.9, 126.4, 123.4, 123.3, 121.9 (d, *J* = 8.0 Hz), 115.5 (d, *J* = 22.5 Hz), 91.5, 44.3.

N-(2-chloro-4-methyl-phenyl)-2-(1-hydroxy-3-oxo-1-phenyl-isoindolin-2-yl)acetamide (5i)

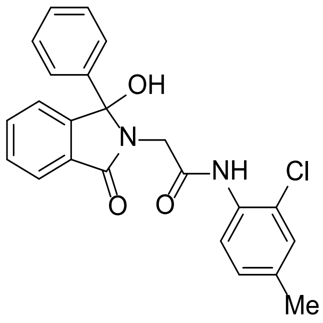

The title compound was prepared according to general procedure **A** from N-(2-chloro-4-methyl-phenyl)-2-(1,3-dioxoisoindolin-2-yl)acetamide (120 mg, 0.37 mmol), phenylmagnesium bromide (3.0 M in Et_2O, 0.37 mL, 1.10 mmol) in DCM (2 mL). Following completion of the reaction (30 mins), purification by FCC (1:9 EtOAc:DCM, 1% NEt_3) afforded the pure product as a white solid (104 mg, 56%).

RF (1:1 EtOAc:Hex): 0.39

IR ν_{max} (cm^{-1}): 3293, 3032, 2917, 1694, 1670, 1523, 1295, 936

HRMS (APCI)m/z: $[M – H_2O]^+$ Calcd for $C_{23}H_{18}ClN_2O_2$ 389.1057; Found 389.1066

^1H NMR (400 MHz, $CDCl_3$): δ 8.09 (d, *J* = 8.3 Hz, 1H), 7.97 (s, 1H), 7.88 (d, *J* = 6.7 Hz, 1H), 7.55 (td, *J* = 7.4, 1.4 Hz, 1H), 7.50 (td, *J* = 7.4, 1.3 Hz, 1H), 7.47 – 7.42 (m, 2H), 7.41 – 7.30 (m, 4H), 7.17 (s, 1H), 7.05 (d, *J* = 8.5 Hz, 1H), 4.82 (s, 1H), 4.62 (d, *J* = 16.5 Hz, 1H), 3.77 (d, *J* = 16.4 Hz, 1H), 2.29 (s, 3H).

^{13}C NMR (101 MHz, $CDCl_3$): δ 168.6, 168.1, 149.8, 145.3, 138.5, 133.5, 129.7, 129.6, 129.4, 129.2, 128.9, 128.5, 126.4, 123.9, 123.2, 123.1, 122.1, 91.1, 44.2, 31.1, 20.8.

N-cyclopropyl-2-(1-hydroxy-3-oxo-1-phenyl-isoindolin-2-yl)acetamide (5j)

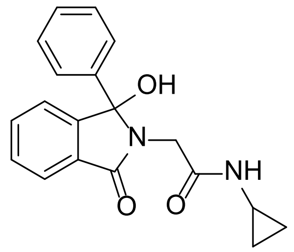

The title compound was prepared according to general procedure **A** from N-cyclopropyl-2-(1,3-dioxoisoindolin-2-yl)acetamide (150 mg, 0.61 mmol), phenylmagnesium bromide (3.0 M in Et_2O, 0.6 mL, 1.84 mmol) in DCM (3 mL). Following completion of the reaction (30 mins), purification by FCC (1:1 EtOAc:DCM, 1% NEt_3) afforded the pure product as a yellow oil (80 mg, 40%).

RF (1:1 EtOAc:Hex): 0.12

IR ν_{max} (cm^{-1}): 3267, 3058, 2924, 1705, 1648, 1370, 1054, 934

HRMS (APCI)m/z: $[M – H_2O]^+$ Calcd for $C_{19}H_{17}N_2O_2$ 305.1290; Found 305.1303

^{1}H NMR (400 MHz, $CDCl_3$): δ 7.82 (d, *J* = 7.1 Hz, 1H), 7.54 (td, *J* = 7.4, 1.3 Hz, 1H), 7.49 (td, *J* = 7.4, 1.2 Hz, 1H), 7.45 – 7.40 (m, 2H), 7.40 – 7.31 (m, 4H), 6.51 (s, 1H), 6.36 (s, 1H), 4.44 (d, *J* = 16.3 Hz, 1H), 3.53 (d, *J* = 16.2 Hz, 1H), 2.77 – 2.68 (m, 1H), 0.83 – 0.70 (m, 2H), 0.62 – 0.48 (m, 2H).

^{13}C NMR (101 MHz, DMSO-d_6): δ 171.5, 168.8, 150.2, 139.1, 133.4, 129.4, 129.3, 128.8, 128. 8, 126.4, 123.5, 123.1, 91.1, 43.4, 38.8, 23.1, 6.5, 6.5.

2-(1-hydroxy-3-oxo-1-phenyl-isoindolin-2-yl)-N-isobutyl-acetamide (5k)

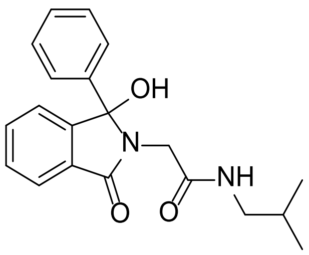

The title compound was prepared according to general procedure **A** from 2-(1,3-dioxoisoindolin-2-yl)-N-isobutyl-acetamide (150 mg, 0.58 mmol), phenylmagnesium bromide (3.0 M in Et_2O, 0.6 mL, 1.73 mmol) in DCM (3 mL). Following completion of the reaction (1 h), purification by FCC (1:5 EtOAc:DCM, 1% NEt_3) afforded the pure product as a pale yellow solid (115 mg, 59%).

RF (1:1 EtOAc:Hex): 0.30

IR ν_{max} (cm^{-1}): 3293, 2958, 2924, 1709, 1638, 1368, 934

HRMS (APCI)m/z: $[M – H_2O]^+$ Calcd for $C_{20}H_{21}N_2O_2$ 321.1603; Found 321.1591

^{1}H NMR (400 MHz, $CDCl_3$): δ 7.82 – 7.75 (m, 1H), 7.52 (td, *J* = 7.4, 1.3 Hz, 1H), 7.46 (td, *J* = 7.4, 1.1 Hz, 1H), 7.43 – 7.39 (m, 2H), 7.37 – 7.29 (m, 4H), 6.58 (s, 1H), 6.50 (s, 1H), 4.51 (d, *J* = 16.2 Hz, 1H), 3.55 (d, *J* = 16.2 Hz, 1H), 3.16 – 2.98 (m, 2H), 1.76 (hept, *J* = 6.7 Hz, 1H), 0.89 (d, *J* = 6.7 Hz, 6H).

^{13}C NMR (101 MHz, $CDCl_3$): δ 170.2, 168.9, 150.3, 139.2, 133.4, 129.3, 129.3, 128.8, 128.8, 126.5, 123.5, 123.1, 91.0, 47.6, 43.6, 28.5, 20.2.

General Procedure E for the Calcium catalysed dehydrative cyclisation of tethered amides

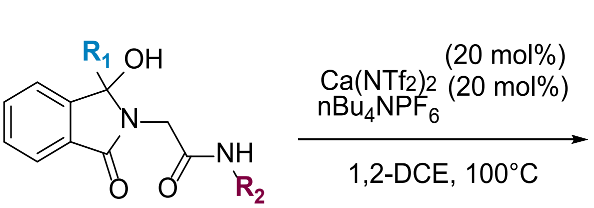

To a 4 mL vial capped with a capped with teflon cap was added $Ca(NTf_2)_2$ (20 mol%) and nBu_4NPF_6 (20 mol%) in 1,2-DCE (0.2 M). 3-hydroxyisoindolinone (1 equiv.) was added and the reaction was stirred at 100 °C until TLC analysis indicated full conversion to the product. The solution was then concentrated and purified by FCC (EtOAc:Hex) to afford the pure compound.

1-benzyl-9b-phenyl-3H-imidazo[2,1-a]isoindole-2,5-dione (6a)

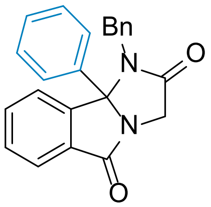

The title compound was prepared according to general procedure **E** from 2-[1-(1,3-benzodioxol-5-yl)-1-hydroxy-3-oxo-isoindolin-2-yl]-N-benzyl-acetamide (70 mg, 0.168 mmol), $Ca(NTf_2)_2$ (20 mg, 0.034 mmol) and nBu_4NPF_6 (10 mg, 0.034 mmol) in 1,2-DCE (0.7 mL). Following completion of the reaction (15 mins), purification by FCC (1:1 EtOAc:Hex) afforded the pure compound as a white solid (62 mg, 42%).

RF (1:9 EtOAc:DCM): 0.59

IR ν_{max} (cm^{-1}): 2911, 2853, 1709, 1388, 1079, 872

HRMS (APCI)m/z: $[M + H]^+$ Calcd for $C_{23}H_{19}N_2O_2$ 355.1447; Found 355.1460

^1H NMR (400 MHz, $CDCl_3$): δ 7.92 (d, J = 7.6 Hz, 1H), 7.51 (td, J = 7.5, 0.9 Hz, 1H), 7.45 – 7.37 (m, 3H), 7.35 (td, J = 7.6, 1.2 Hz, 1H), 7.16 (d, J = 7.7 Hz, 1H), 7.10 – 7.00 (m, 5H), 6.82 – 6.77 (m, 2H), 5.31 (d, J = 16.0 Hz, 1H), 4.64 (d, J = 16.4 Hz, 1H), 4.28 (d, J = 16.0 Hz, 1H), 3.77 (d, J = 16.4 Hz, 1H).

^{13}C NMR (101 MHz, $CDCl_3$): δ 173.0, 172.3, 145.5, 136.5, 136.0, 132.8, 132.3, 130.6, 129.8, 129.4, 128.4, 127.3, 126.9, 126.9, 125.2, 125.0, 86.7, 46.4, 45.4.

1-benzyl-9b-(4-methoxyphenyl)-3H-imidazo[2,1-a]isoindole-2,5-dione (6b)

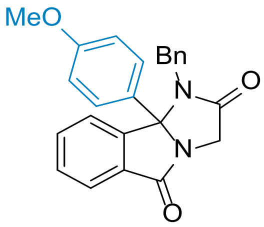

The title compound was prepared according to general procedure **E** from N-benzyl-2-[1-hydroxy-1-(4-methoxyphenyl)-3-oxo-isoindolin-2-yl]acetamide (80 mg, 0.20 mmol), $Ca(NTf_2)_2$ (24 mg, 0.040 mmol) and nBu_4NPF_6 (15 mg, 0.040 mmol) in 1,2-DCE (1.0 mL). Following completion of the reaction (1 h), purification by FCC (1:1 EtOAc:Hex) afforded the pure compound as a white solid (61 mg, 80%).

RF (1:1 EtOAc:Hex): 0.52

IR ν_{max} (cm^{-1}): 3021, 2922, 2838, 1707, 1418, 1397, 1258, 1028

HRMS (APCI)m/z: $[M + H]^+$ Calcd for $C_{24}H_{21}N_2O_3$ 385.1552; Found 285.1545

^{1}H NMR (400 MHz, $CDCl_3$): δ 7.91 (d, *J* = 7.5 Hz, 1H), 7.50 (td, *J* = 7.5, 0.9 Hz, 1H), 7.34 (td, *J* = 7.6, 1.1 Hz, 1H), 7.16 (d, *J* = 7.7 Hz, 1H), 7.05 – 7.02 (m, 3H), 6.99 (d, *J* = 9.0 Hz, 2H), 6.89 (d, *J* = 9.0 Hz, 2H), 6.83 – 6.75 (m, 2H), 5.27 (d, *J* = 16.0 Hz, 1H), 4.63 (d, *J* = 16.4 Hz, 1H), 4.26 (d, *J* = 16.0 Hz, 1H), 3.83 (s, 3H), 3.77 (d, *J* = 16.4 Hz, 1H).

^{13}C NMR (101 MHz, $CDCl_3$): δ 172.9, 172.3, 160.7, 145.6, 136.1, 132.8, 132.3, 130.6, 128.4, 128.4, 128.1, 127.3, 126.9, 125.1, 125.0, 114.7, 86.6, 55.6, 46.3, 45.3.

1-benzyl-9b-[4-(trifluoromethyl)phenyl]-3H-imidazo[2,1-a]isoindole-2,5-dione (6c)

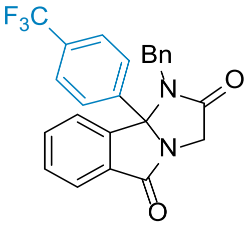

The title compound was prepared according to general procedure **E** from N-benzyl-2-[1-hydroxy-3-oxo-1-[4-(trifluoromethyl)phenyl]isoindolin-2-yl]acetamide (80 mg, 0.182 mmol), $Ca(NTf_2)_2$ (22 mg, 0.036 mmol) and nBu_4NPF_6 (14 mg, 0.036 mmol) in 1,2-DCE (0.9 mL). Following completion of the reaction (2 h), purification by FCC (1:1 EtOAc:Hex) afforded the pure compound as a white solid (56 mg, 73%).

RF (1:1 EtOAc:Hex): 0.70

IR ν_{max} (cm^{-1}): 3027, 2948, 2922, 1710, 1399, 1320, 760

HRMS (APCI)m/z: $[M + H]^+$ Calcd for $C_{24}H_{18}F_3N_2O_2$ 423.1230; Found 423.1236

^{1}H NMR (400 MHz, $CDCl_3$): δ 7.94 (d, *J* = 7.6 Hz, 1H), 7.64 (d, *J* = 8.2 Hz, 2H), 7.54 (td, *J* = 7.6, 0.9 Hz, 1H), 7.39 (td, *J* = 7.6, 1.1 Hz, 1H), 7.20 (d, *J* = 8.2 Hz, 2H), 7.15 (d, *J* = 7.7 Hz,

1H), 7.10 – 6.99 (m, 3H), 6.82 – 6.75 (m, 2H), 5.30 (d, *J* = 15.9 Hz, 1H), 4.66 (d, *J* = 16.5 Hz, 1H), 4.31 (d, *J* = 15.9 Hz, 1H), 3.74 (d, *J* = 16.5 Hz, 1H).

^{13}C NMR (101 MHz, $CDCl_3$): δ 172.8, 172.0, 144.9, 140.9, 135.6, 133.2, 132.1, 132.1 (d, *J* = 32.7 Hz), 131.0, 128.5, 127.5, 127.5, 127.0, 126.4 (q, *J* = 3.7 Hz), 125.3, 125.0, 86.1, 46.3, 45.4.

^{19}F NMR (376 MHz, $CDCl_3$): δ -62.9

1-benzyl-9b-(4-bromophenyl)-3H-imidazo[2,1-a]isoindole-2,5-dione (6d)

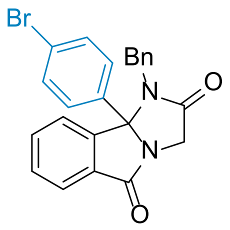

The title compound was prepared according to general procedure **E** from N-benzyl-2-[1-(4-bromophenyl)-1-hydroxy-3-oxo-isoindolin-2-yl]acetamide (70 mg, 0.155 mmol), $Ca(NTf_2)_2$ (19 mg, 0.031 mmol) and nBu_4NPF_6 (12 mg, 0.031 mmol) in 1,2-DCE (0.8 mL). Following completion of the reaction (2 h), purification by FCC (1:1 EtOAc:Hex) afforded the pure compound as a white solid (58 mg, 86%).

RF (1:1 EtOAc:Hex): 0.73

IR ν_{max} (cm^{-1}): 3032, 2924, 2249, 1707, 1392, 870

HRMS (APCI)m/z: $[M + H]^+$ Calcd for $C_{23}H_{18}BrN_2O_2$ 433.0552; Found 433.0561

^1H NMR (400 MHz, $CDCl_3$): δ 7.91 (d, *J* = 7.6 Hz, 1H), 7.50 (d, *J* = 8.3 Hz, 3H), 7.41 – 7.33 (m, 1H), 7.14 (d, *J* = 7.7 Hz, 1H), 7.07 – 6.99 (m, 3H), 6.93 (d, *J* = 8.5 Hz, 2H), 6.84 – 6.74 (m, 2H), 5.26 (d, *J* = 15.9 Hz, 1H), 4.63 (d, *J* = 16.5 Hz, 1H), 4.27 (d, *J* = 15.9 Hz, 1H), 3.73 (d, *J* = 16.5 Hz, 1H).

^{13}C NMR (101 MHz, $CDCl_3$): δ 172.8, 172.0, 145.0, 135.7, 133.0, 132.5, 132.0, 130.8, 128.6, 128.4, 127.4, 126.9, 125.1, 125.0, 124.2, 86.2, 77.4, 46.2, 45.2.

1-benzyl-9b-(6-methoxy-2-pyridyl)-3H-imidazo[2,1-a]isoindole-2,5-dione (6e)

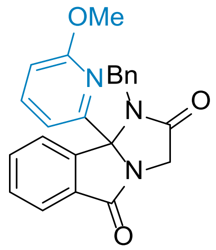

The title compound was prepared according to general procedure **E** from N-benzyl-2-[1-hydroxy-3-oxo-1-[4-(trifluoromethyl)phenyl]isoindolin-2-yl]acetamide (35 mg, 0.087 mmol), $Ca(NTf_2)_2$ (10 mg, 0.0017 mmol) and nBu_4NPF_6 (7 mg, 0.0017 mmol) in 1,2-DCE (0.4 mL).

Following completion of the reaction (2h), purification by FCC (1:1 EtOAc:Hex) afforded the pure compound as a pale yellow oil (25mg, 75%).

RF (1:1 EtOAc:Hex): 0.58

IR ν_{max} (cm^{-1}): 3030, 2932, 2857, 1707, 1575, 1467, 1388, 706

HRMS (APCI)m/z: $[M + H]^+$ Calcd for $C_{23}H_{20}N_3O_3$ 386.1505; Found 386.1510

^1H NMR (400 MHz, $CDCl_3$): δ 7.91 (d, J = 7.6 Hz, 1H), 7.55 – 7.47 (m, 3H), 7.44 – 7.39 (m, 1H), 7.10 – 7.00 (m, 3H), 6.88 (dd, J = 6.4, 3.1 Hz, 2H), 6.79 – 6.69 (m, 2H), 5.09 (d, J = 16.1 Hz, 1H), 4.64 (d, J = 15.9 Hz, 1H), 4.51 (d, J = 16.1 Hz, 1H), 4.02 (d, J = 15.9 Hz, 1H), 3.95 (s, 3H).

^{13}C NMR (101 MHz, $CDCl_3$): δ 172.9, 172.7, 164.2, 153.1, 143.8, 139.8, 136.6, 132.5, 132.4, 130.6, 128.3, 127.2, 127.2, 125. 4, 125.2, 113.9, 112.2, 85.7, 53.9, 47.6, 45.6.

9b-(1,3-benzodioxol-5-yl)-1-benzyl-3H-imidazo[2,1-a]isoindole-2,5-dione (6f)

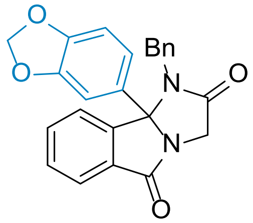

The title compound was prepared according to general procedure **E** from N-benzyl-2-[1-hydroxy-3-oxo-1-[4-(trifluoromethyl)phenyl]isoindolin-2-yl]acetamide (80 mg, 0.182 mmol), $Ca(NTf_2)_2$ (22 mg, 0.036 mmol) and nBu_4NPF_6 (14 mg, 0.036 mmol) in 1,2-DCE (0.9 mL). Following completion of the reaction (30 mins), purification by FCC (1:1 EtOAc:Hex) afforded the pure compound as a white solid (56 mg, 84%).

RF (1:1 EtOAc:Hex): 0.48

IR ν_{max} (cm^{-1}): 2994, 2906, 1716, 1702, 1489, 1313, 1251, 926

HRMS (APCI)m/z: $[M + H]^+$ Calcd for $C_{24}H_{19}N_2O_4$ 399.1345; Found 399.1338

^1H NMR (400 MHz, $CDCl_3$): δ 7.90 (d, J = 7.6 Hz, 1H), 7.50 (td, J = 7.6, 0.9 Hz, 1H), 7.35 (td, J = 7.6, 1.1 Hz, 1H), 7.17 (d, J = 7.7 Hz, 1H), 7.07 – 6.98 (m, 3H), 6.80 (d, J = 8.2 Hz, 2H), 6.78 – 6.75 (m, 1H), 6.62 (dd, J = 8.2, 2.0 Hz, 1H), 6.43 (d, J = 2.0 Hz, 1H), 5.99 (s, 2H), 5.26 (d, J = 16.0 Hz, 1H), 4.62 (d, J = 16.4 Hz, 1H), 4.27 (d, J = 16.0 Hz, 1H), 3.77 (d, J = 16.4 Hz, 1H).

^{13}C NMR (101 MHz, $CDCl_3$): δ 172.9, 172.2, 149.0, 148.8, 145.4, 136.0, 132.8, 132.2, 130.7, 130.2, 128.4, 127.3, 126.9, 125.1, 125.0, 120.7, 108.6, 107.5, 101.9, 86.6, 46.3, 45.3.

1-[(3-chlorophenyl)methyl]-9b-phenyl-3H-imidazo[2,1-a]isoindole-2,5-dione (6g)

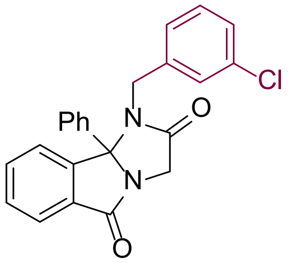

The title compound was prepared according to general procedure **E** from N-[(3-chlorophenyl)methyl]-2-(1-hydroxy-3-oxo-1-phenyl-isoindolin-2-yl)acetamide (44 mg, 0.108 mmol), $Ca(NTf_2)_2$ (13 mg, 0.022 mmol) and nBu_4NPF_6 (8 mg, 0.022 mmol) in 1,2-DCE (0.6 mL). Following completion of the reaction (40 mins), purification by FCC (1:1 EtOAc:Hex) afforded the pure compound as a white solid (34 mg, 81%).

RF (1:1 EtOAc:Hex): 0.58

IR v_{max} (cm^{-1}): 3058, 2924, 1707, 1597, 1467, 1388, 939

HRMS (APCI)m/z: $[M + H]^+$ Calcd for $C_{23}H_{18}ClN_2O_2$ 389.1057; Found 389.1062

^1H NMR (400 MHz, $CDCl_3$): δ 7.97 – 7.92 (m, 1H), 7.55 (td, *J* = 7.5, 0.9 Hz, 1H), 7.48 – 7.36 (m, 4H), 7.17 (d, *J* = 7.7 Hz, 1H), 7.10 – 7.05 (m, 2H), 7.05 – 7.00 (m, 1H), 6.96 (t, *J* = 7.8 Hz, 1H), 6.78 (s, 1H), 6.66 (d, *J* = 7.6 Hz, 1H), 5.27 (d, *J* = 16.0 Hz, 1H), 4.65 (d, *J* = 16.5 Hz, 1H), 4.24 (d, *J* = 16.1 Hz, 1H), 3.77 (d, *J* = 16.4 Hz, 1H).

^{13}C NMR (101 MHz, $CDCl_3$): δ 172.4, 145.4, 138.1, 136.2, 134.3, 132.9, 132.2, 130.9, 130.0, 130.8, 129.5, 127.8, 127.6, 127.2, 126.9, 125.2, 125.0, 125.0, 86.6, 46.3, 44.8.

1-(4-fluorophenyl)-9b-phenyl-3H-imidazo[2,1-a]isoindole-2,5-dione (6h)

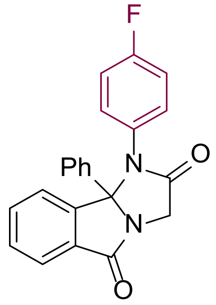

The title compound was prepared according to general procedure **E** from N-(4-fluorophenyl)-2-(1-hydroxy-3-oxo-1-phenyl-isoindolin-2-yl)acetamide (40 mg, 0.11 mmol), $Ca(NTf_2)_2$ (13 mg, 0.021 mmol) and nBu_4NPF_6 (8 mg, 0.021 mmol) in 1,2-DCE (0.5 mL). Following completion of the reaction (1 h), purification by FCC (1:5 EtOAc:Hex) afforded the pure compound as a white solid (20 mg, 53%).

RF (1:1 EtOAc:Hex): 0.70

IR v_{max} (cm^{-1}): 3058, 2919, 2853, 1711, 1510, 1221, 744

HRMS (APCI)m/z: $[M + H]^+$ Calcd for $C_{22}H_{16}FN_2O_2$ 359.1196; Found 359.1205

^1H NMR (400 MHz, $CDCl_3$): δ 8.05 – 7.99 (m, 1H), 7.66 (td, *J* = 7.5, 0.9 Hz, 1H), 7.55 (td, *J* = 7.6, 1.2 Hz, 1H), 7.51 – 7.40 (m, 3H), 7.37 – 7.29 (m, 2H), 7.11 (d, *J* = 7.7 Hz, 1H), 7.09 – 6.99 (m, 4H), 4.74 (d, *J* = 16.5 Hz, 1H), 4.02 (d, *J* = 16.5 Hz, 1H).

^{13}C NMR (101 MHz, $CDCl_3$): δ 172.5, 170.9, 161.9 (d, *J* = 248.8 Hz), 145.0, 137.7, 133.0, 132.6, 131.0, 130.9 (d, *J* = 3.3 Hz), 129.9, 129.4, 129.4 (d, *J* = 8.5 Hz), 126.6, 125.7, 125.4, 116.3 (d, *J* = 22.7 Hz), 87.3, 46.8.

1-(2-chloro-4-methyl-phenyl)-9b-phenyl-3H-imidazo[2,1-a]isoindole-2,5-dione (6i)

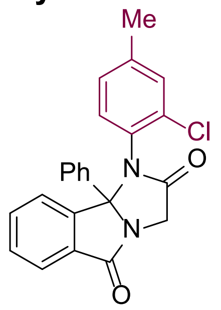

The title compound was prepared according to general procedure **E** from N-(2-chloro-4-methyl-phenyl)-2-(1-hydroxy-3-oxo-1-phenyl-isoindolin-2-yl)acetamide (34 mg, 0.084 mmol), $Ca(NTf_2)_2$ (10 mg, 0.017 mmol) and nBu_4NPF_6 (7 mg, 0.017 mmol) in 1,2-DCE (0.4 mL). Following completion of the reaction (12 h), purification by FCC (1:19 EtOAc:Hex) afforded the pure compound as a white solid (24 mg, 74%).

RF (1:1 EtOAc:Hex): 0.57

IR ν_{max} (cm^{-1}): 3055, 2967, 2926, 1715, 1497, 1374, 1224, 1055

HRMS (APCI)m/z: $[M + H]^+$ Calcd for $C_{23}H_{18}ClN_2O_2$ 389.1057; Found 389.1052

^1H NMR (400 MHz, $CDCl_3$): δ 8.03 – 7.99 (m, 1H), 7.64 (td, *J* = 7.6, 1.0 Hz, 1H), 7.51 – 7.45 (m, 3H), 7.45 – 7.41 (m, 3H), 7.18 – 7.12 (m, 2H), 7.08 – 7.03 (m, 1H), 6.75 – 6.70 (m, 1H), 4.74 (d, *J* = 16.6 Hz, 1H), 4.07 (d, *J* = 16.6 Hz, 1H), 2.33 (s, 3H).

^{13}C NMR (101 MHz, $CDCl_3$): δ 172.9, 170.8, 144.0, 140.9, 137.8, 134.3, 132.8, 131.0, 130.6, 129.7, 129.7, 129.2, 129.0, 129.0, 128.3, 126.9, 125.1, 124.5, 87.4, 46.9, 21.0.

1-cyclopropyl-9b-phenyl-3H-imidazo[2,1-a]isoindole-2,5-dione (6j)

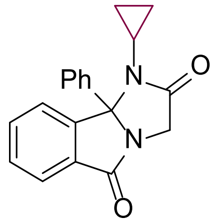

The title compound was prepared according to general procedure **E** from N-cyclopropyl-2-(1-hydroxy-3-oxo-1-phenyl-isoindolin-2-yl)acetamide (40 mg, 0.12 mmol), $Ca(NTf_2)_2$ (15 mg, 0.025 mmol) and nBu_4NPF_6 (10 mg, 0.025 mmol) in 1,2-DCE (0.6 mL). Following completion of the reaction (1 h), purification by FCC (1:5 EtOAc:Hex) afforded the pure compound as a white solid (31 mg, 82%).

RF (1:1 EtOAc:Hex): 0.45

IR v_{max} (cm^{-1}): 3006, 2926, 1707, 1450, 1325, 1133, 746

HRMS (APCI)m/z: $[M + H]^+$ Calcd for $C_{19}H_{17}N_2O_2$ 305.1290; Found 305.1302

^{1}H NMR (400 MHz, $CDCl_3$): δ 8.02 – 7.94 (m, 1H), 7.75 – 7.62 (m, 3H), 7.46 – 7.34 (m, 3H), 7.18 – 7.09 (m, 2H), 4.45 (d, *J* = 16.4 Hz, 1H), 3.69 (d, *J* = 16.4 Hz, 1H), 2.53 – 2.44 (m, 1H), 1.09 – 0.97 (m, 1H), 0.95 – 0.82 (m, 1H), 0.81 – 0.65 (m, 1H), 0.46 – 0.35 (m, 1H).

^{13}C NMR (101 MHz, $CDCl_3$): δ 172.8, 172.4, 145.4, 137.4, 132.9, 132.7, 130.8, 129.7, 129.3, 126.6, 126.3, 125.1, 87.0, 46.5, 23.6, 7.4, 5.0.

1-isobutyl-9b-phenyl-3H-imidazo[2,1-a]isoindole-2,5-dione (6k)

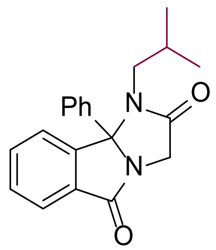

The title compound was prepared according to general procedure **E** from 2-(1-hydroxy-3-oxo-1-phenyl-isoindolin-2-yl)-N-isobutyl-acetamide (60 mg, 0.18 mmol), $Ca(NTf_2)_2$ (21 mg, 0.036 mmol) and nBu_4NPF_6 (14 mg, 0.036 mmol) in 1,2-DCE (0.9 mL). Following completion of the reaction (1.5 h), purification by FCC (1:5 EtOAc:Hex) afforded the pure compound as a white solid (46 mg, 81%).

RF (1:1 EtOAc:Hex): 0.58

IR v_{max} (cm^{-1}): 2932, 2904, 2868, 1711, 1450, 1346, 915

HRMS (APCI)m/z: $[M + H]^+$ Calcd for $C_{20}H_{21}N_2O_2$ 321.1603; Found 321.1597

^{1}H NMR (400 MHz, $CDCl_3$): δ 8.00 (ddd, *J* = 5.6, 3.1, 0.7 Hz, 1H), 7.72 – 7.65 (m, 2H), 7.45 – 7.33 (m, 4H), 7.01 (dd, *J* = 8.1, 1.6 Hz, 2H), 4.54 (d, *J* = 16.3 Hz, 1H), 3.75 (dd, *J* = 13.8, 8.3 Hz, 1H), 3.62 (d, *J* = 16.3 Hz, 1H), 2.94 (dd, *J* = 13.8, 6.8 Hz, 1H), 0.72 (d, *J* = 6.7 Hz, 3H), 0.52 (d, *J* = 6.7 Hz, 3H).

^{13}C NMR (101 MHz, $CDCl_3$): δ 172.7, 172.6, 146.4, 136.9, 133.1, 132.8, 131.0, 129.7, 129.3, 126.9, 125.3, 124.8, 86.8, 50.0, 46.2, 28.1, 20.3, 20.1.

Crystallography Data

A single crystal was selected and mounted, on a Mitegen loop using Paratone-N oil, on a SuperNova, Cu, AtlasS2 diffractometer. The crystal was kept at 100(2) K during data collection. Using Olex2 [1], the structure was solved with the SHELXT [2] structure solution program using Intrinsic Phasing and refined with the SHELXL [3] refinement package using Least Squares minimisation.

1. Dolomanov, O.V., Bourhis, L.J., Gildea, R.J, Howard, J.A.K. & Puschmann, H. (2009), J. Appl. Cryst. 42, 339-341.
2. Sheldrick, G.M. (2015). Acta Cryst. A71, 3-8.
3. Sheldrick, G.M. (2015). Acta Cryst. C71, 3-8.

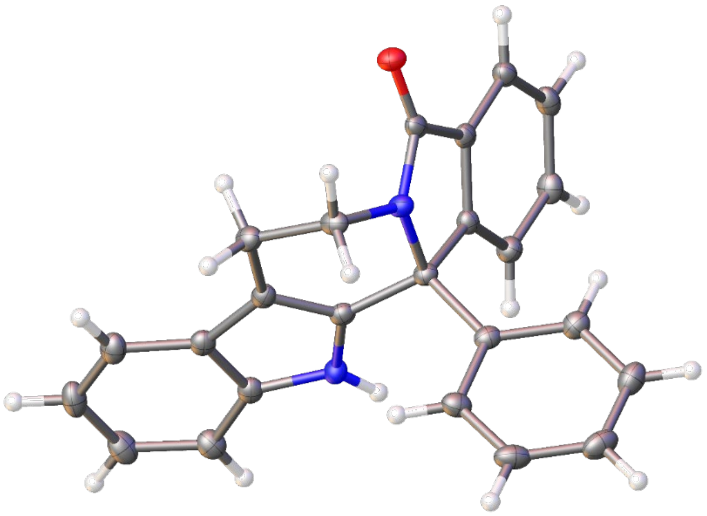

Crystal data and structure refinement for CCDC 2154226, this data is available free-of-charge from www.ccdc.cam.ac.uk.

Identification code	Compound **4a**
Empirical formula	$C_{24}H_{18}N_2O$
Formula weight g/mol	350.40
Temperature/K	100(2)
Crystal system	orthorhombic
Space group	$P2_12_12_1$
a/Å	10.84410(10)
b/Å	11.71540(10)
c/Å	13.6836(2)
α/°	90
β/°	90
γ/°	90
Volume/Å^3	1738.41(3)
Z	4
ρ_{calc} g/cm^3	1.339
μ/mm^{-1}	0.649
F(000)	736.0
Crystal size/mm^3	0.13 × 0.06 × 0.03
Radiation	Cu Kα (λ = 1.54184 Å)
2Θ range for data collection/°	9.94 to 152.482

Index ranges	-12 ≤ h ≤ 13, -14 ≤ k ≤ 11, -16 ≤ l ≤ 17
Reflections collected	19183
Independent reflections	3596 [R_{int} = 0.0270, R_{sigma} = 0.0155]
Data/restraints/parameters	3596/0/245
Goodness-of-fit on F^2	1.034
Final R indexes [I>=2σ (I)]	R_1 = 0.0296, wR_2 = 0.0773
Final R indexes [all data]	R_1 = 0.0301, wR_2 = 0.0779
Largest diff. peak/hole / e Å^{-3}	0.22/-0.16
Flack parameter	-0.12(7)

References

[1] A. G. Griesbeck, J. Hirt, W. Kramer, P. Dallakian, *Tetrahedron* **1998**, *54*, 3169-3180.

[2] P. Feng, Y. Fan, F. Xue, W. Liu, S. Li, Y. Shi, *Org. Lett.* **2011**, *13*, 5827-5829.

[3] A. S. Surur, C. Bock, K. Beirow, K. Wurm, L. Schulig, M. K. Kindermann, W. Siegmund, P. J. Bednarski, A. Link, *Org. Biomol. Chem.* **2019**, *17*, 4512-4522.

Copies of Spectra

Figure S. 1. N-(2-mercaptoethyl)-phthalimide (1)
^{1}H NMR (400 MHz, $CDCl_3$)

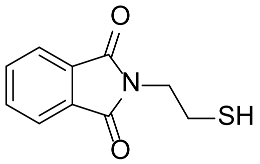

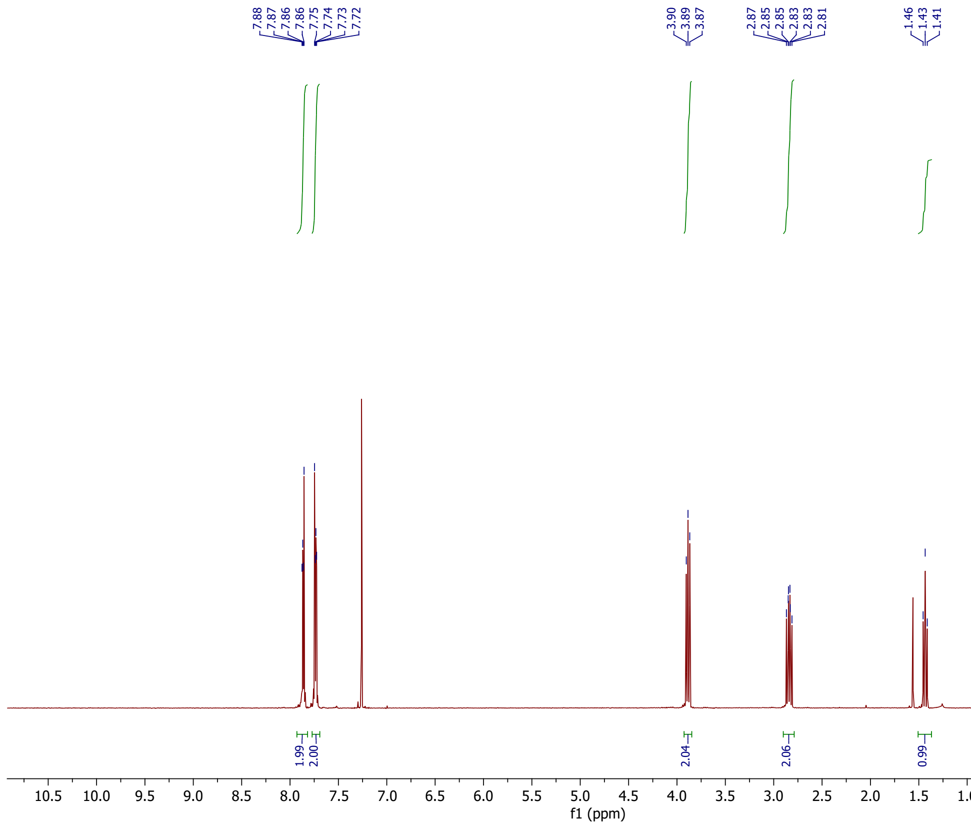

^{13}C NMR (101 MHz, $CDCl_3$)

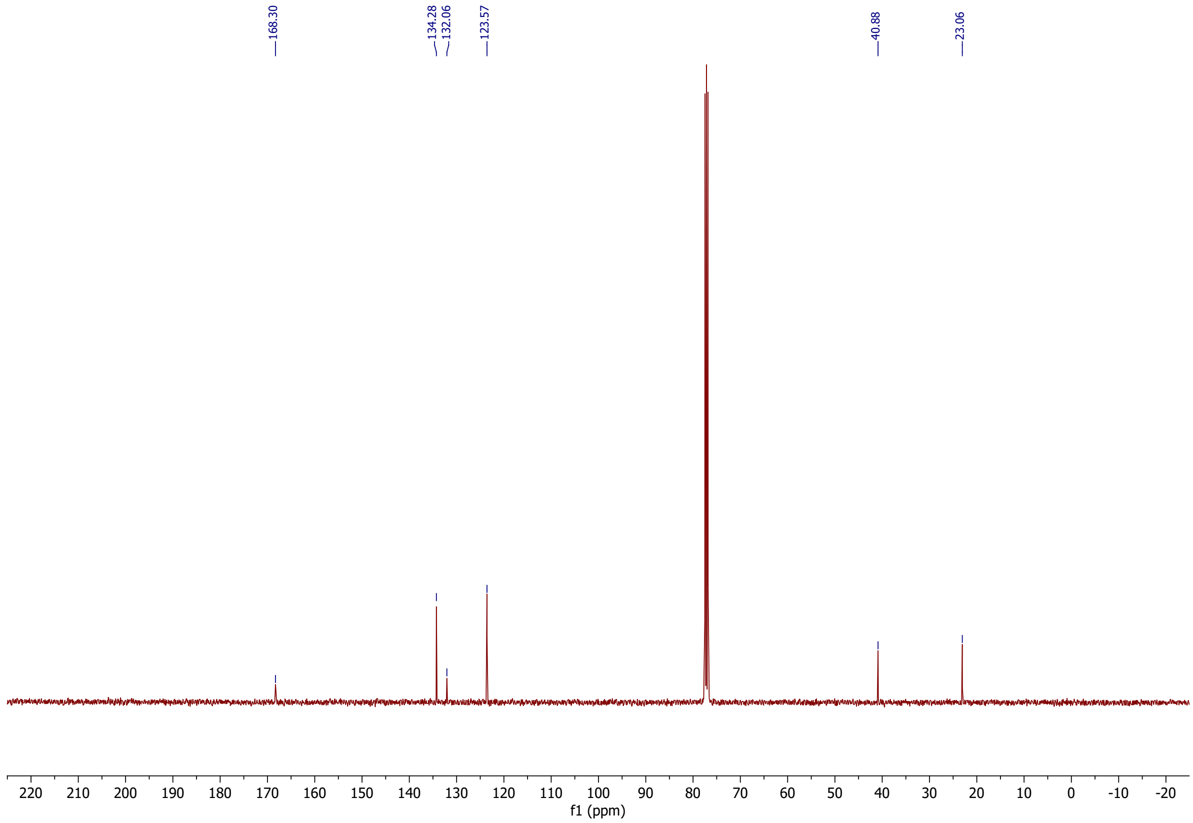

Figure S. 2. 3-hydroxy-3-phenyl-2-(2-sulfanylethyl)isoindolin-1-one (1a)

^{1}H NMR (400 MHz, DMSO-d_6)

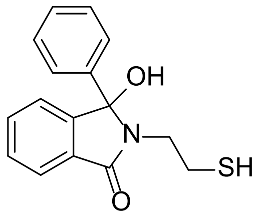

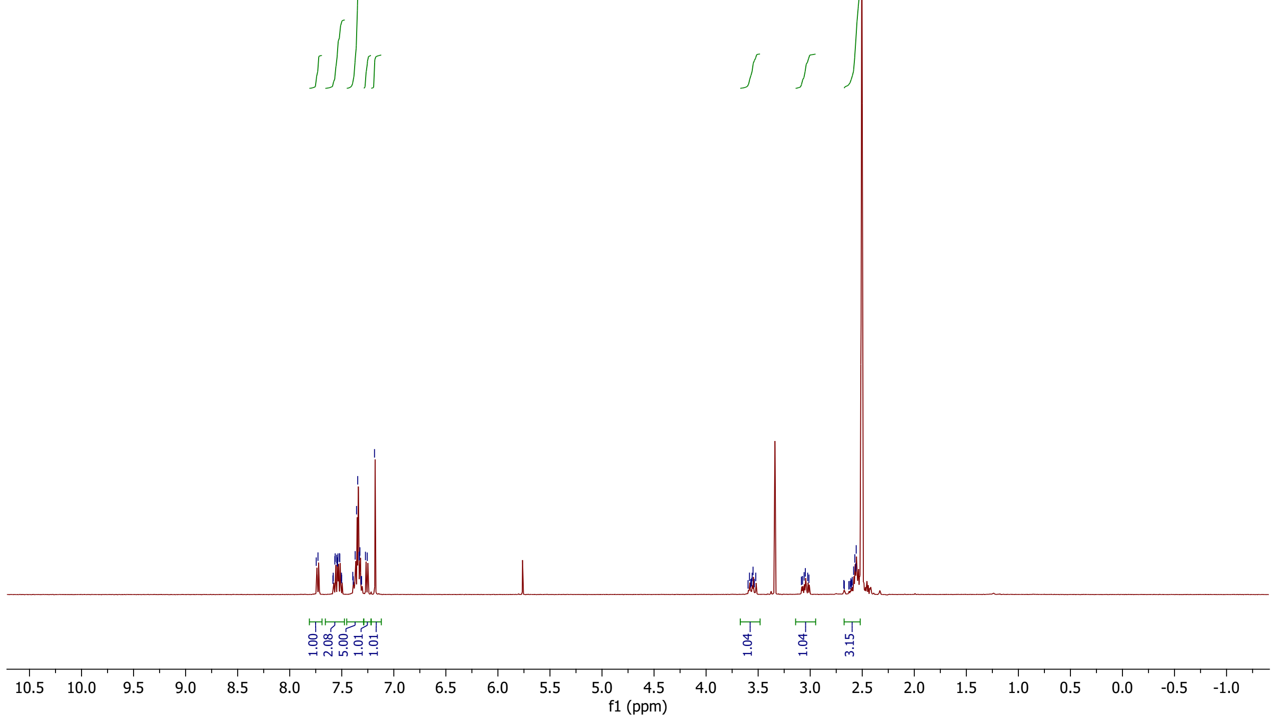

^{13}C NMR (101 MHz, DMSO-d_6)

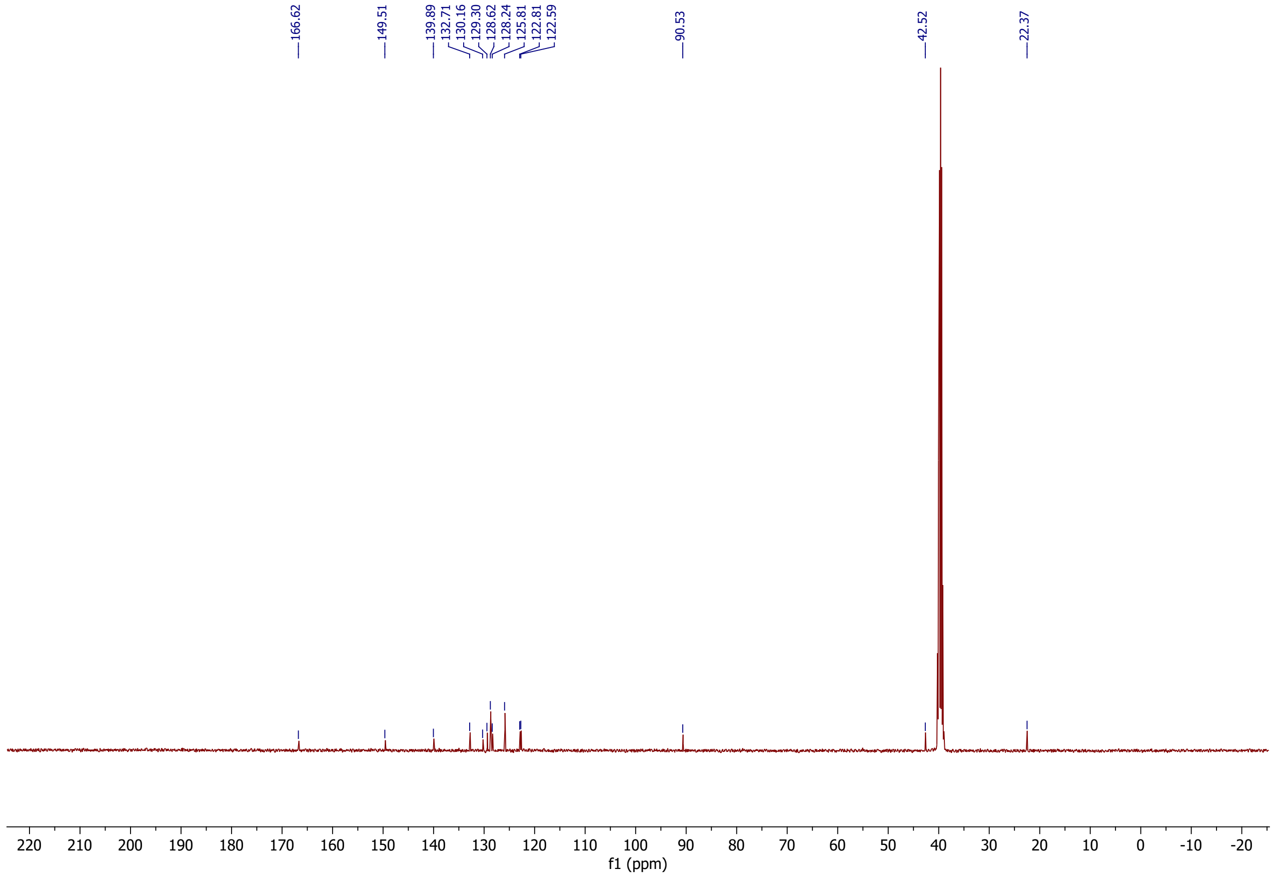

Figure S. 3. 3-(4-chlorophenyl)-3-hydroxy-2-(2-sulfanylethyl)isoindolin-1-one (1b)

^{1}H NMR (400 MHz, $CDCl_3$)

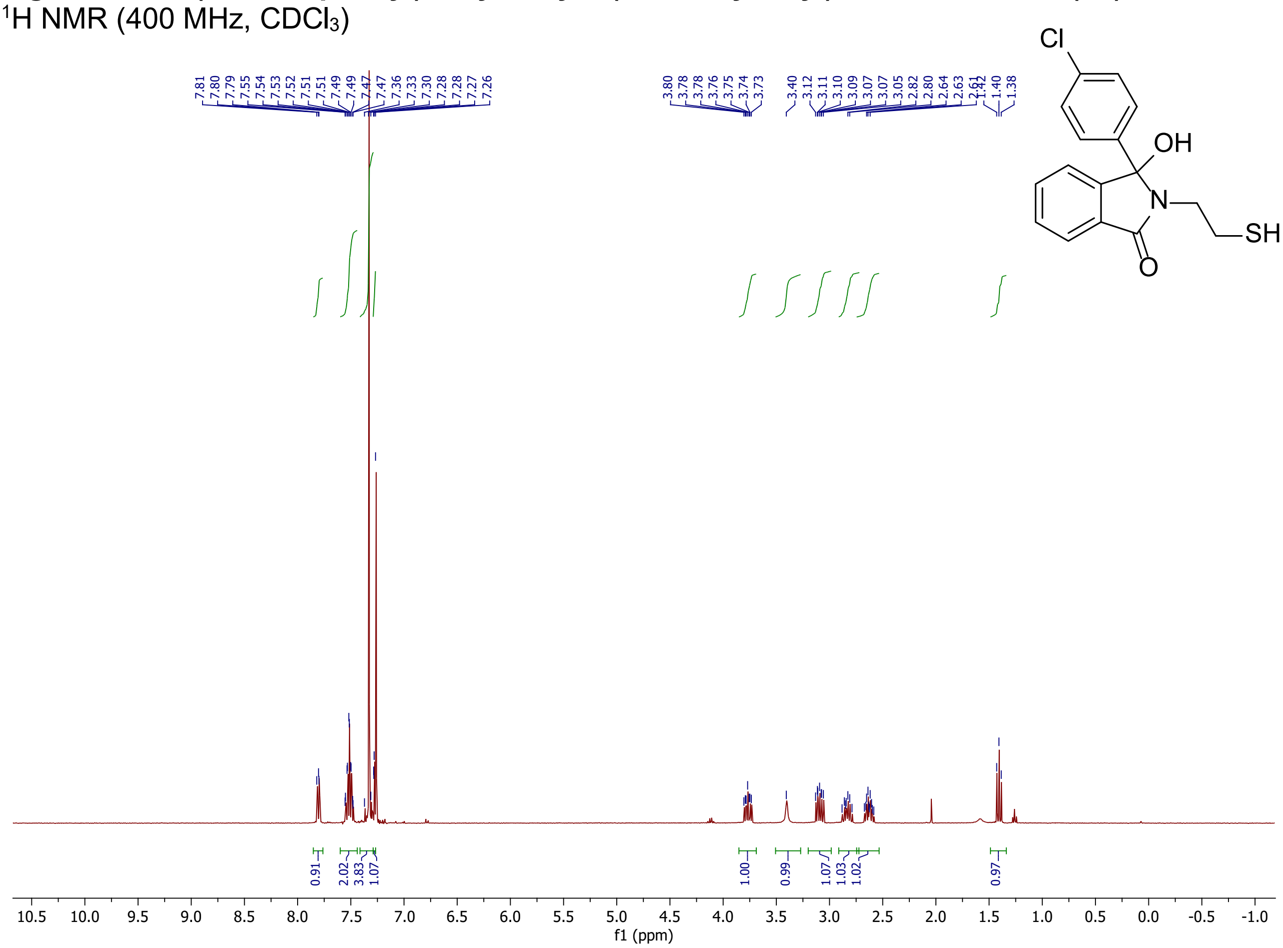

^{13}C NMR (101 MHz, $CDCl_3$)

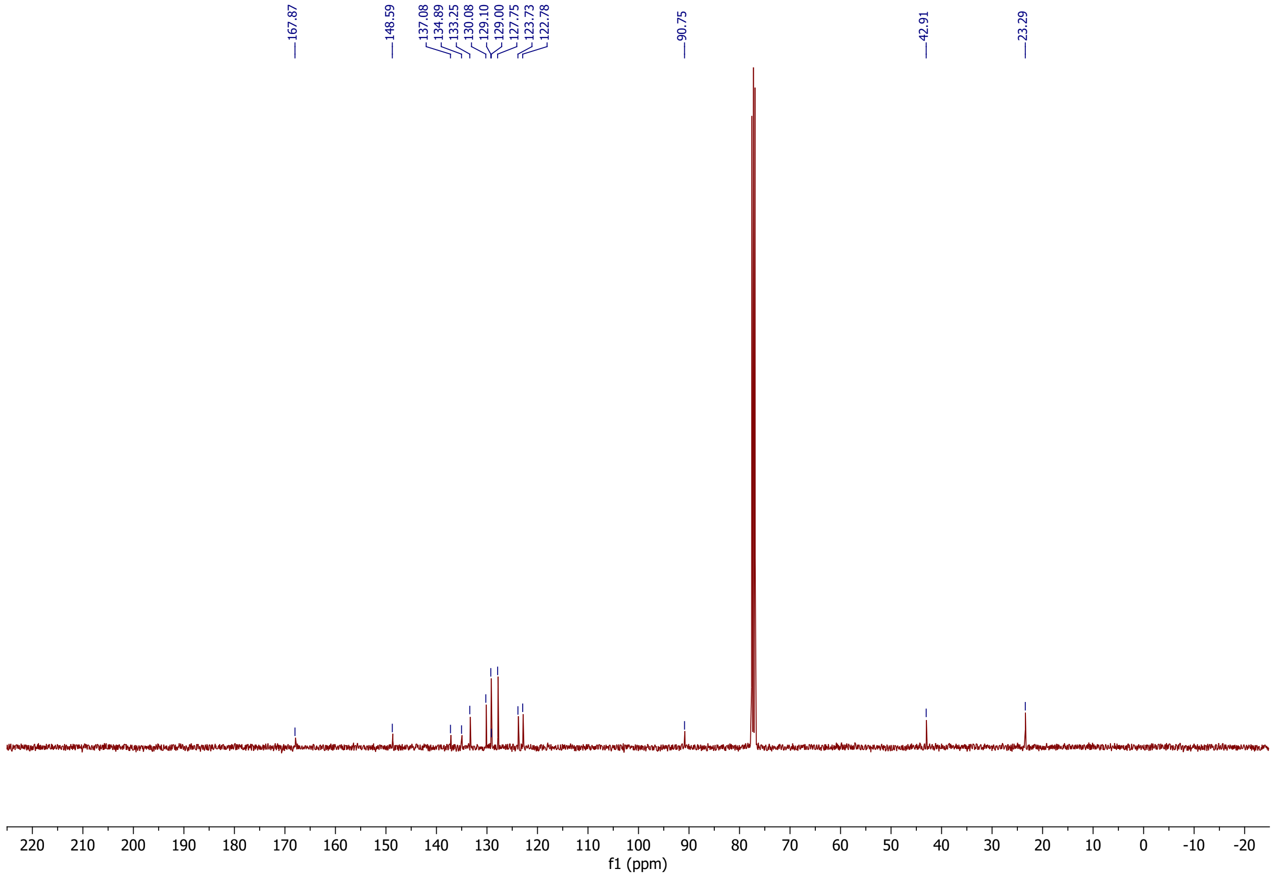

Figure S. 4. 3-hydroxy-2-(2-sulfanylethyl)-3-[4-(trifluoromethyl)phenyl]isoindolin-1-one (1c)

^{1}H NMR (400 MHz, $CDCl_3$)

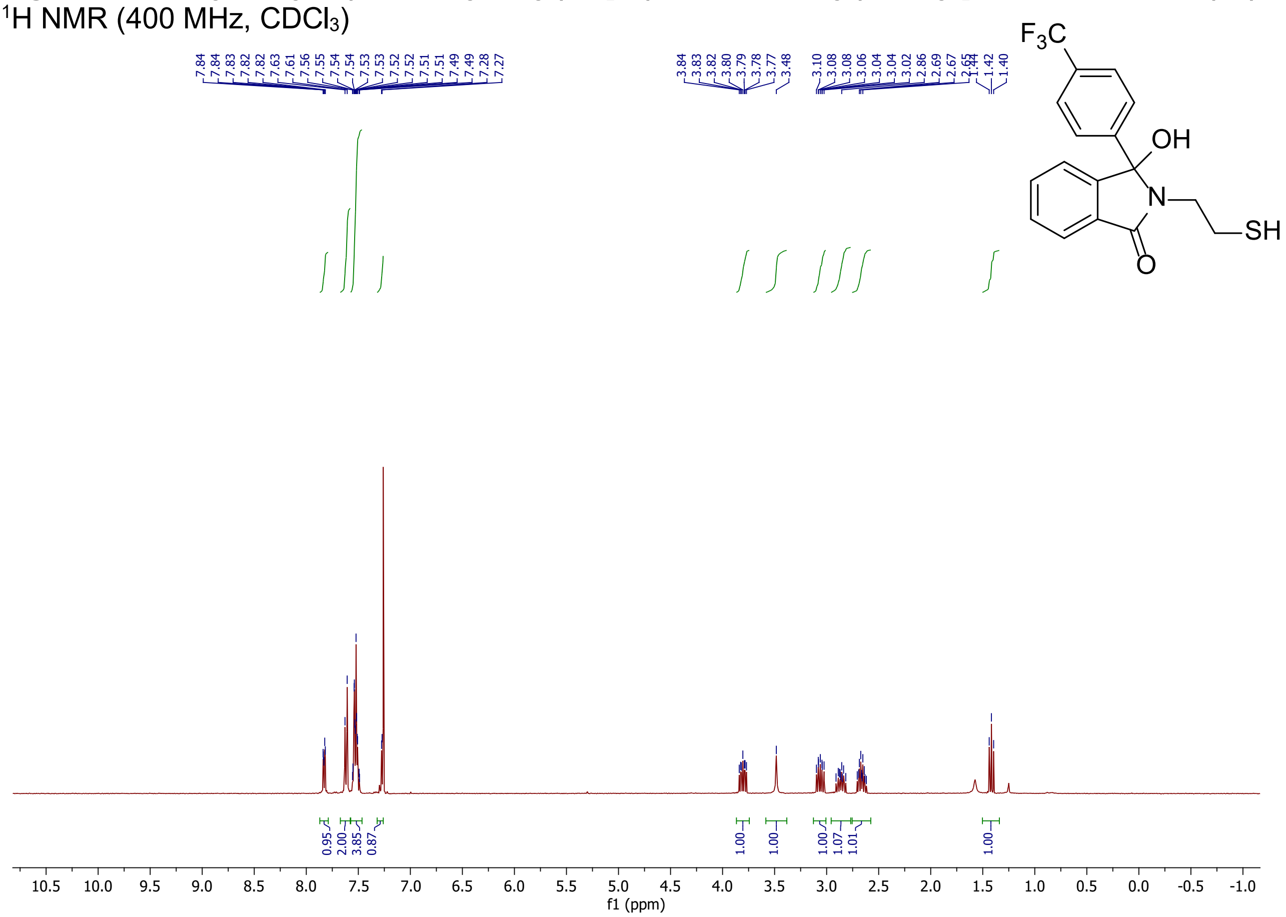

^{13}C NMR (101 MHz, $CDCl_3$)

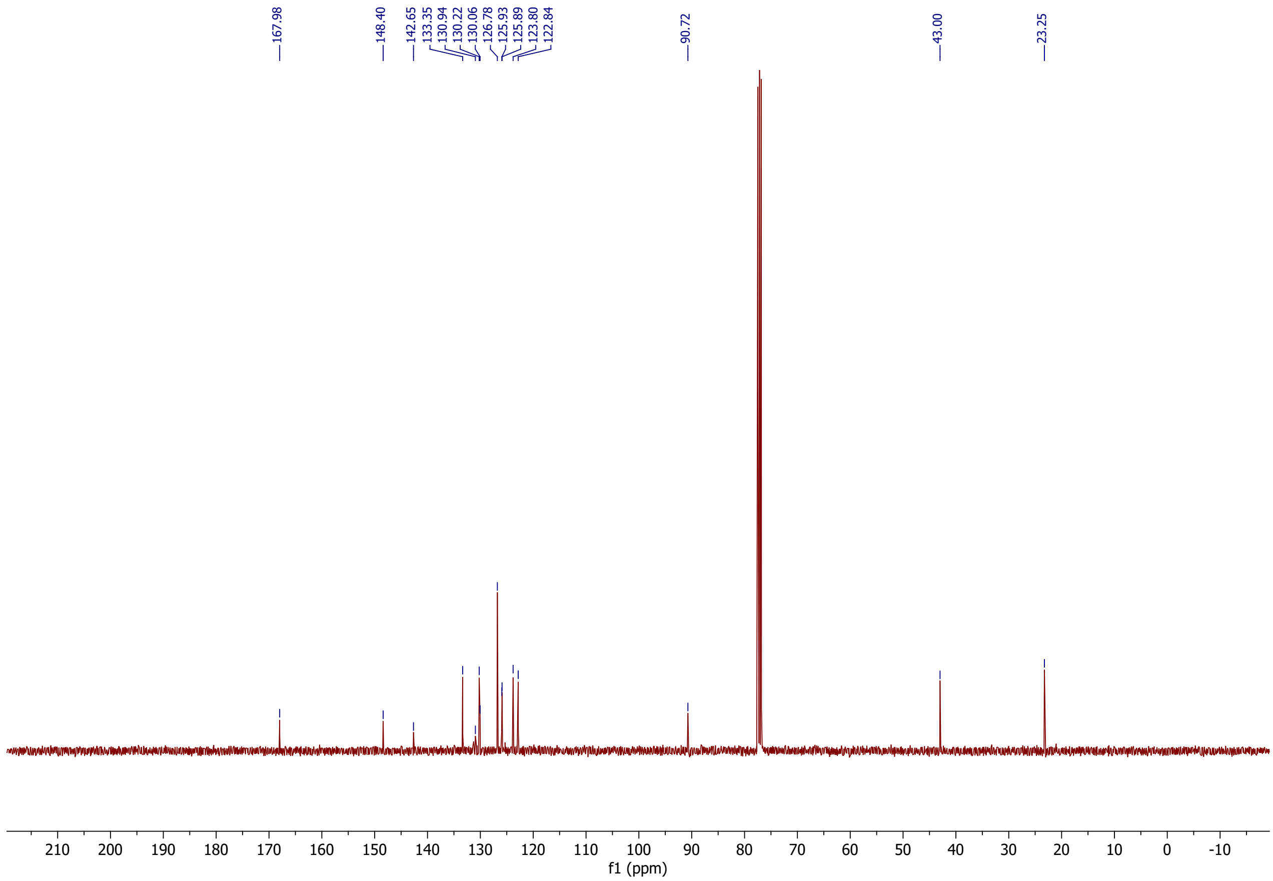

Figure S. 5. 3-hydroxy-3-(4-methoxyphenyl)-2-(2-sulfanylethyl)isoindolin-1-one (1d)

^{1}H NMR (400 MHz, $CDCl_3$)

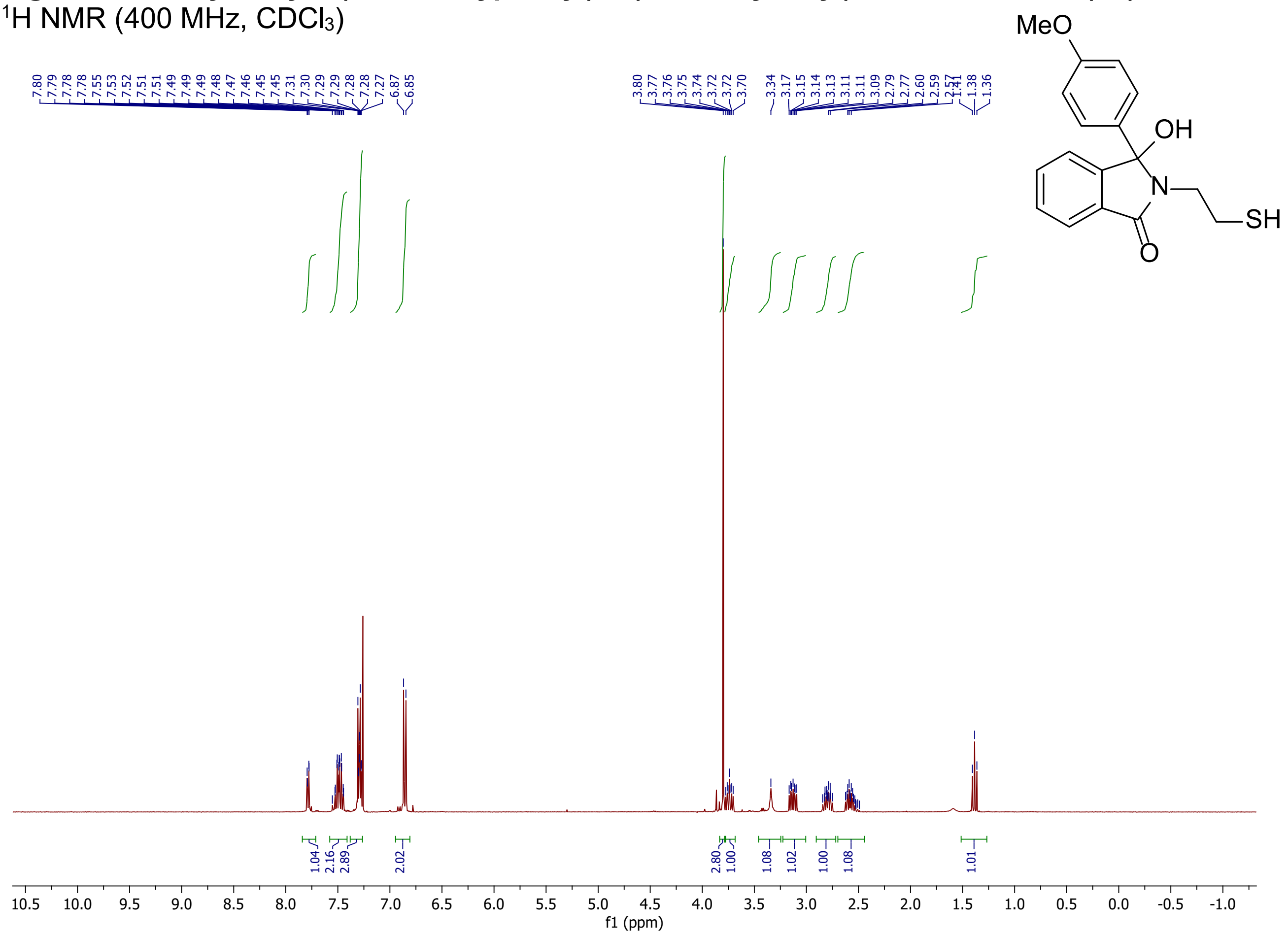

^{13}C NMR (101 MHz, $CDCl_3$)

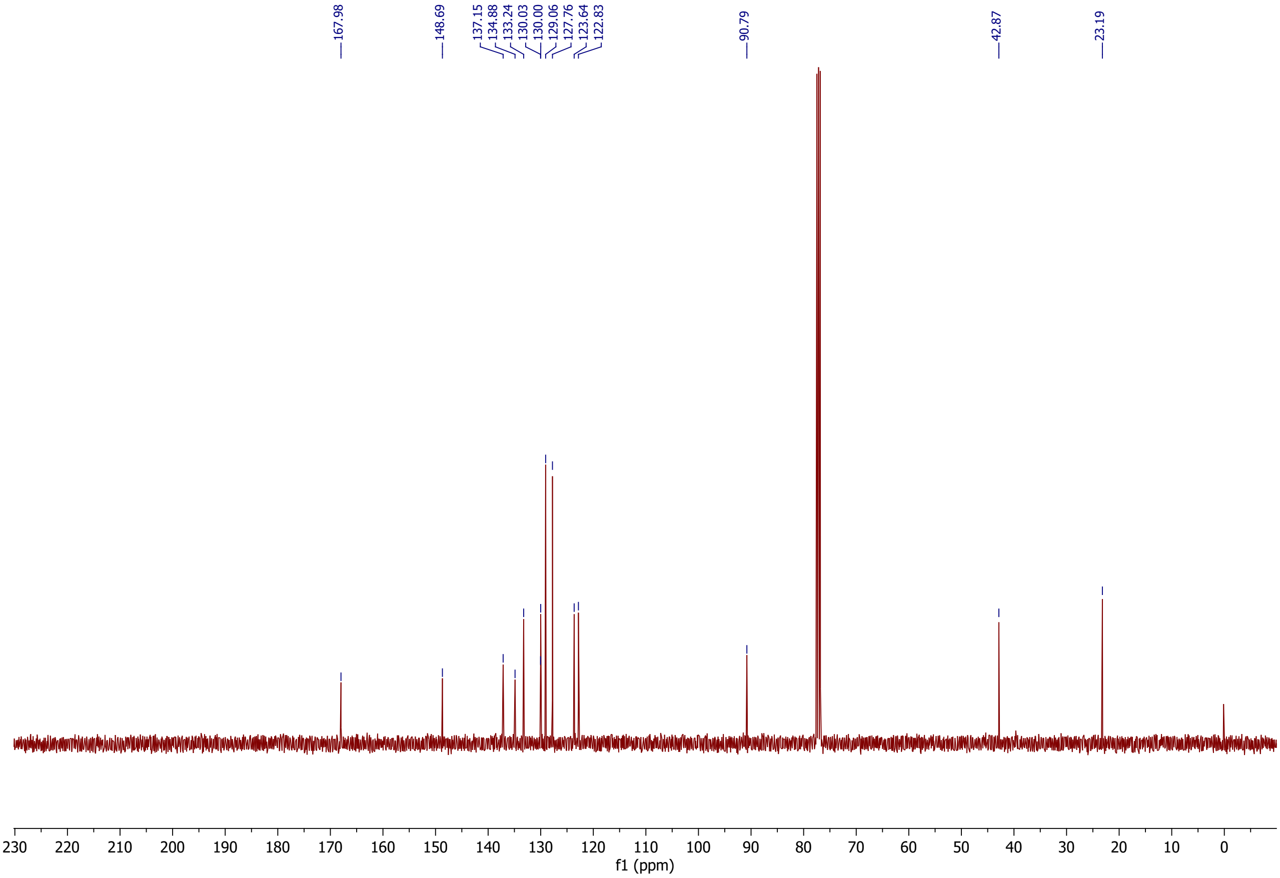

Figure S. 6. 3-hydroxy-3-(3-methoxyphenyl)-2-(2-sulfanylethyl)isoindolin-1-one (1e)

^{1}H NMR (400 MHz, $CDCl_3$)

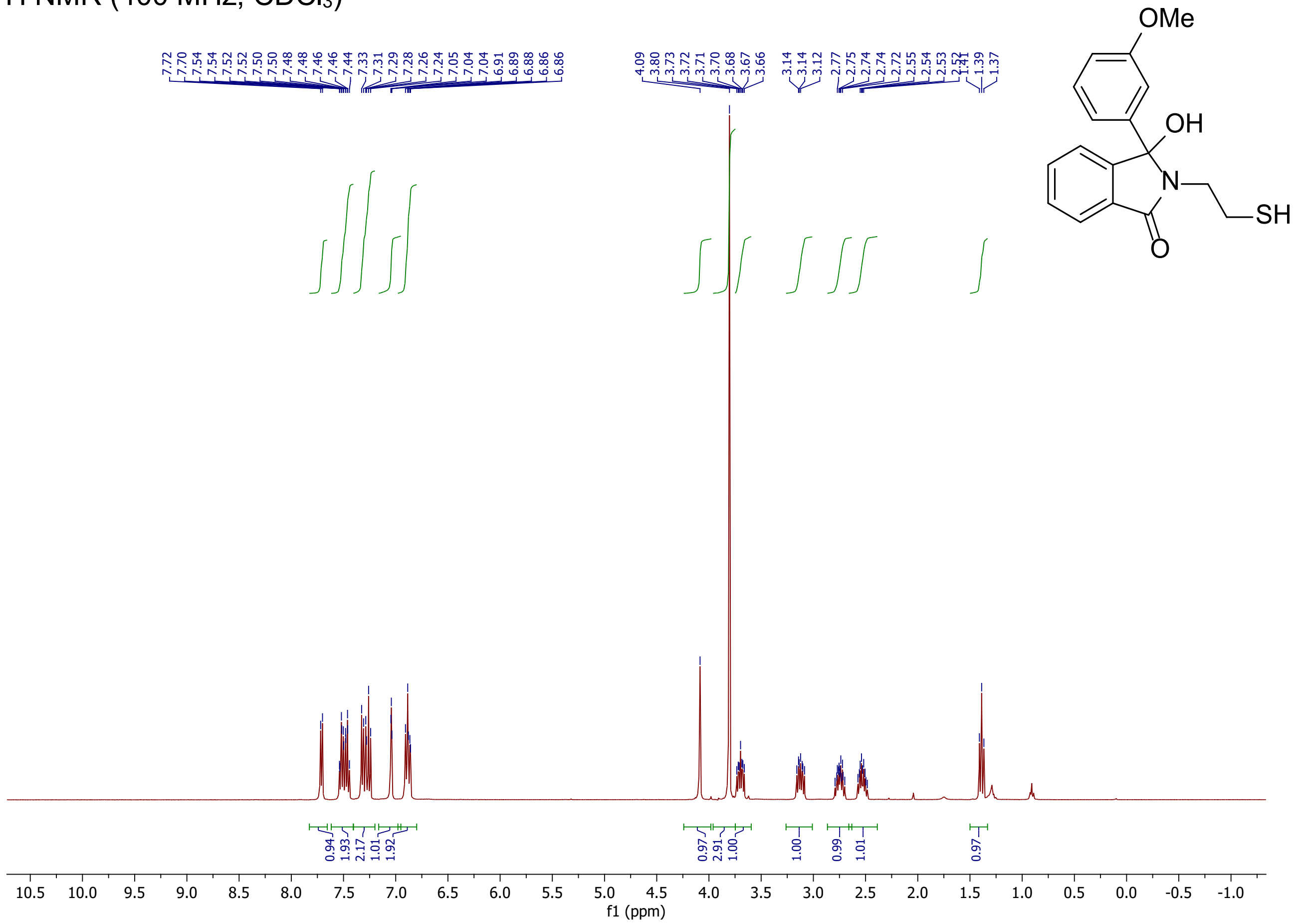

^{13}C NMR (101 MHz, $CDCl_3$)

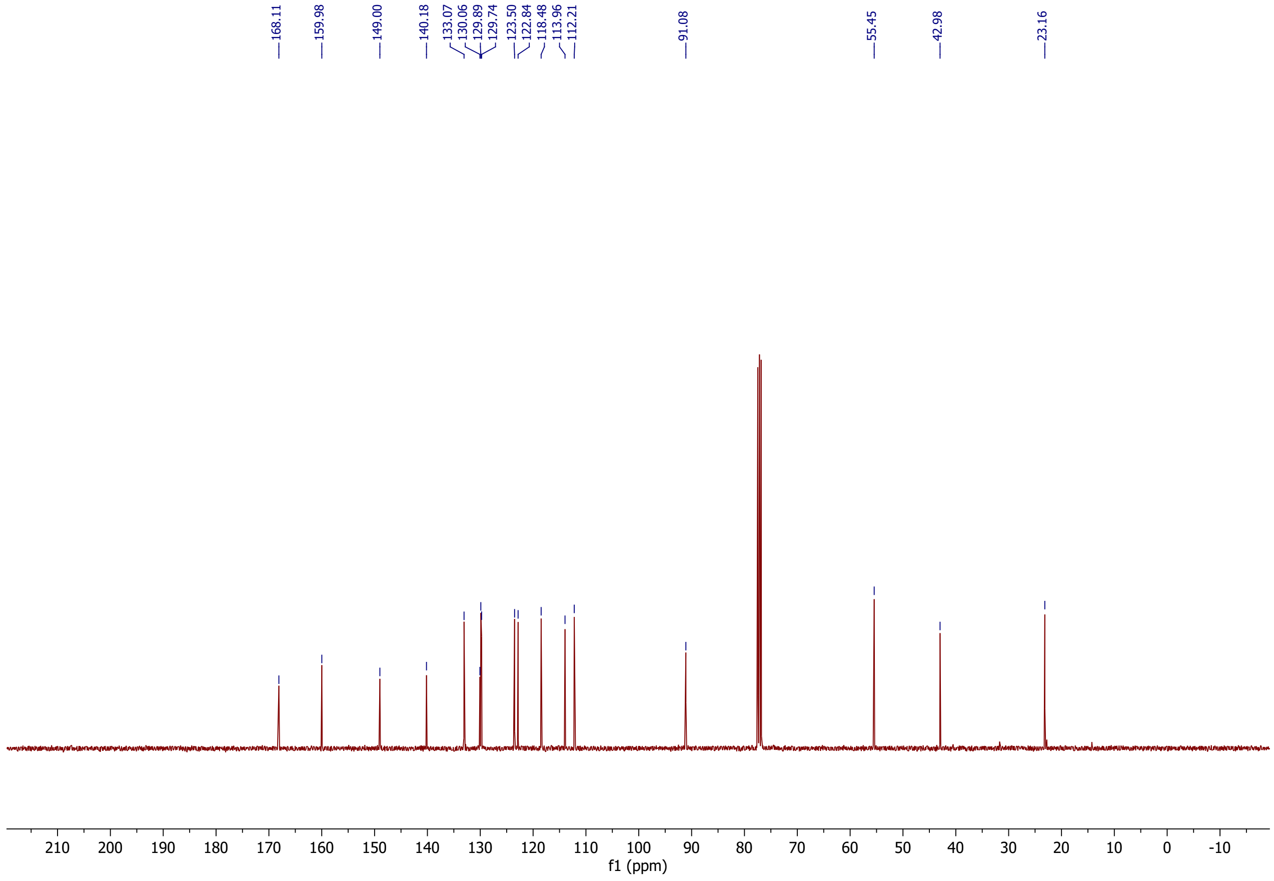

Figure S. 7. 3-[3,5-bis(trifluoromethyl)phenyl]-3-hydroxy-2-(2-sulfanylethyl)isoindolin-1-one (1f)

^{1}H NMR (400 MHz, $CDCl_3$)

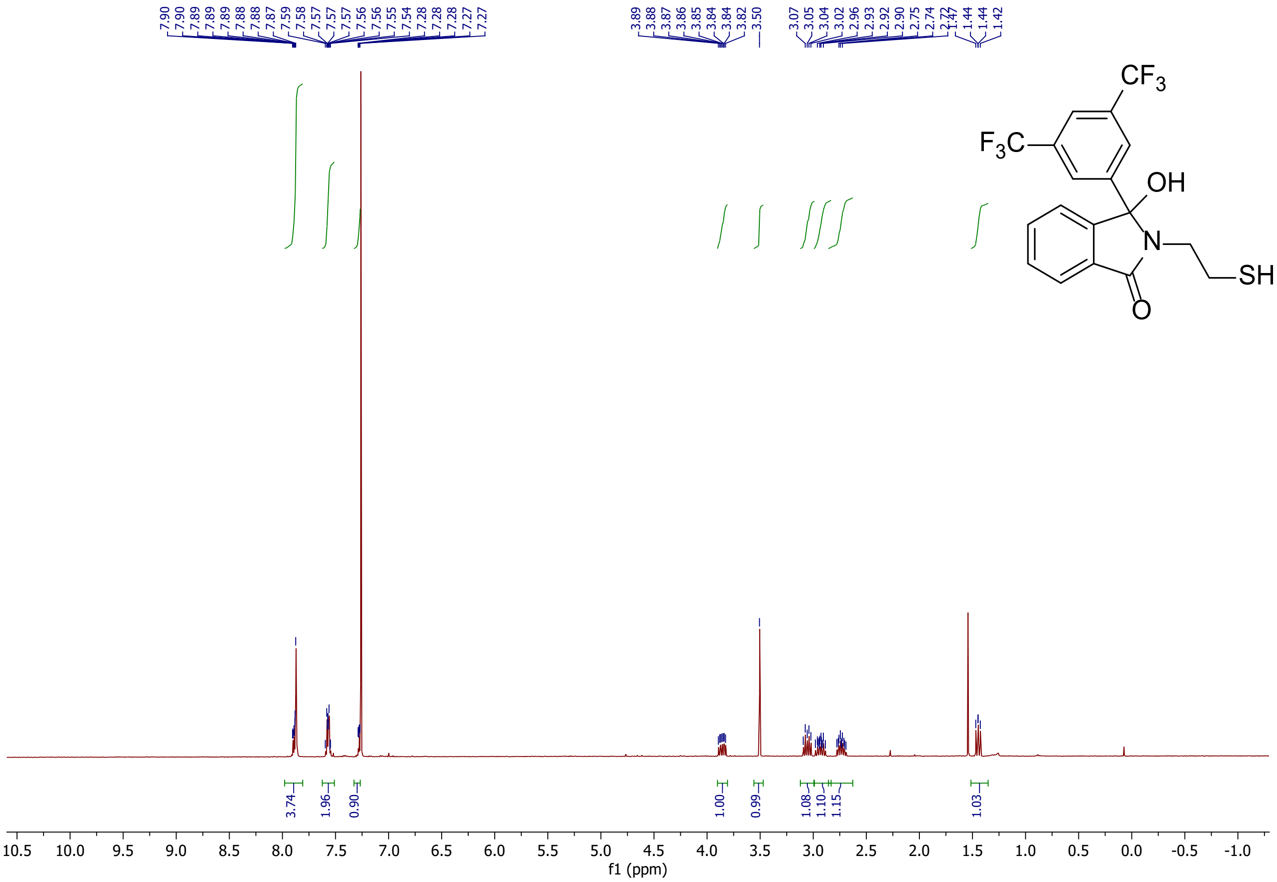

^{13}C NMR (101 MHz, $CDCl_3$)

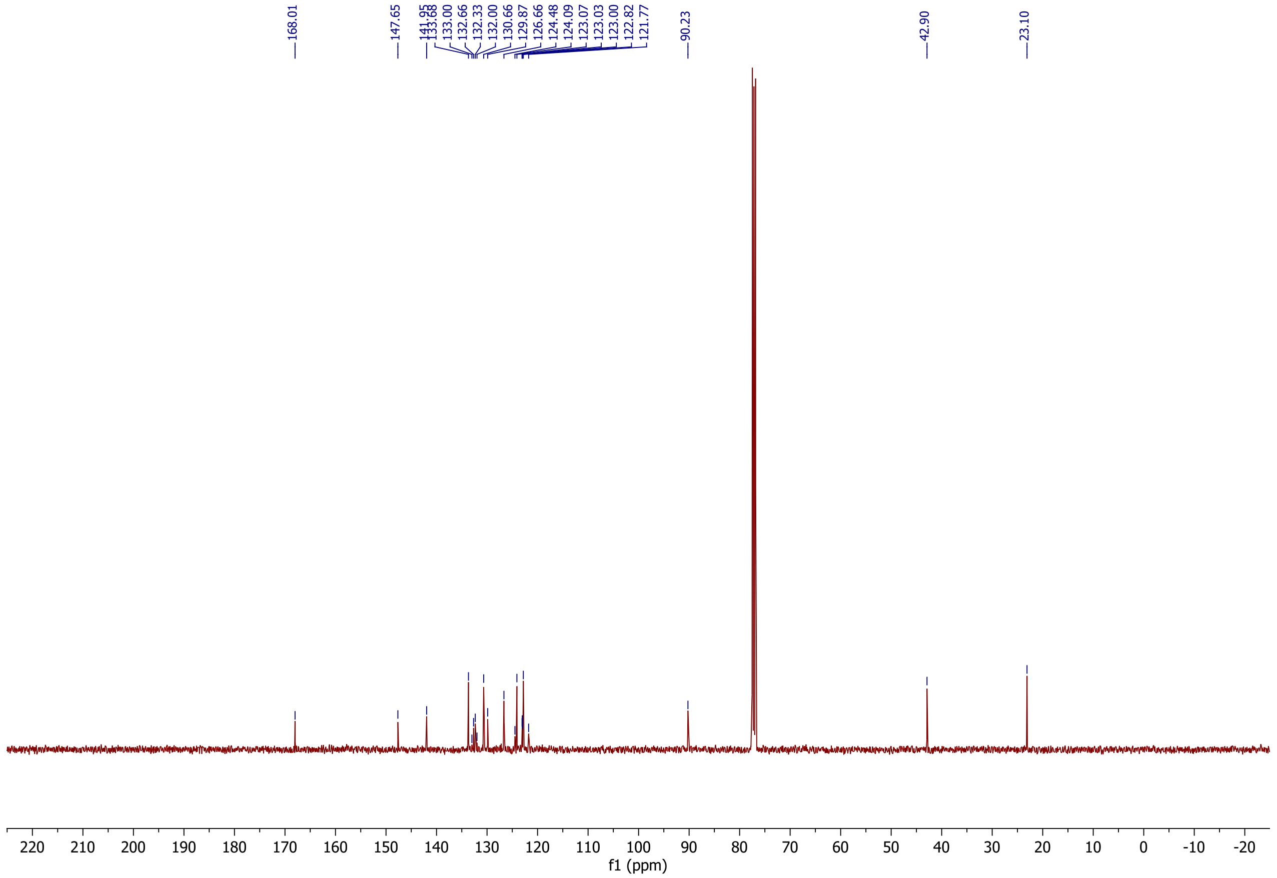

Figure S. 8. 3-(2,4-dimethoxyphenyl)-3-hydroxy-2-(2-sulfanylethyl)isoindolin-1-one (1g)

^{1}H NMR (400 MHz, DMSO-d$_6$)

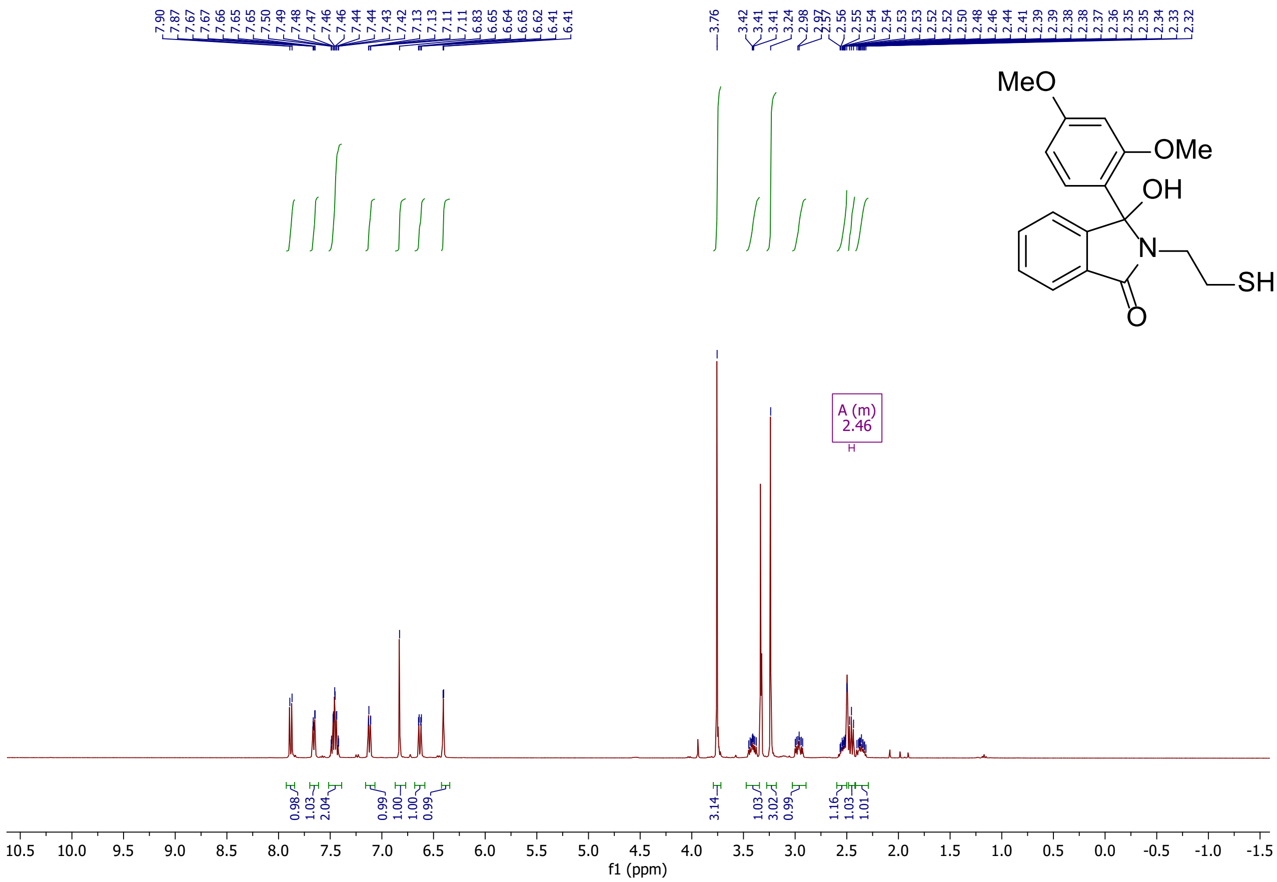

^{13}C NMR (101 MHz, $CDCl_3$)

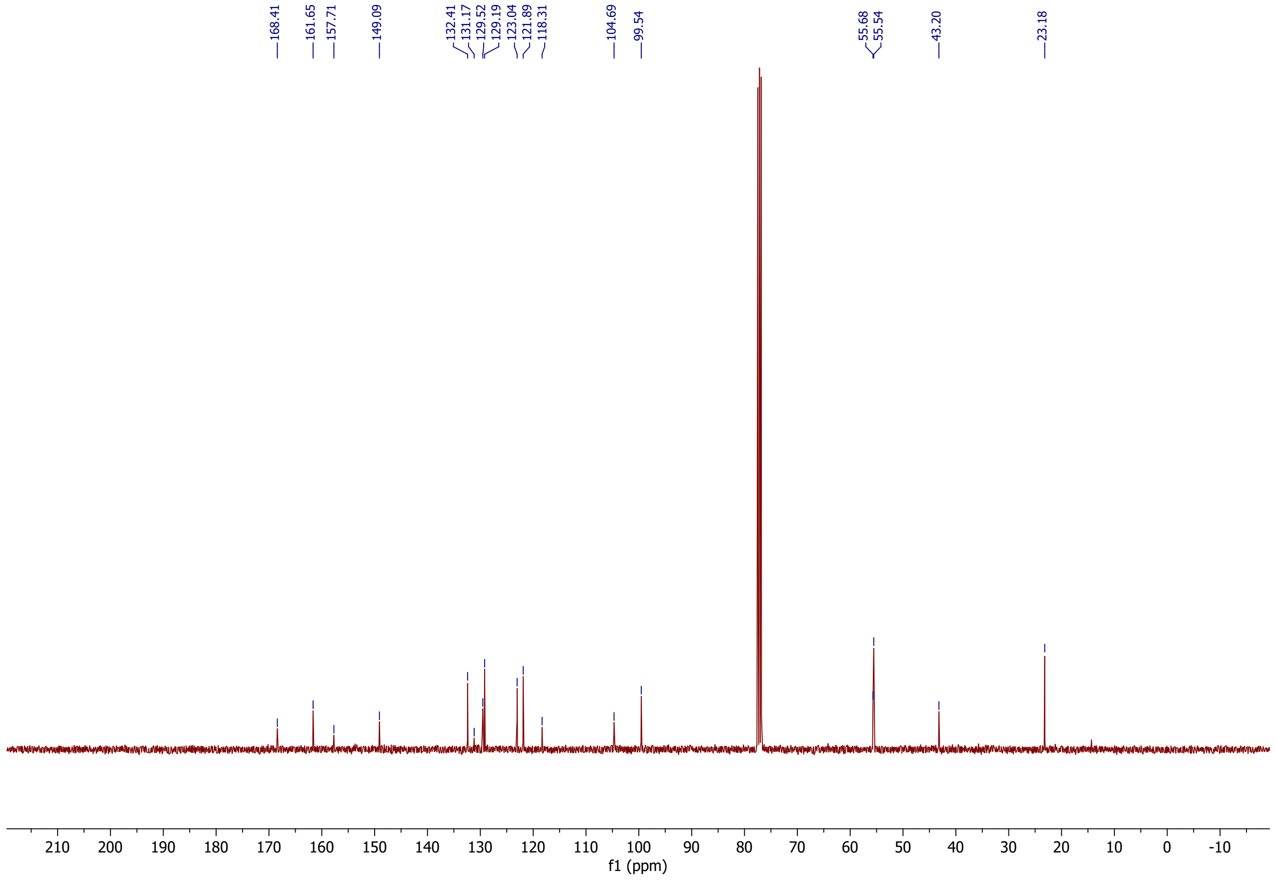

Figure S. 9. 3-(1,3-benzodioxol-5-yl)-3-hydroxy-2-(2-sulfanylethyl)isoindolin-1-one (1h)
^{1}H NMR (400 MHz, $CDCl_3$)

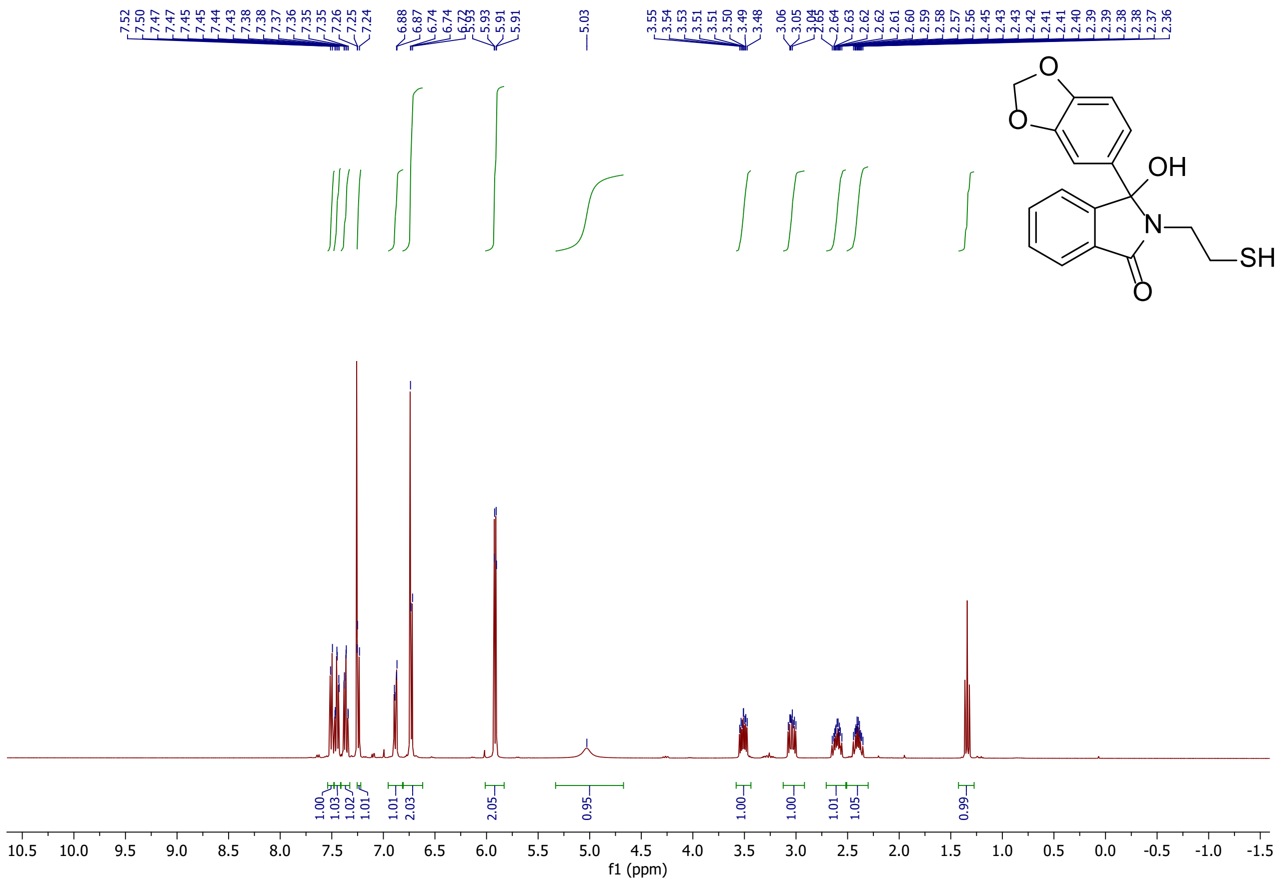

^{13}C NMR (101 MHz, $CDCl_3$)

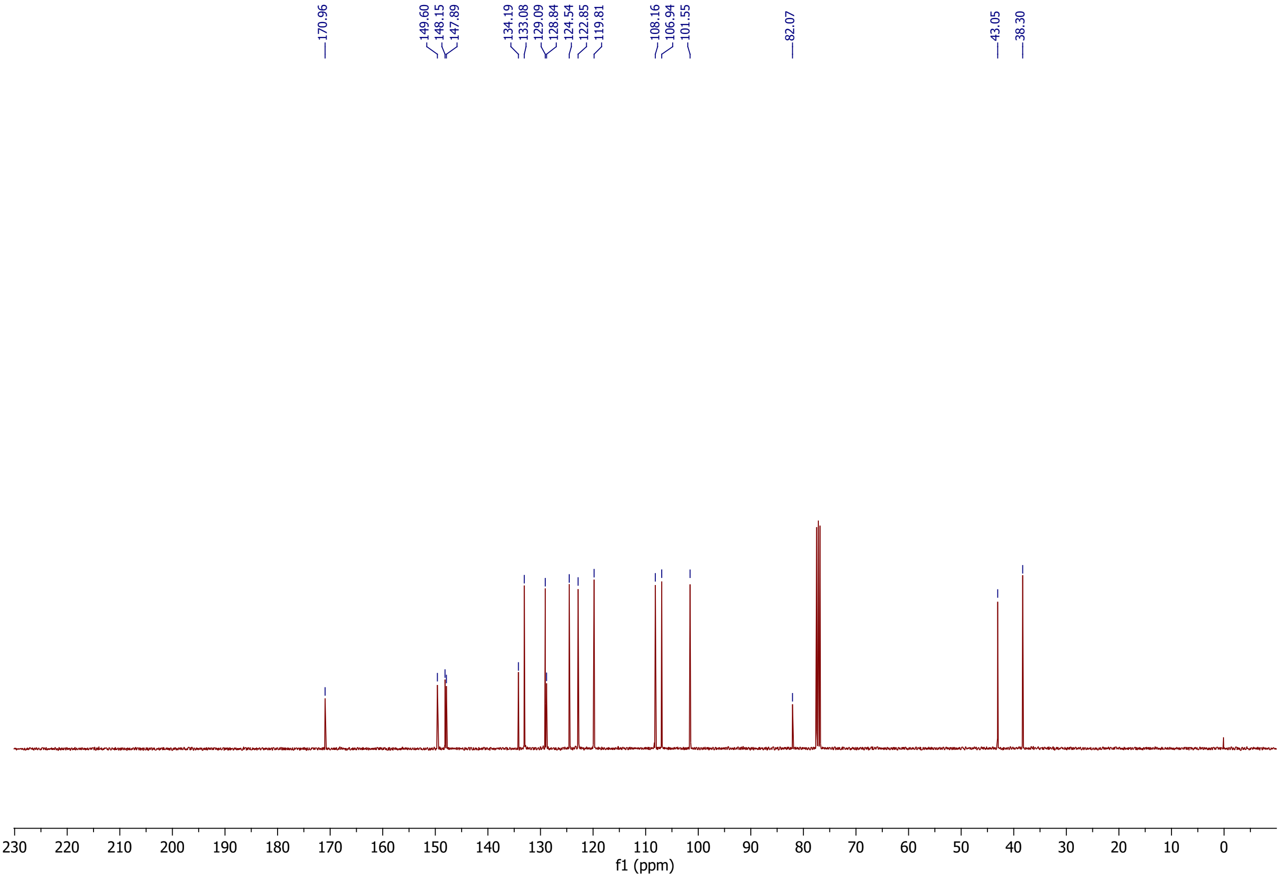

Figure S. 10. 3-hydroxy-3-(4-morpholinophenyl)-2-(2-sulfanylethyl)isoindolin-1-one (1i)

^{1}H NMR (400 MHz, $CDCl_3$)

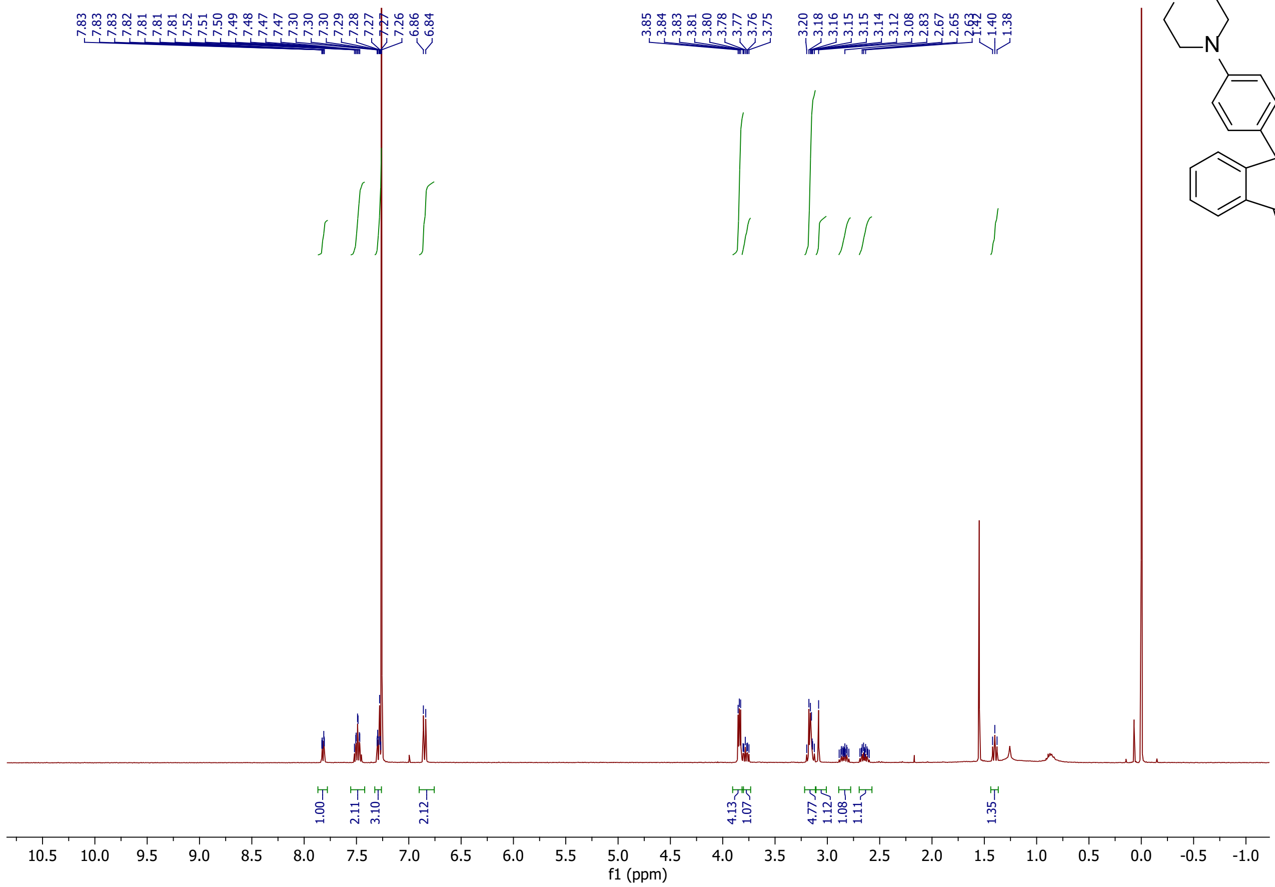

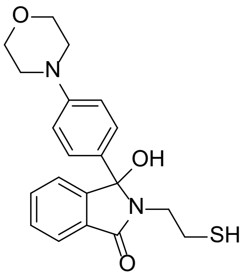

^{13}C NMR (101 MHz, $CDCl_3$)

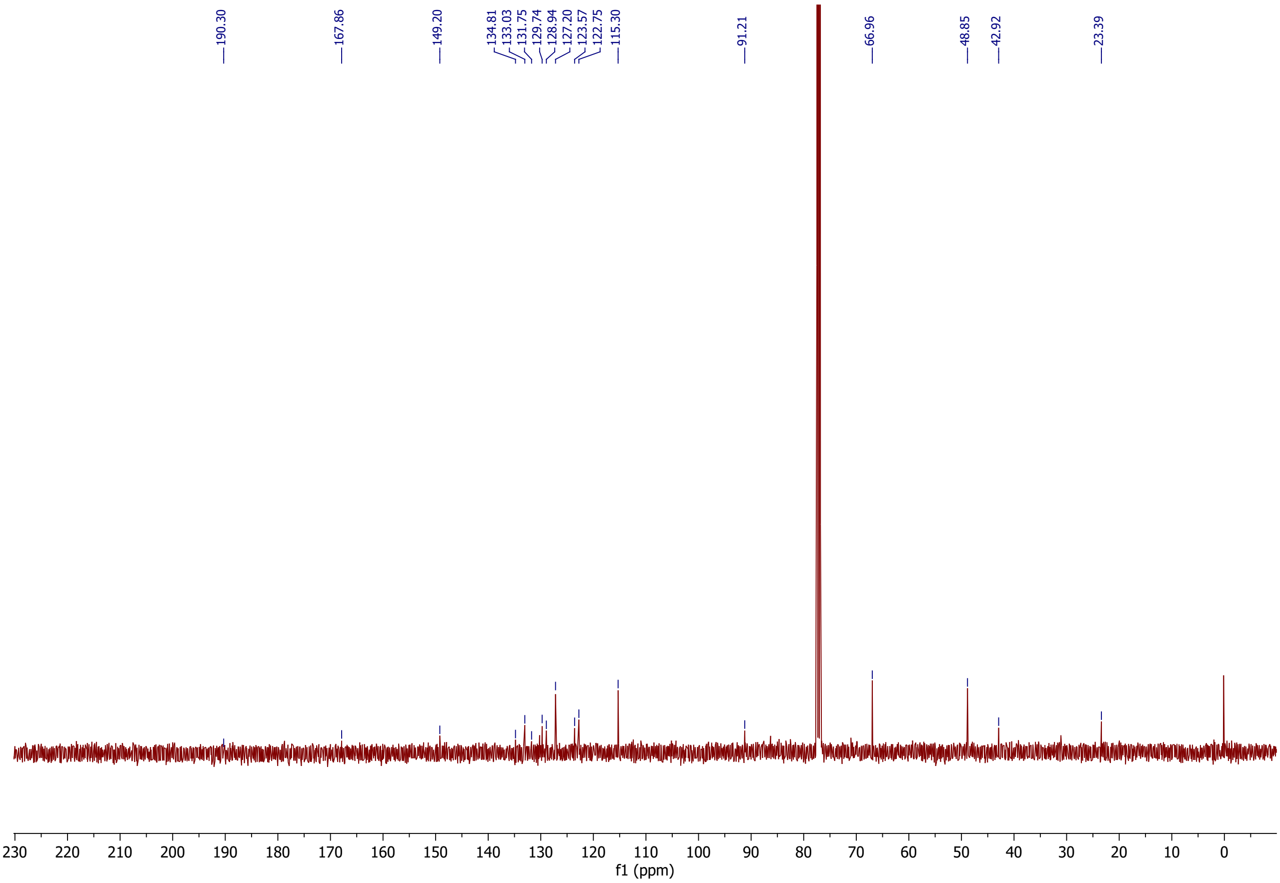

Figure S. 11. 9b-phenyl-2,3-dihydrothiazolo[2,3-a]isoindol-5-one (2a)

^{1}H NMR (400 MHz, $CDCl_3$)

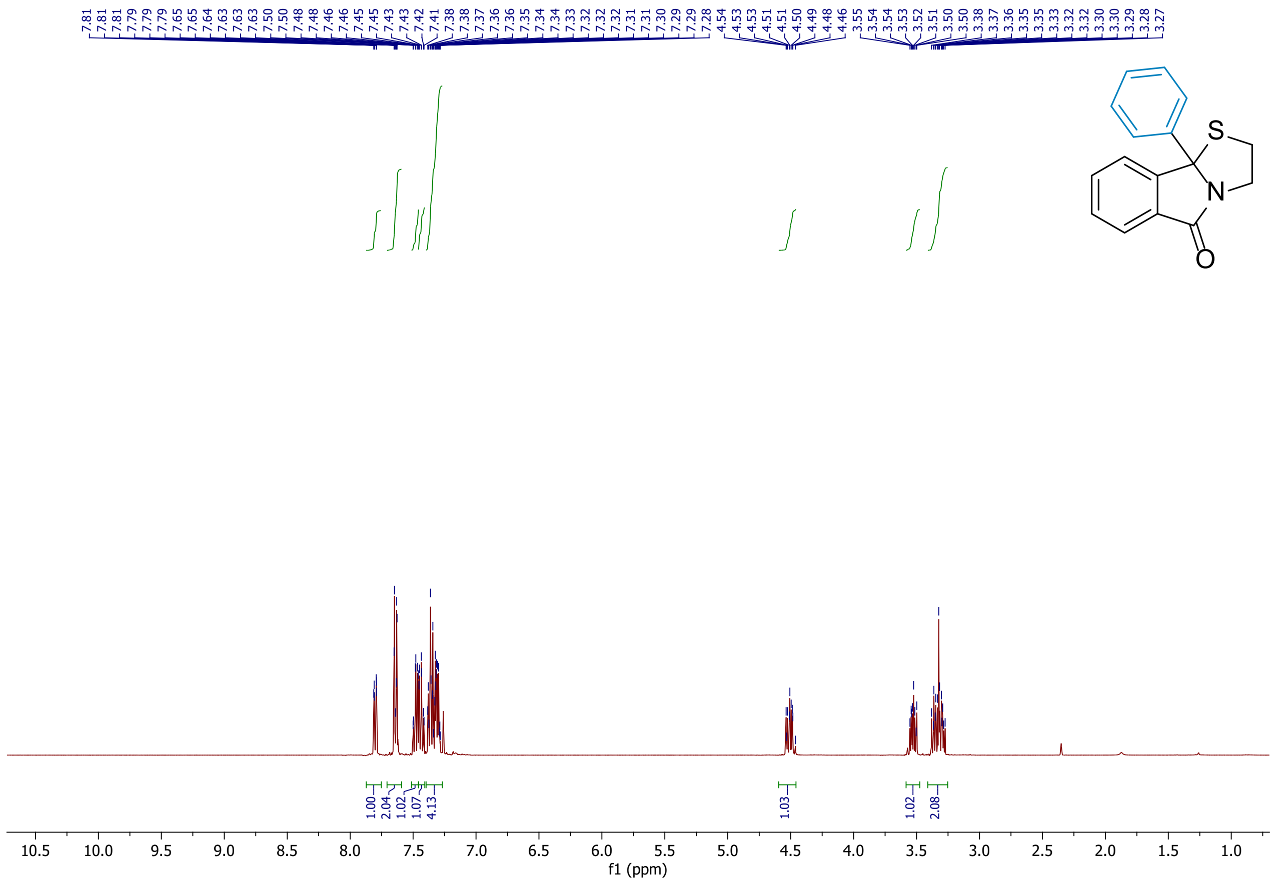

^{13}C NMR (101 MHz, $CDCl_3$)

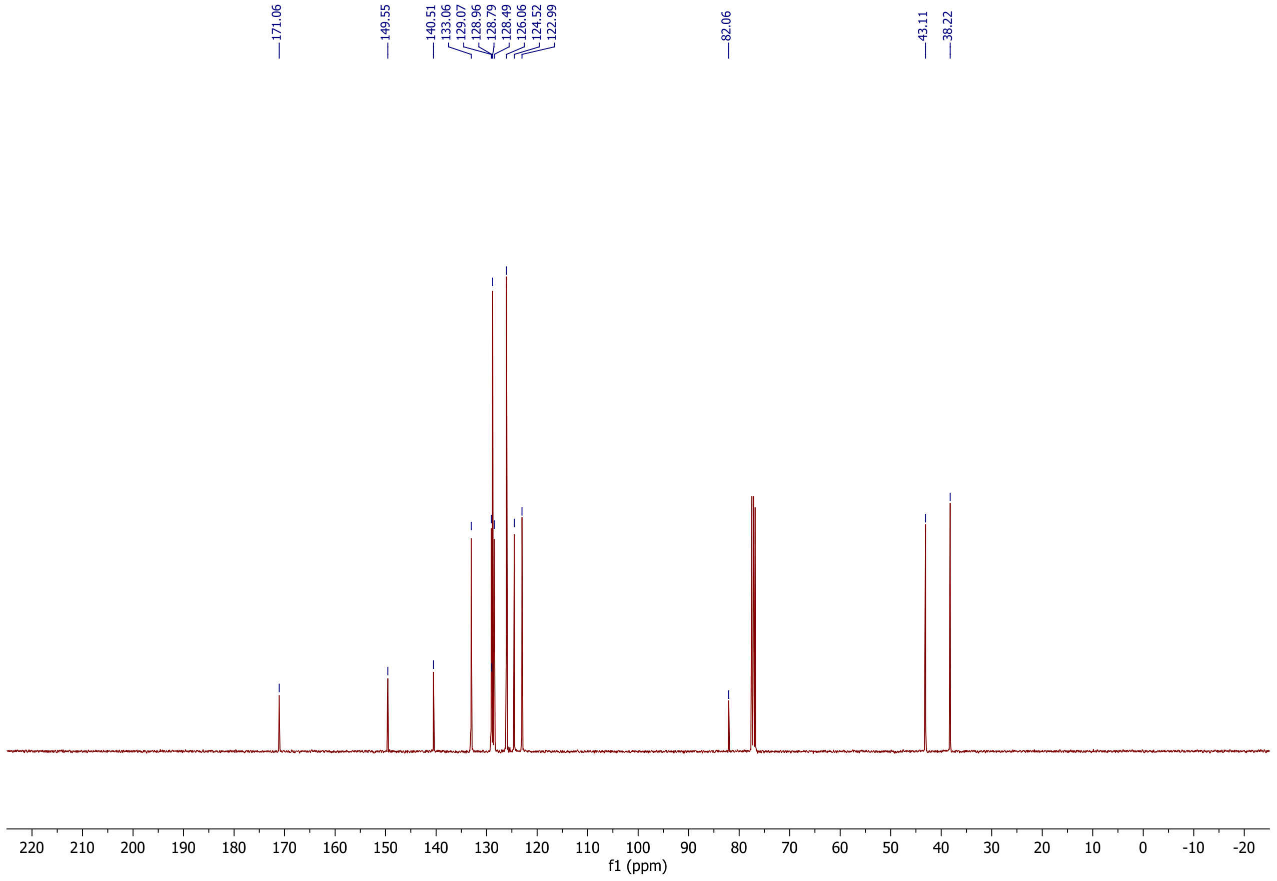

Figure S. 12. 9b-(4-chlorophenyl)-2,3-dihydrothiazolo[2,3-a]isoindol-5-one (2b)

^{1}H NMR (400 MHz, $CDCl_3$)

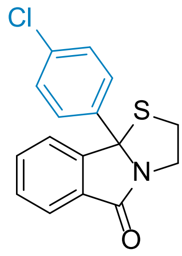

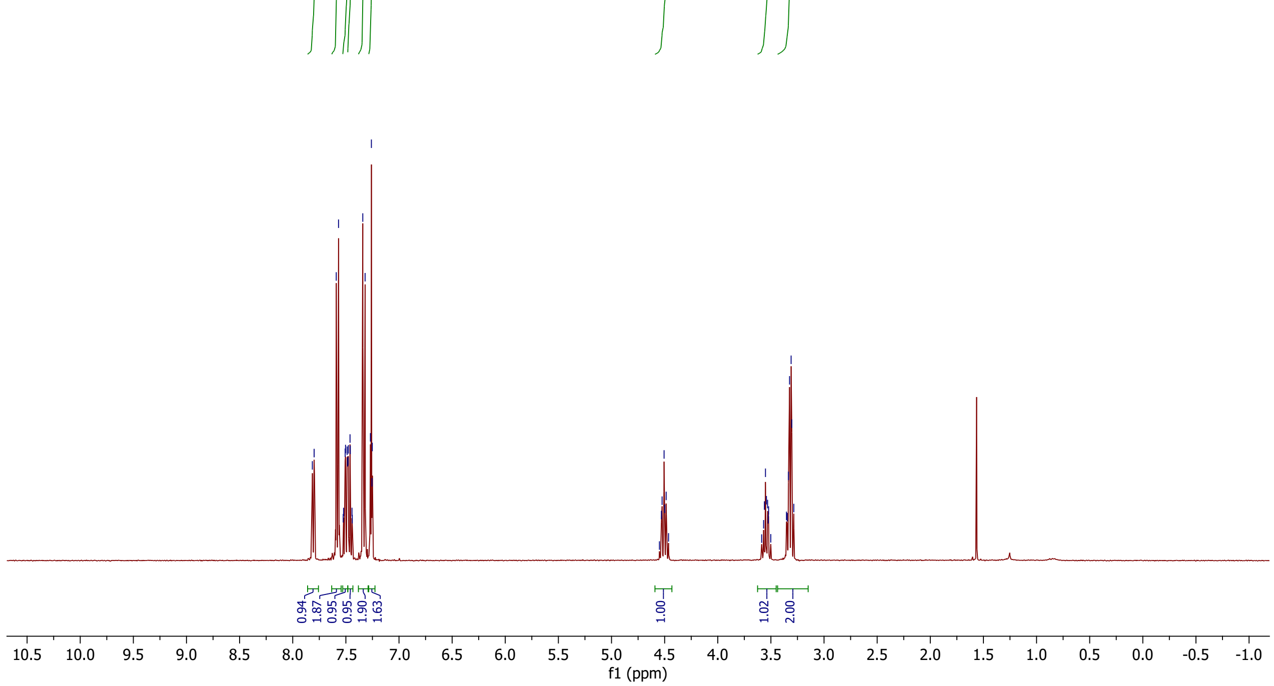

^{13}C NMR (101 MHz, $CDCl_3$)

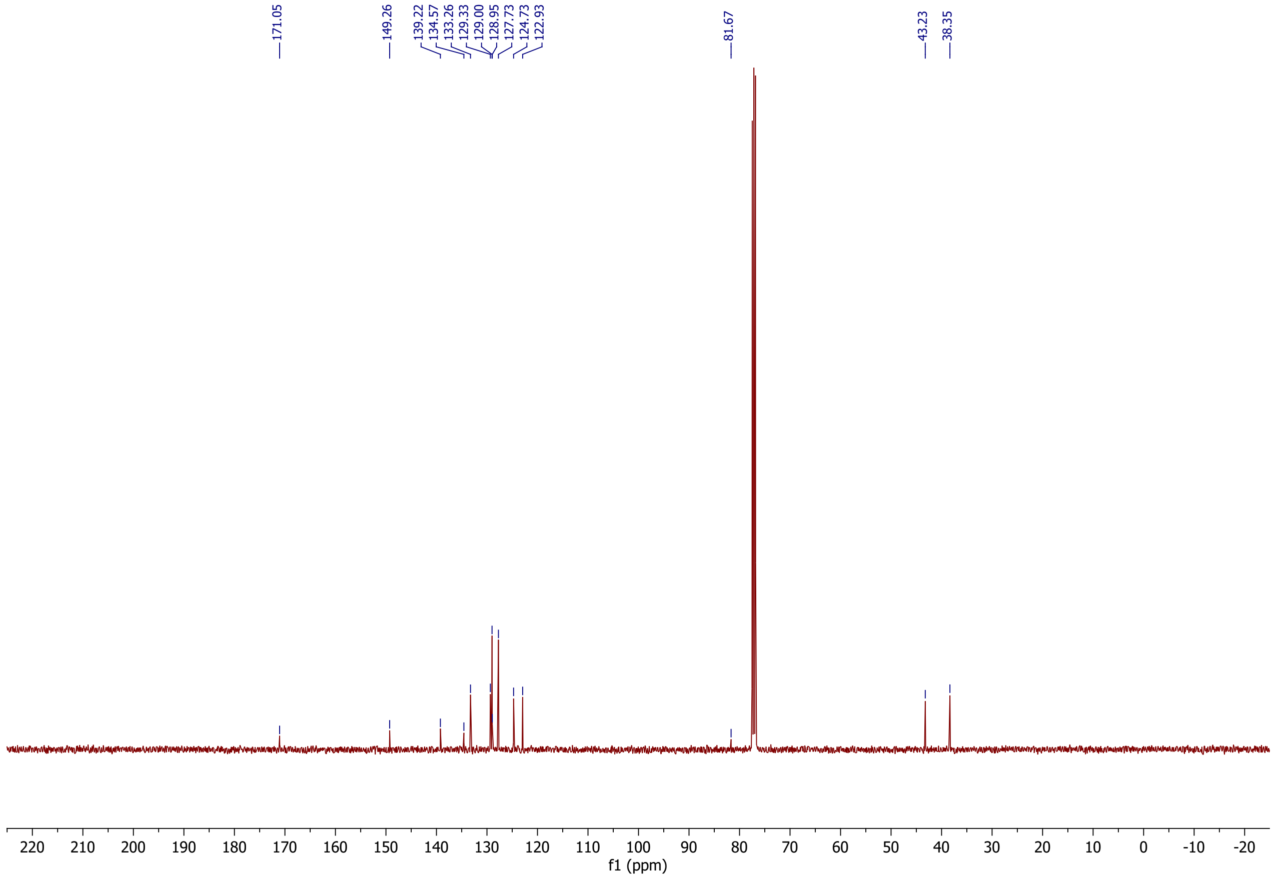

Figure S. 13. 9b-[4-(trifluoromethyl)phenyl]-2,3-dihydrothiazolo[2,3-a]isoindol-5-one (2c)

^{1}H NMR (400 MHz, $CDCl_3$)

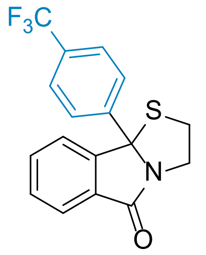

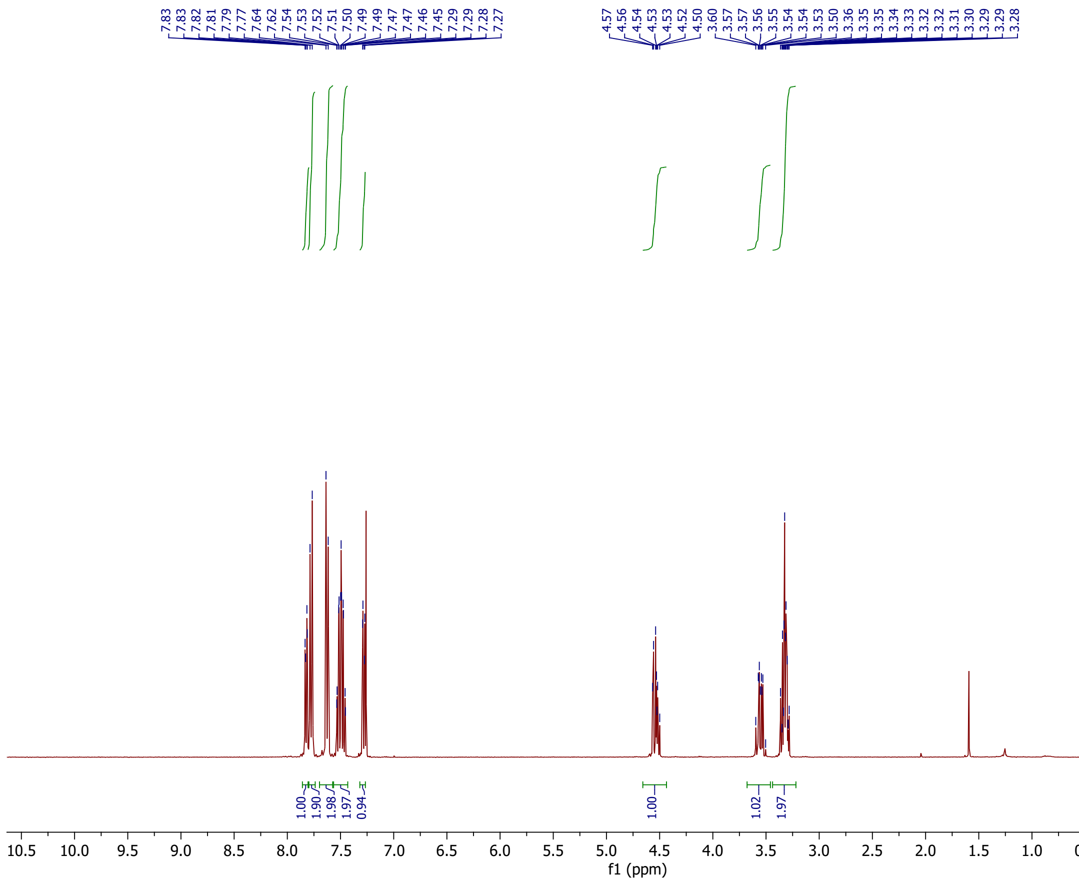

^{13}C NMR (101 MHz, $CDCl_3$)

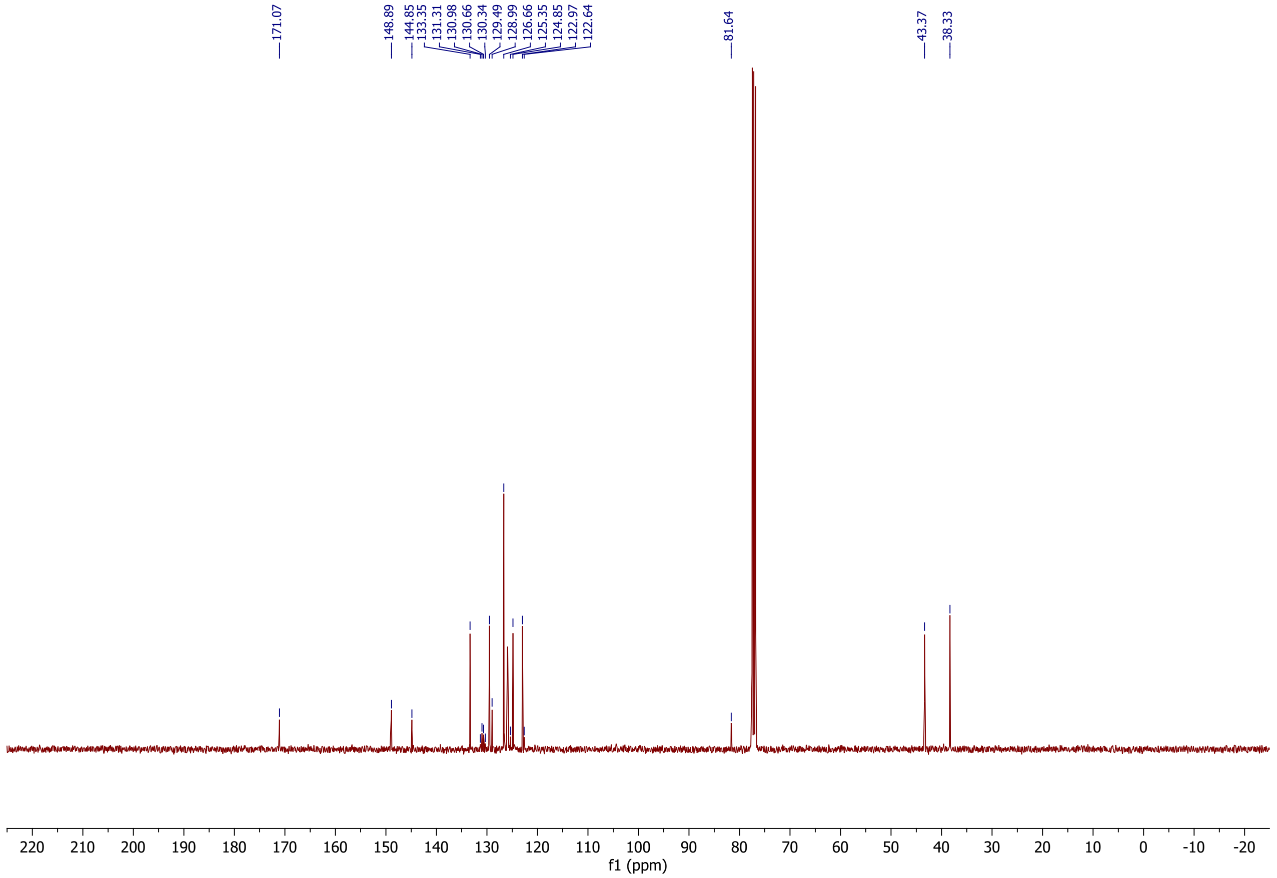

Figure S. 14. 9b-(4-methoxyphenyl)-2,3-dihydrothiazolo[2,3-a]isoindol-5-one (2d)

^{1}H NMR (400 MHz, $CDCl_3$)

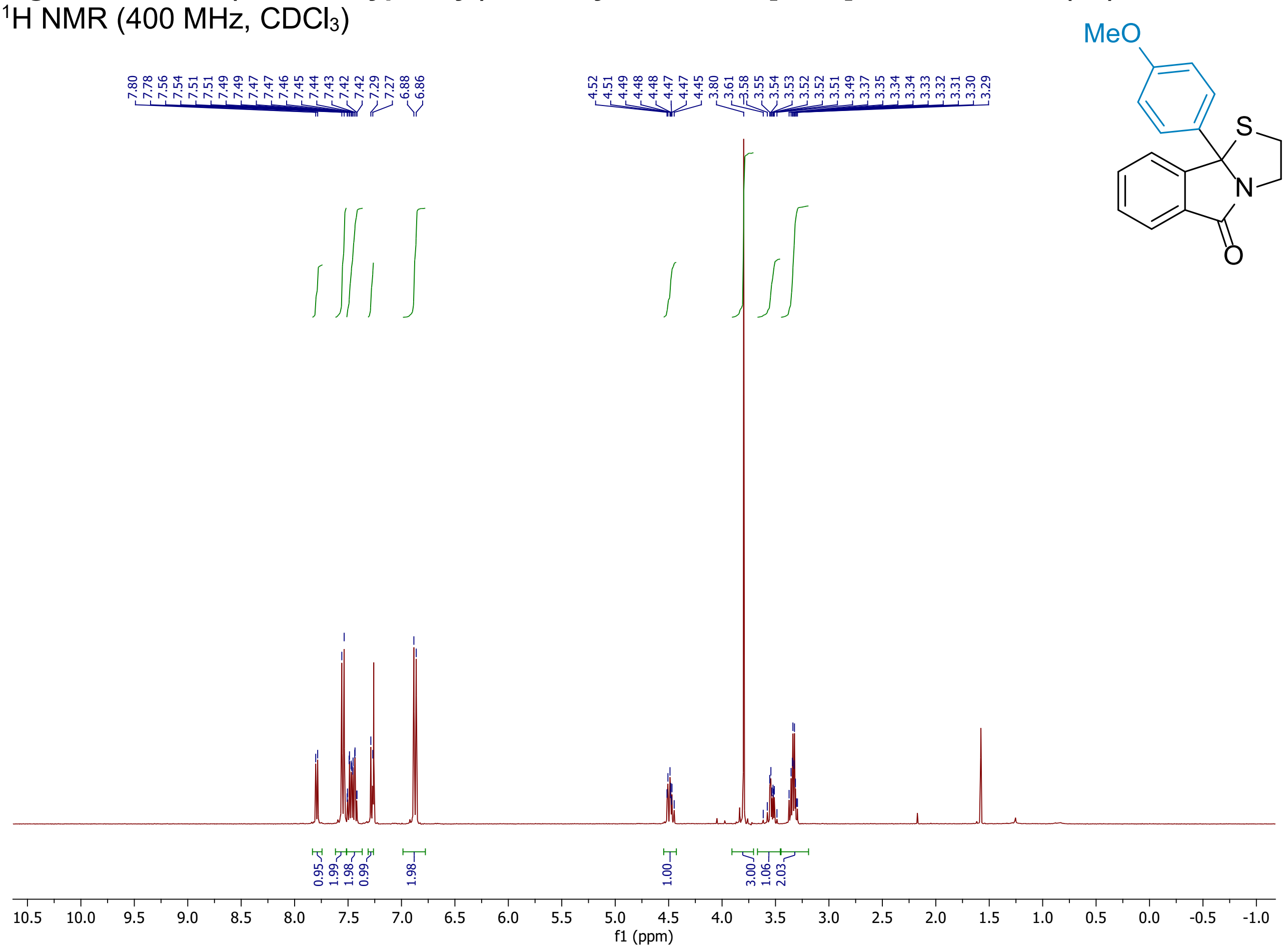

^{13}C NMR (101 MHz, $CDCl_3$)

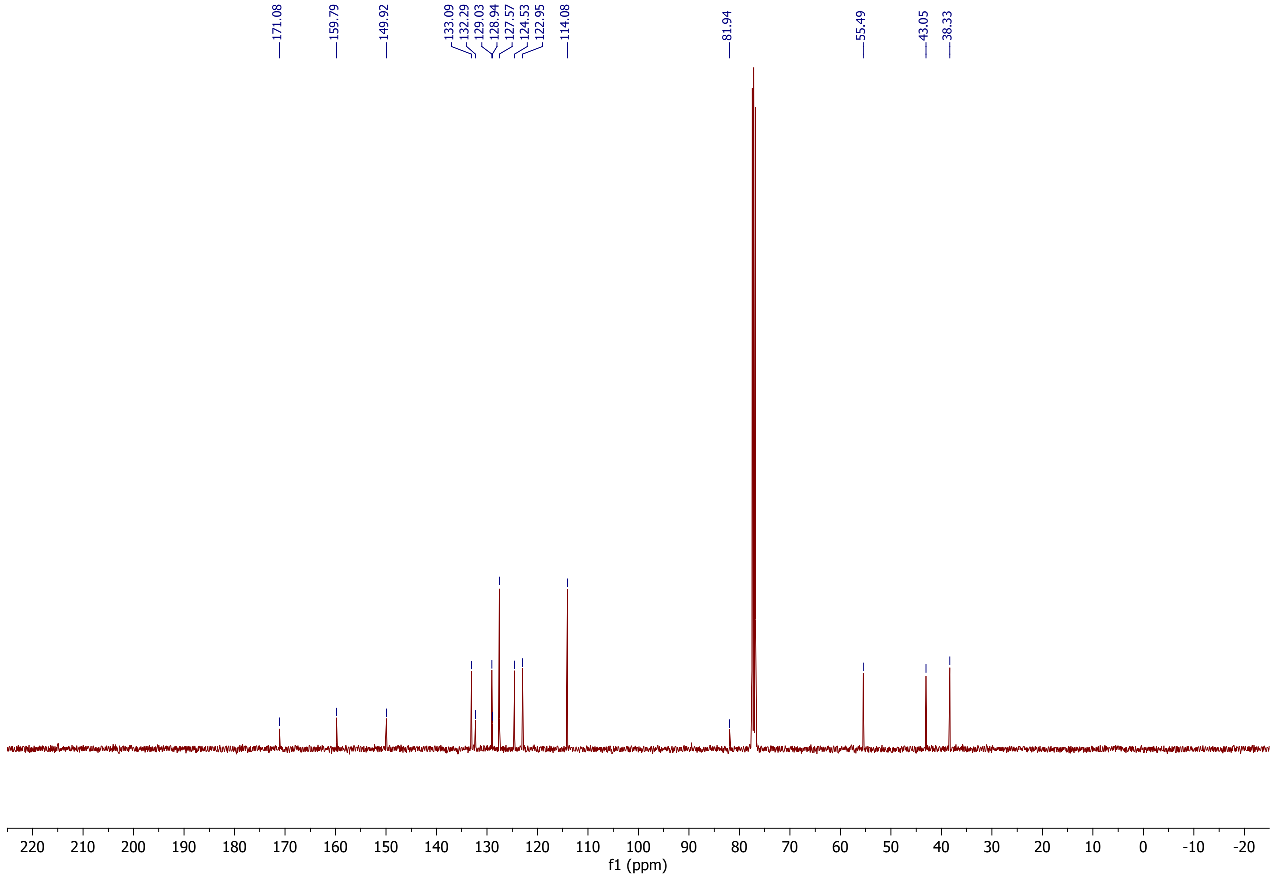

Figure S. 15. 9b-(3-methoxyphenyl)-2,3-dihydrothiazolo[2,3-a]isoindol-5-one (2e)

^{1}H NMR (400 MHz, $CDCl_3$)

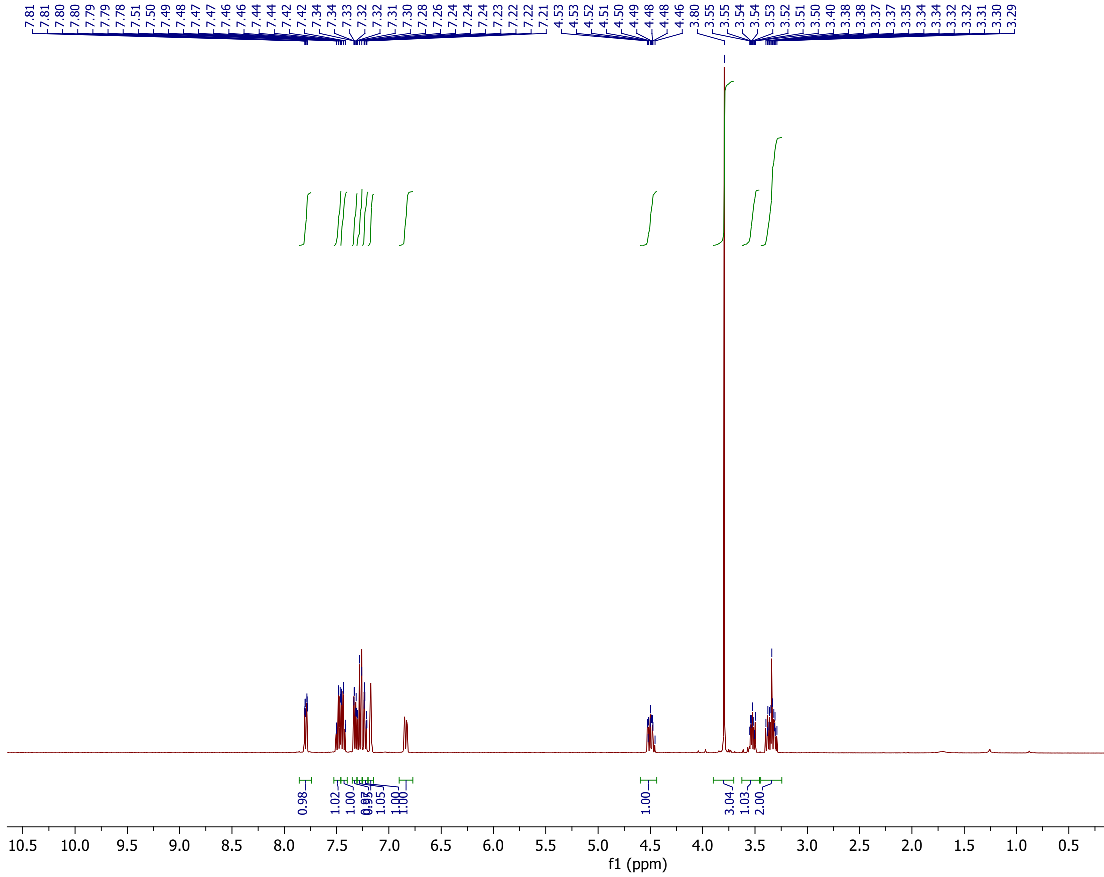

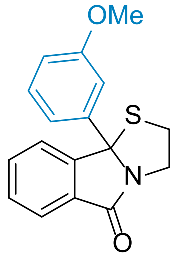

^{13}C NMR (101 MHz, $CDCl_3$)

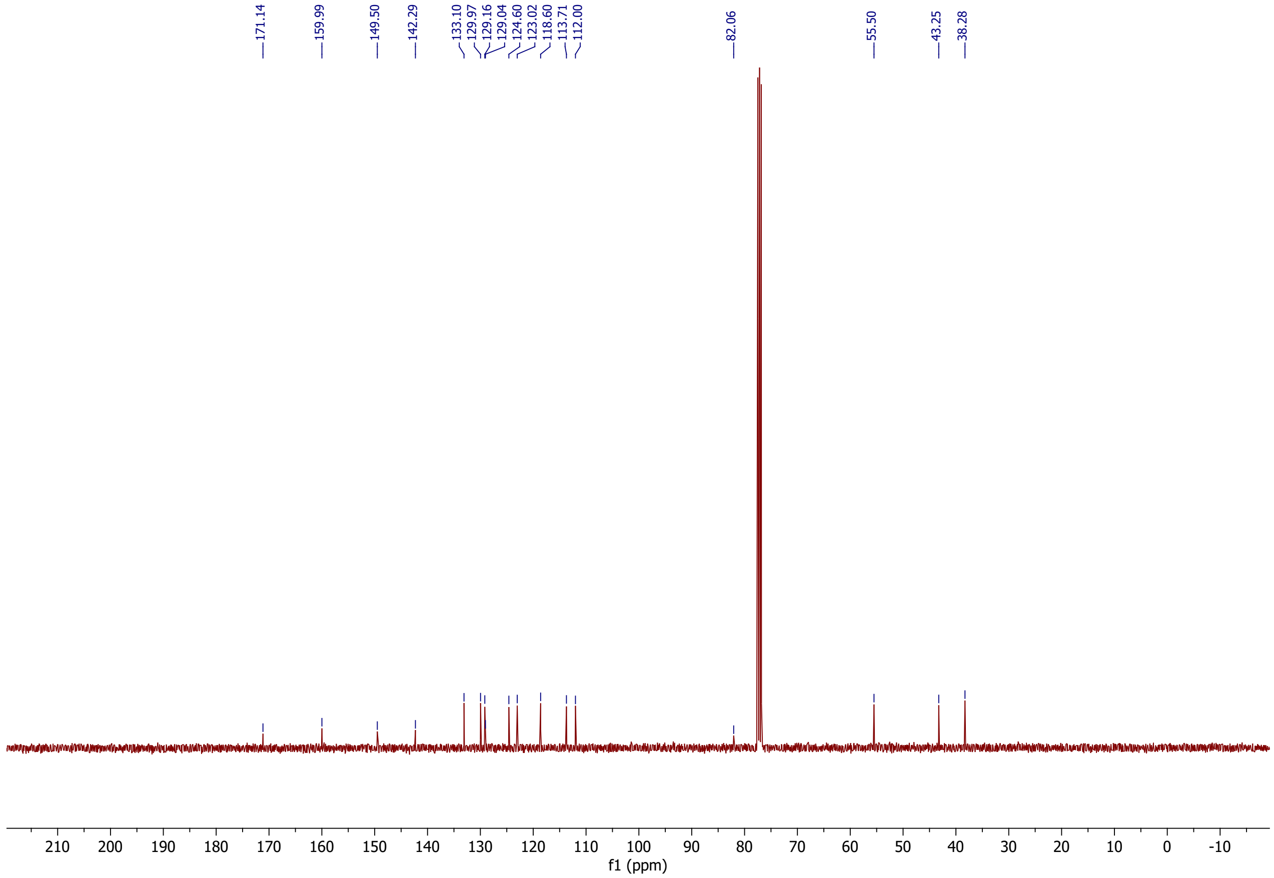

Figure S. 16. 9b-[3,5-bis(trifluoromethyl)phenyl]-2,3-dihydrothiazolo[2,3-a]isoindol-5-one (2f)

^{1}H NMR (400 MHz, $CDCl_3$)

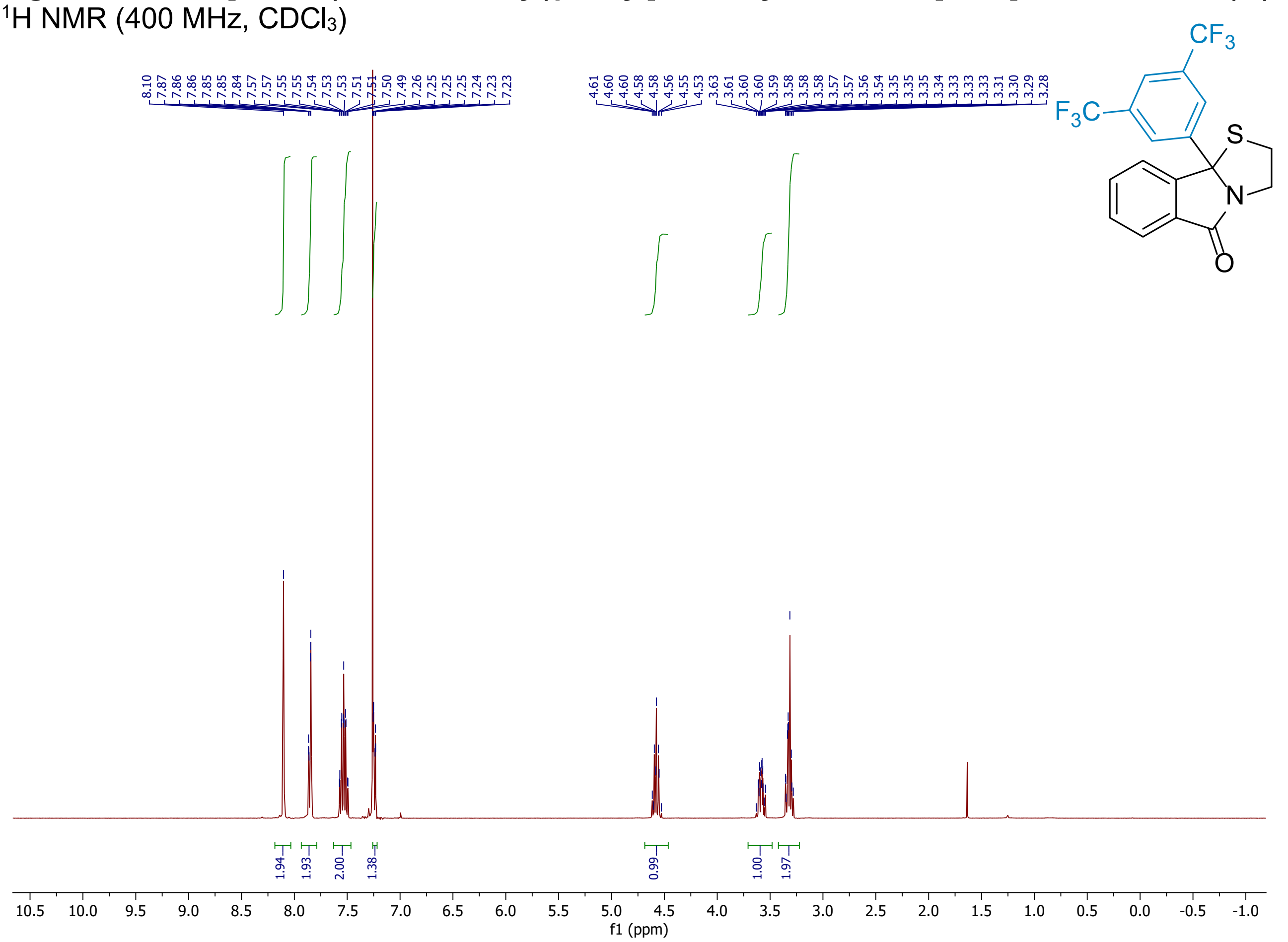

^{13}C NMR (101 MHz, $CDCl_3$)

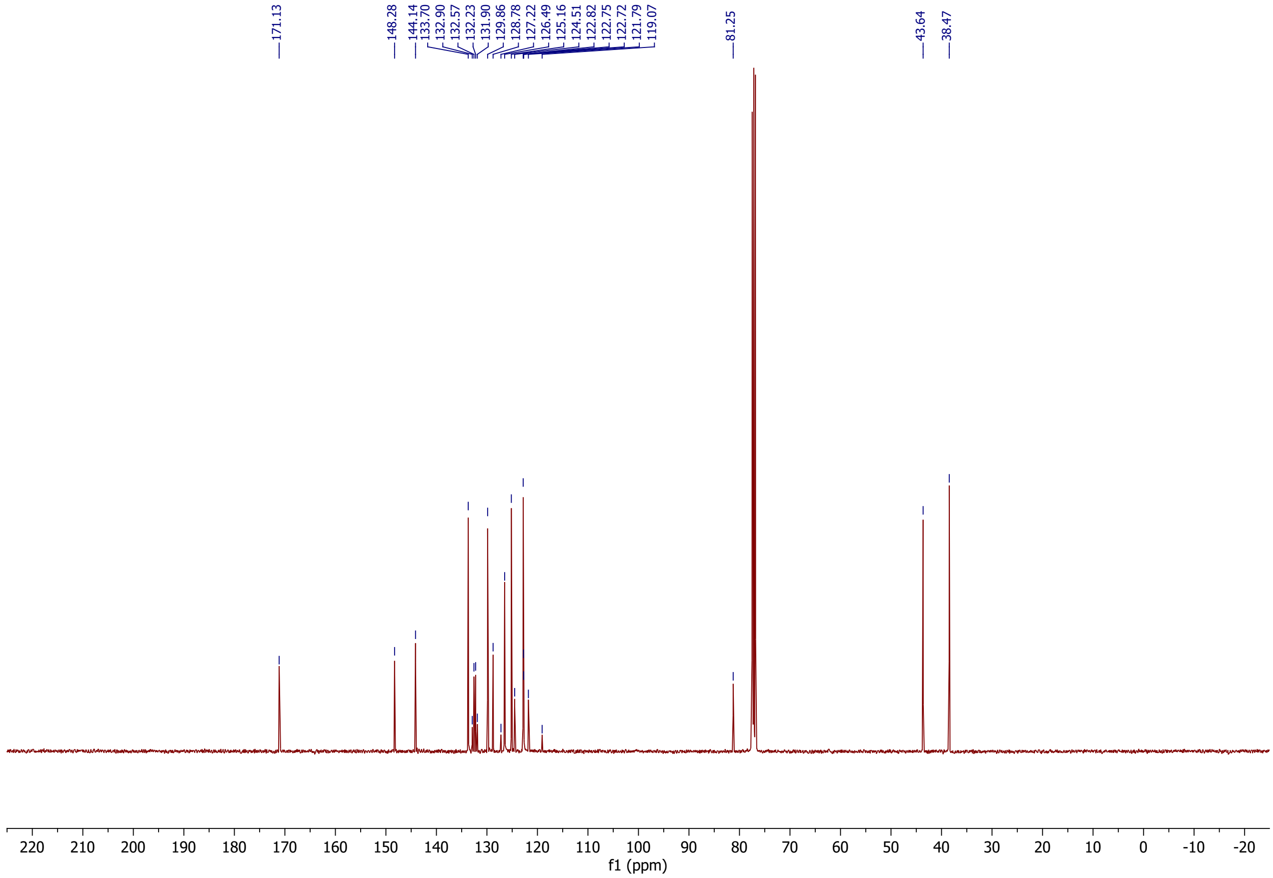

Figure S. 17. 9b-(2,4-dimethoxyphenyl)-2,3-dihydrothiazolo[2,3-a]isoindol-5-one (2g)

^{1}H NMR (400 MHz, $CDCl_3$)

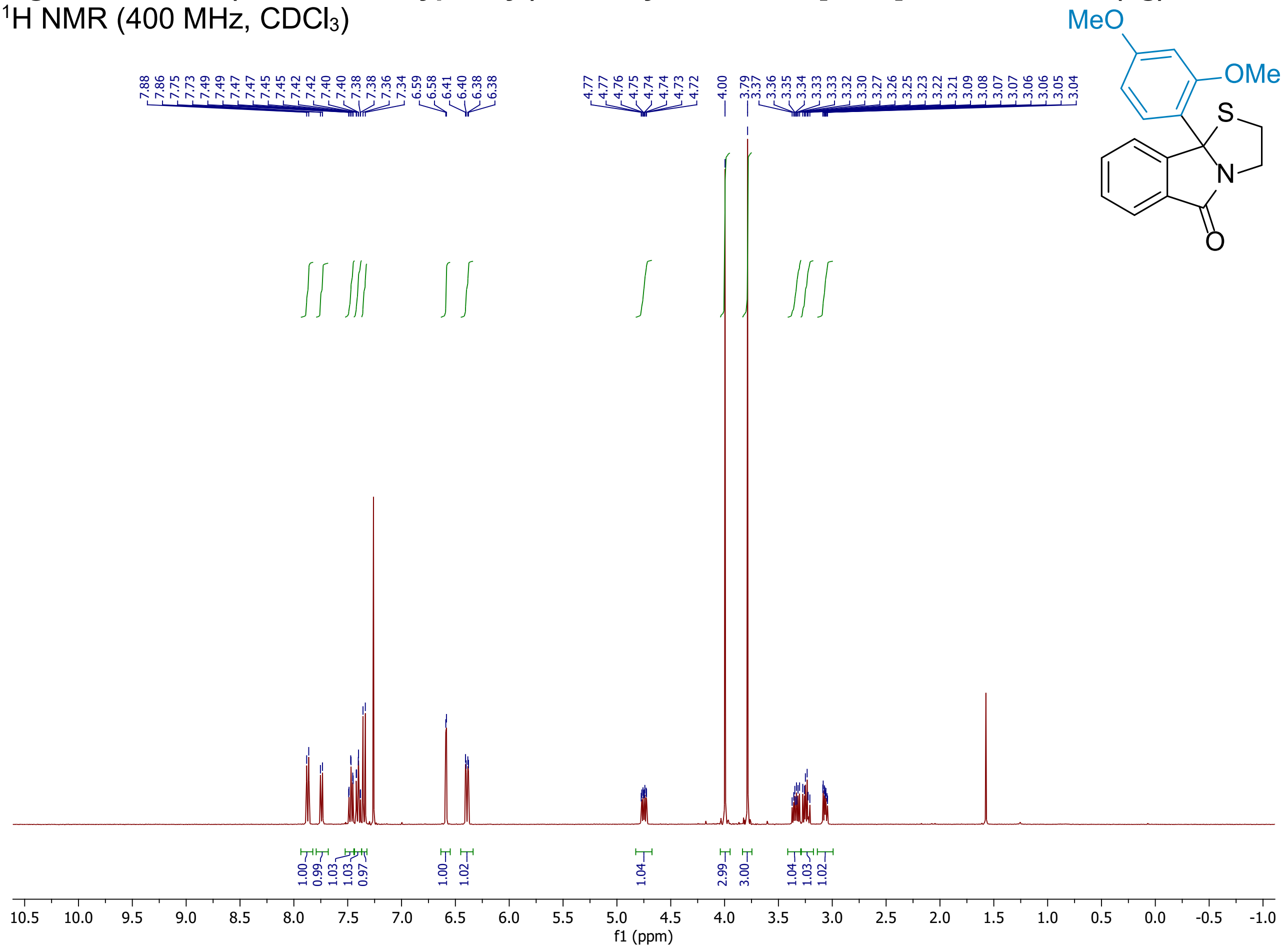

^{13}C NMR (101 MHz, $CDCl_3$)

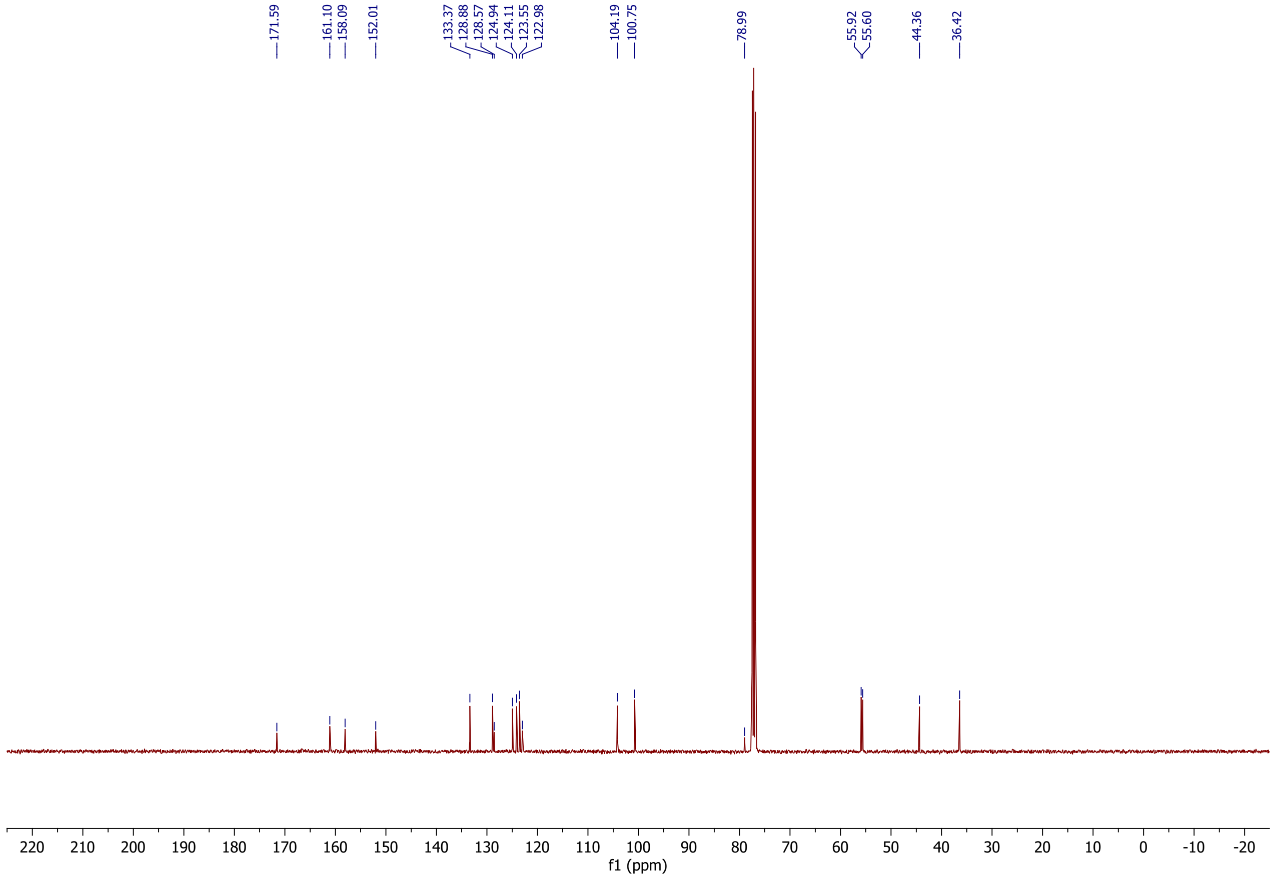

Figure S. 18. 9b-(1,3-benzodioxol-5-yl)-2,3-dihydrothiazolo[2,3-a]isoindol-5-one (2h)

^{1}H NMR (400 MHz, $CDCl_3$)

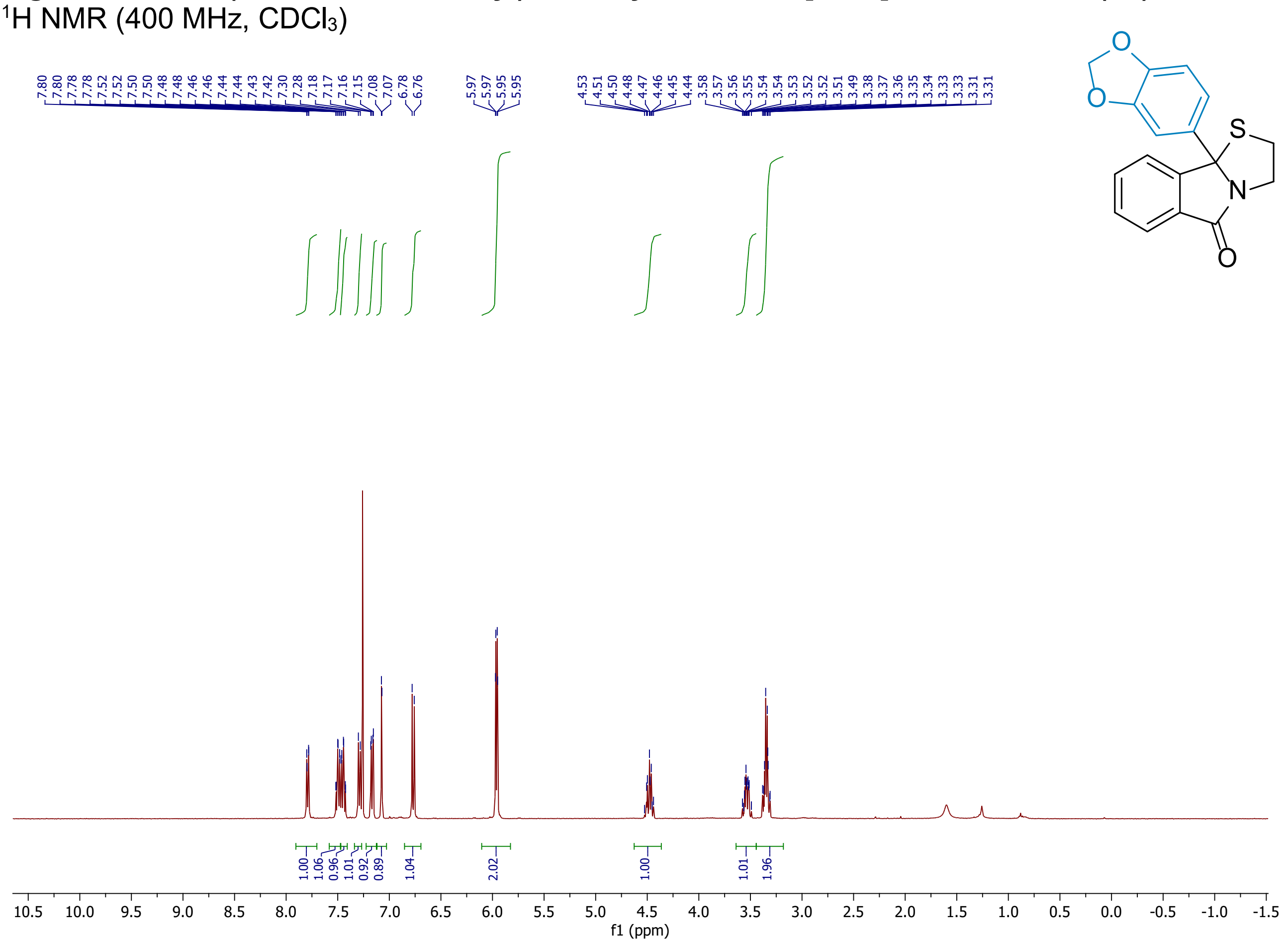

^{13}C NMR (101 MHz, $CDCl_3$)

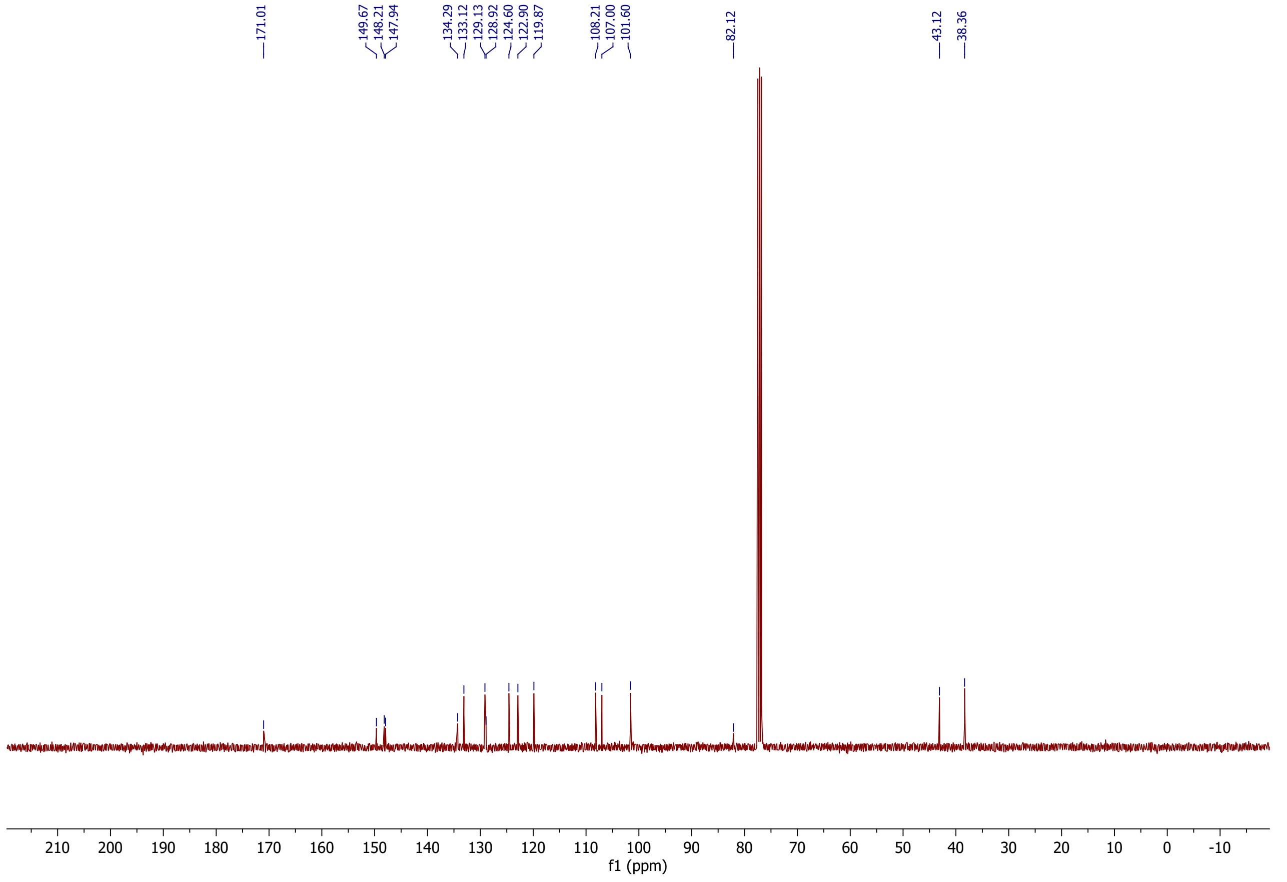

Figure S. 19. 9b-(4-morpholinophenyl)-2,3-dihydrothiazolo[2,3-a]isoindol-5-one (2i)

^{1}H NMR (400 MHz, $CDCl_3$)

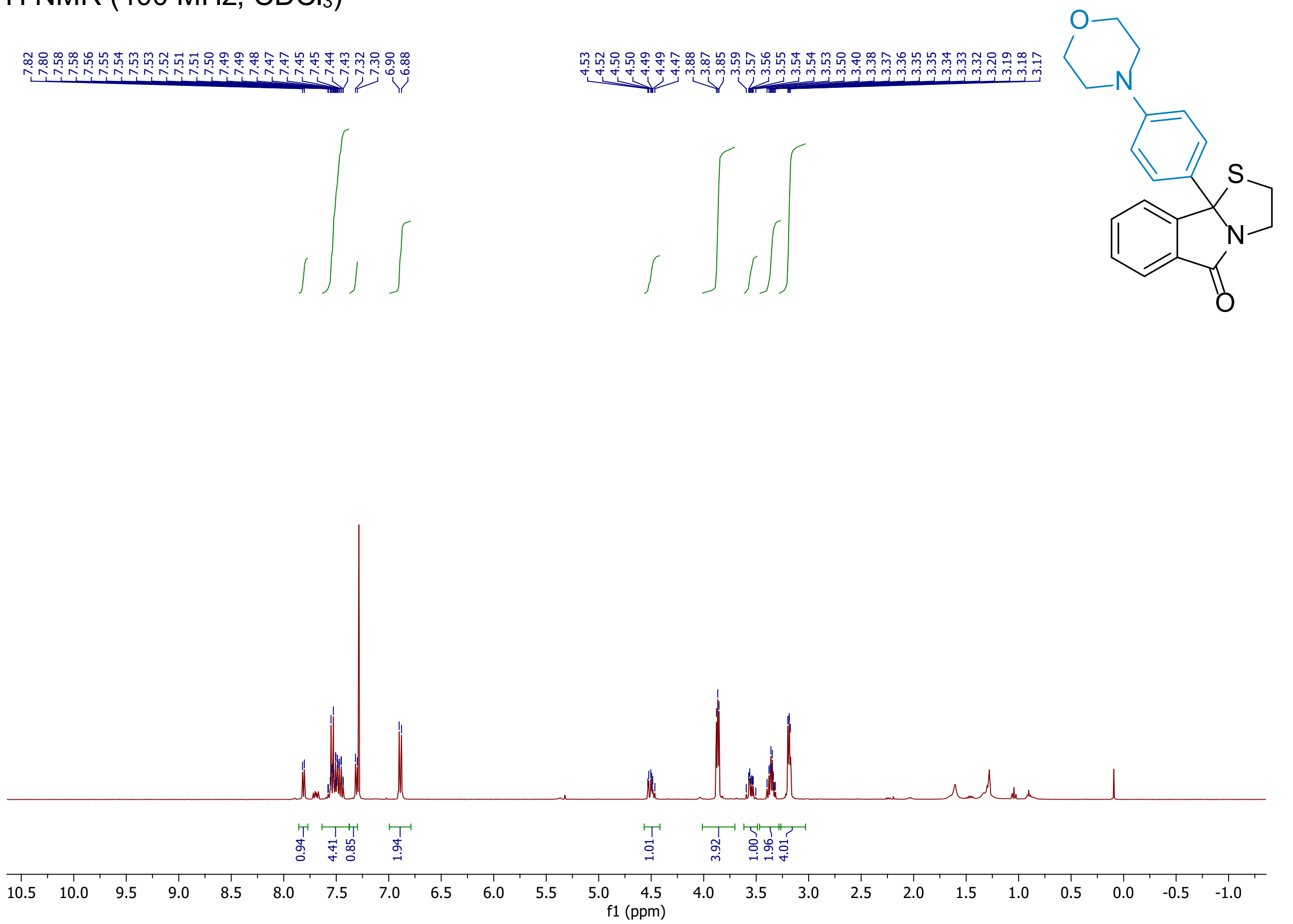

^{13}C NMR (101 MHz, $CDCl_3$)

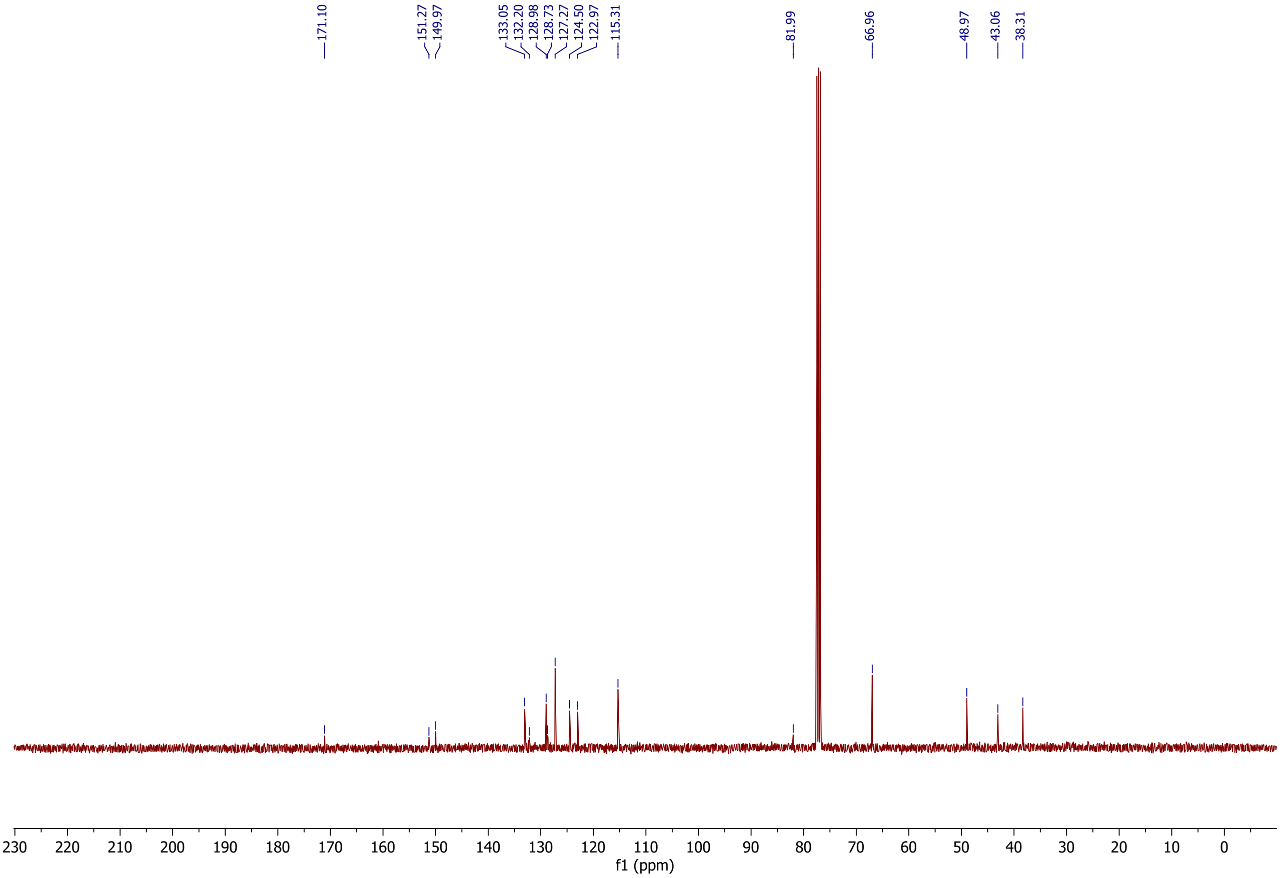

Figure S. 20. 2-[2-(1H-indol-3-yl)ethyl]isoindoline-1,3-dione (3)

^{1}H NMR (400 MHz, $CDCl_3$)

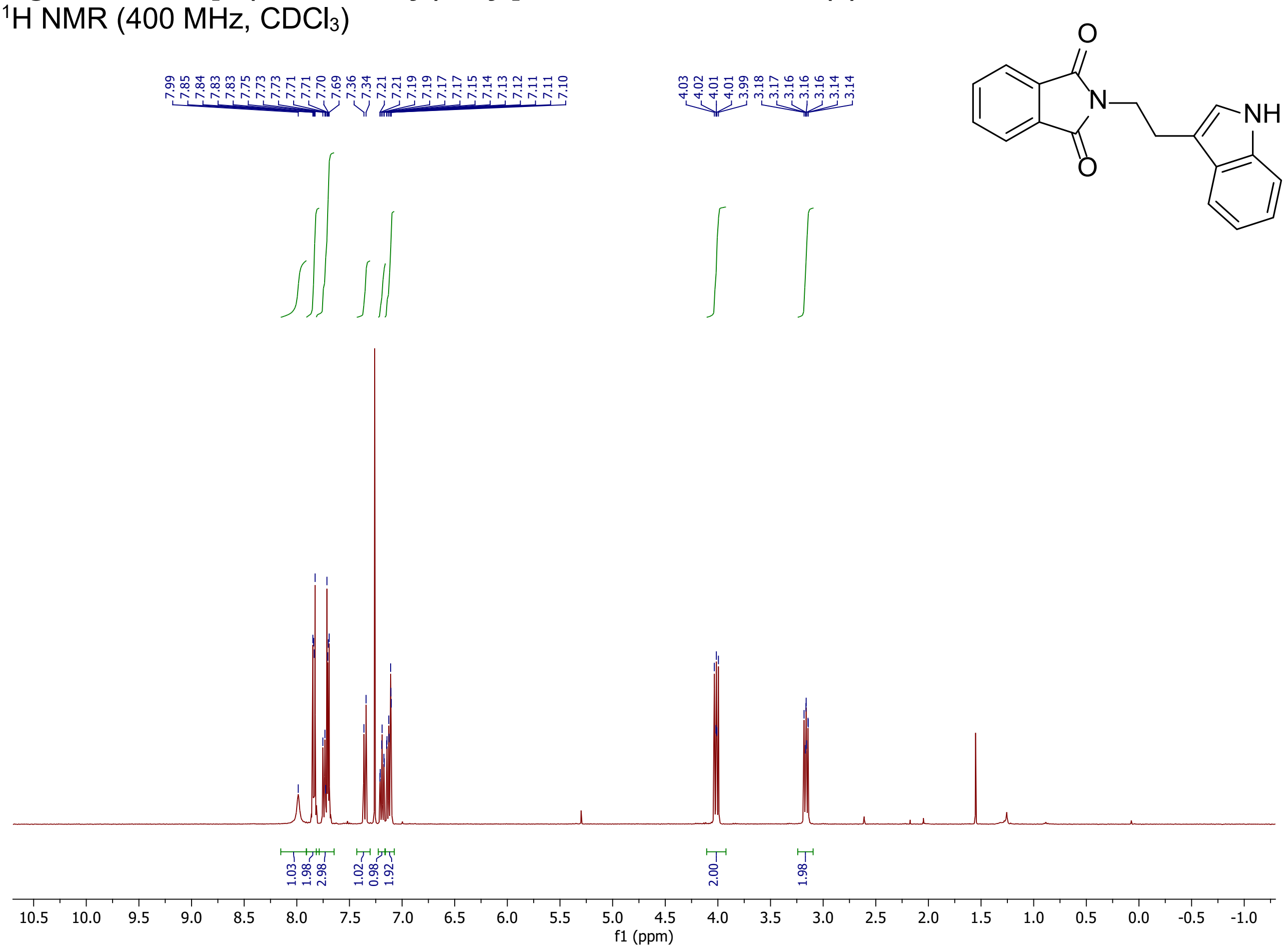

^{13}C NMR (101 MHz, $CDCl_3$)

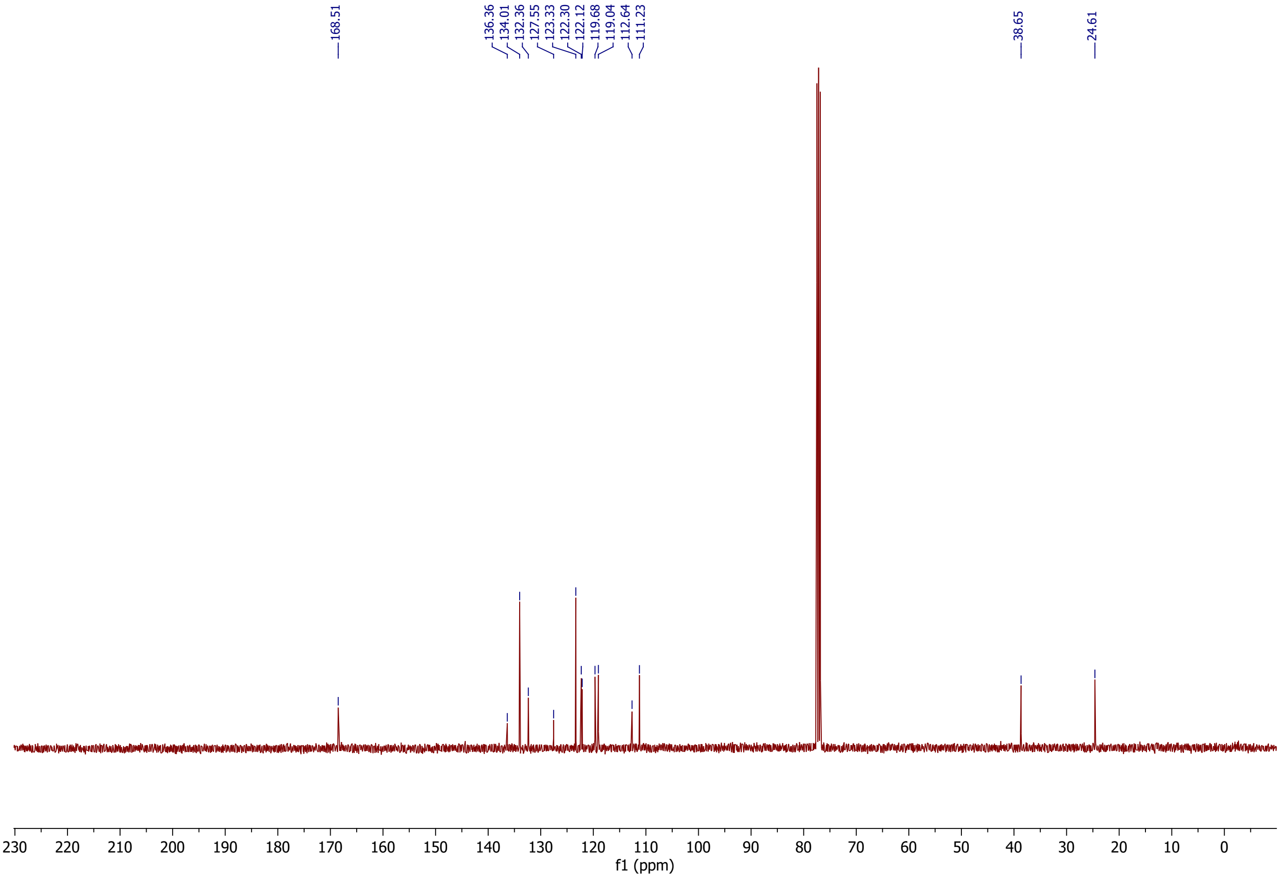

Figure S. 21. 3-hydroxy-2-[2-(1H-indol-3-yl)ethyl]-3-phenyl-isoindolin-1-one (3a)

^{1}H NMR (400 MHz, DMSO-d_6)

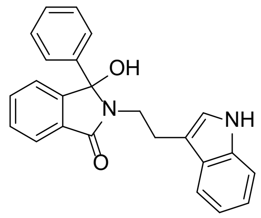

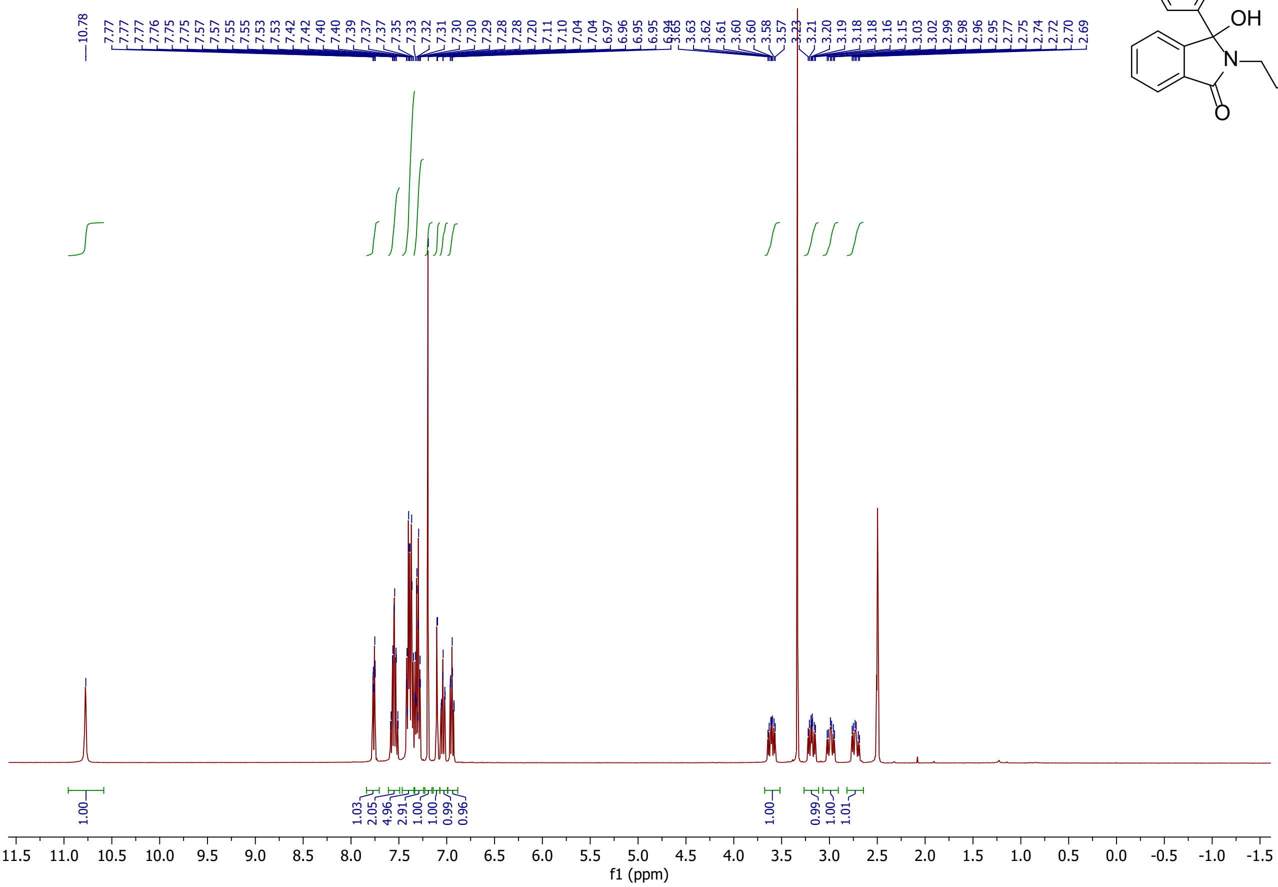

^{13}C NMR (101 MHz, $CDCl_3$)

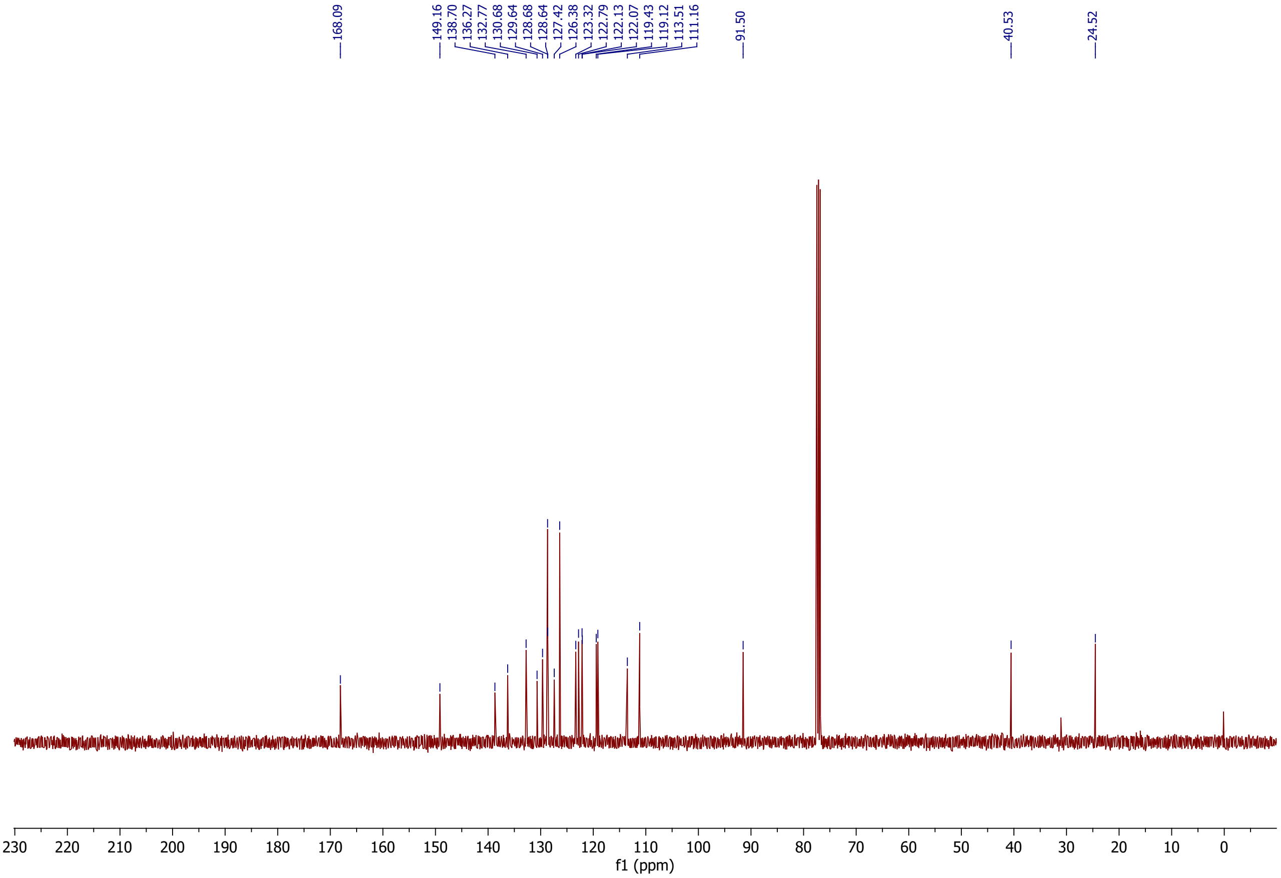

Figure S. 22. 3-hydroxy-2-[2-(1H-indol-3-yl)ethyl]-3-[4-(trifluoromethyl)phenyl]isoindolin-1-one (3b)

^{1}H NMR (400 MHz, DMSO-d$_6$)

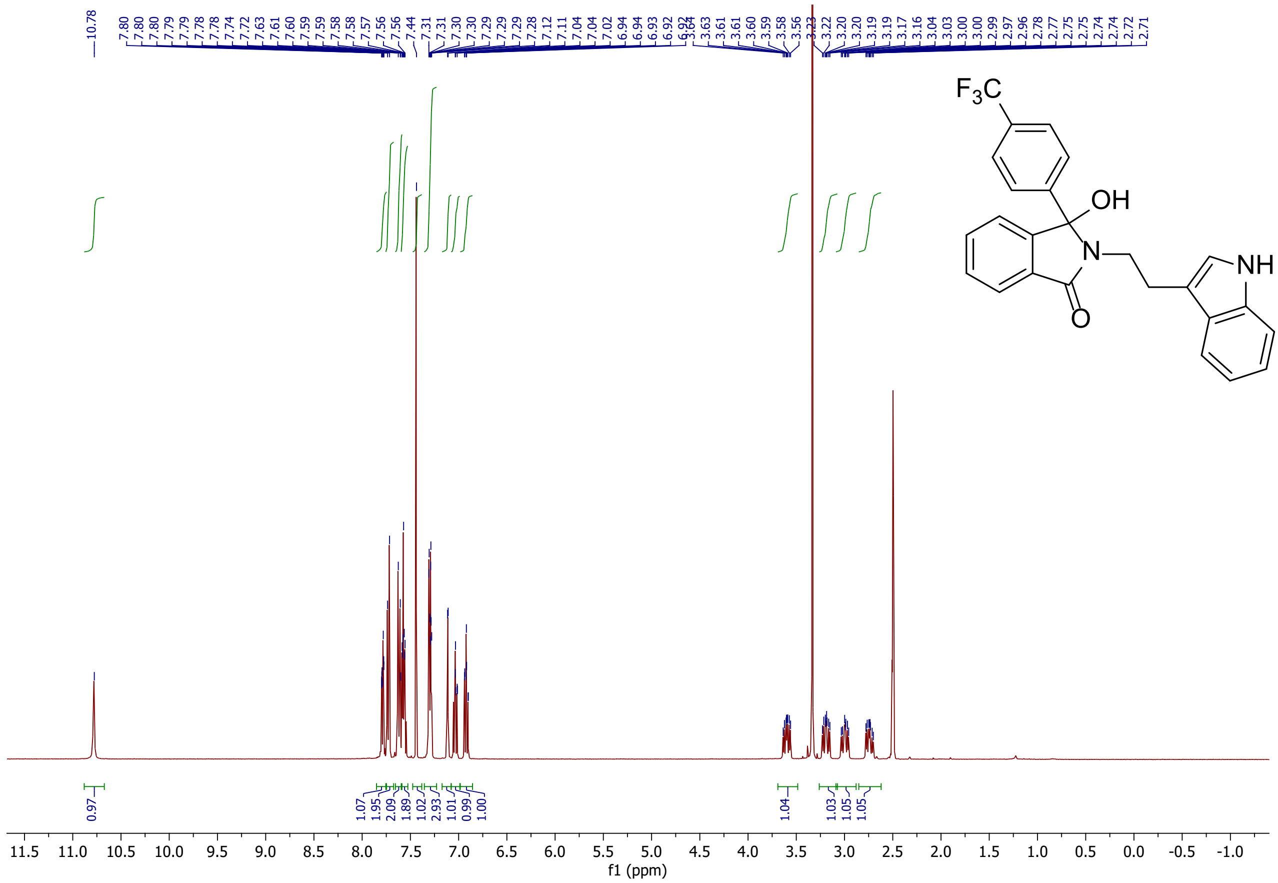

^{13}C NMR (101 MHz, DMSO-d$_6$)

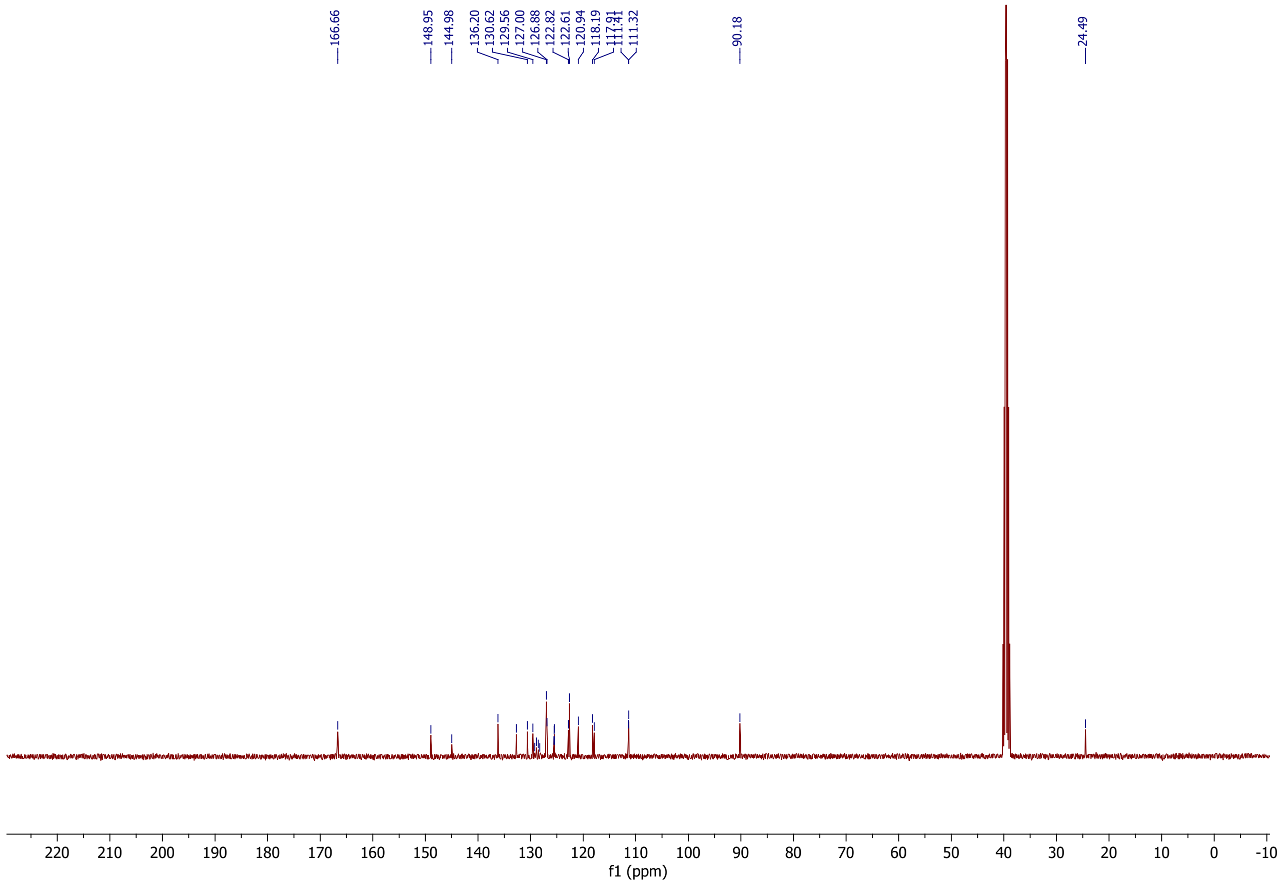

Figure S. 23. 3-(4-bromophenyl)-3-hydroxy-2-[2-(1H-indol-3-yl)ethyl]isoindolin-1-one (3c)
^{1}H NMR (400 MHz, DMSO-d_6)

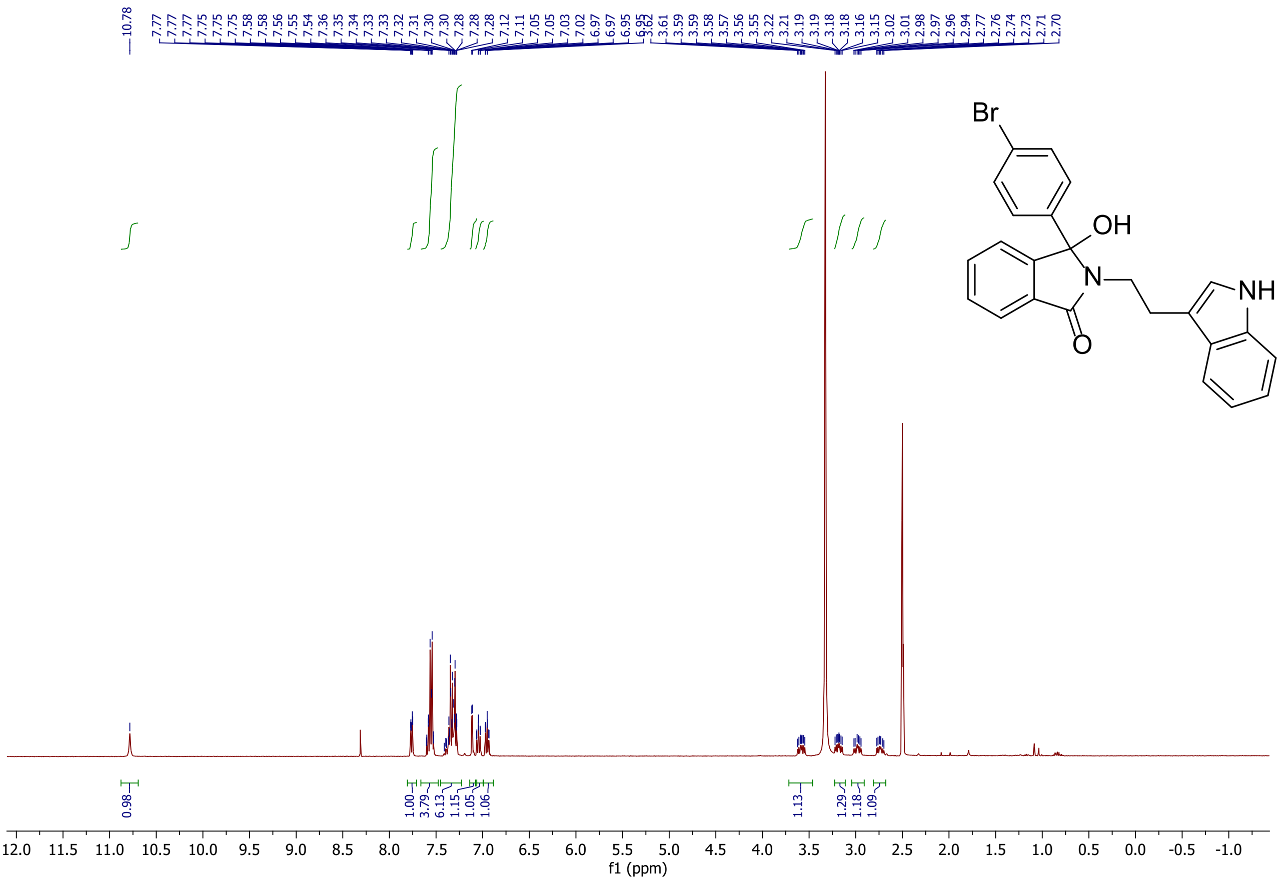

^{13}C NMR (101 MHz, DMSO-d_6)

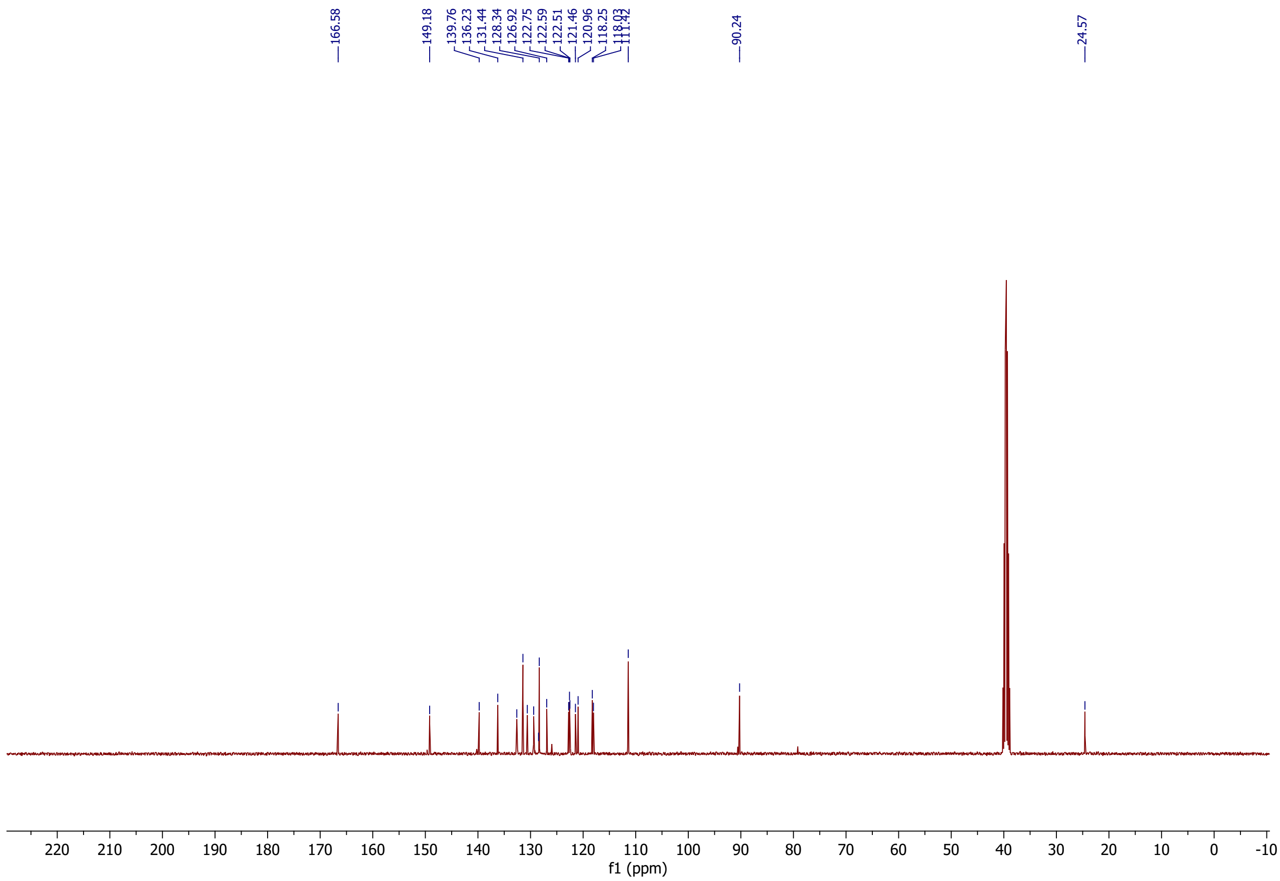

Figure S. 24. 3-[3,5-bis(trifluoromethyl)phenyl]-3-hydroxy-2-[2-(1H-indol-3-yl)ethyl]isoindolin-1-one (3d)

^{1}H NMR (400 MHz, $CDCl_3$)

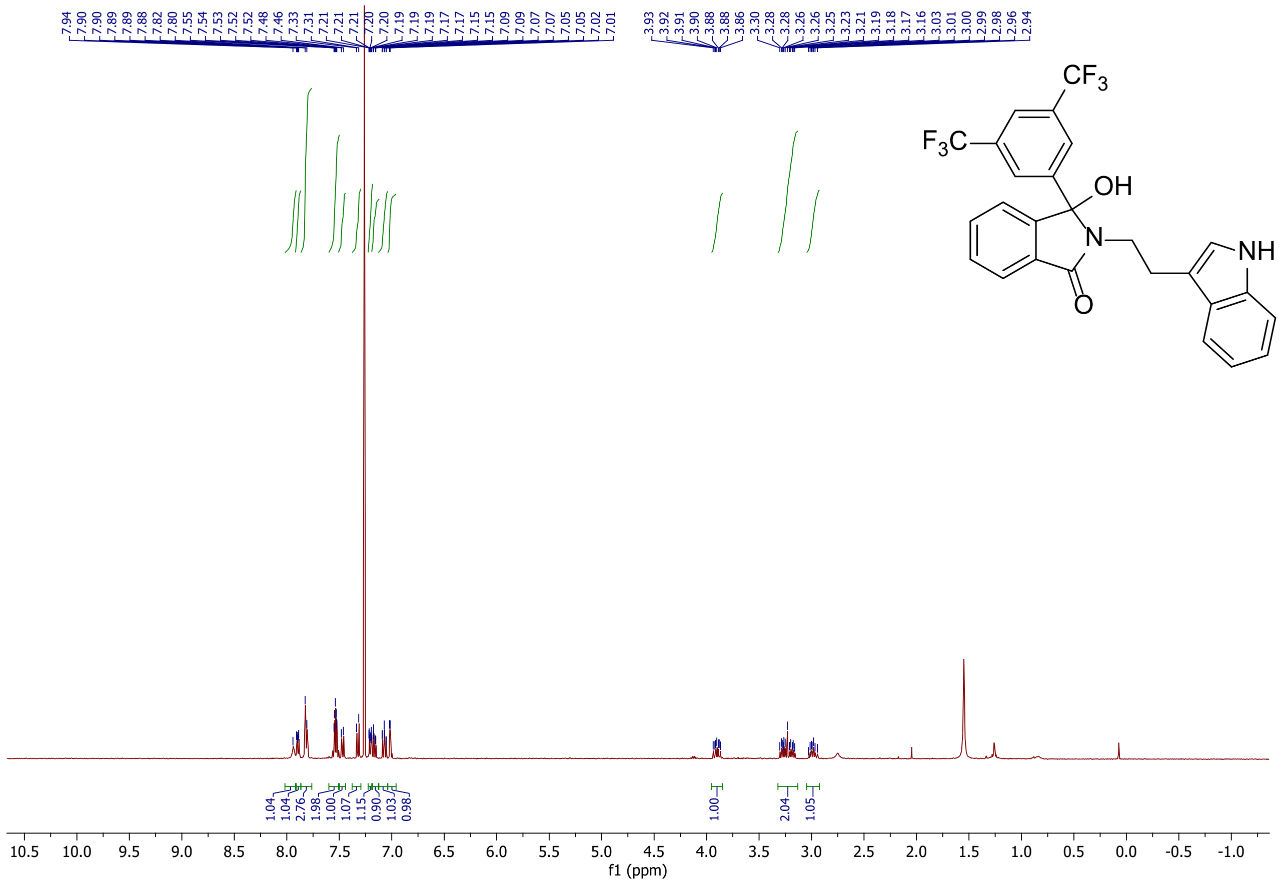

^{13}C NMR (101 MHz, DMSO-d_6)

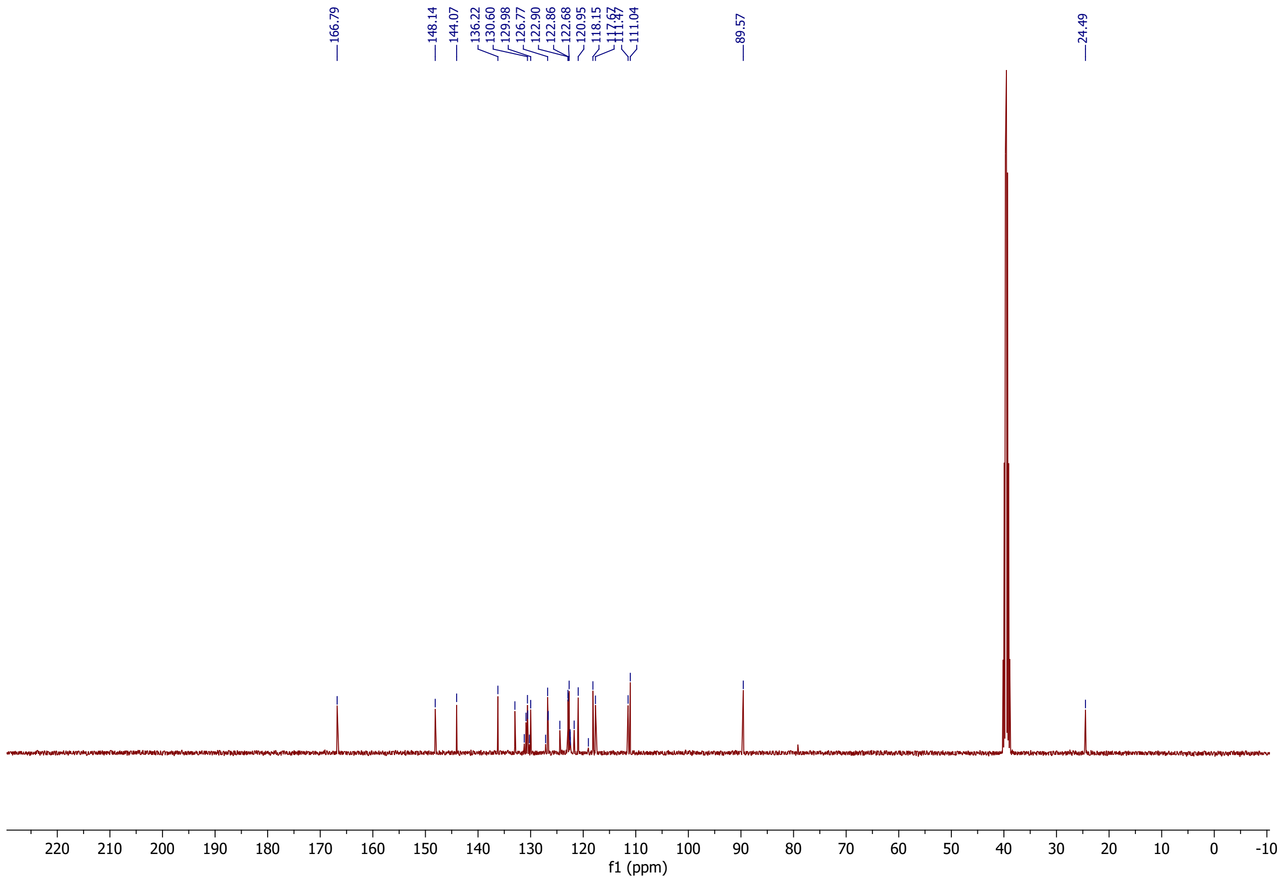

Figure S. 25. 3-hydroxy-2-[2-(1H-indol-3-yl)ethyl]-3-(4-methoxyphenyl)isoindolin-1-one (3e)
^{1}H NMR (400 MHz, DMSO-d$_6$)

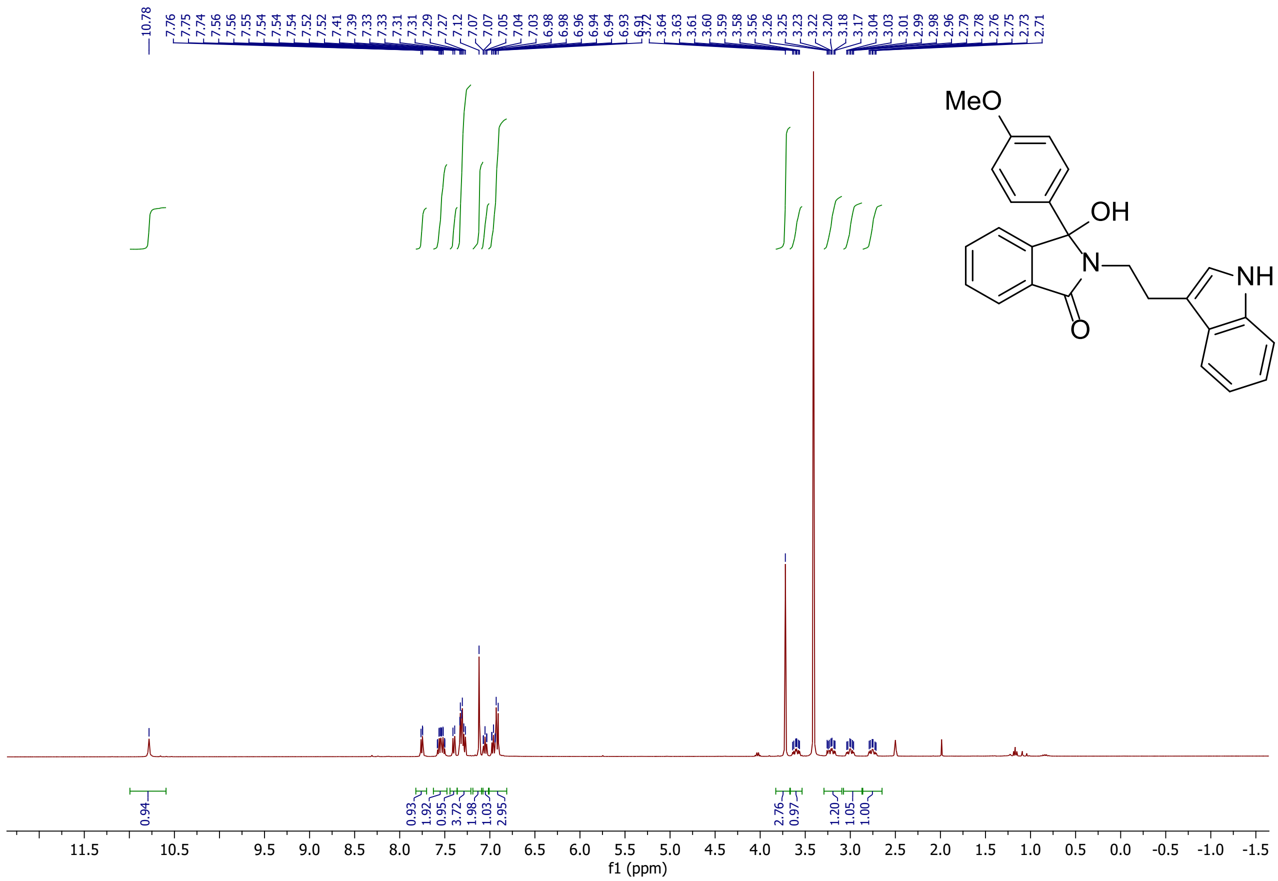

^{13}C NMR (101 MHz, DMSO-d$_6$)

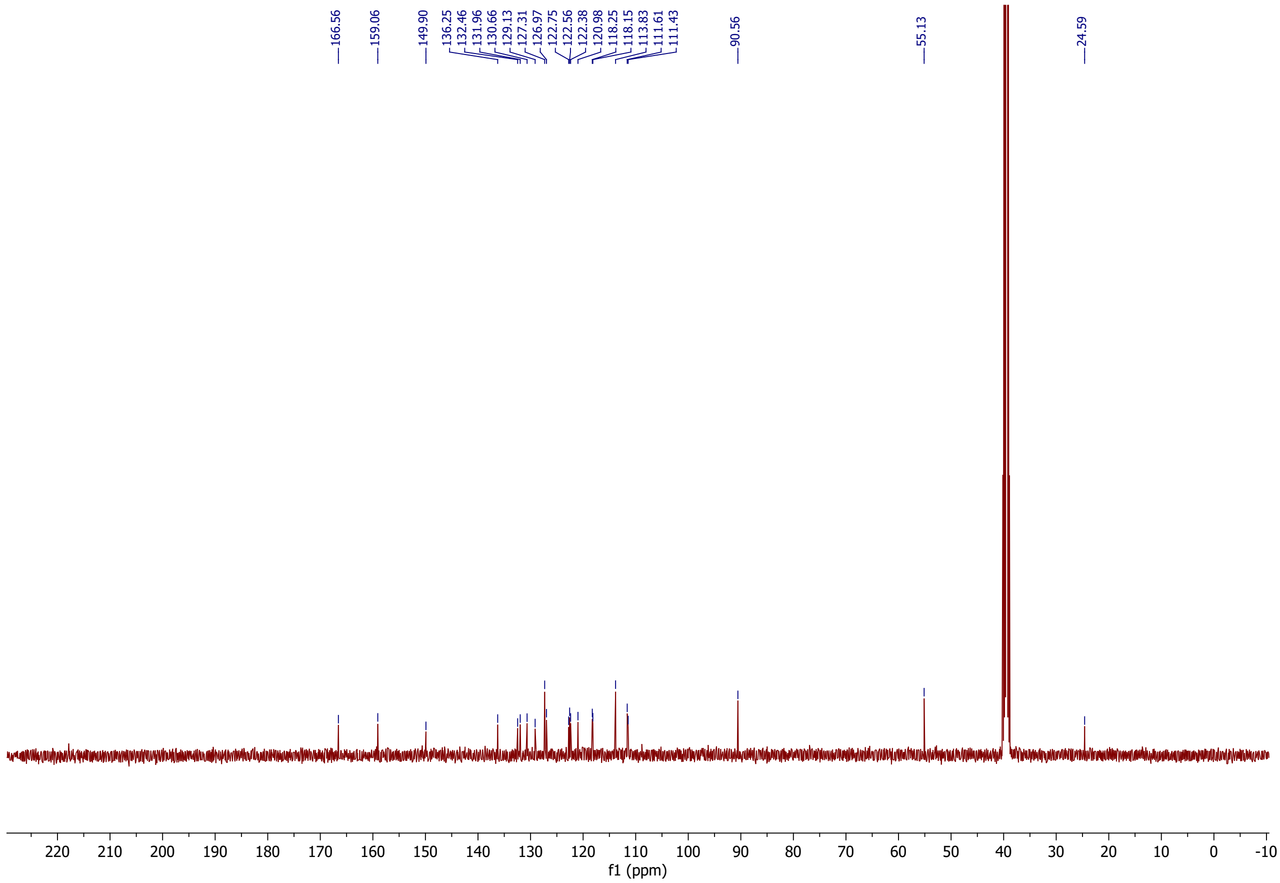

Figure S. 26. 3-(2,4-dimethoxyphenyl)-3-hydroxy-2-[2-(1H-indol-3-yl)ethyl]isoindolin-1-one (3f)

^{1}H NMR (400 MHz, DMSO-d_6)

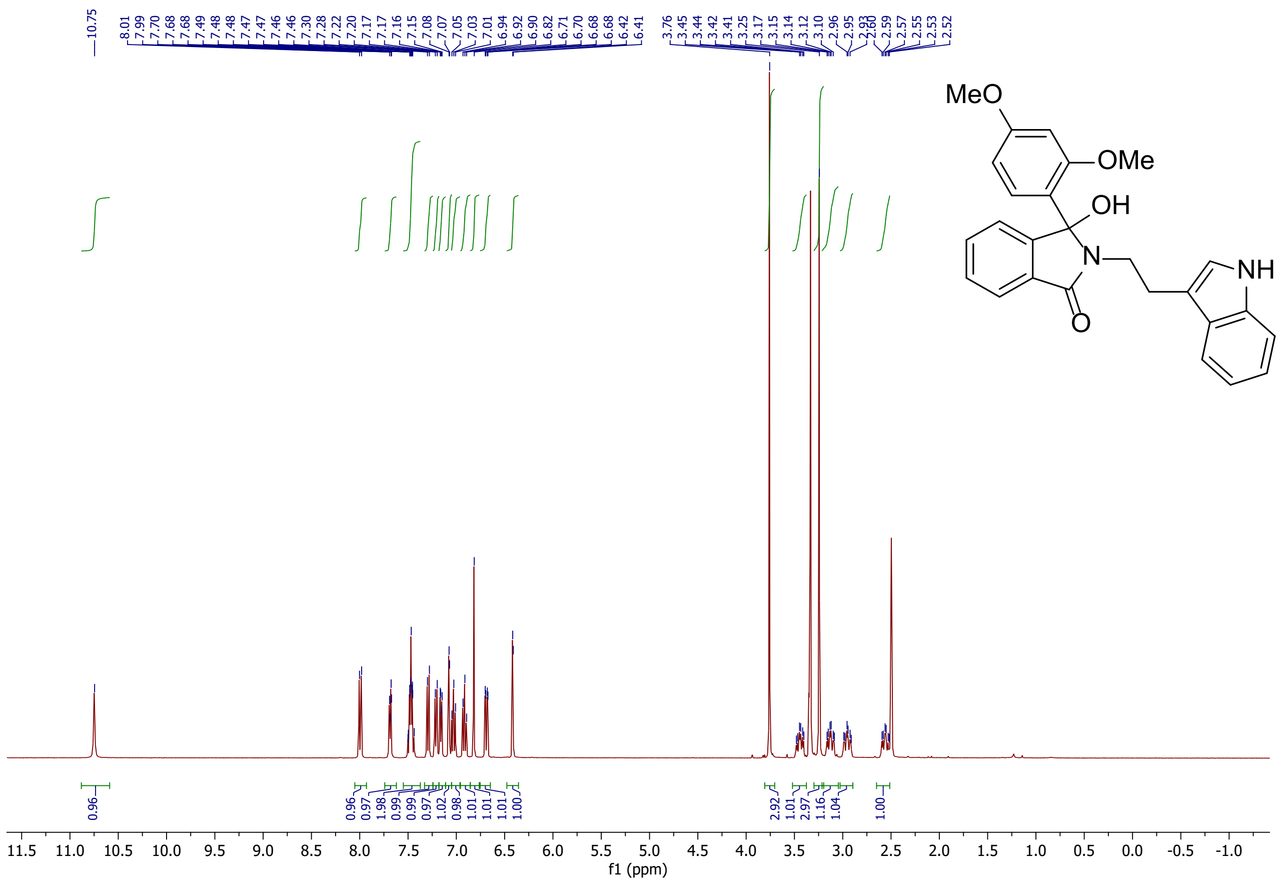

^{13}C NMR (101 MHz, DMSO-d_6)

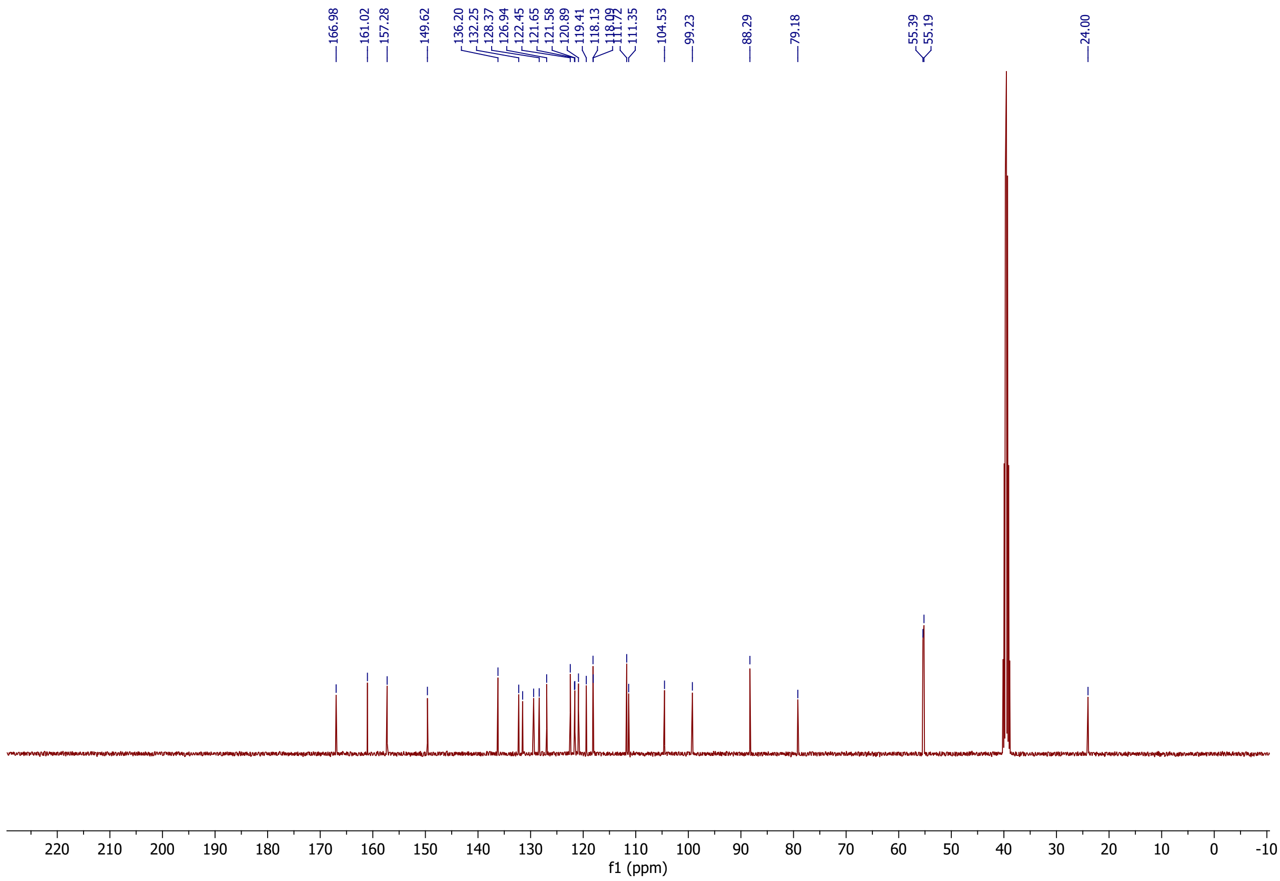

Figure S. 27. 3-(2-furyl)-3-hydroxy-2-[2-(1H-indol-3-yl)ethyl]isoindolin-1-one (3g)

^{1}H NMR (400 MHz, $CDCl_3$)

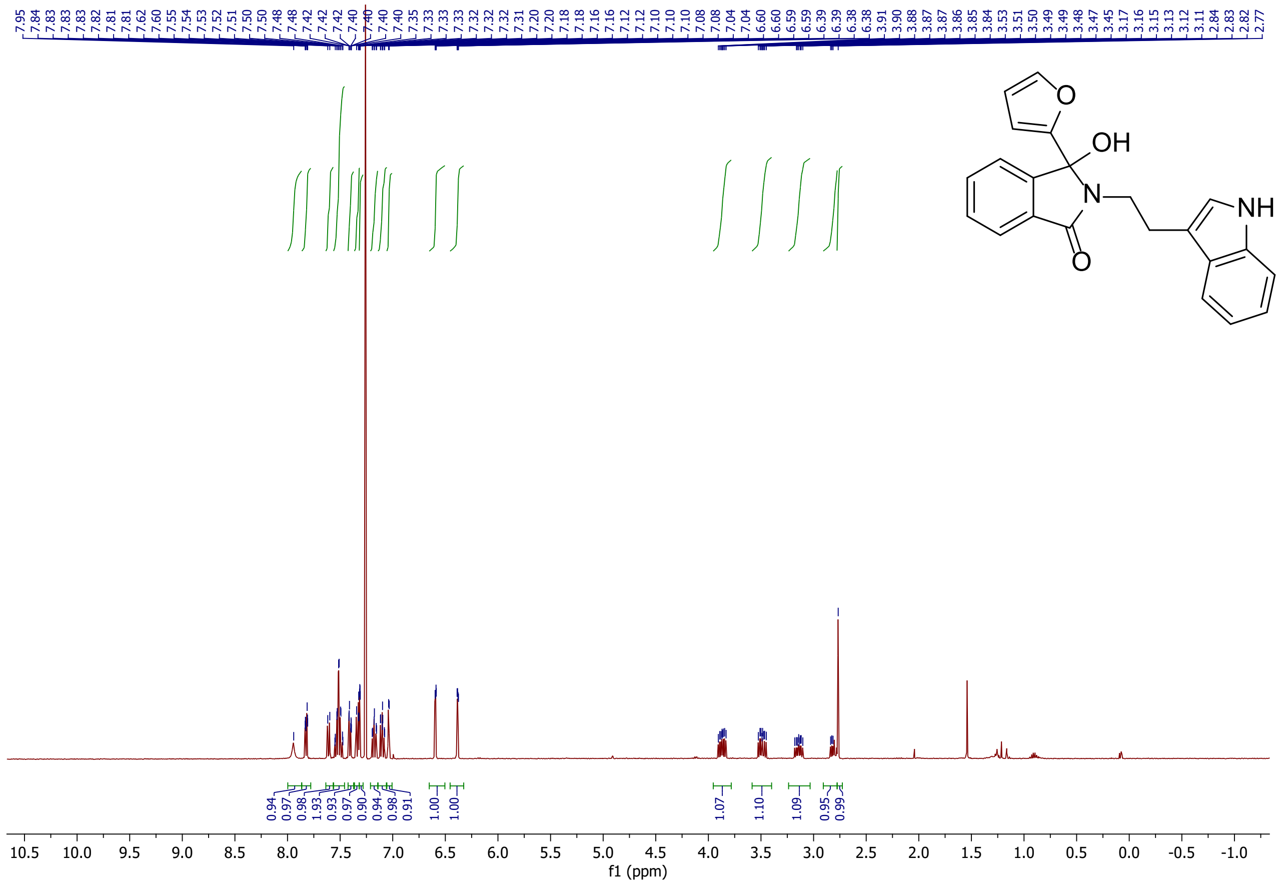

^{13}C NMR (101 MHz, $CDCl_3$)

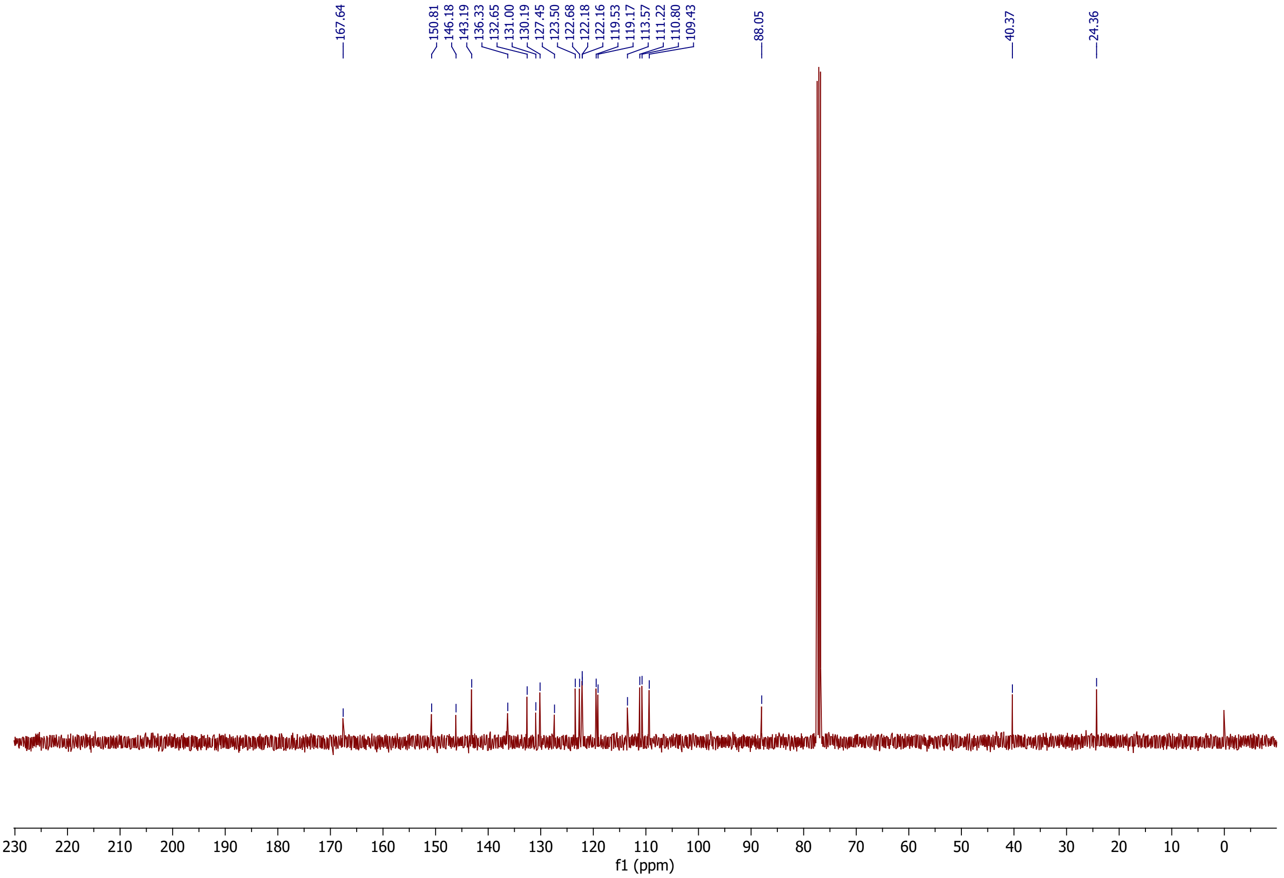

Figure S. 28. 3-hydroxy-2-[2-(1H-indol-3-yl)ethyl]-3-(6-methoxy-2-pyridyl)isoindolin-1-one (3h)

^{1}H NMR (400 MHz, $CDCl_3$)

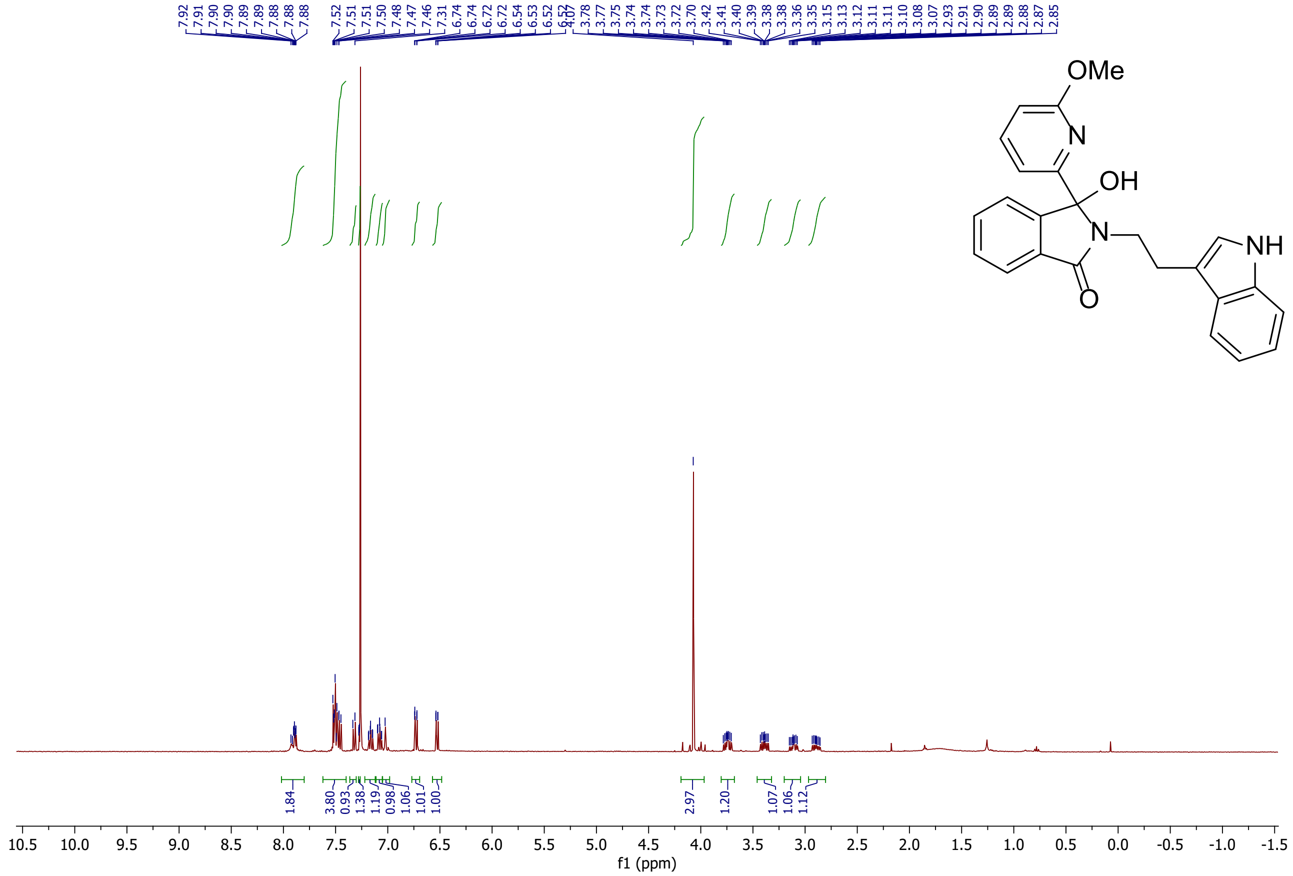

^{13}C NMR (101 MHz, $CDCl_3$)

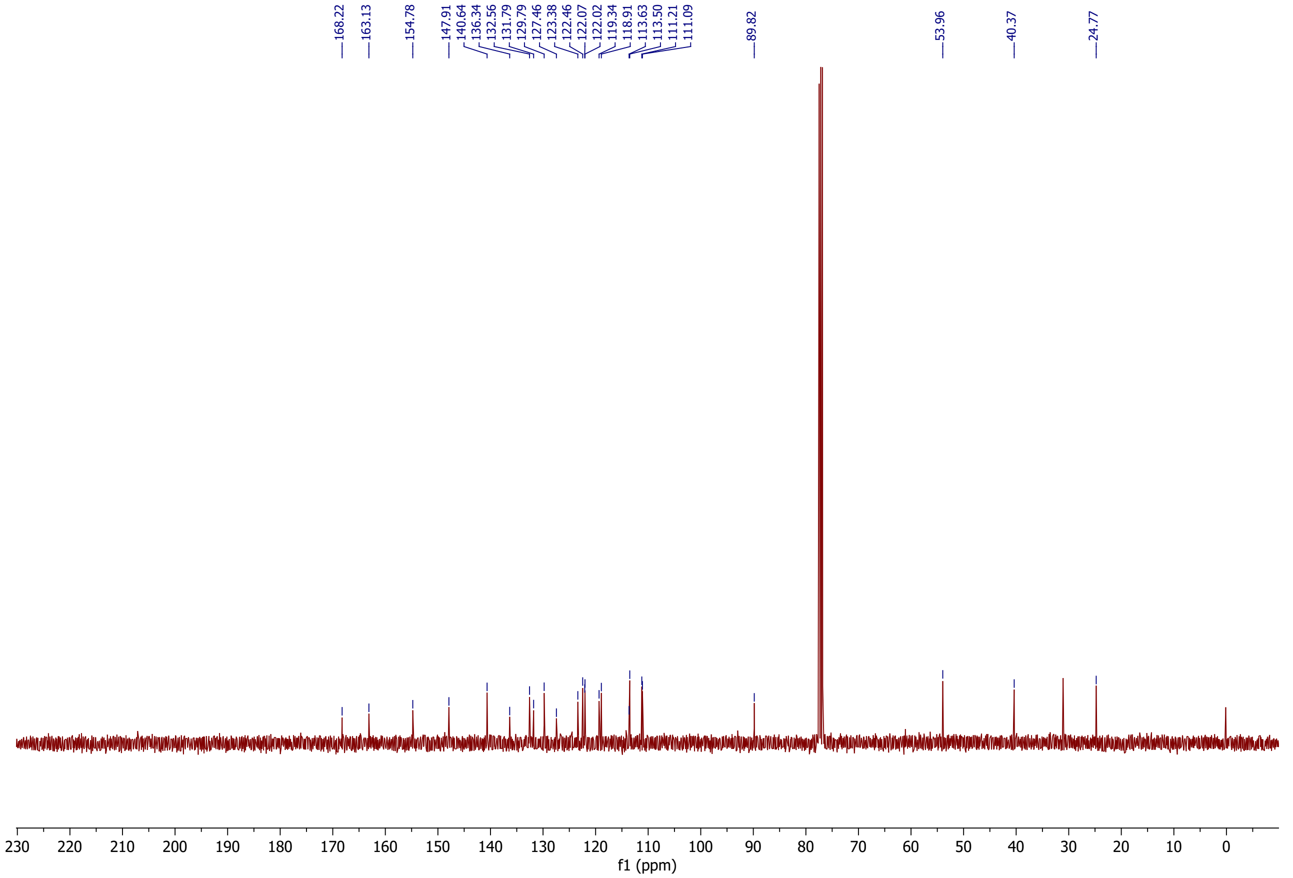

Figure S. 29. 3-(1,3-benzodioxol-5-yl)-3-hydroxy-2-[2-(1H-indol-3-yl)ethyl]isoindolin-1-one (3i)

^{1}H NMR (400 MHz, DMSO-d_6)

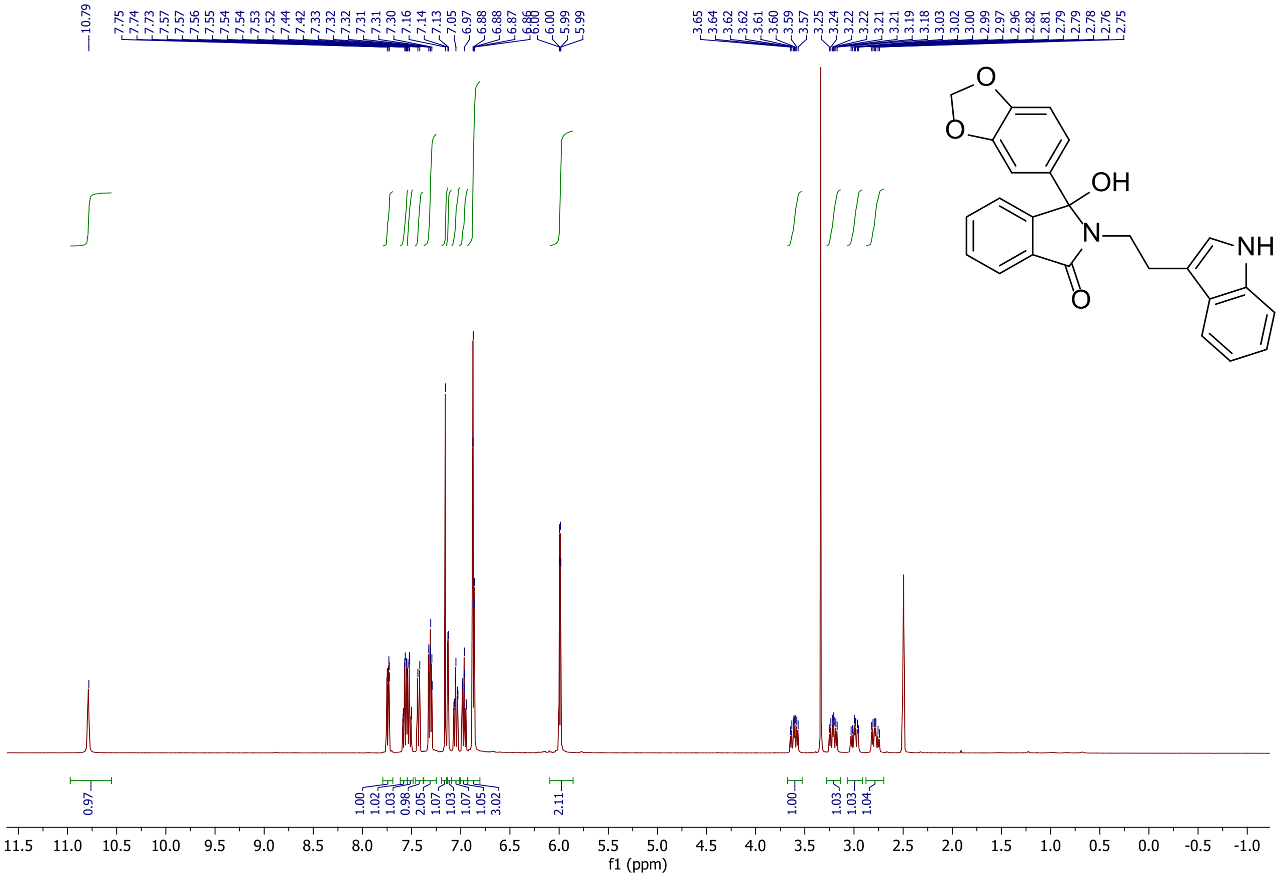

^{13}C NMR (101 MHz, DMSO-d_6)

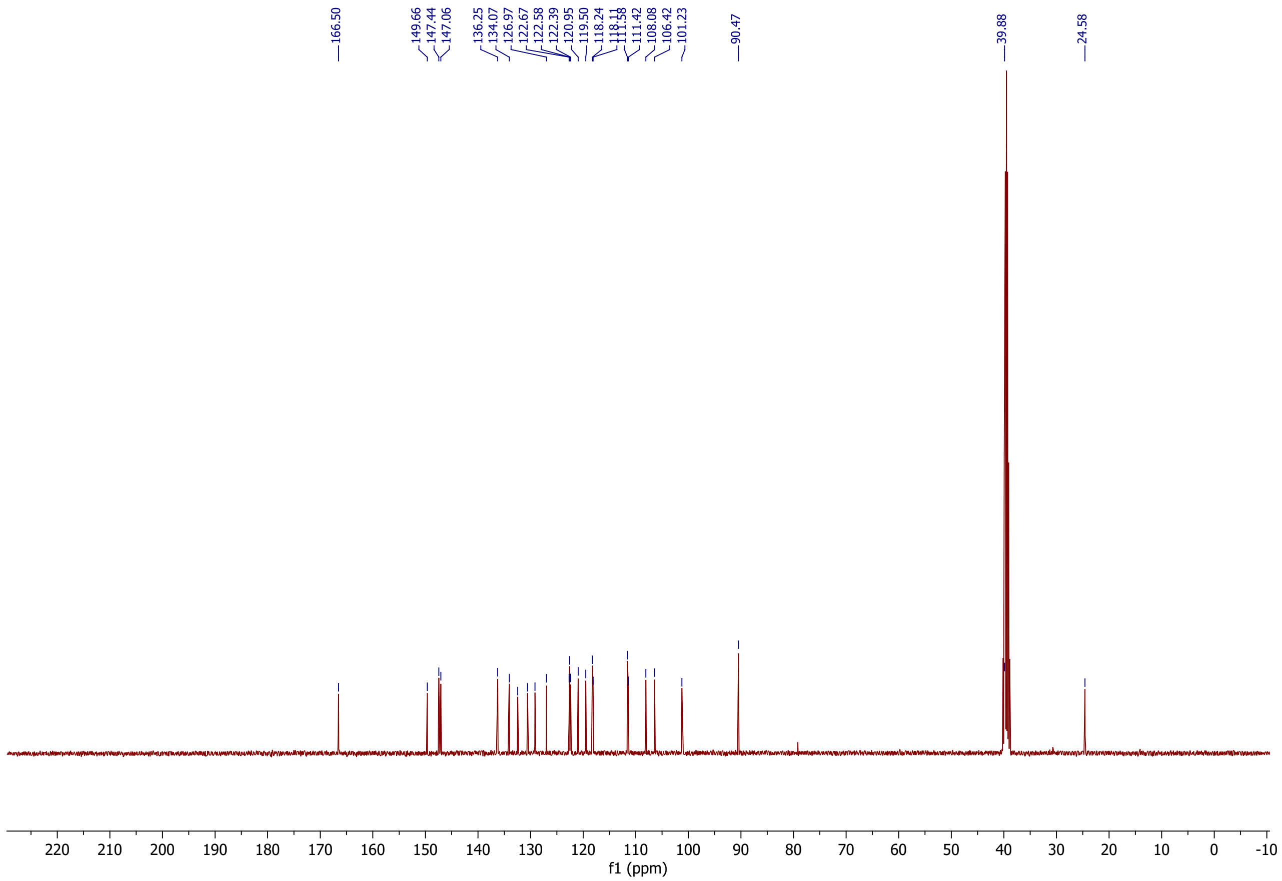

Figure S. 30. 2-phenyl-10,20-diazapentacyclo[11.7.0.02,10.03,8.014,19]icosa-1(13),3,5,7,14(19),15,17-heptaen-9-one (4a)

^{1}H NMR (400 MHz, $CDCl_3$)

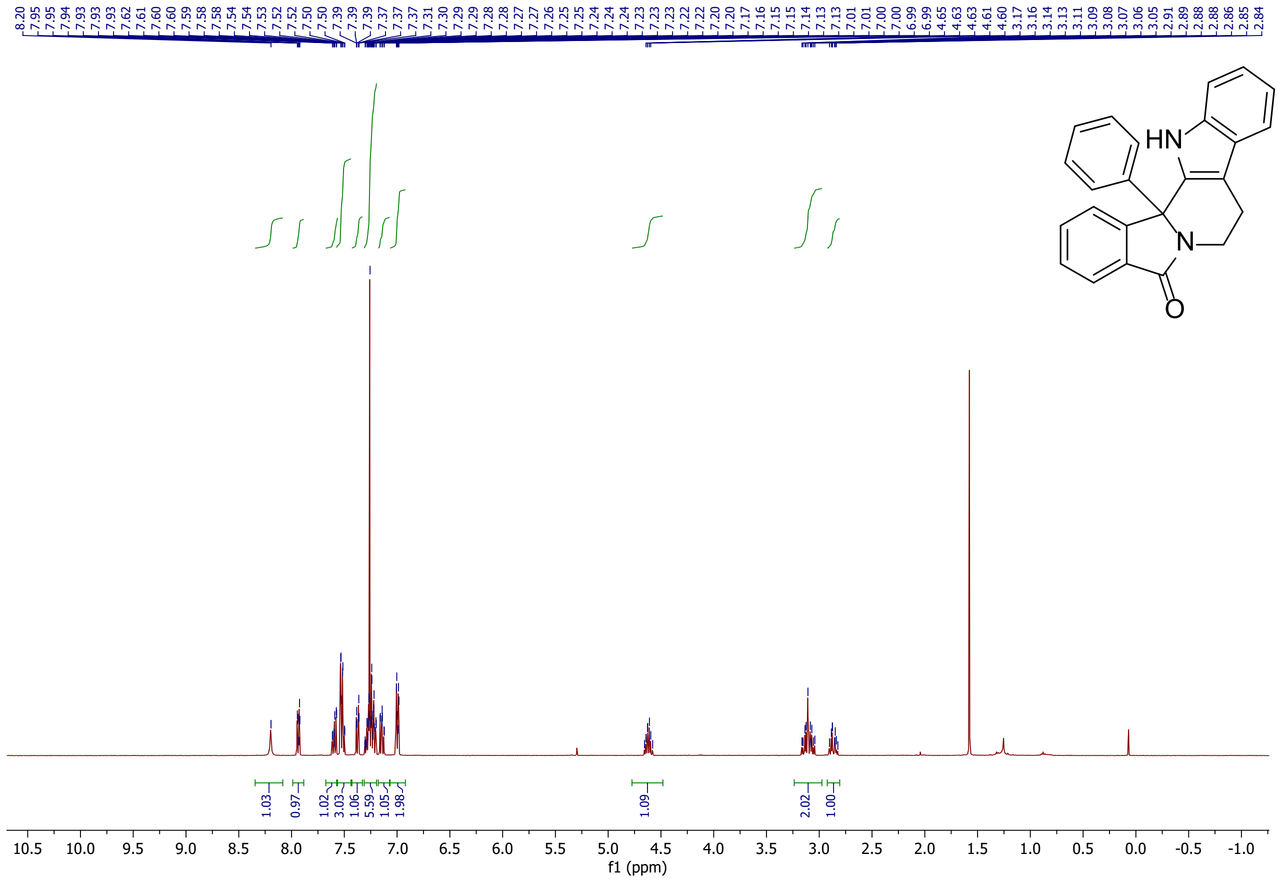

^{13}C NMR (101 MHz, $CDCl_3$)

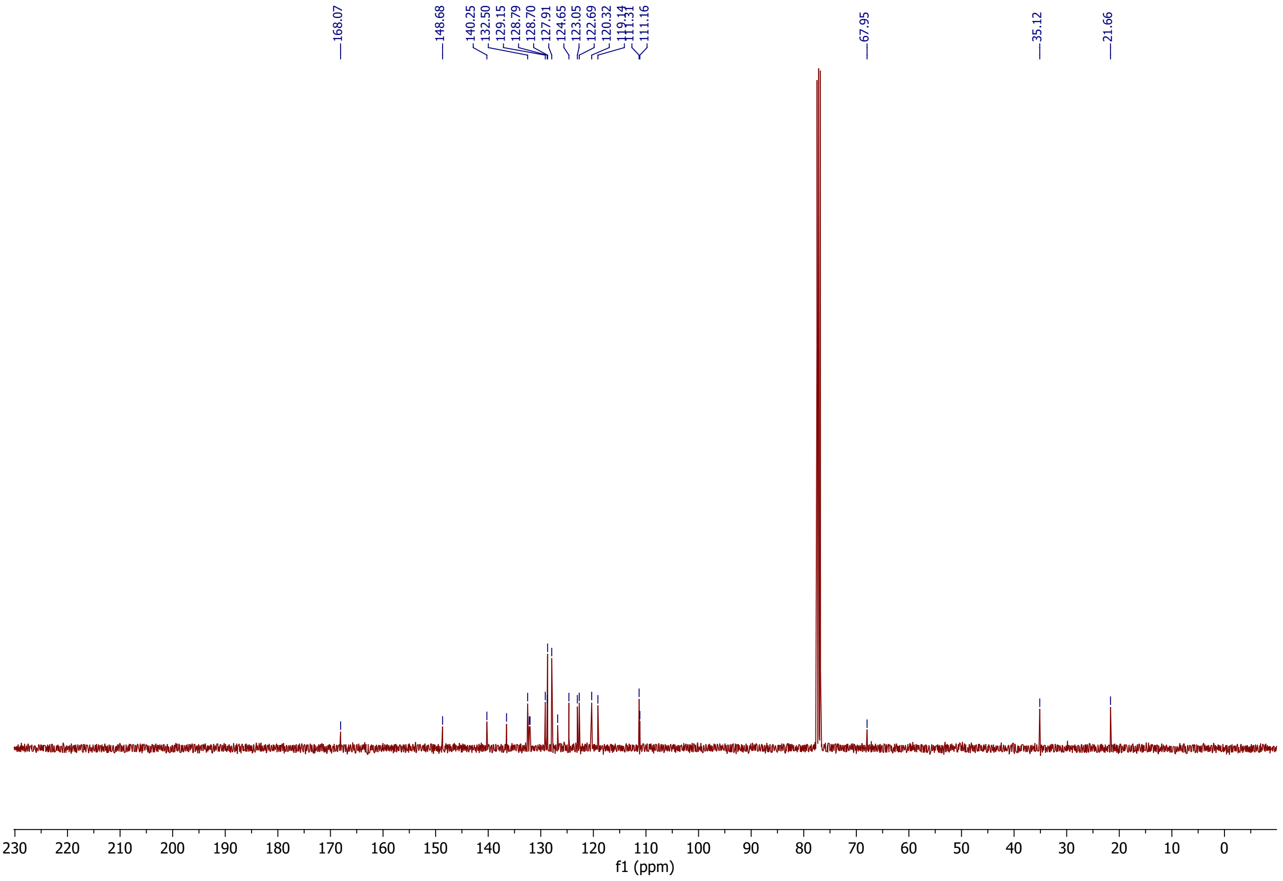

Figure S. 31. 2-[4-(trifluoromethyl)phenyl]-10,20-diazapentacyclo[11.7.0.02,10.03,8.014,19]icosa-1(13),3,5,7,14(19),15,17-heptaen-9-one (4b)

^{1}H NMR (400 MHz, $CDCl_3$)

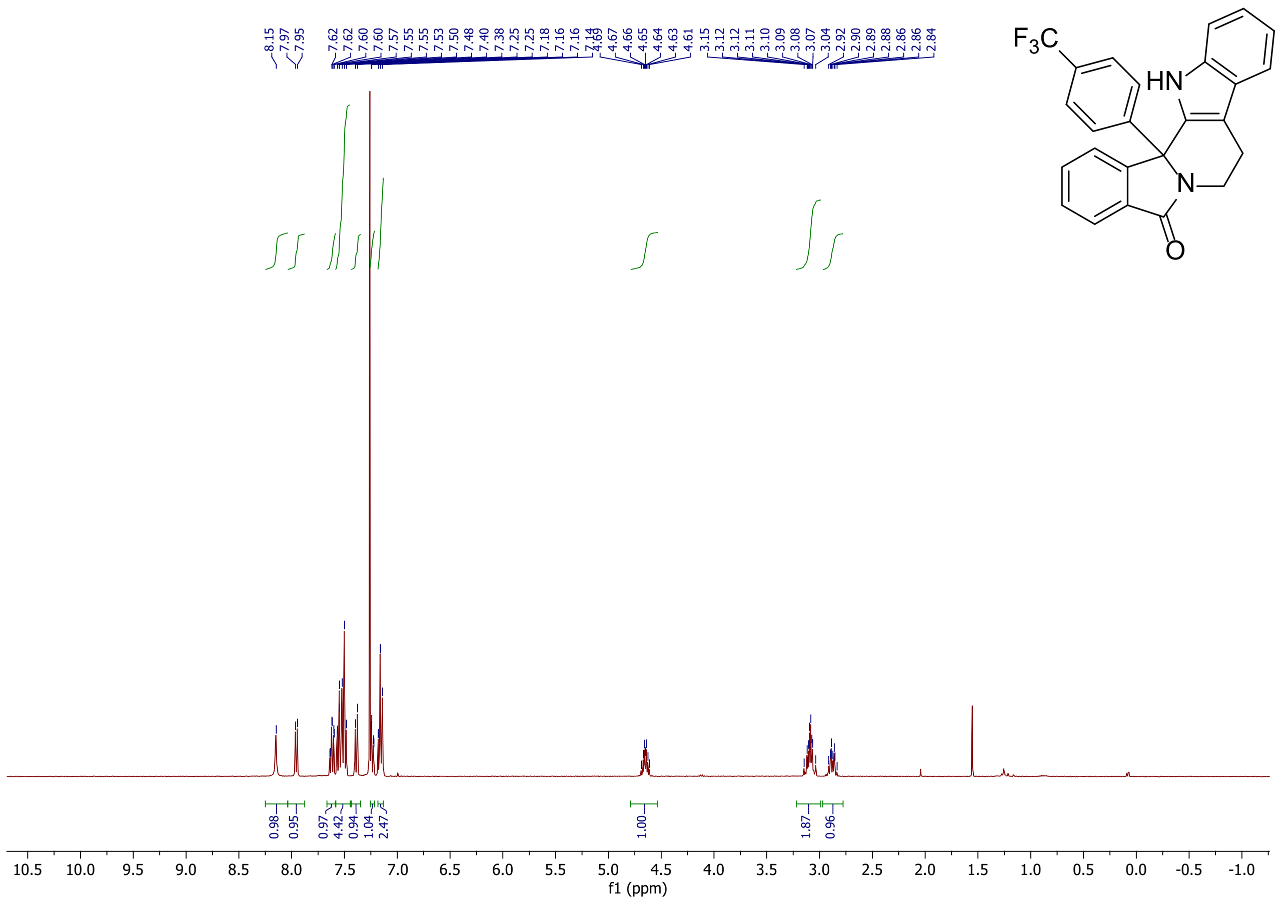

^{13}C NMR (101 MHz, DMSO-d_6)

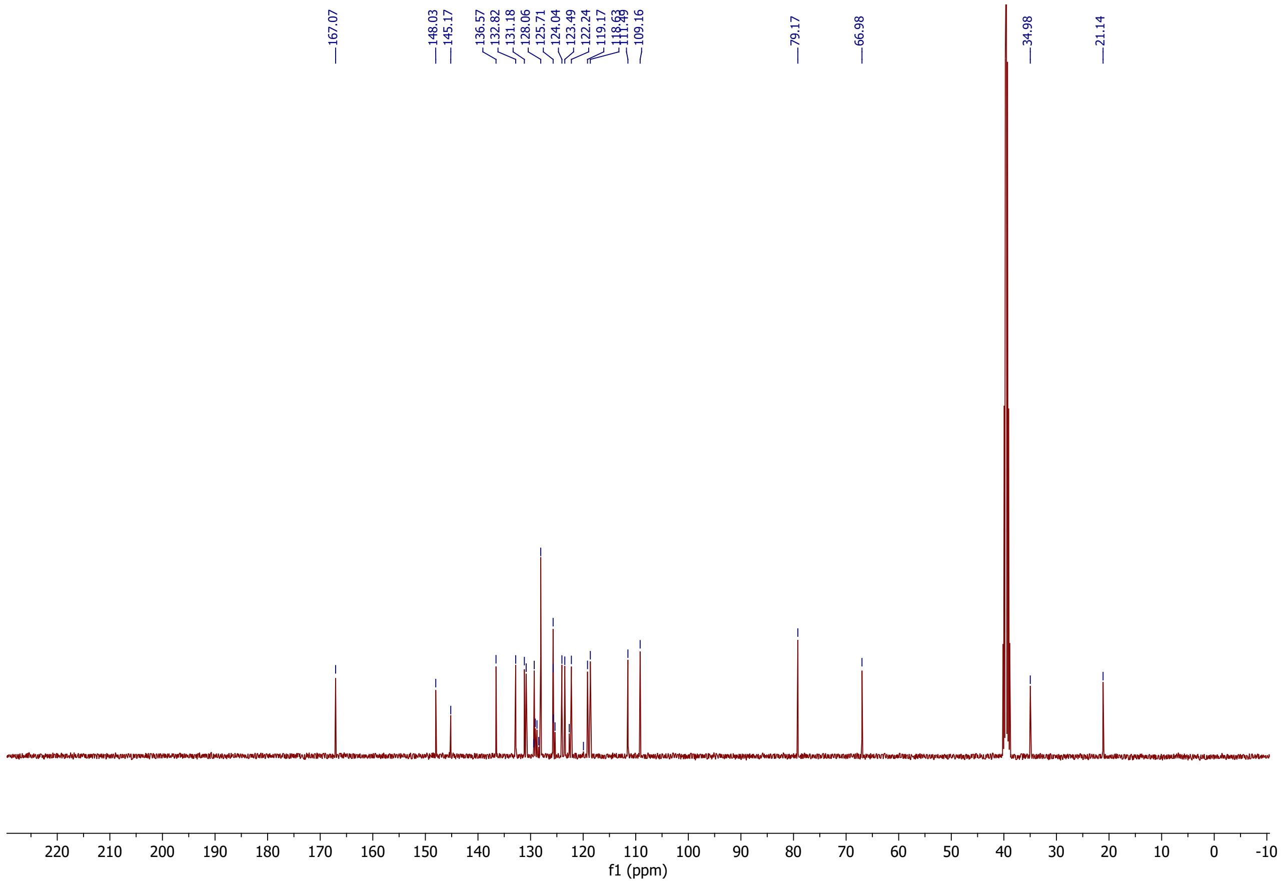

Figure S. 32. 2-(4-bromophenyl)-10,20-diazapentacyclo[11.7.0.02,10.03,8.014,19]icosa-1(13),3,5,7,14(19),15,17-heptaen-9-one (4c)

^{1}H NMR (400 MHz, $CDCl_3$)

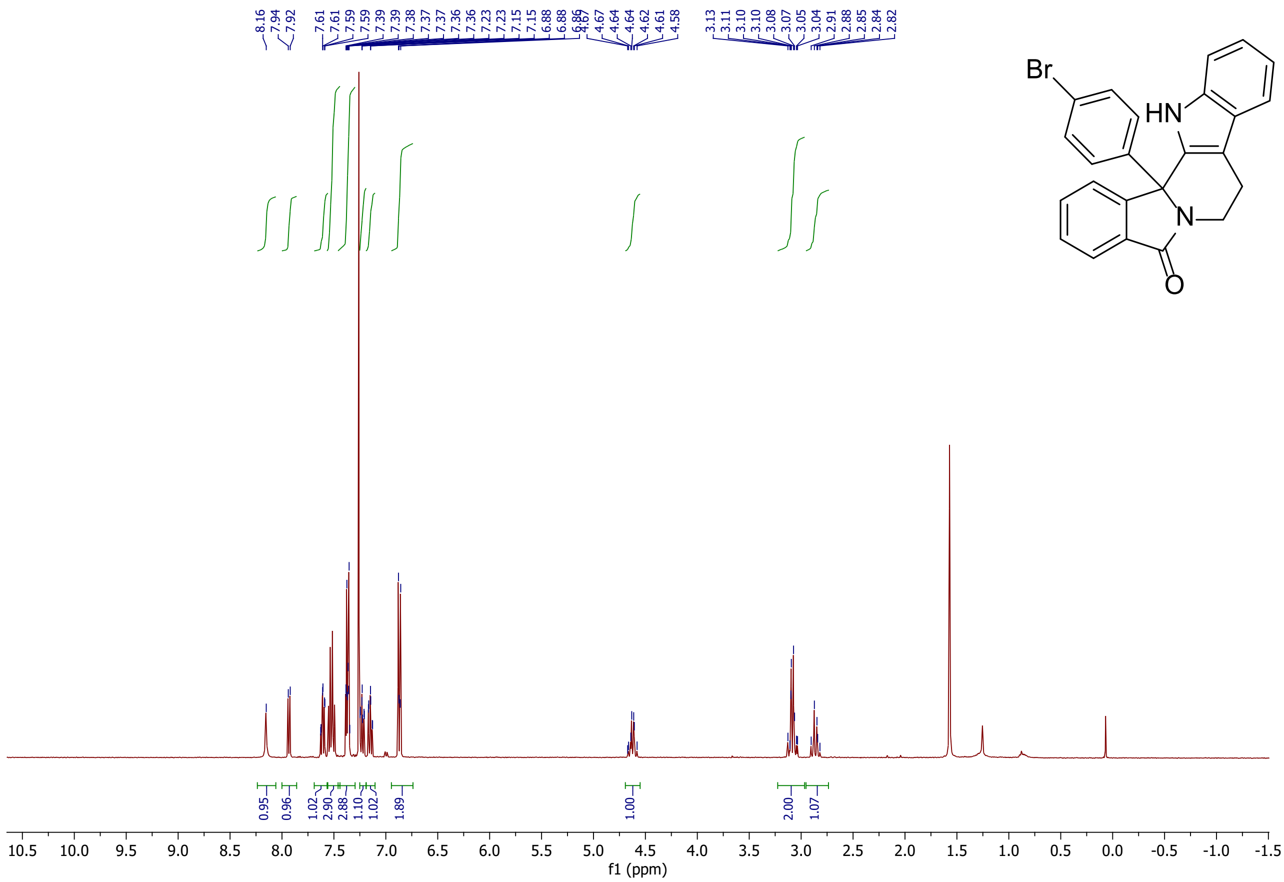

^{13}C NMR (101 MHz, $CDCl_3$)

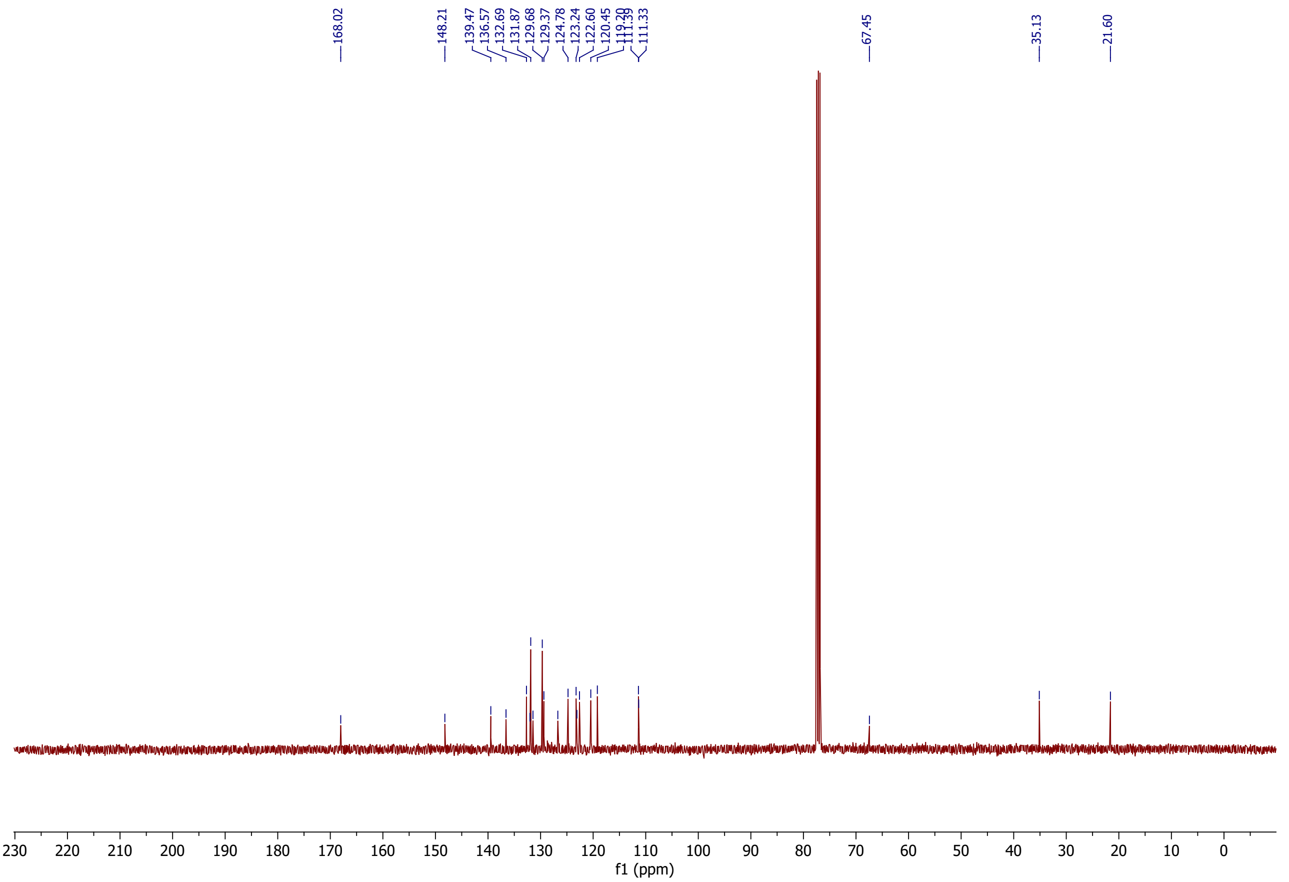

Figure S. 33. 2-[3,5-bis(trifluoromethyl)phenyl]-10,20-diazapentacyclo[11.7.0.02,10.03,8.014,19]icosa-1(13),3,5,7,14(19),15,17-heptaen-9-one (4d)

^{1}H NMR (400 MHz, DMSO-d$_6$)

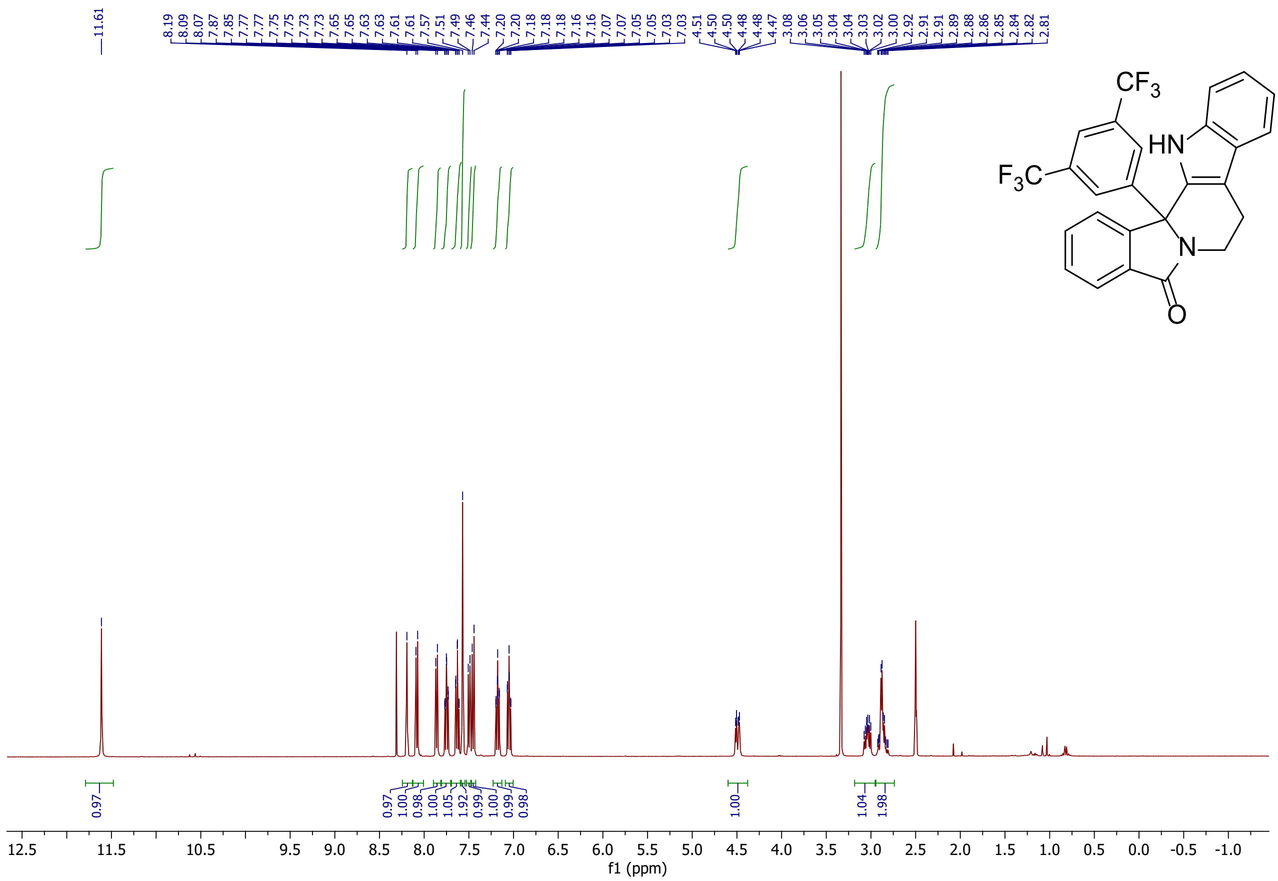

^{13}C NMR (101 MHz, DMSO-d$_6$)

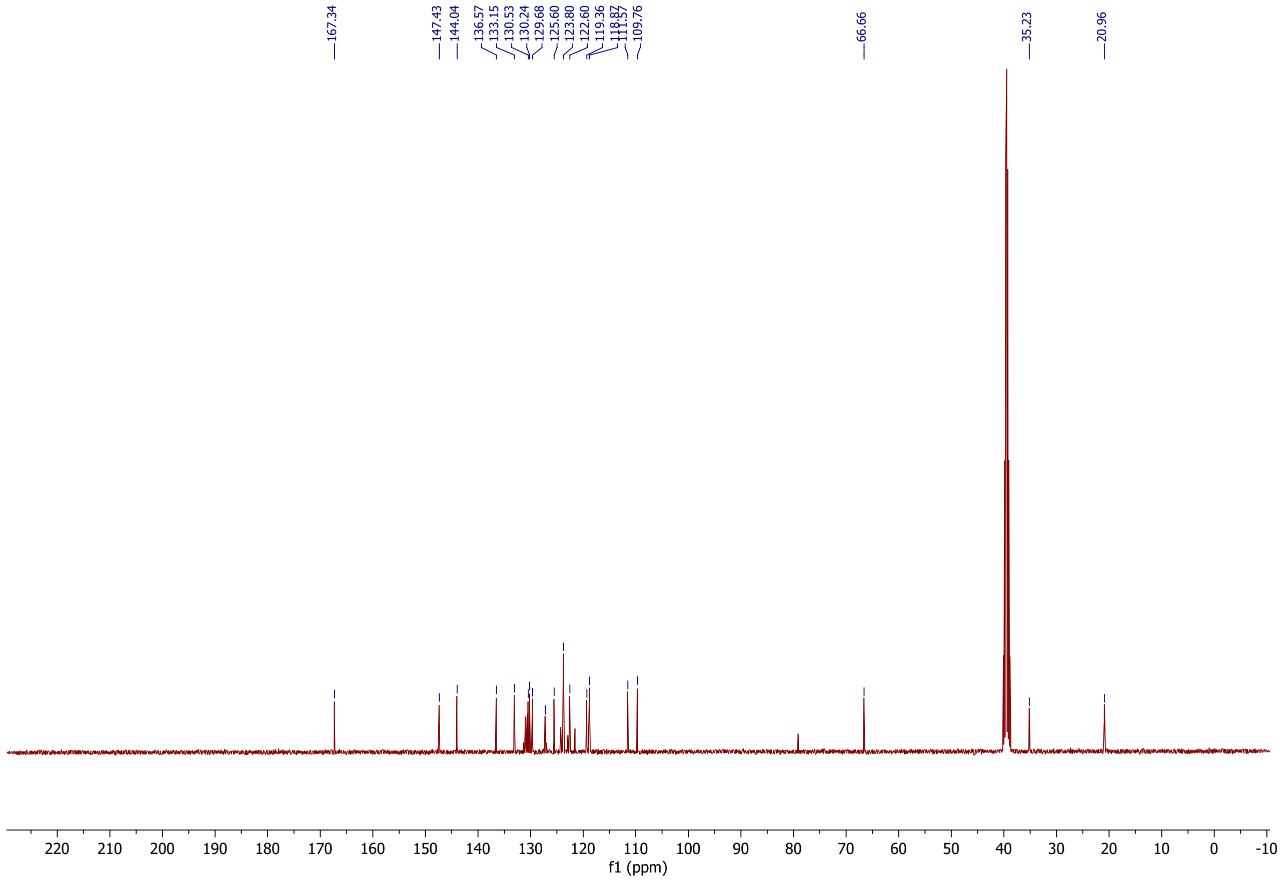

Figure S. 34. 2-(4-methoxyphenyl)-10,20-diazapentacyclo[11.7.0.02,10.03,8.014,19]icosa-1(13),3,5,7,14(19),15,17-heptaen-9-one (4e)

^{1}H NMR (400 MHz, $CDCl_3$)

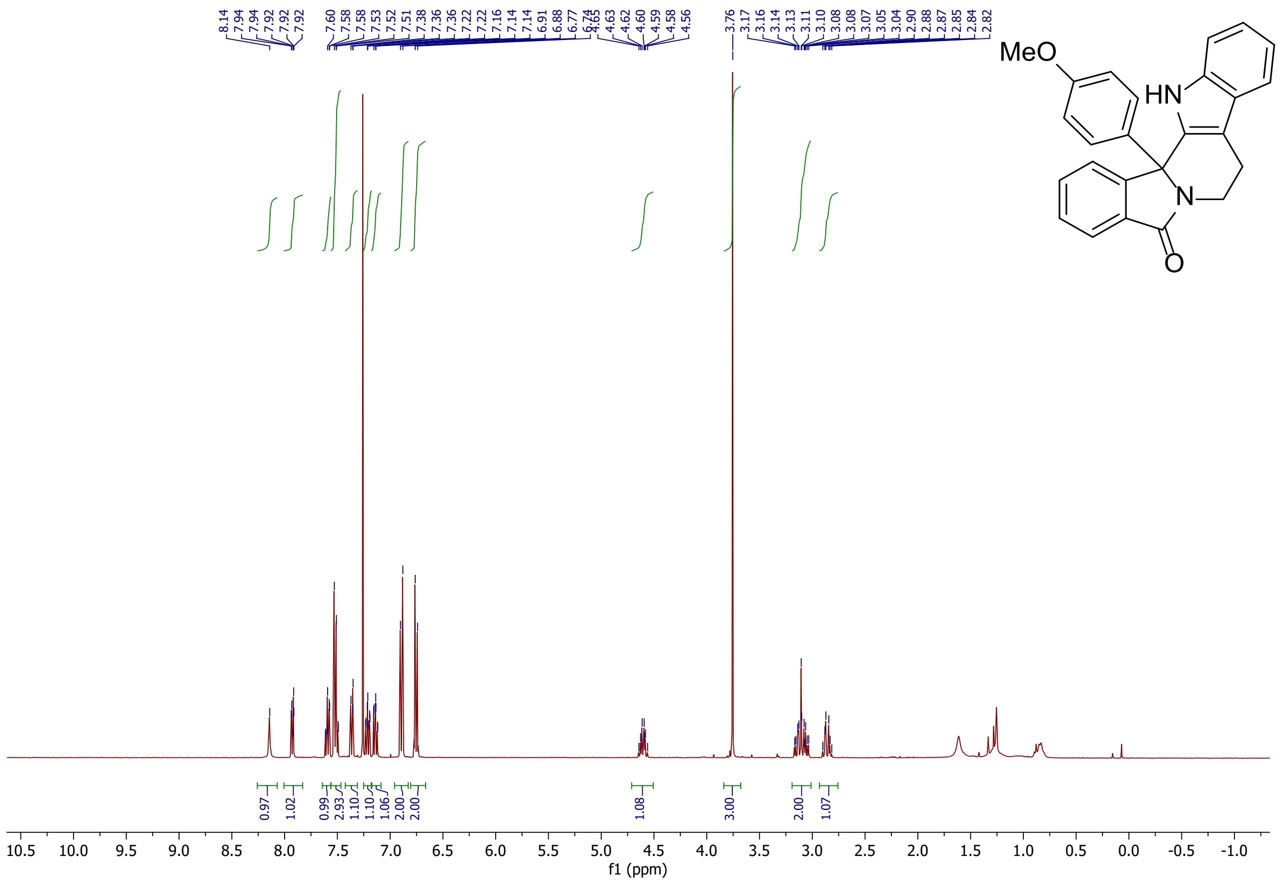

^{13}C NMR (101 MHz, $CDCl_3$)

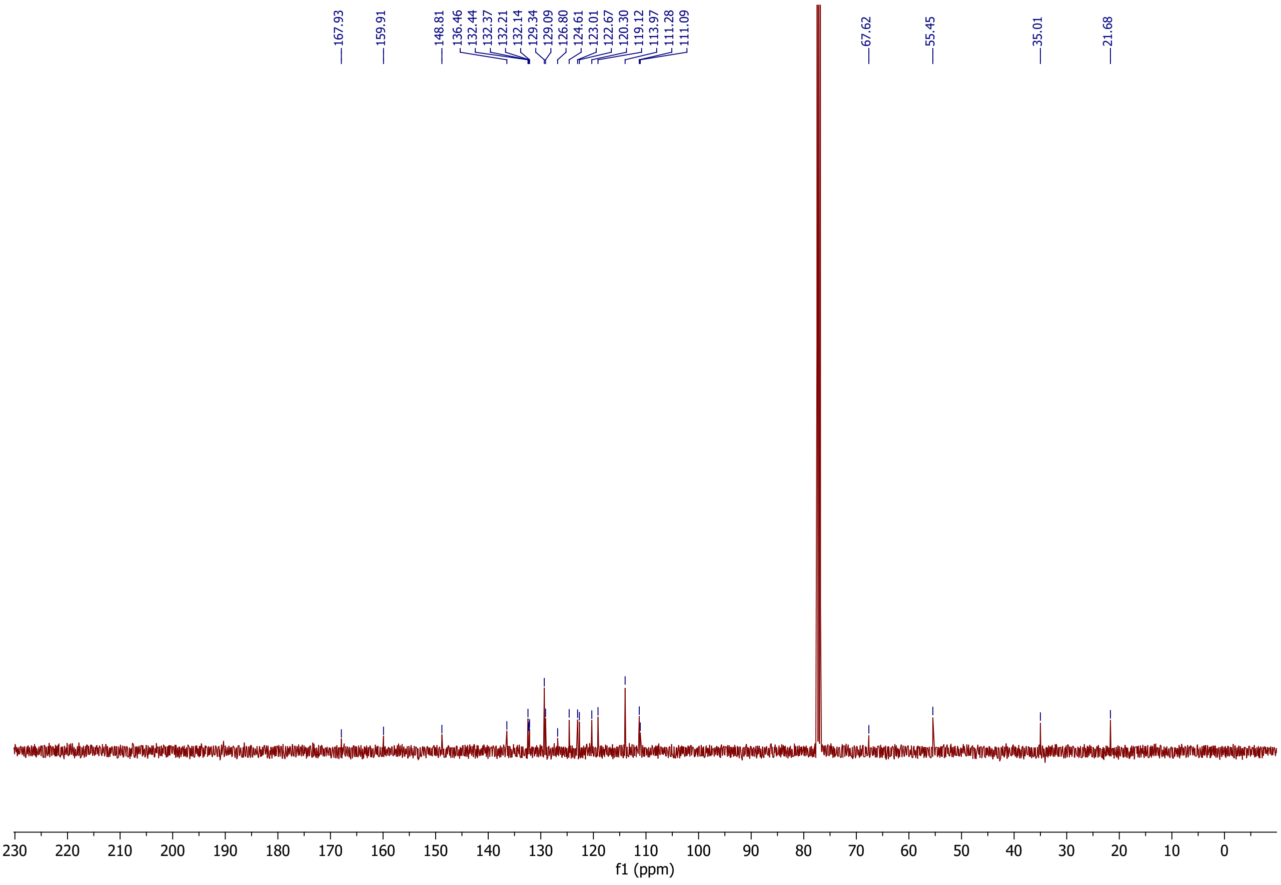

Figure S. 35. 2-(2,4-dimethoxyphenyl)-10,20-diazapentacyclo[11.7.0.02,10.03,8.014,19]icosa-1(13),3,5,7,14(19),15,17-heptaen-9-one (4f)

^{1}H NMR (400 MHz, $CDCl_3$)

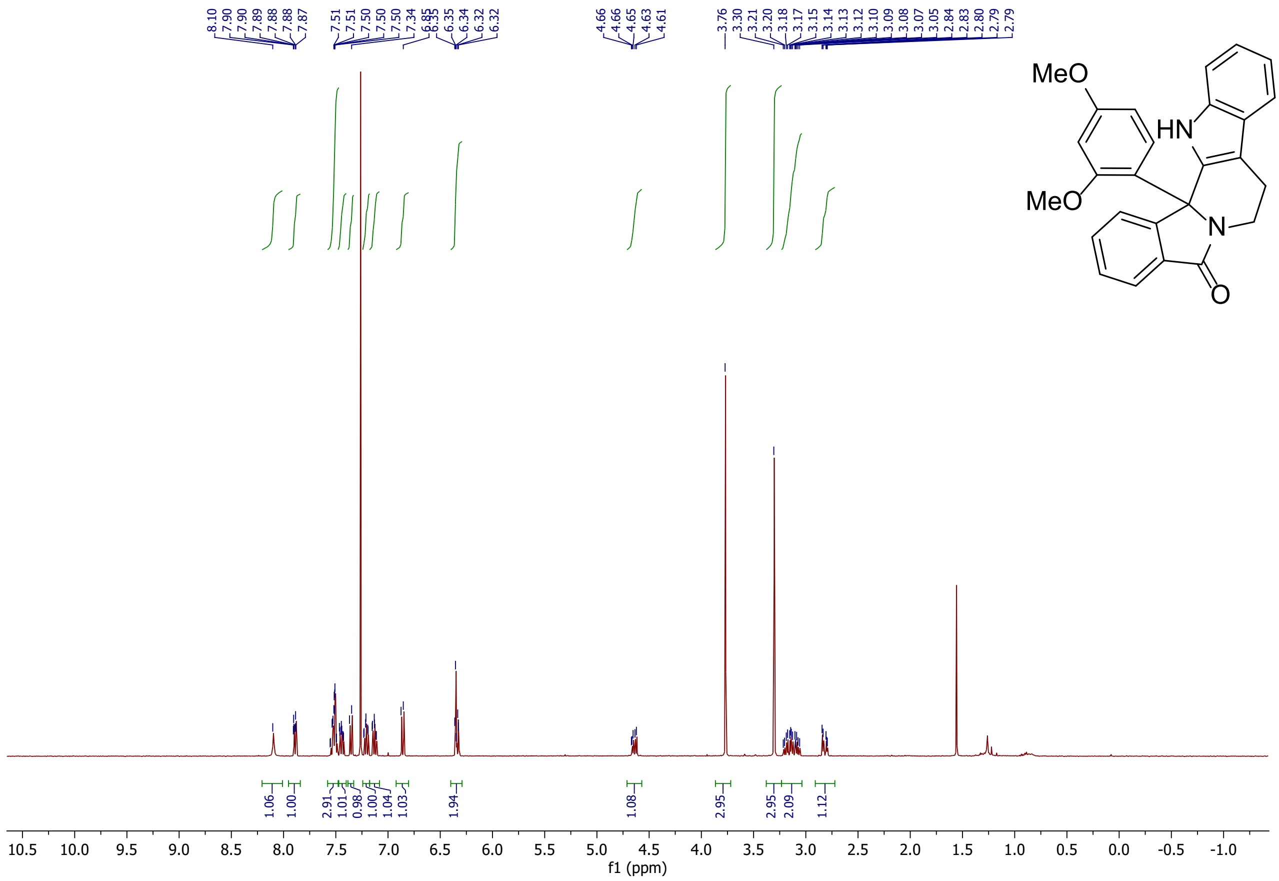

^{13}C NMR (101 MHz, $CDCl_3$)

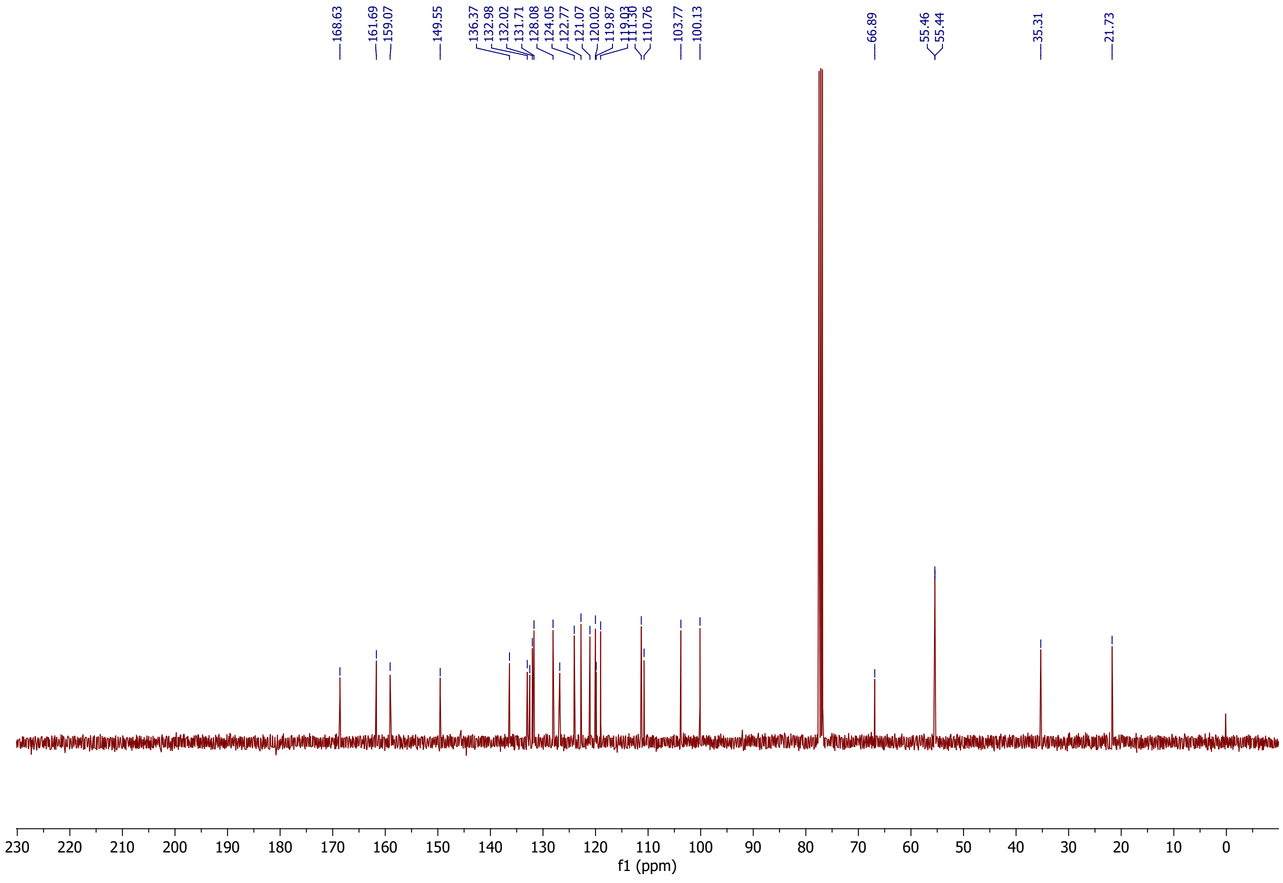

Figure S. 36. 2-(2-furyl)-10,20-diazapentacyclo[11.7.0.02,10.03,8.014,19]icosa-1(13),3,5,7,14(19),15,17-heptaen-9-one (4g)

^{1}H NMR (400 MHz, $CDCl_3$)

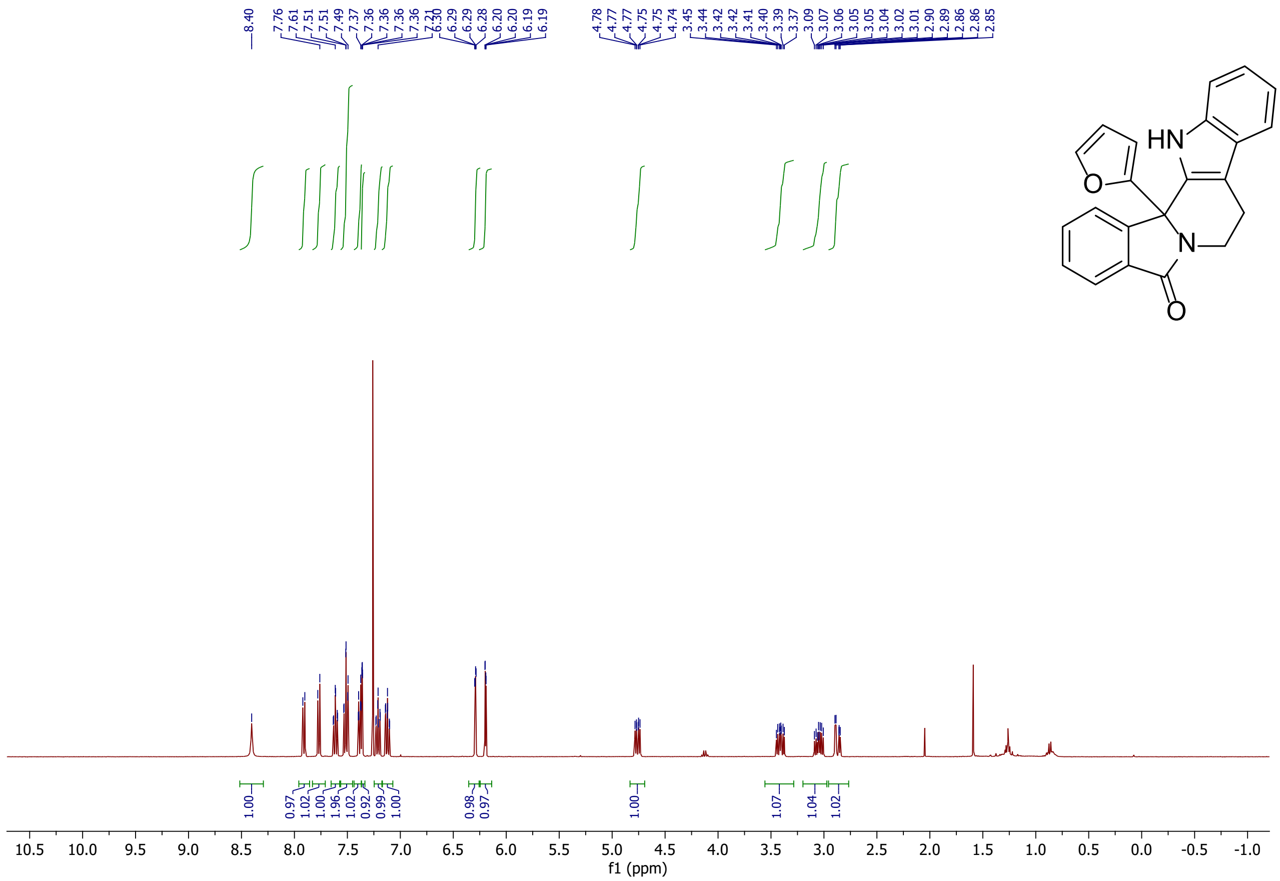

^{13}C NMR (101 MHz, $CDCl_3$)

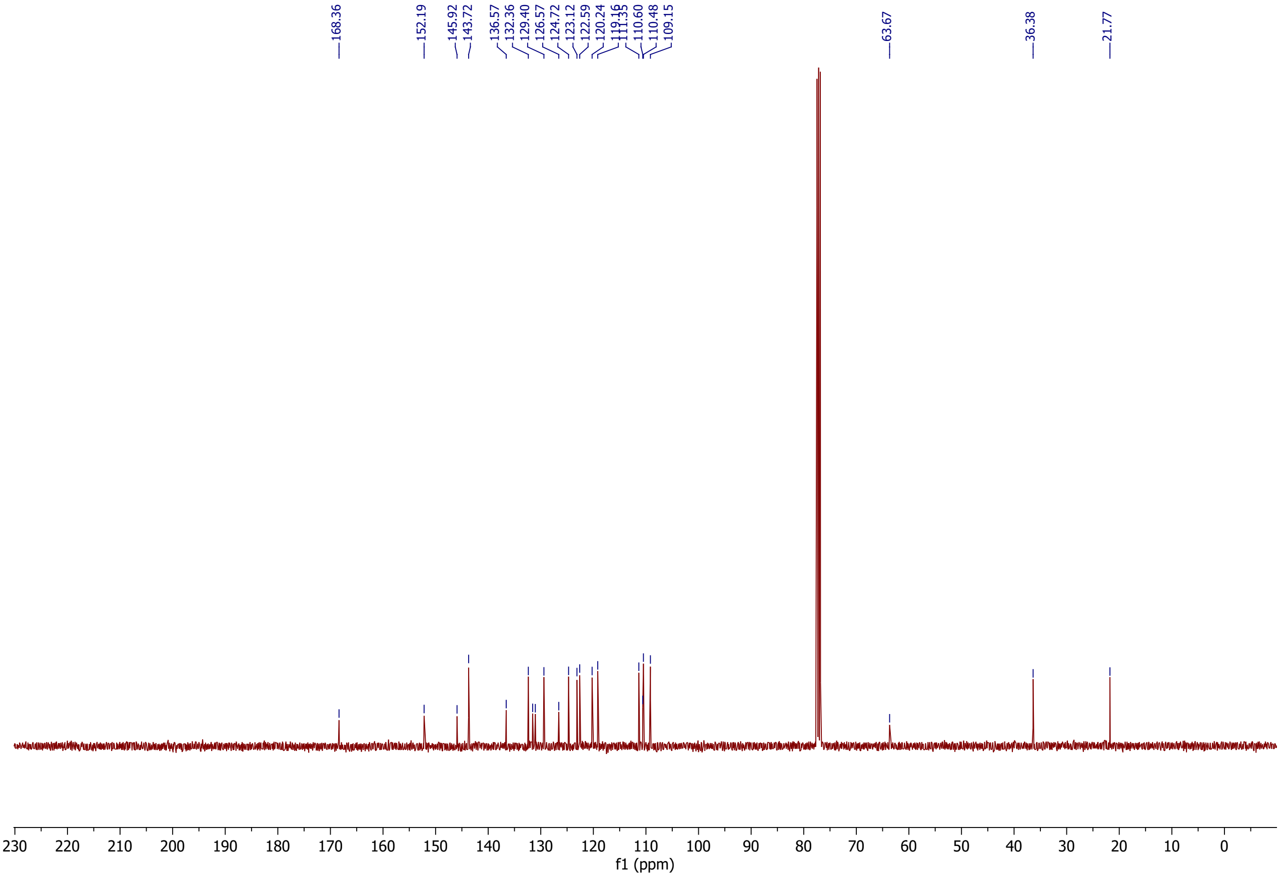

Figure S. 37. 2-(6-methoxy-2-pyridyl)-10,20-diazapentacyclo[11.7.0.02,10.03,8.014,19]icosa-1(13),3,5,7,14(19),15,17-heptaen-9-one (4h)

^{1}H NMR (400 MHz, $CDCl_3$)

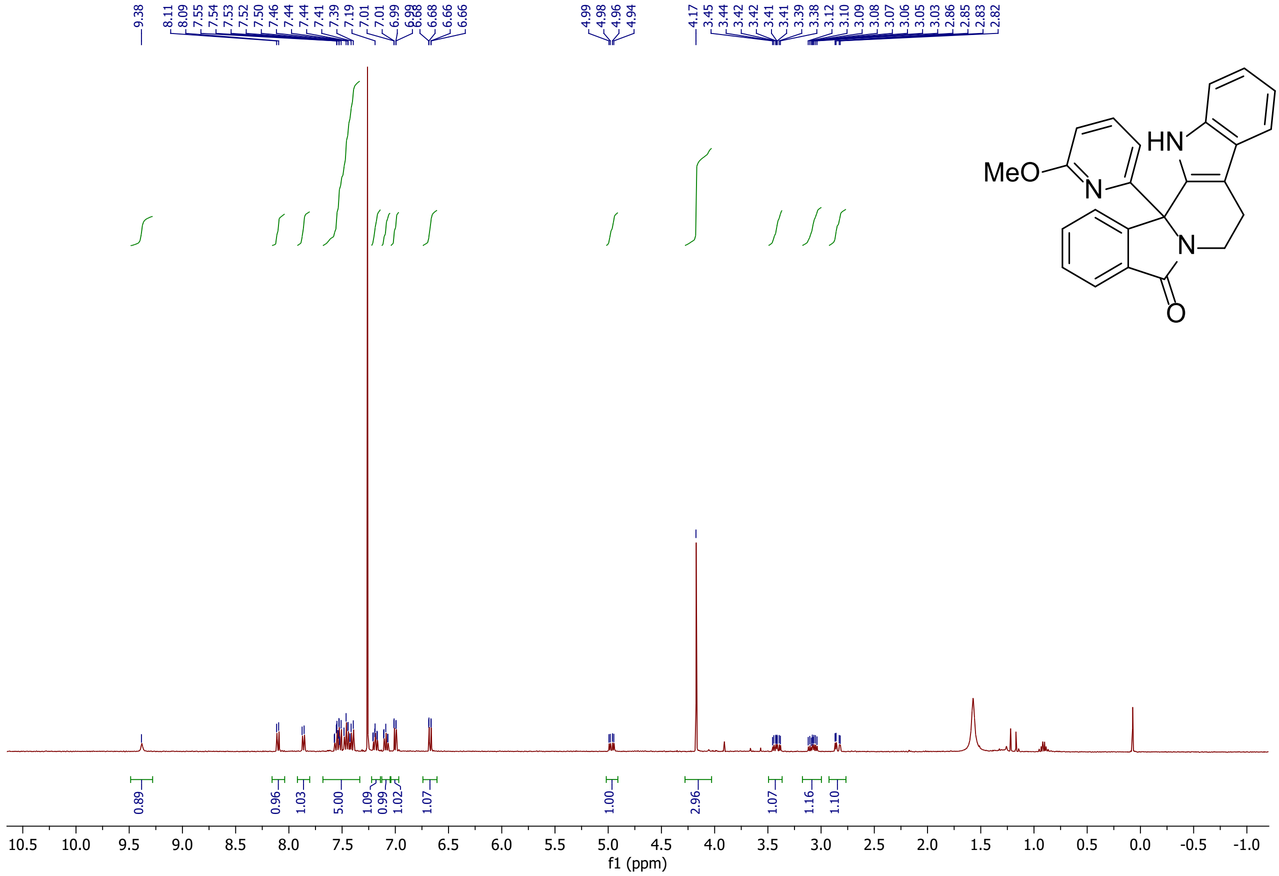

^{13}C NMR (101 MHz, $CDCl_3$)

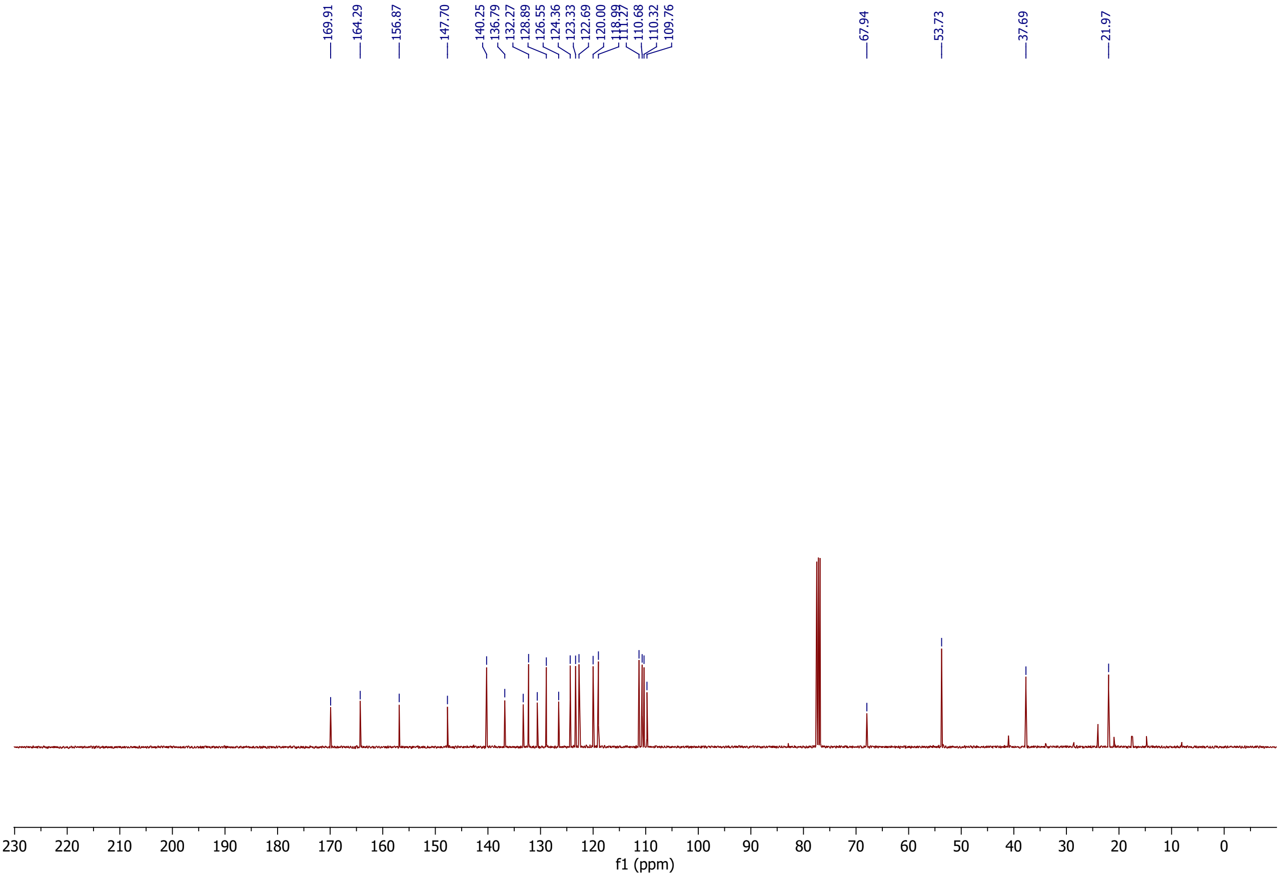

Figure S. 38. 2-(1,3-benzodioxol-5-yl)-10,20-diazapentacyclo[11.7.0.02,10.03,8.014,19]icosa-1(13),3,5,7,14(19),15,17-heptaen-9-one (4i)

^{1}H NMR (400 MHz, DMSO-d$_6$)

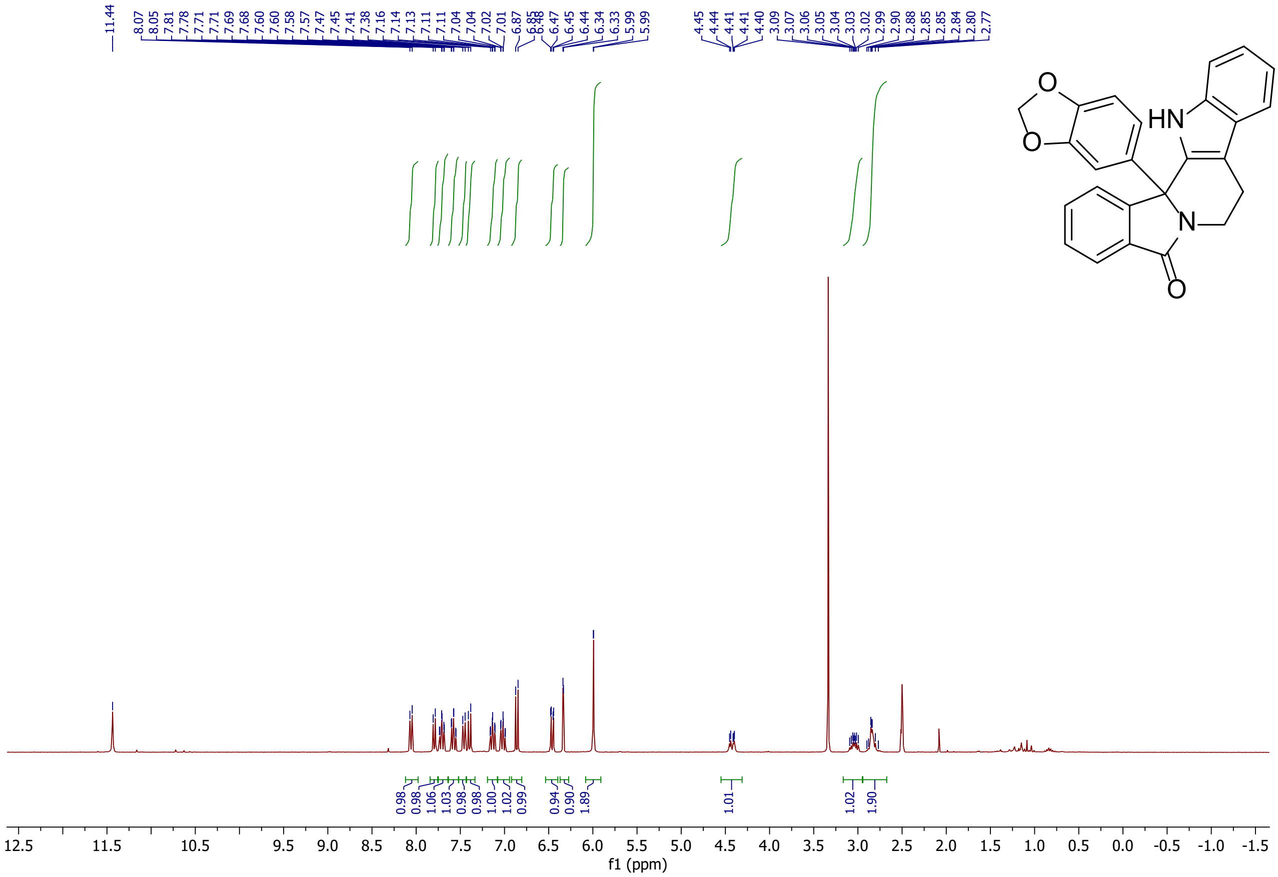

^{13}C NMR (101 MHz, DMSO-d$_6$)

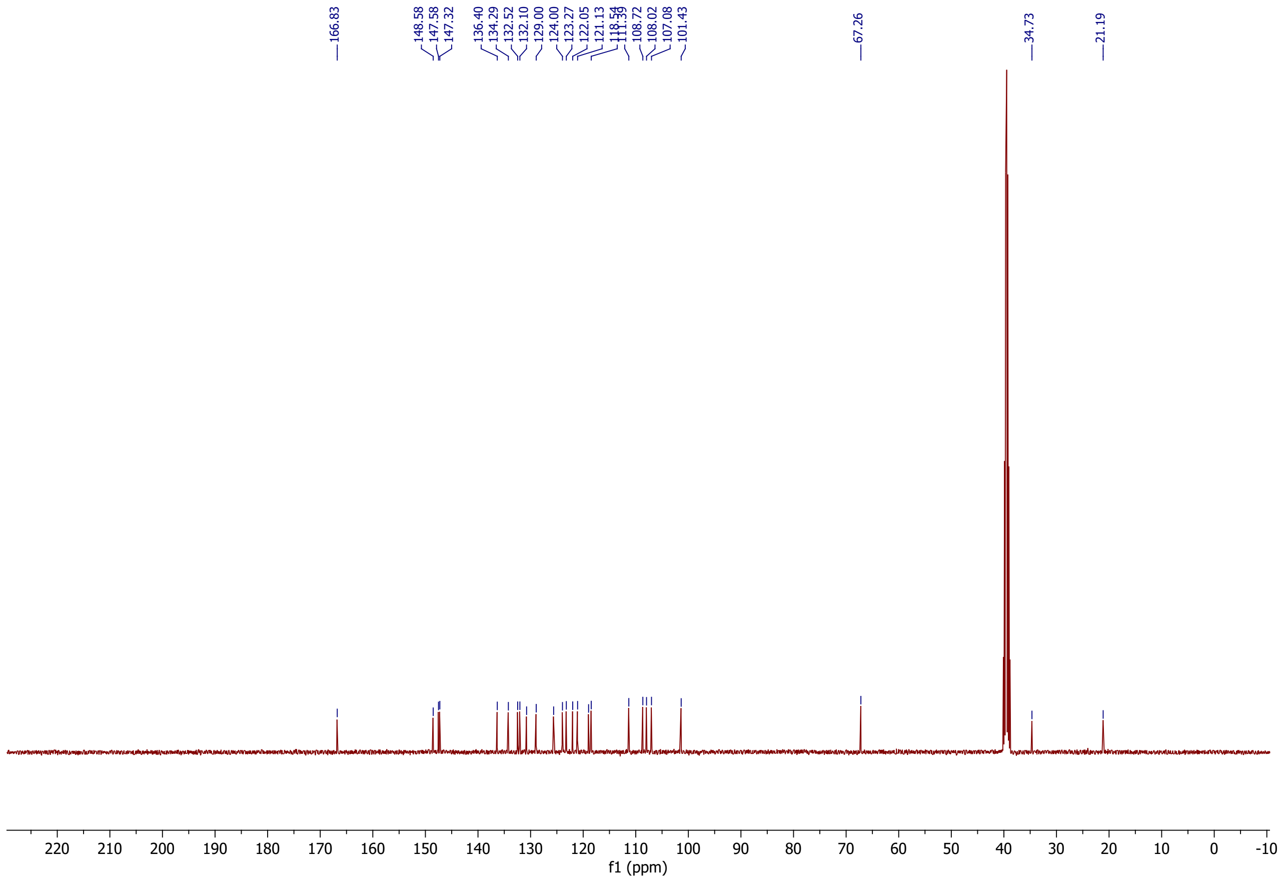

Figure S. 39. (1,3-dioxo-1,3-dihydro-2*H*-isoindol-2-yl)acetic acid (Phthalimidoglycine)

^{1}H NMR (400 MHz, DMSO-d_6)

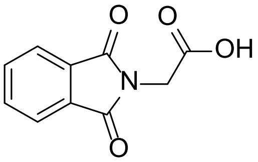

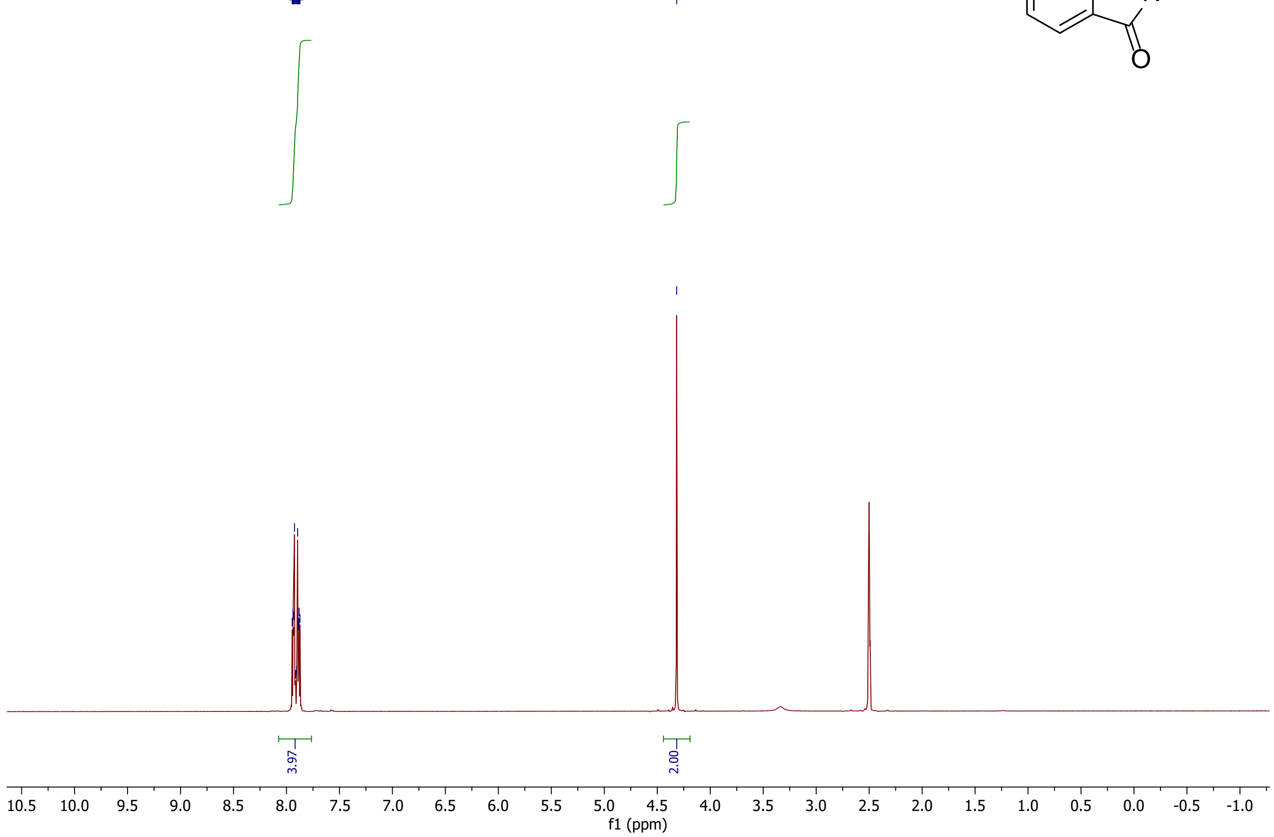

^{13}C NMR (101 MHz, DMSO-d_6)

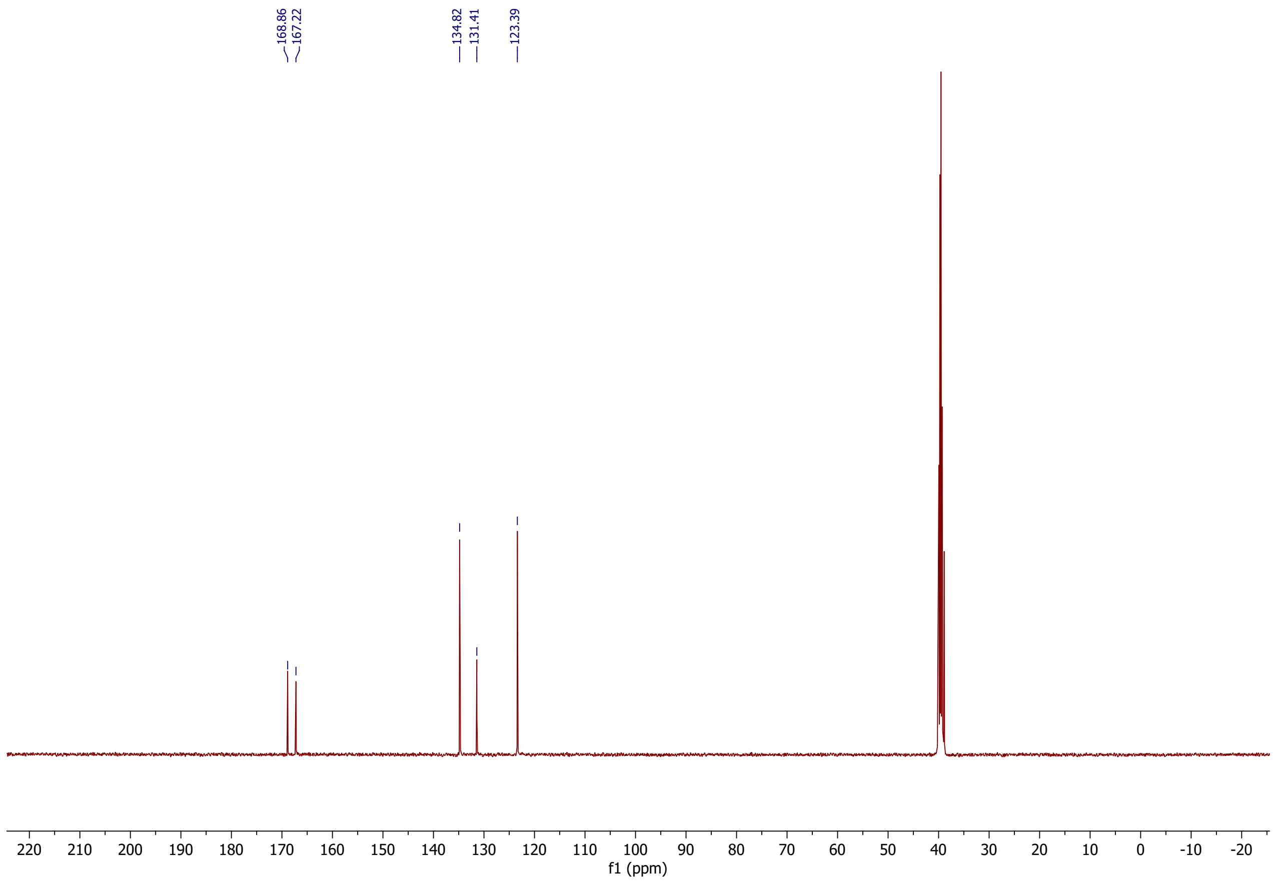

Figure S. 40. N-benzyl-2-(1,3-dioxoisoindolin-2-yl)acetamide

^{1}H NMR (400 MHz, $CDCl_3$)

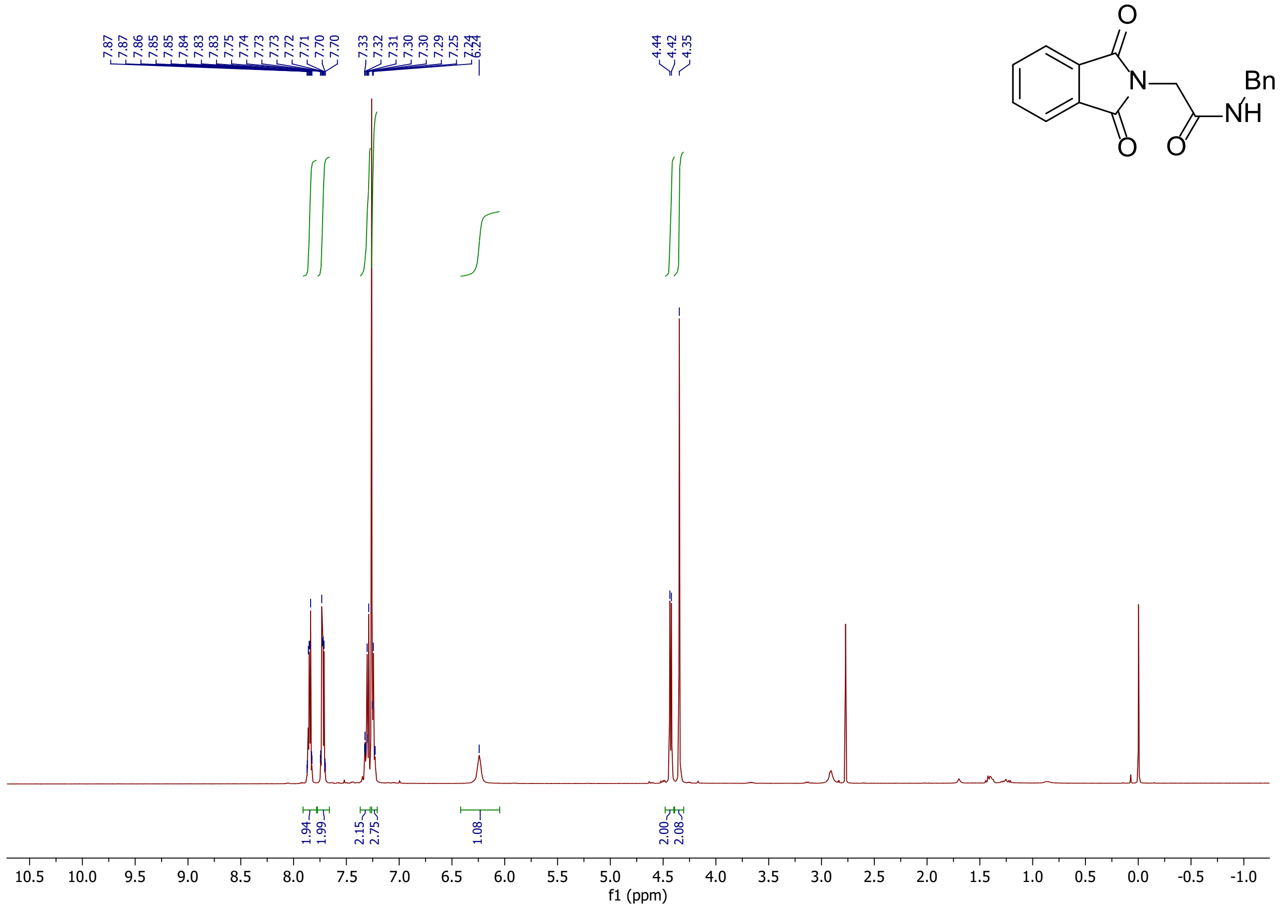

^{13}C NMR (101 MHz, $CDCl_3$)

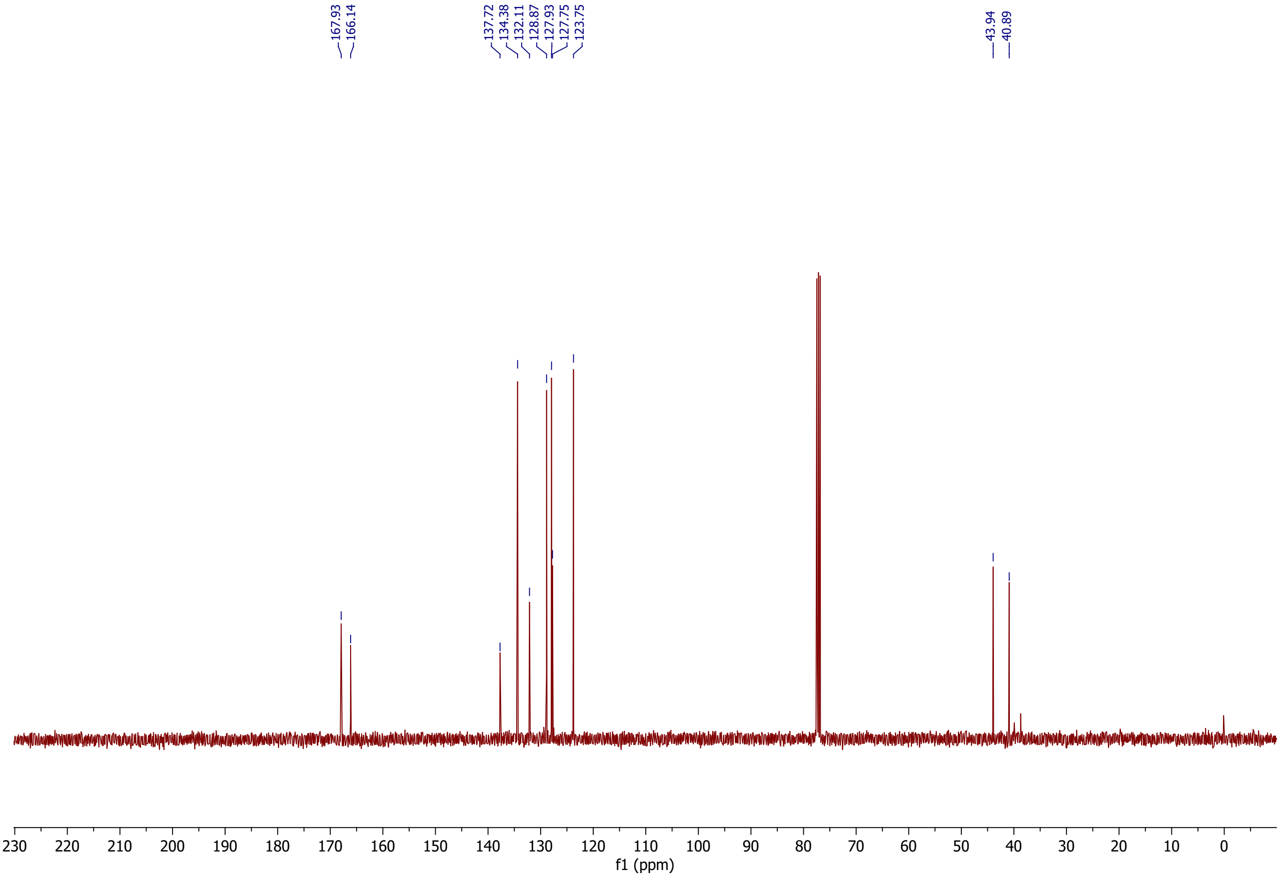

Figure S. 41. N-cyclopropyl-2-(1,3-dioxoisoindolin-2-yl)acetamide

^{1}H NMR (400 MHz, DMSO-d_6)

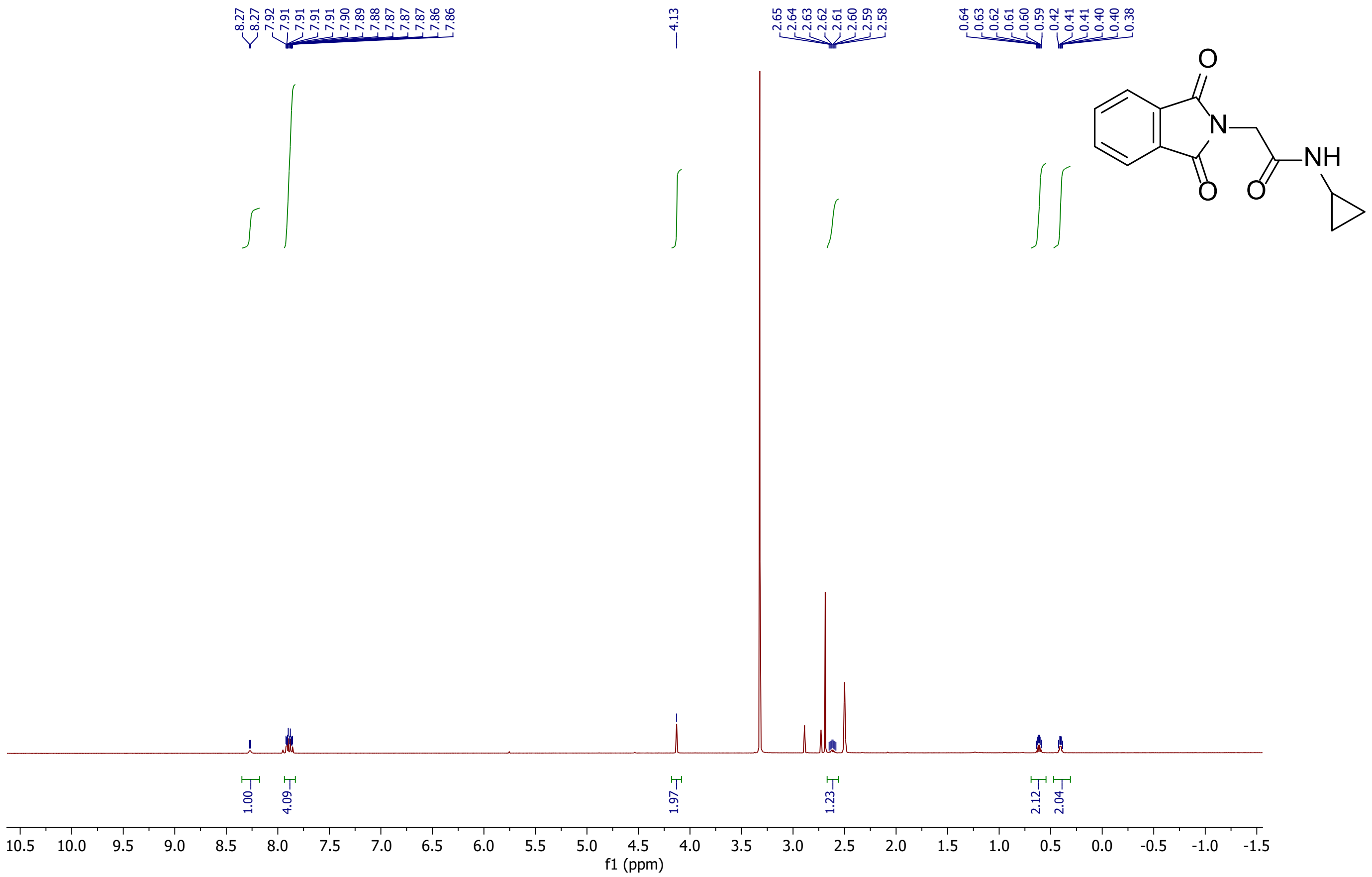

^{13}C NMR (101 MHz, DMSO-d_6)

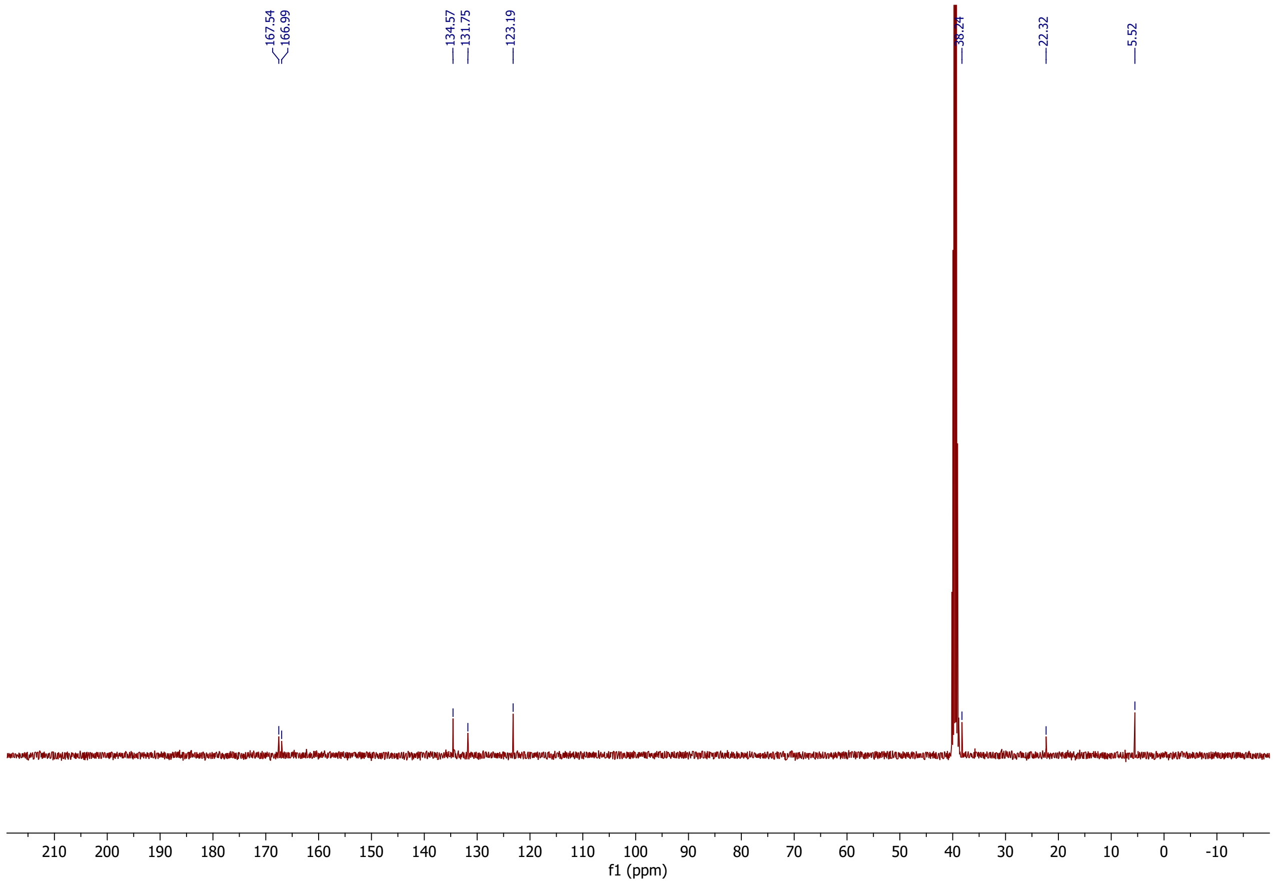

Figure S. 42. 2-(1,3-dioxoisoindolin-2-yl)-N-isobutyl-acetamide

^{1}H NMR (400 MHz, $CDCl_3$)

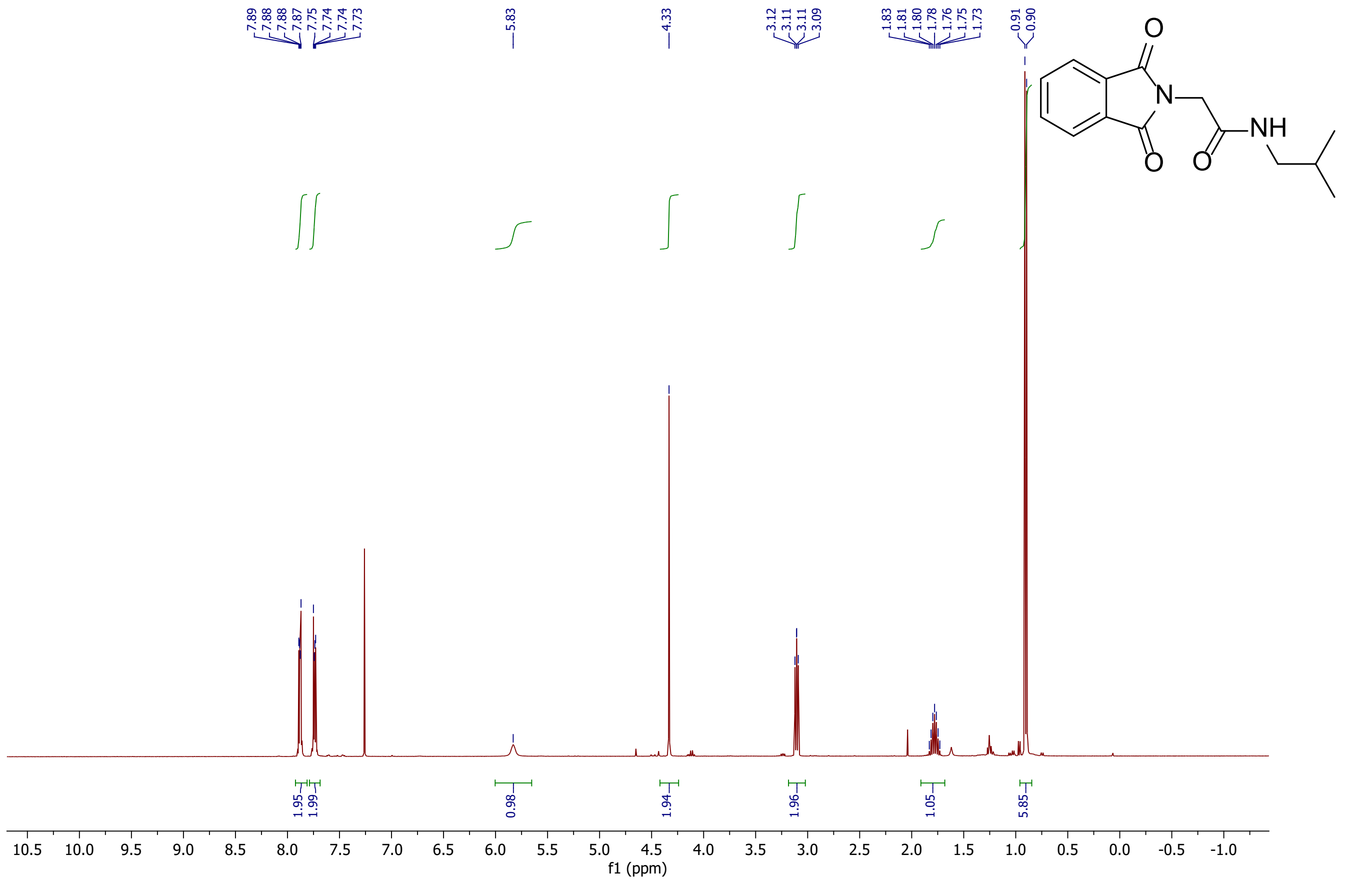

^{13}C NMR (101 MHz, $CDCl_3$)

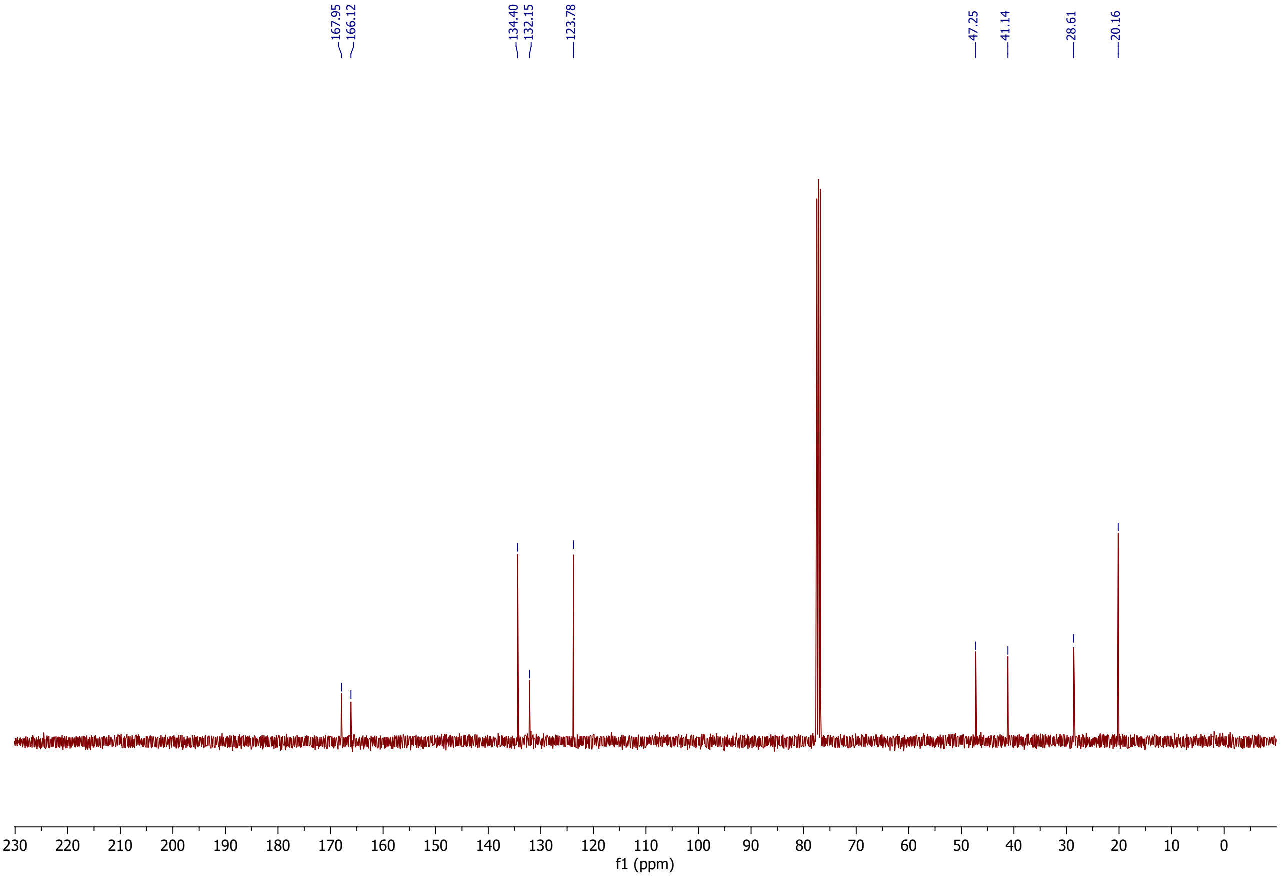

Figure S. 43. N-[(3-chlorophenyl)methyl]-2-(1,3-dioxoisoindolin-2-yl)acetamide

^{1}H NMR (400 MHz, $CDCl_3$)

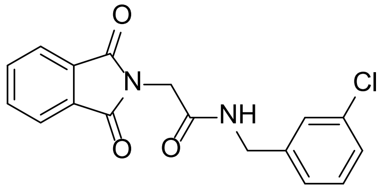

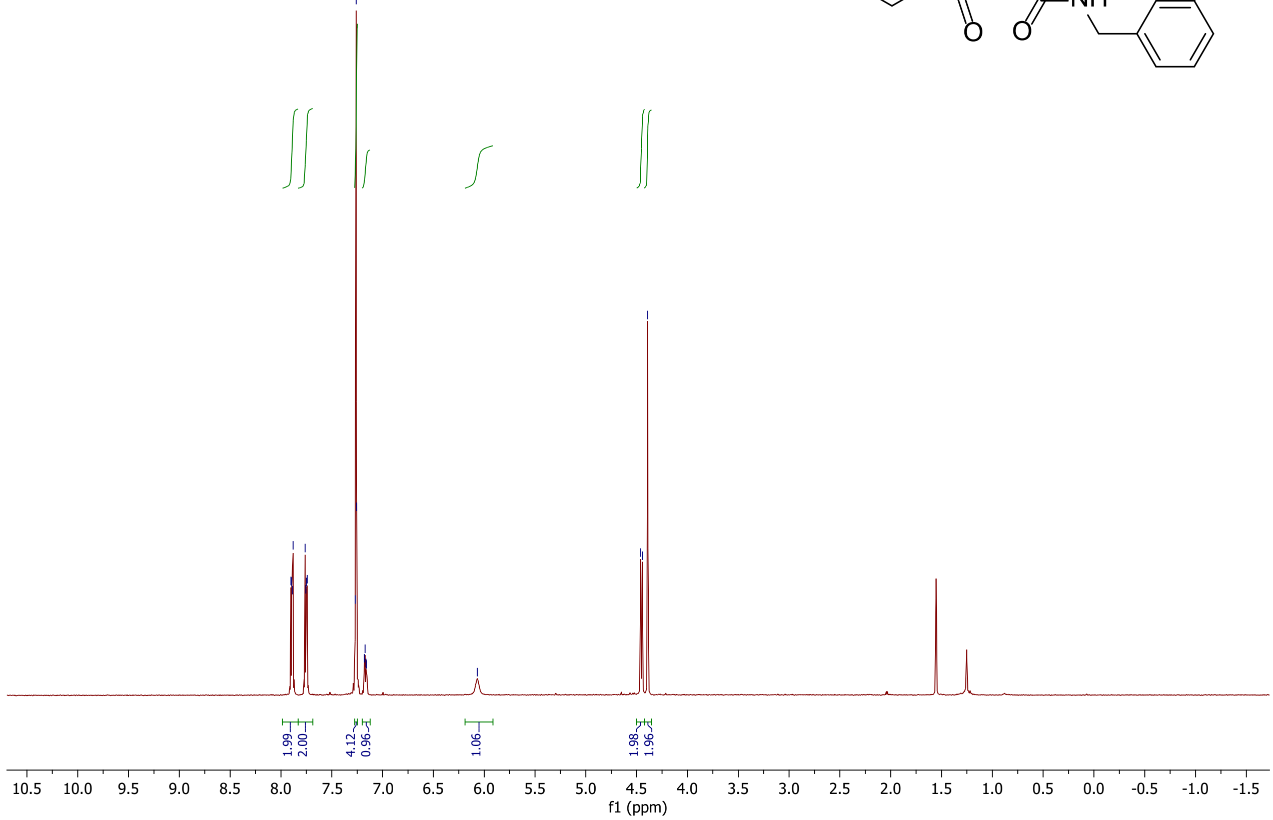

^{13}C NMR (101 MHz, $CDCl_3$)

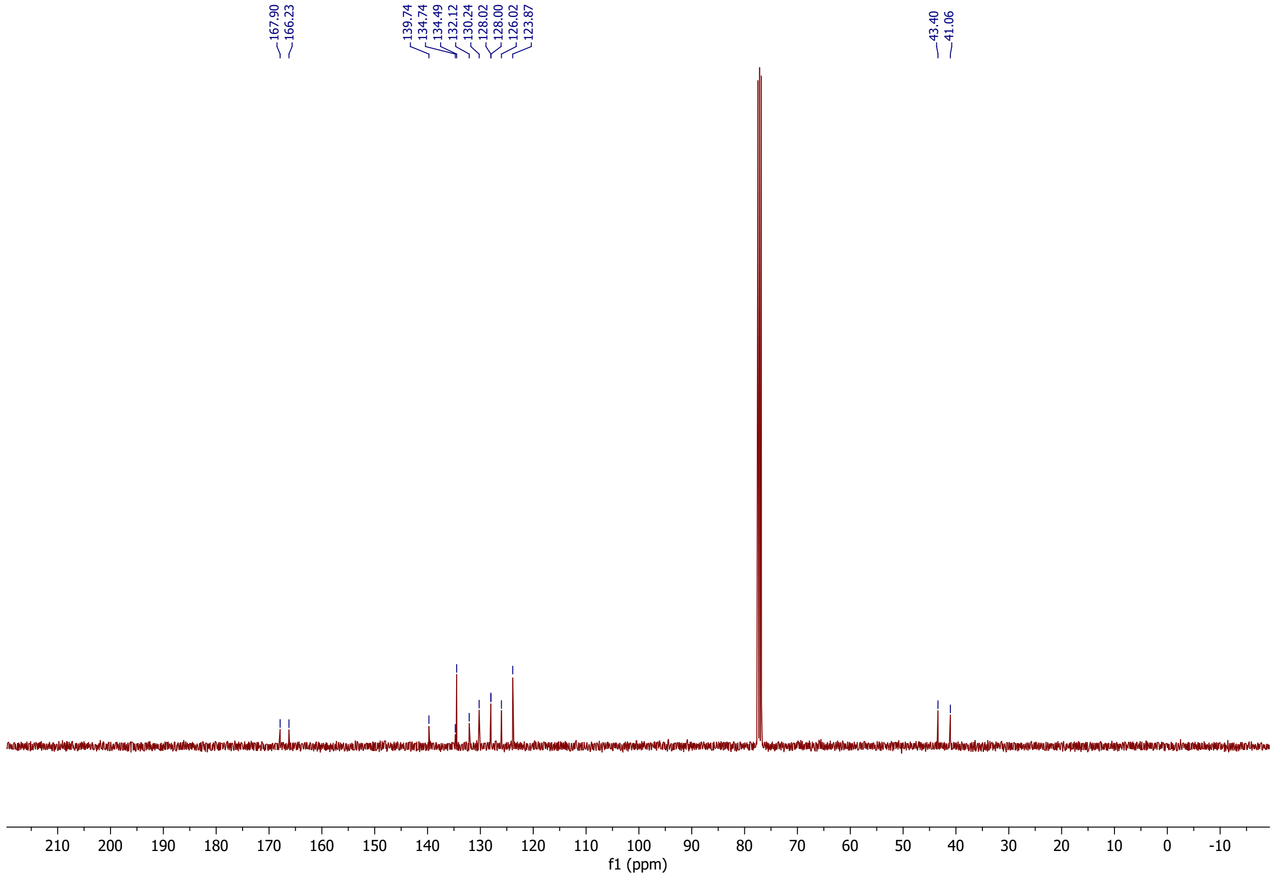

Figure S. 44. 2-(1,3-dioxoisoindolin-2-yl)-N-(4-fluorophenyl)acetamide

^{1}H NMR (400 MHz, $CDCl_3$)

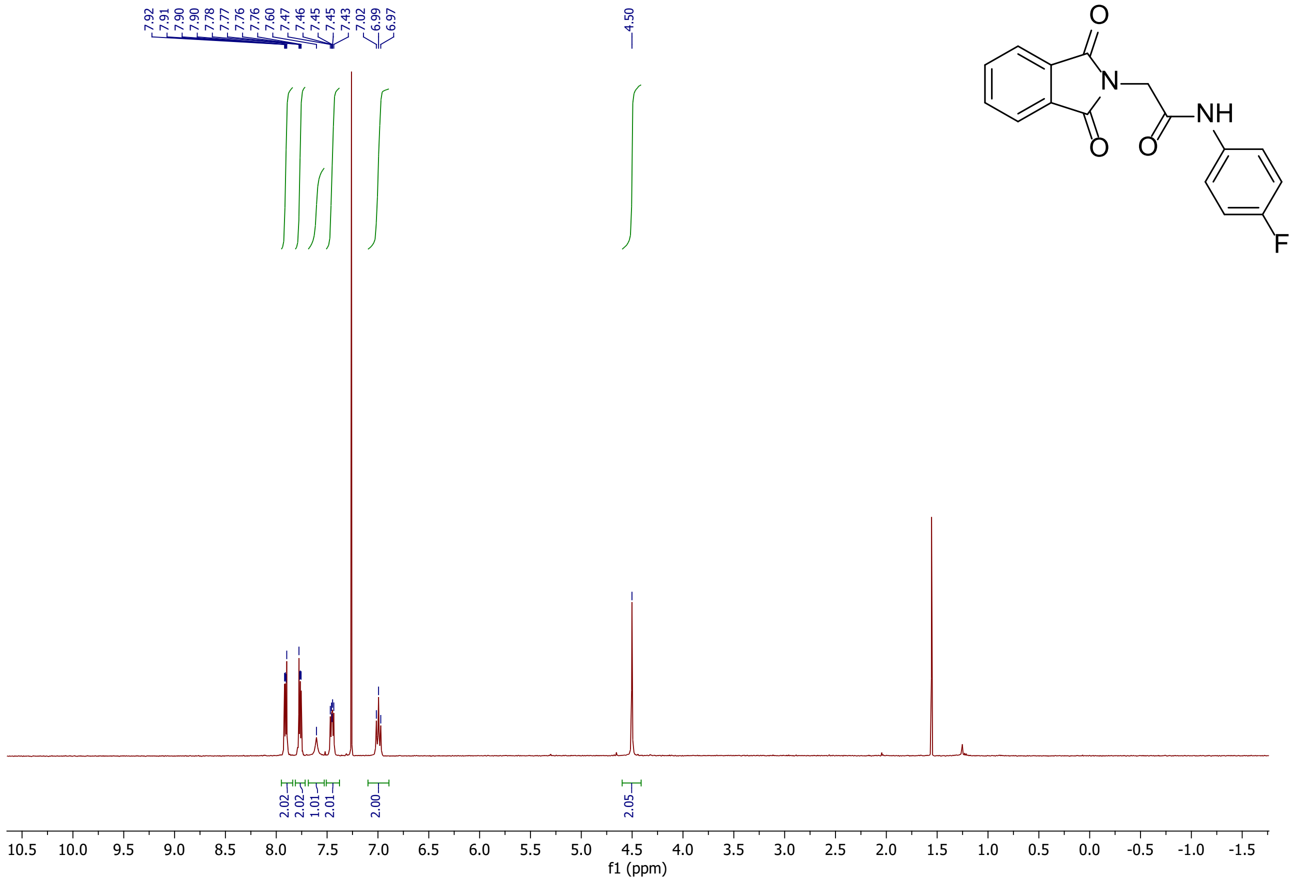

^{13}C NMR (101 MHz, DMSO-d_6)

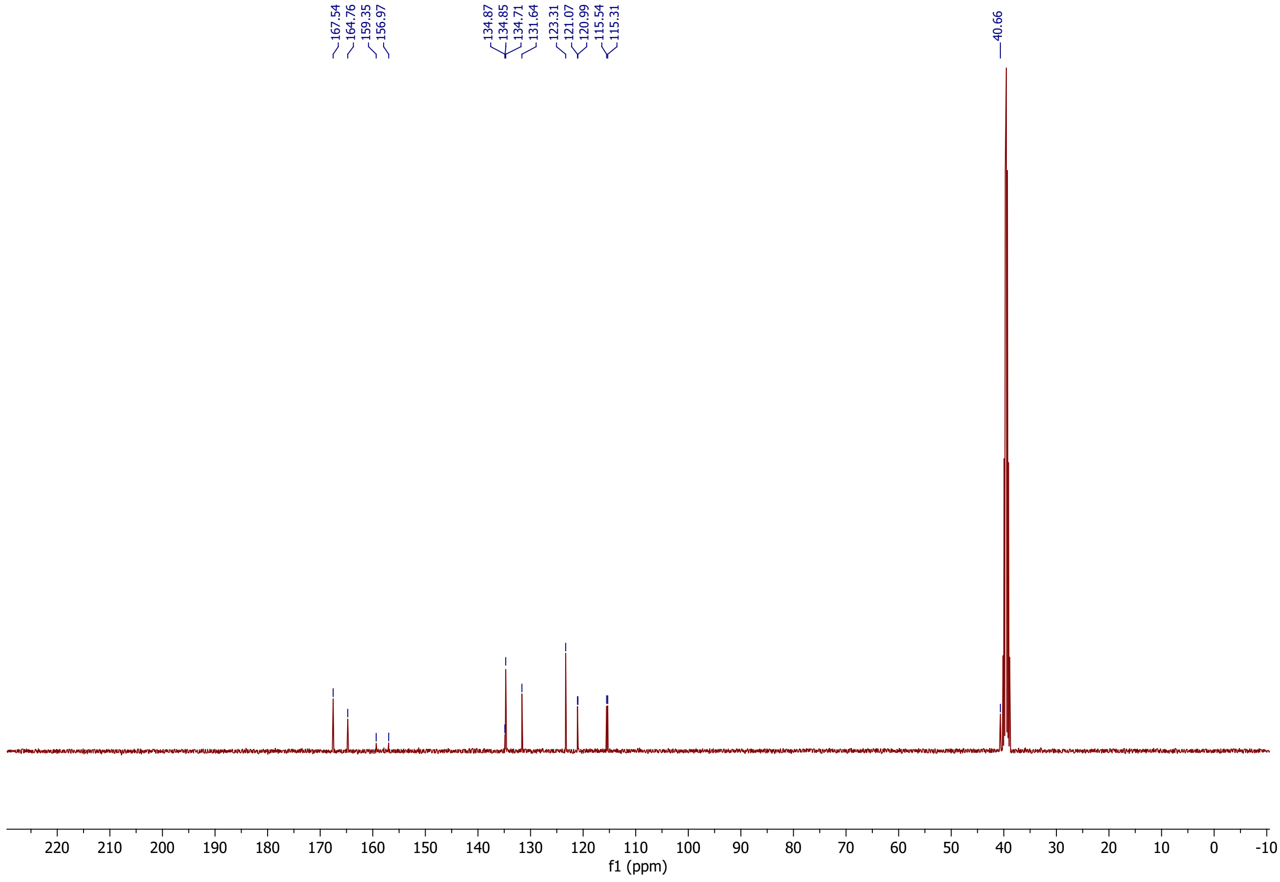

Figure S. 45. N-(2-chloro-4-methyl-phenyl)-2-(1,3-dioxoisoindolin-2-yl)acetamide

^{1}H NMR (400 MHz, $CDCl_3$)

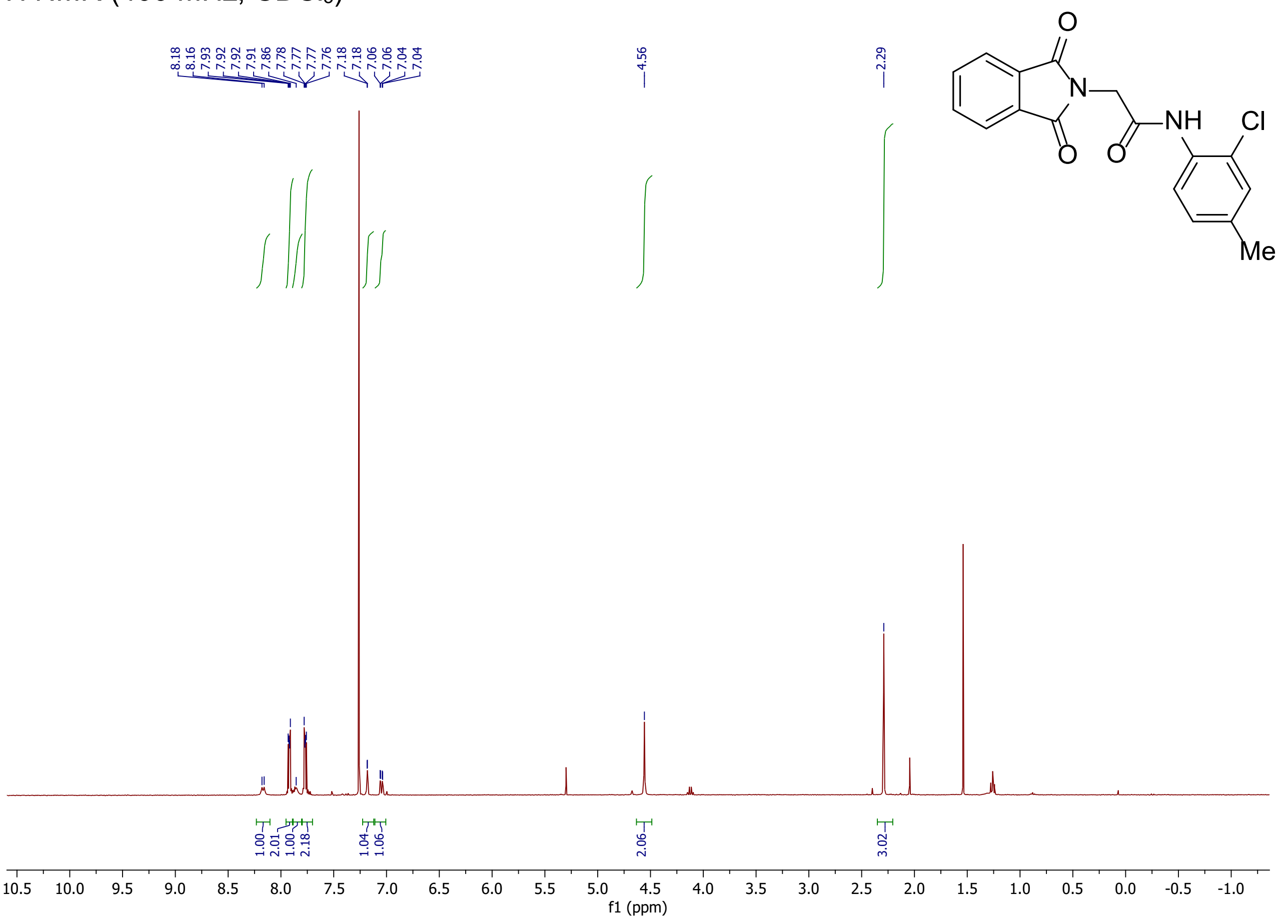

^{13}C NMR (101 MHz, $CDCl_3$)

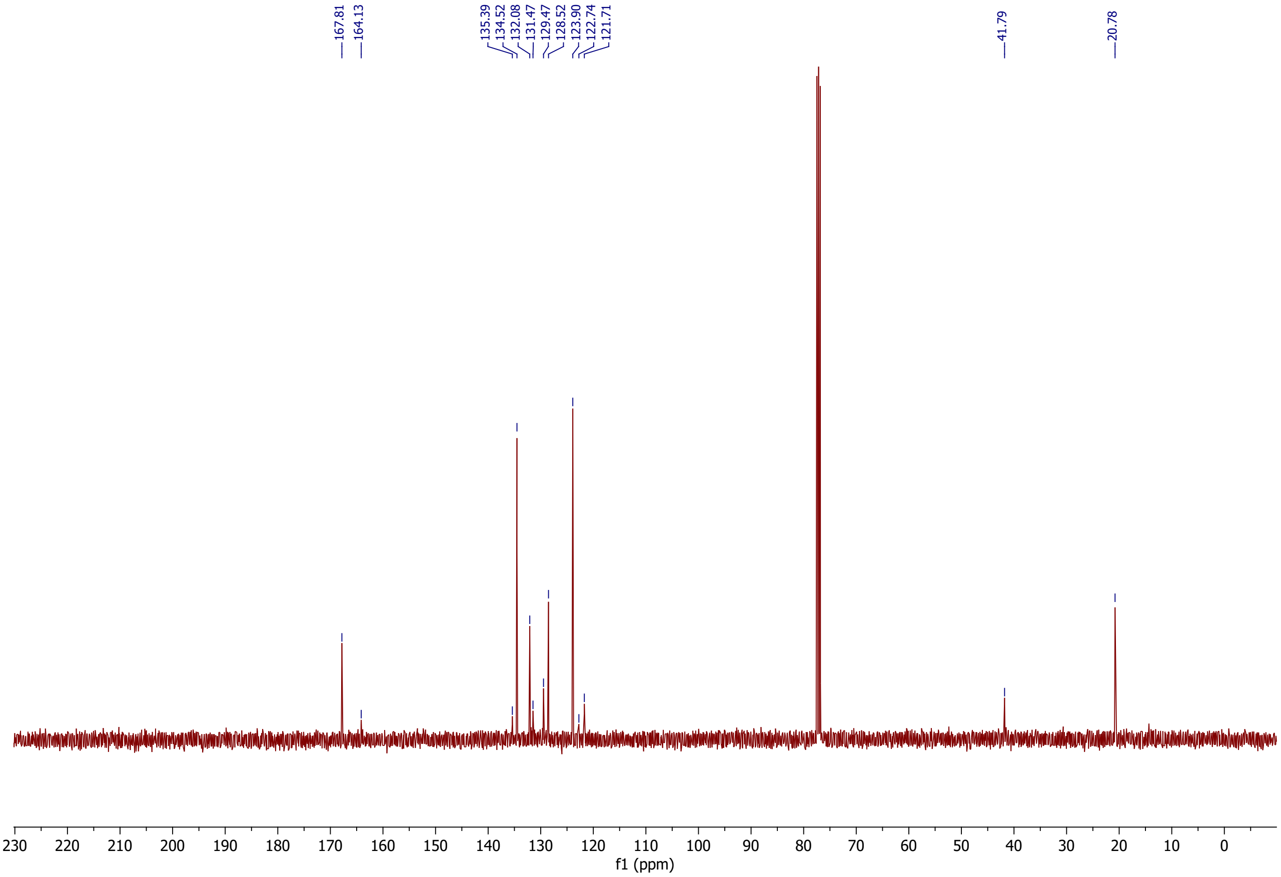

Figure S. 46. N-benzyl-2-(1-hydroxy-3-oxo-1-phenyl-isoindolin-2-yl)acetamide (5a)

^{1}H NMR (400 MHz, $CDCl_3$)

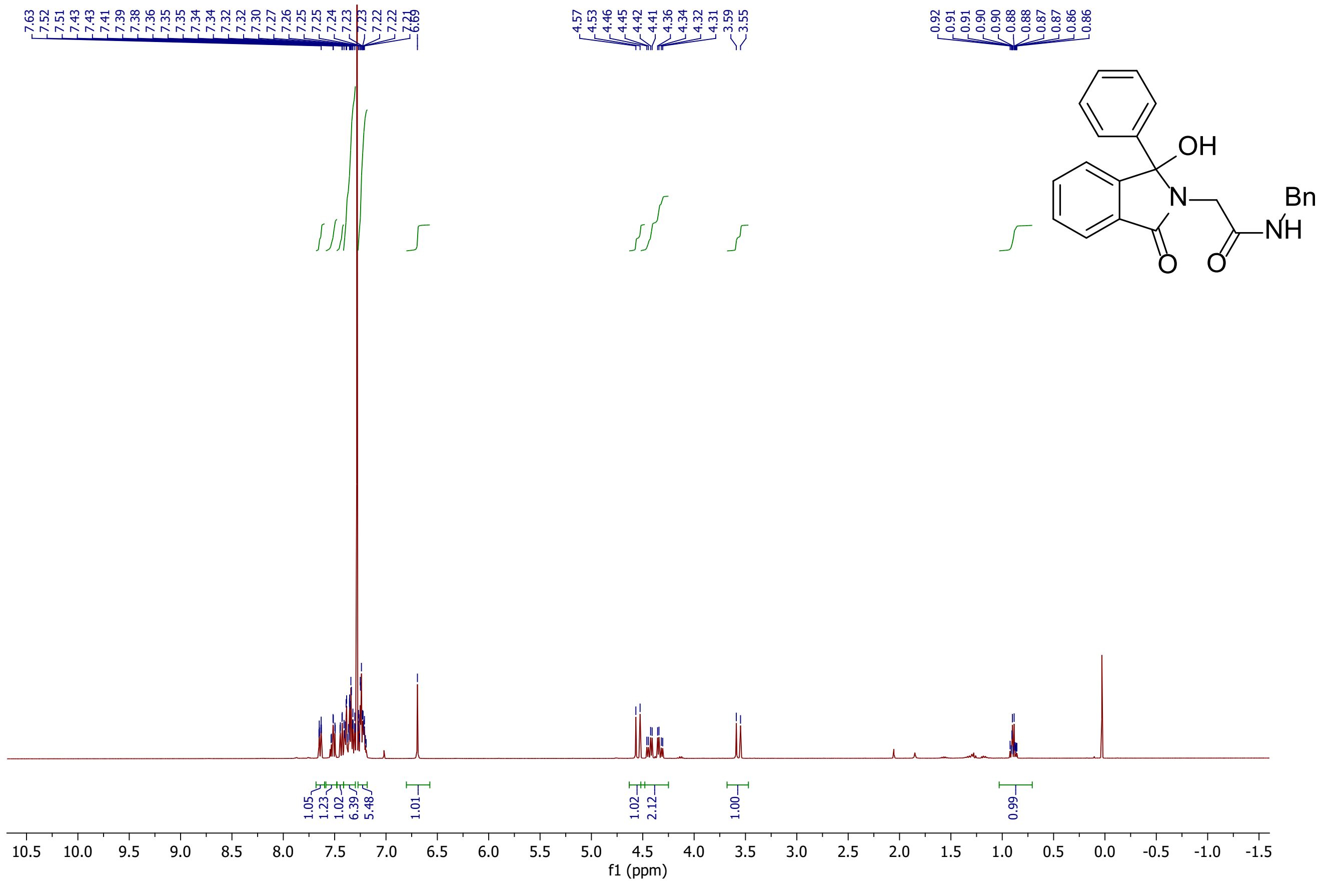

^{13}C NMR (101 MHz, $CDCl_3$)

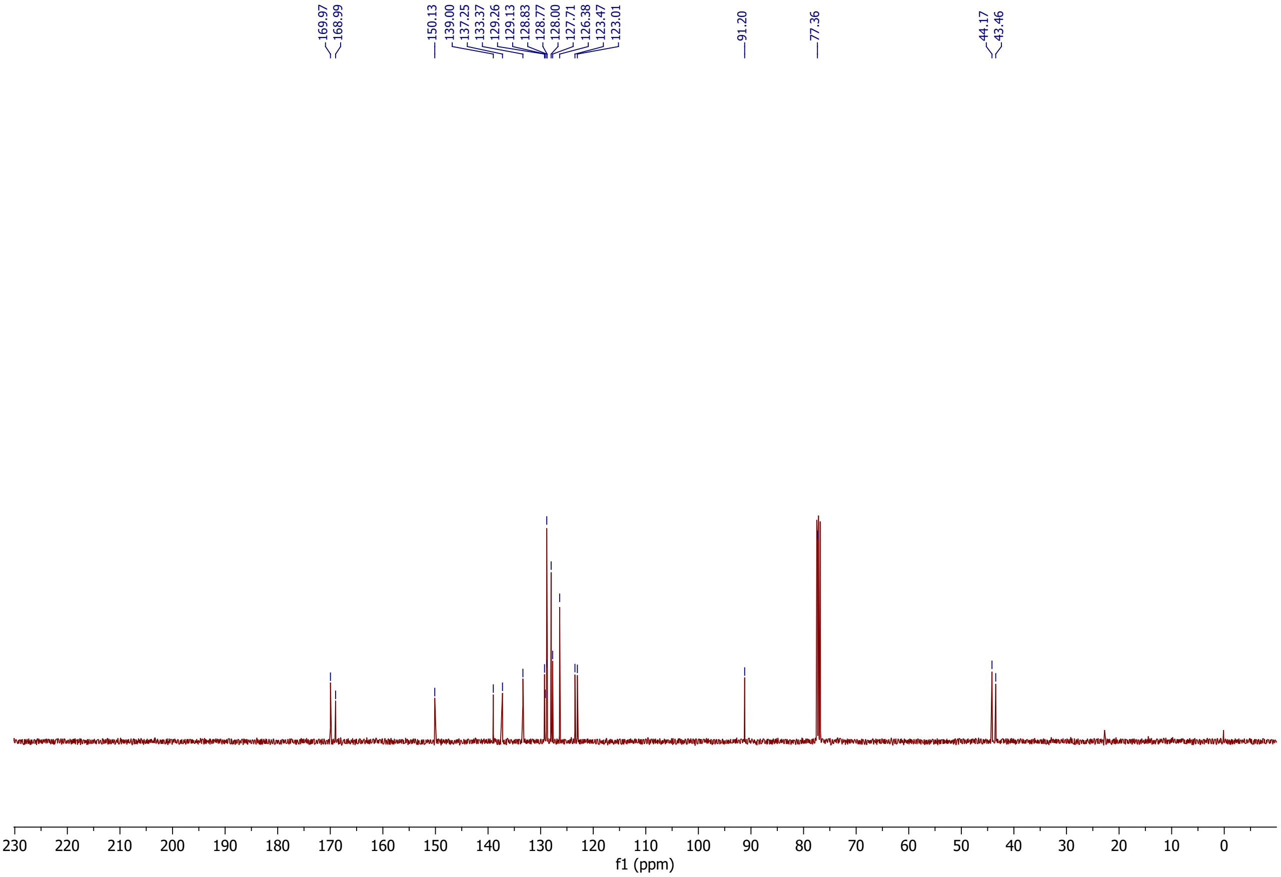

Figure S. 47. N-benzyl-2-[1-hydroxy-1-(4-methoxyphenyl)-3-oxo-isoindolin-2-yl]acetamide (5b)

^{1}H NMR (400 MHz, $CDCl_3$)

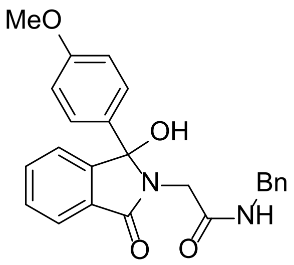

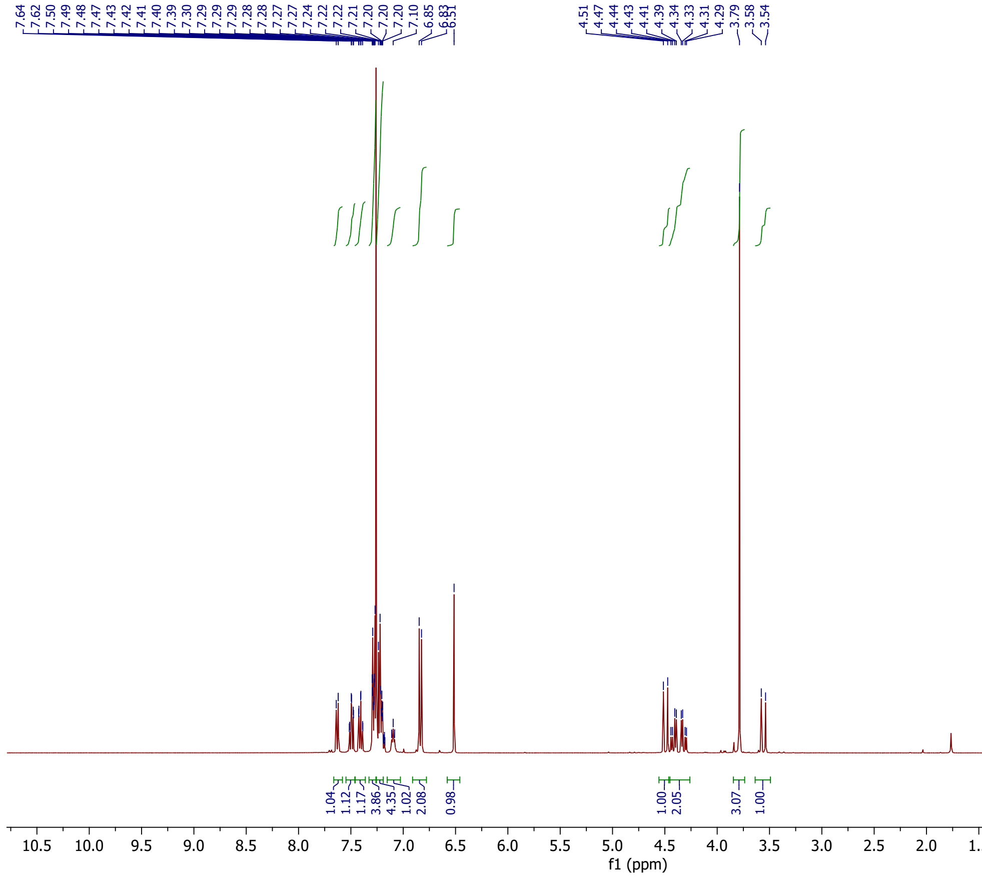

^{13}C NMR (101 MHz, $CDCl_3$)

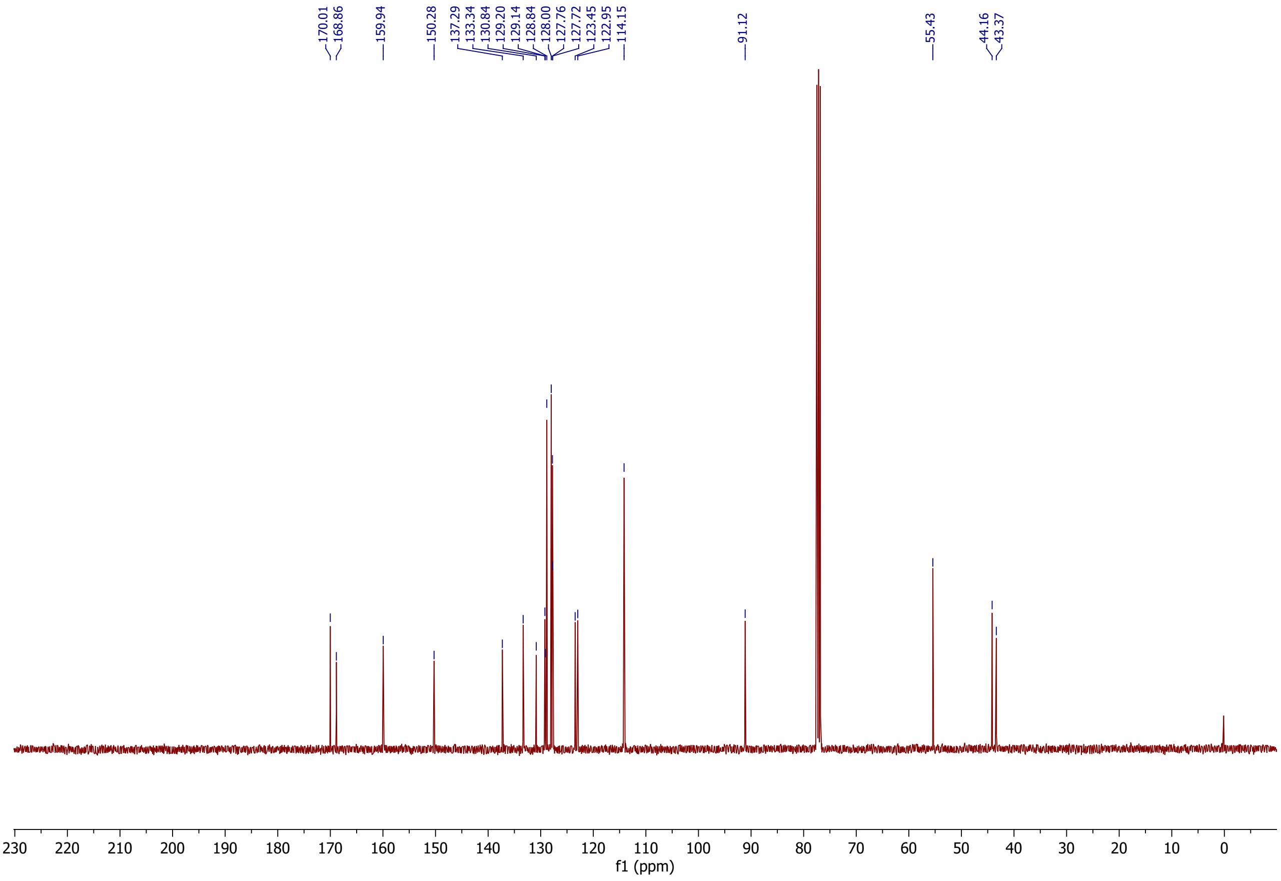

Figure S. 48. N-benzyl-2-[1-hydroxy-3-oxo-1-[4-(trifluoromethyl)phenyl]isoindolin-2-yl]acetamide (5c)

^{1}H NMR (400 MHz, $CDCl_3$)

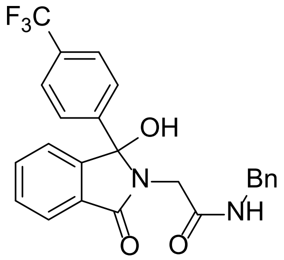

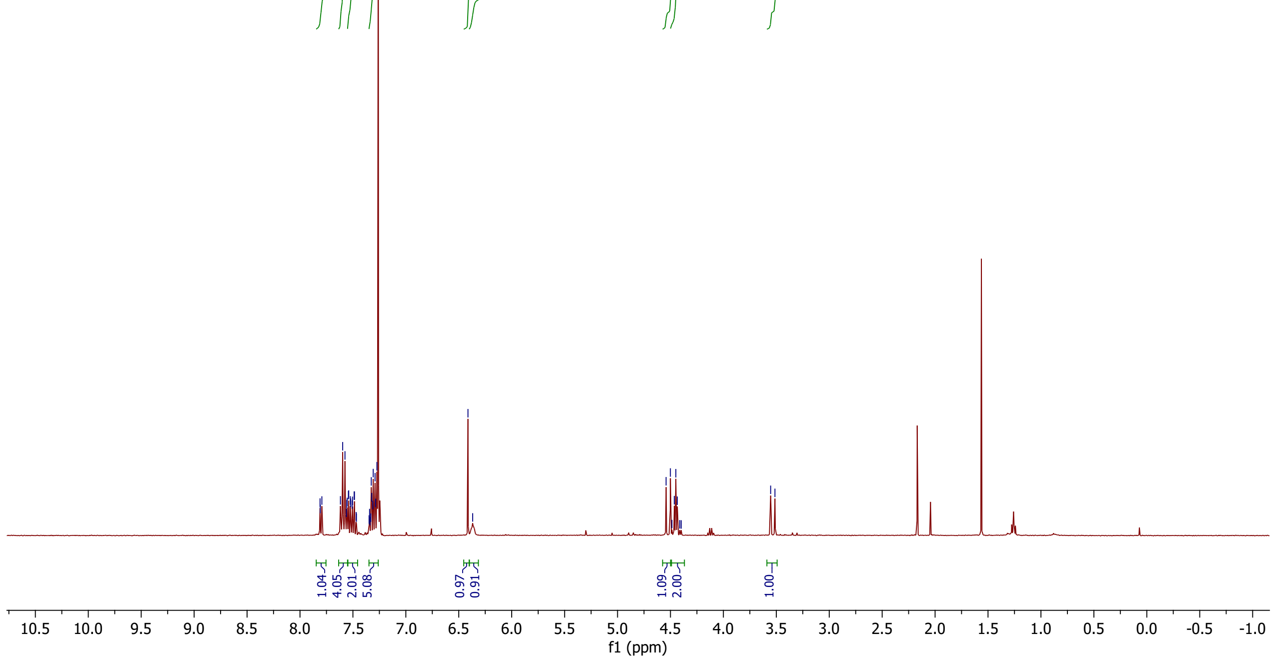

^{13}C NMR (101 MHz, $CDCl_3$)

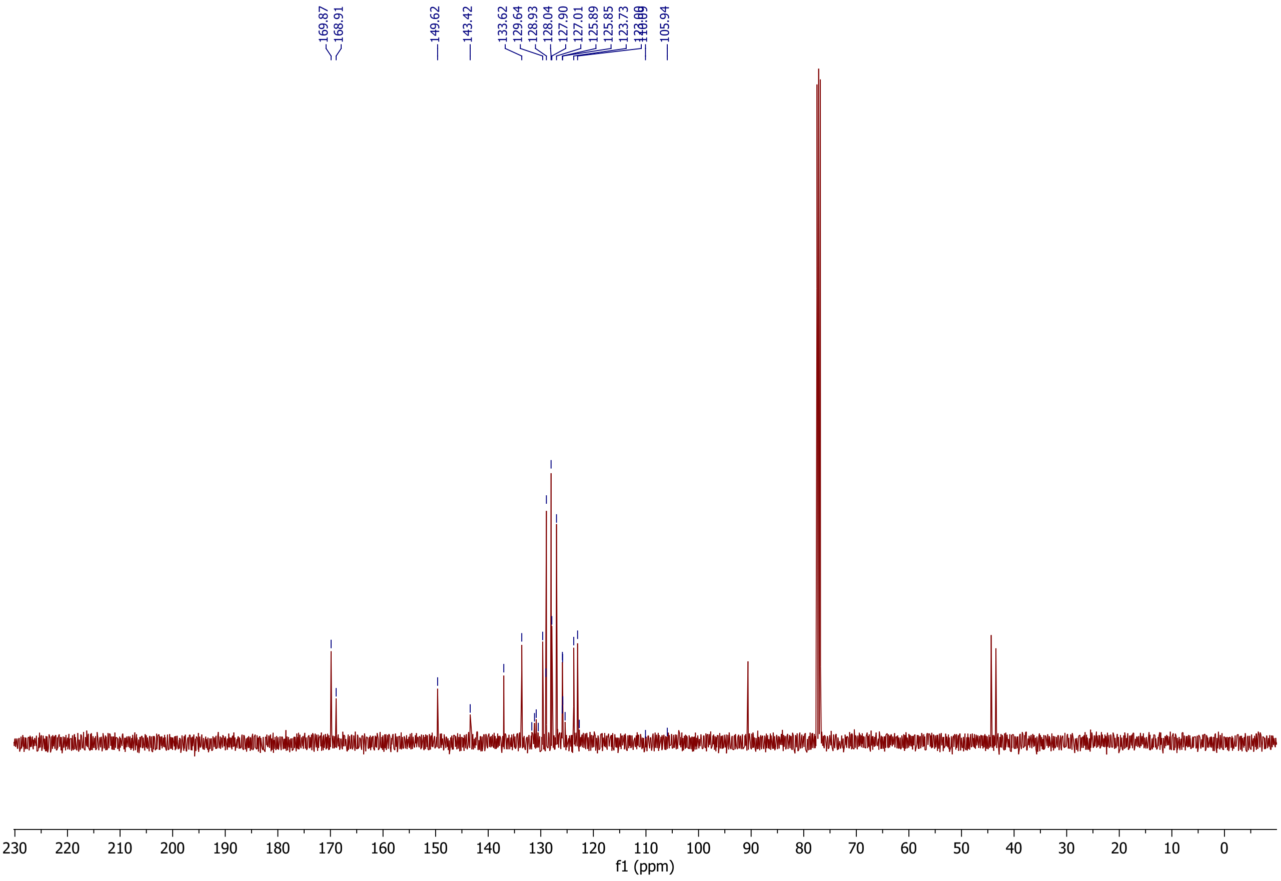

Figure S. 49. N-benzyl-2-[1-(4-bromophenyl)-1-hydroxy-3-oxo-isoindolin-2-yl]acetamide (5d)

^{1}H NMR (400 MHz, $CDCl_3$)

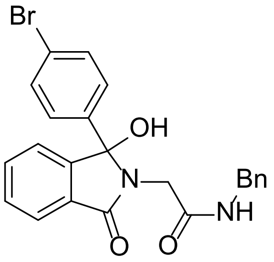

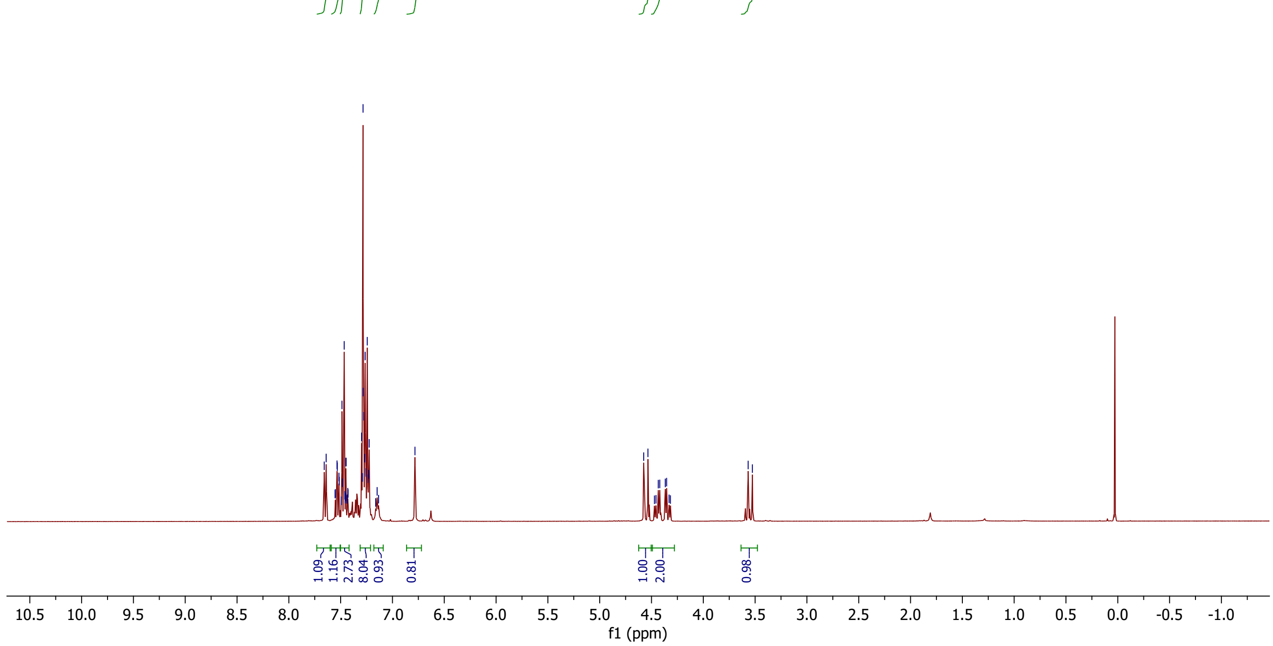

^{13}C NMR (101 MHz, $CDCl_3$)

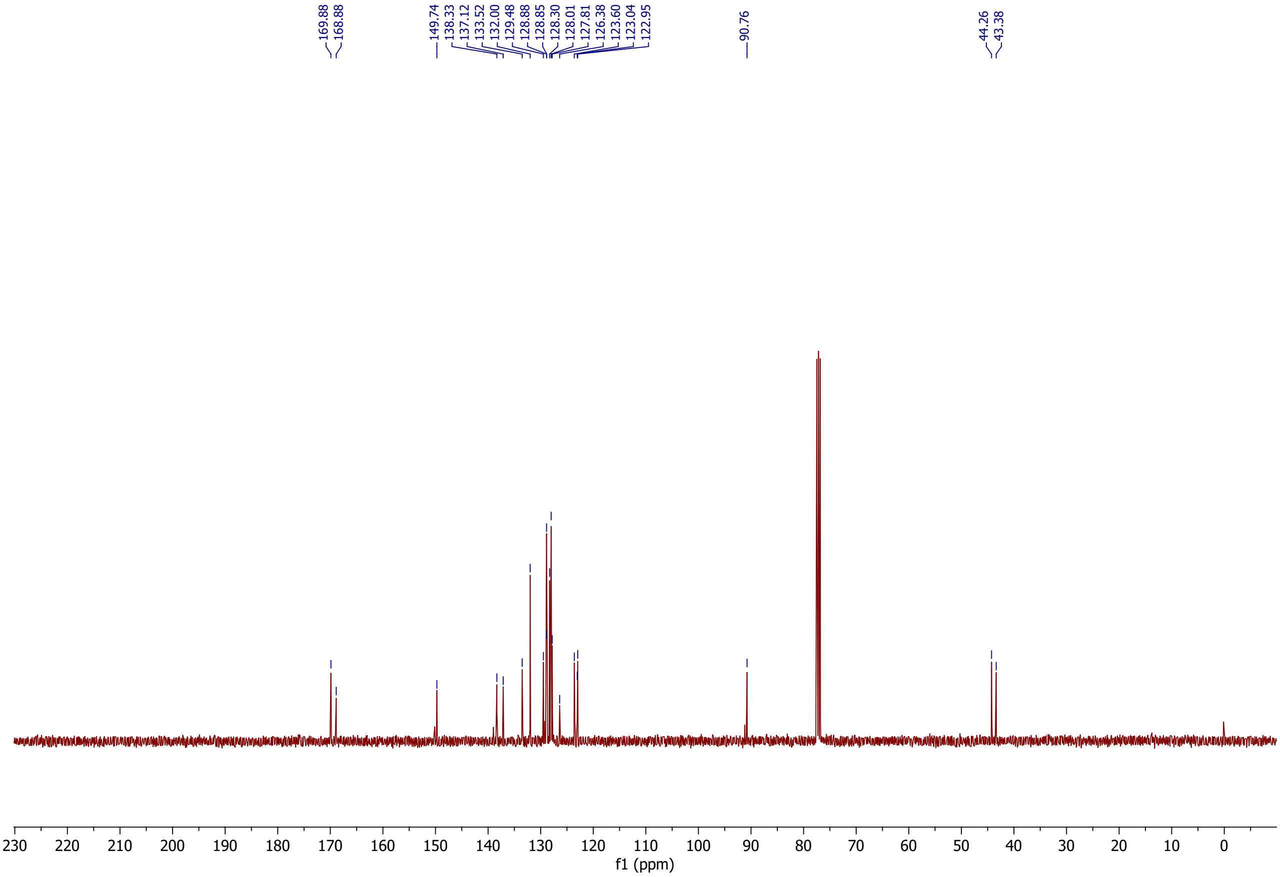

Figure S. 50. N-benzyl-2-[1-hydroxy-1-(6-methoxy-2-pyridyl)-3-oxo-isoindolin-2-yl]acetamide (5e)

^{1}H NMR (400 MHz, $CDCl_3$)

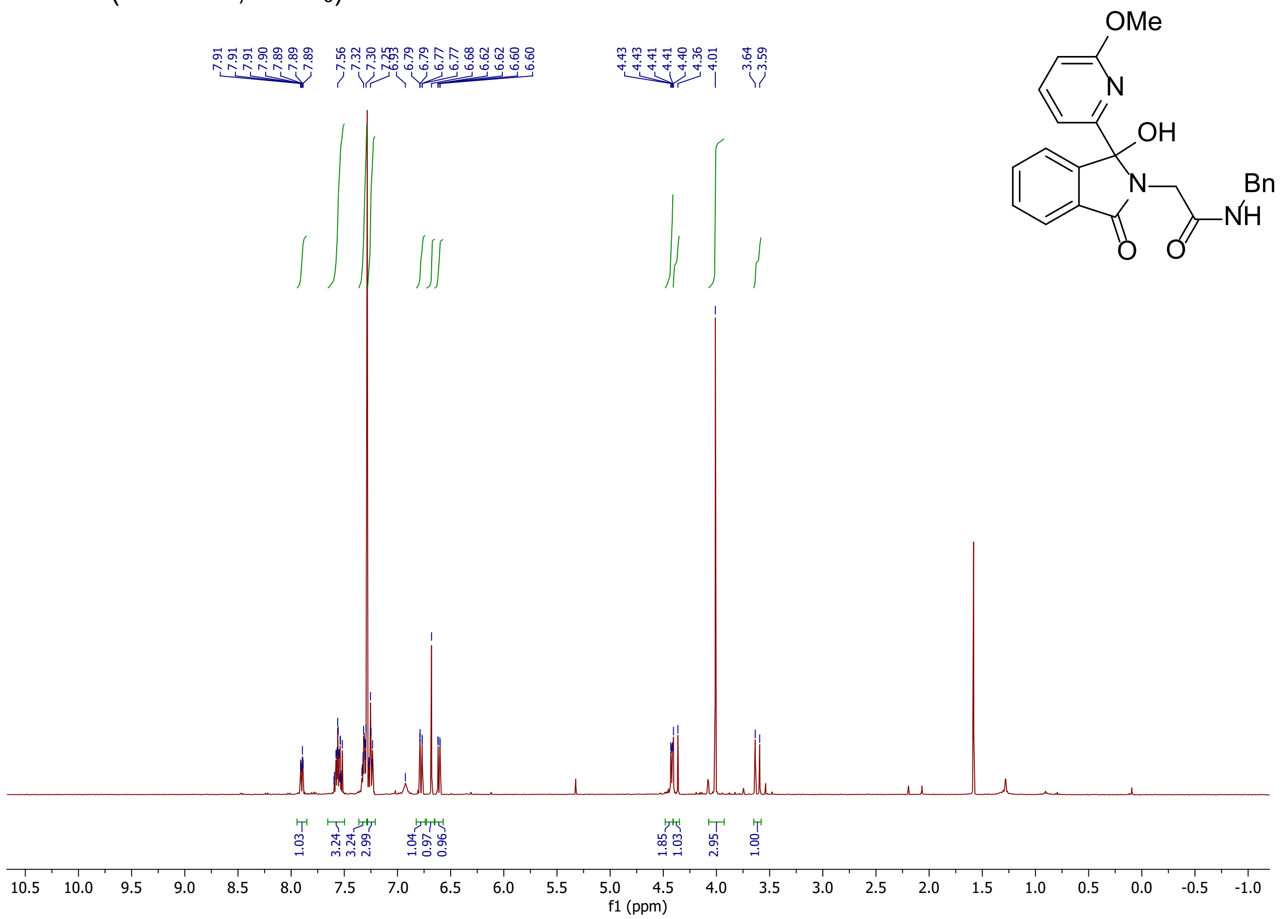

^{13}C NMR (101 MHz, $CDCl_3$)

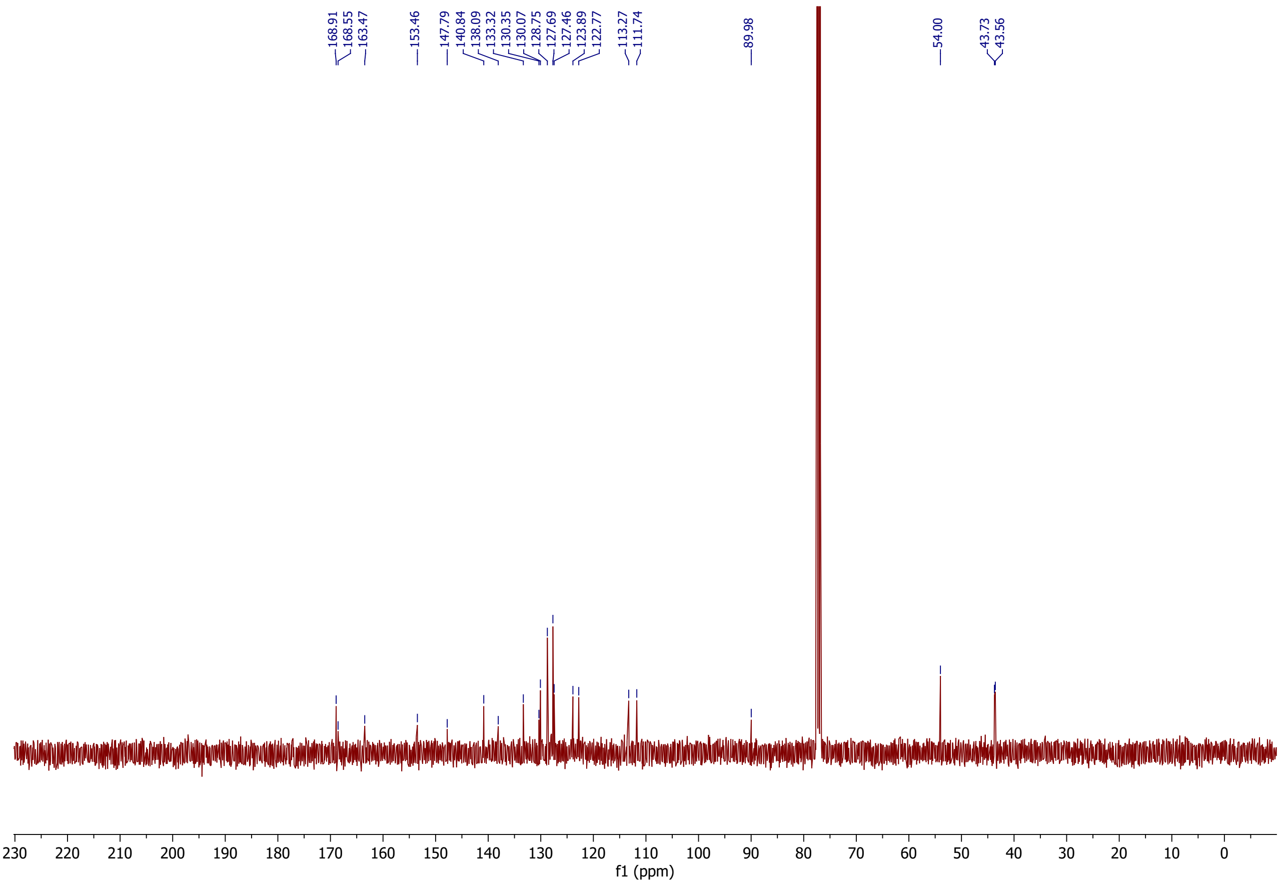

Figure S. 51. 2-[1-(1,3-benzodioxol-5-yl)-1-hydroxy-3-oxo-isoindolin-2-yl]-N-benzyl-acetamide (5f)

^{1}H NMR (400 MHz, $CDCl_3$)

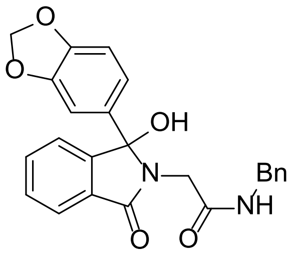

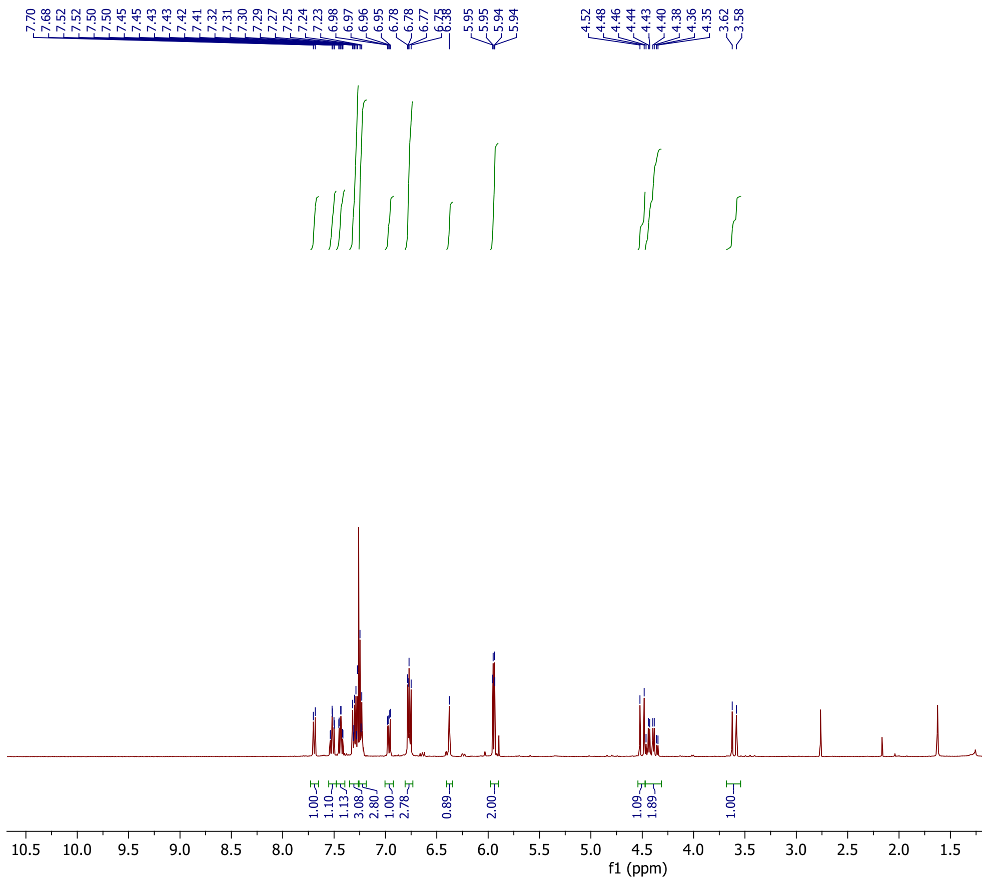

^{13}C NMR (101 MHz, $CDCl_3$)

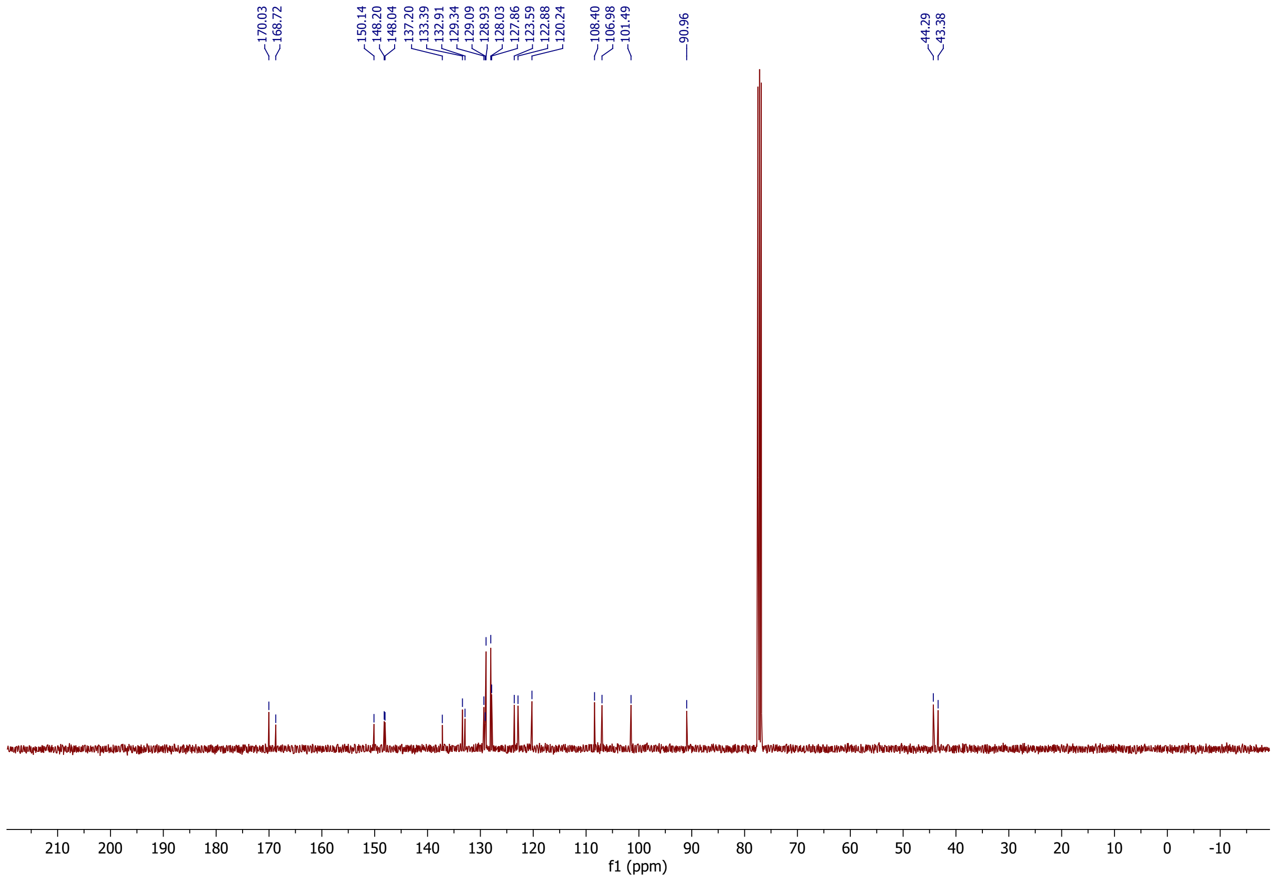

Figure S. 52. N-[(3-chlorophenyl)methyl]-2-(1-hydroxy-3-oxo-1-phenyl-isoindolin-2-yl)acetamide (5g)

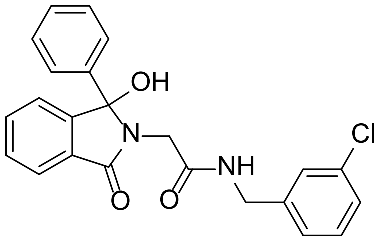

^{1}H NMR (400 MHz, $CDCl_3$)

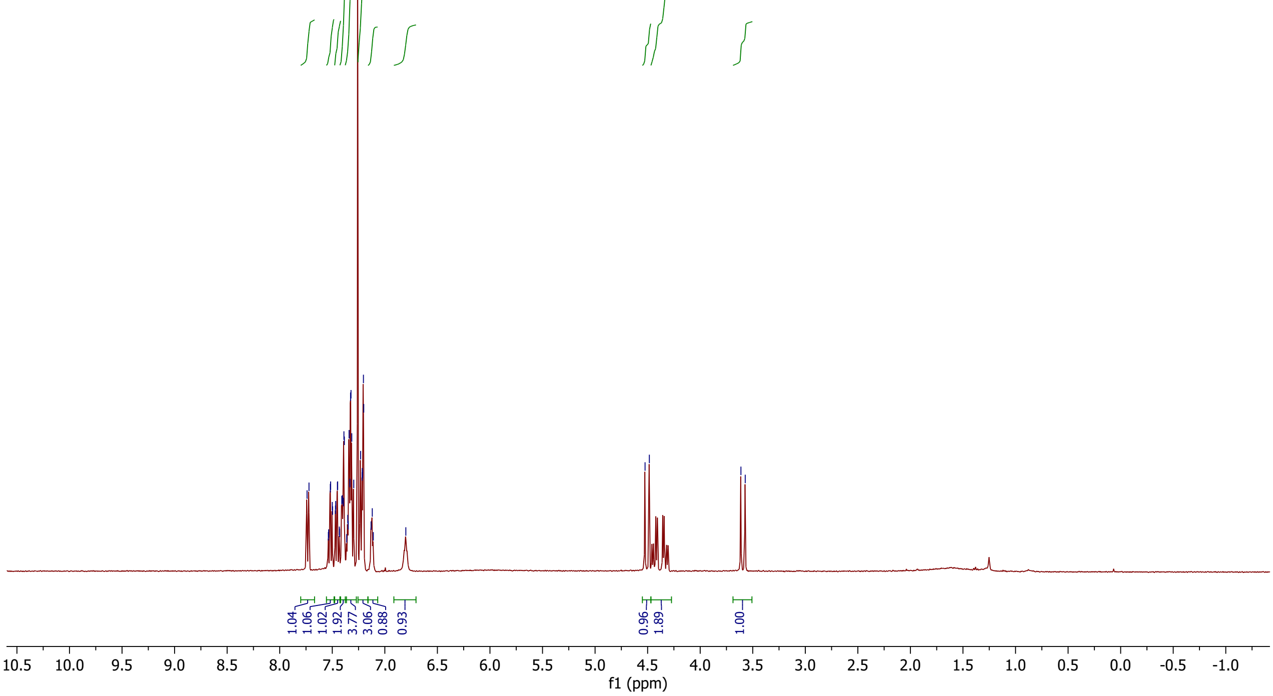

^{13}C NMR (101 MHz, $CDCl_3$)

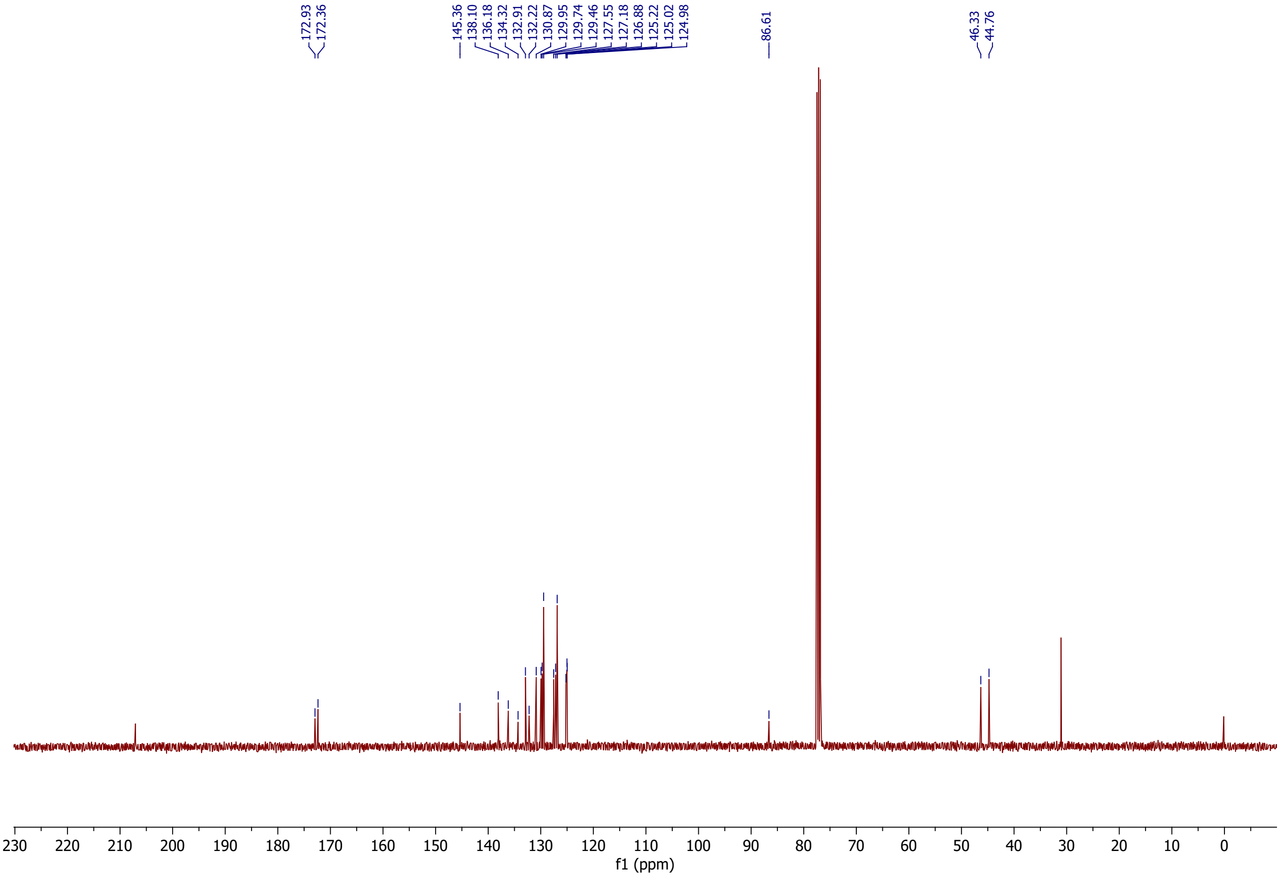

Figure S. 53. N-(4-fluorophenyl)-2-(1-hydroxy-3-oxo-1-phenyl-isoindolin-2-yl)acetamide (5h)

^{1}H NMR (400 MHz, $CDCl_3$)

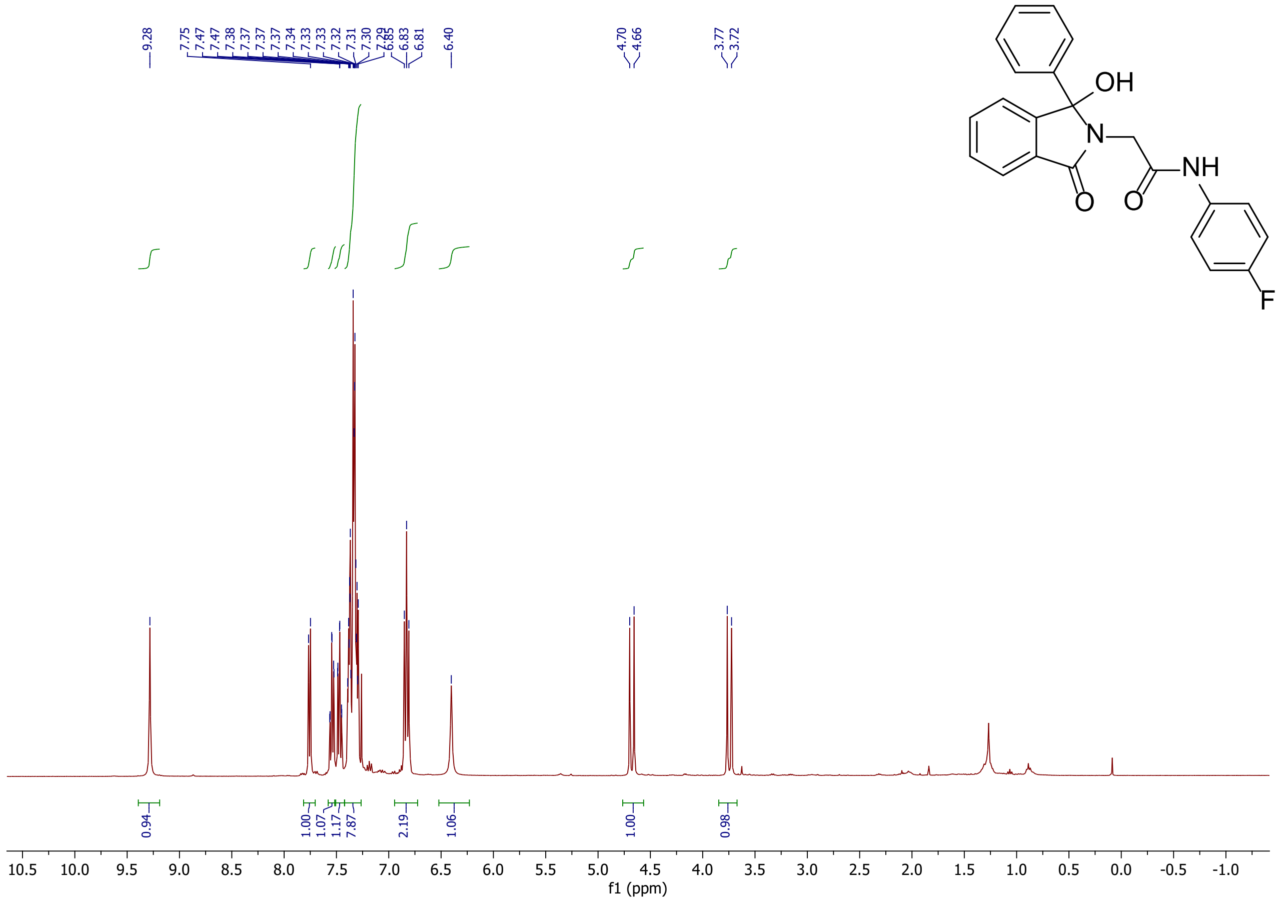

^{13}C NMR (101 MHz, $CDCl_3$)

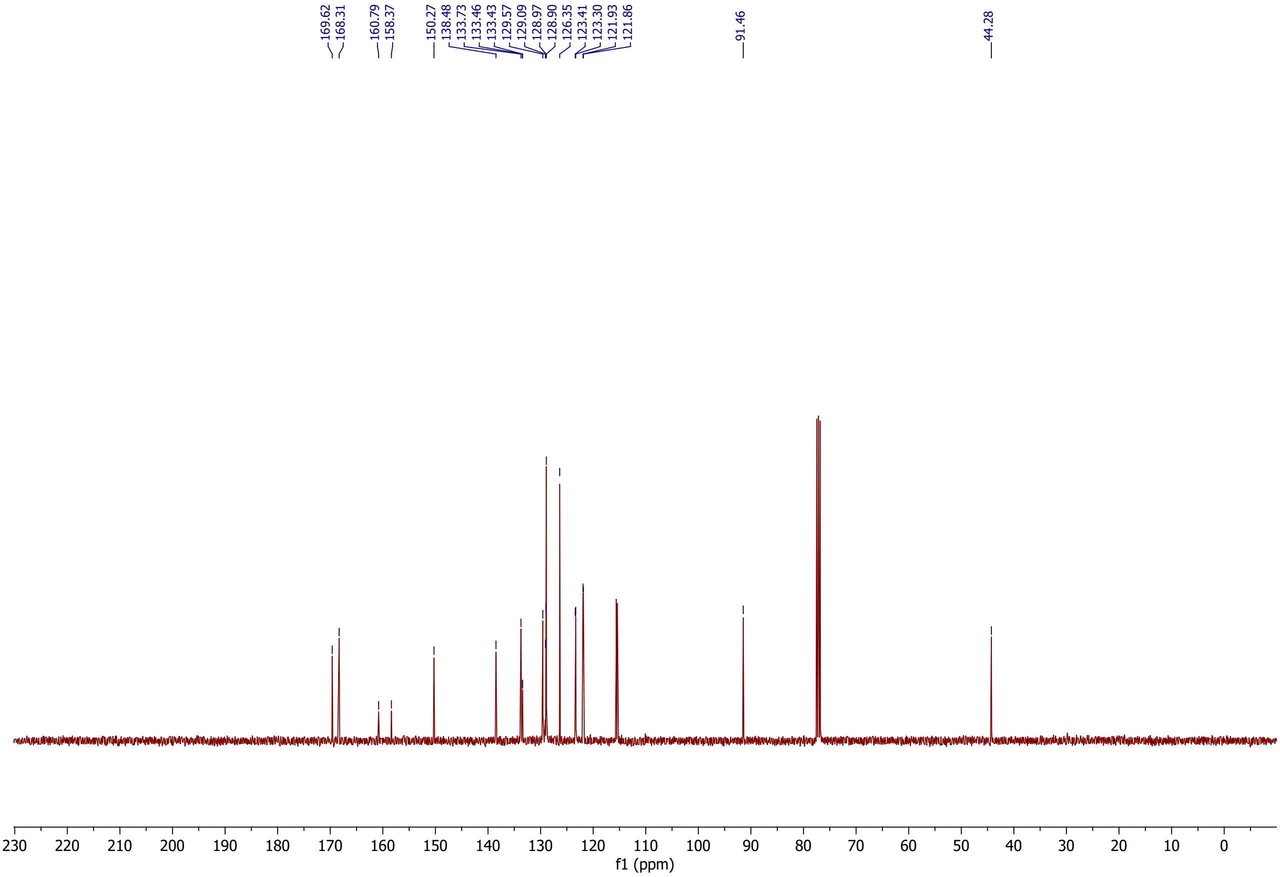

Figure S. 54. N-(2-chloro-4-methyl-phenyl)-2-(1-hydroxy-3-oxo-1-phenyl-isoindolin-2-yl)acetamide (5i)

^{1}H NMR (400 MHz, $CDCl_3$)

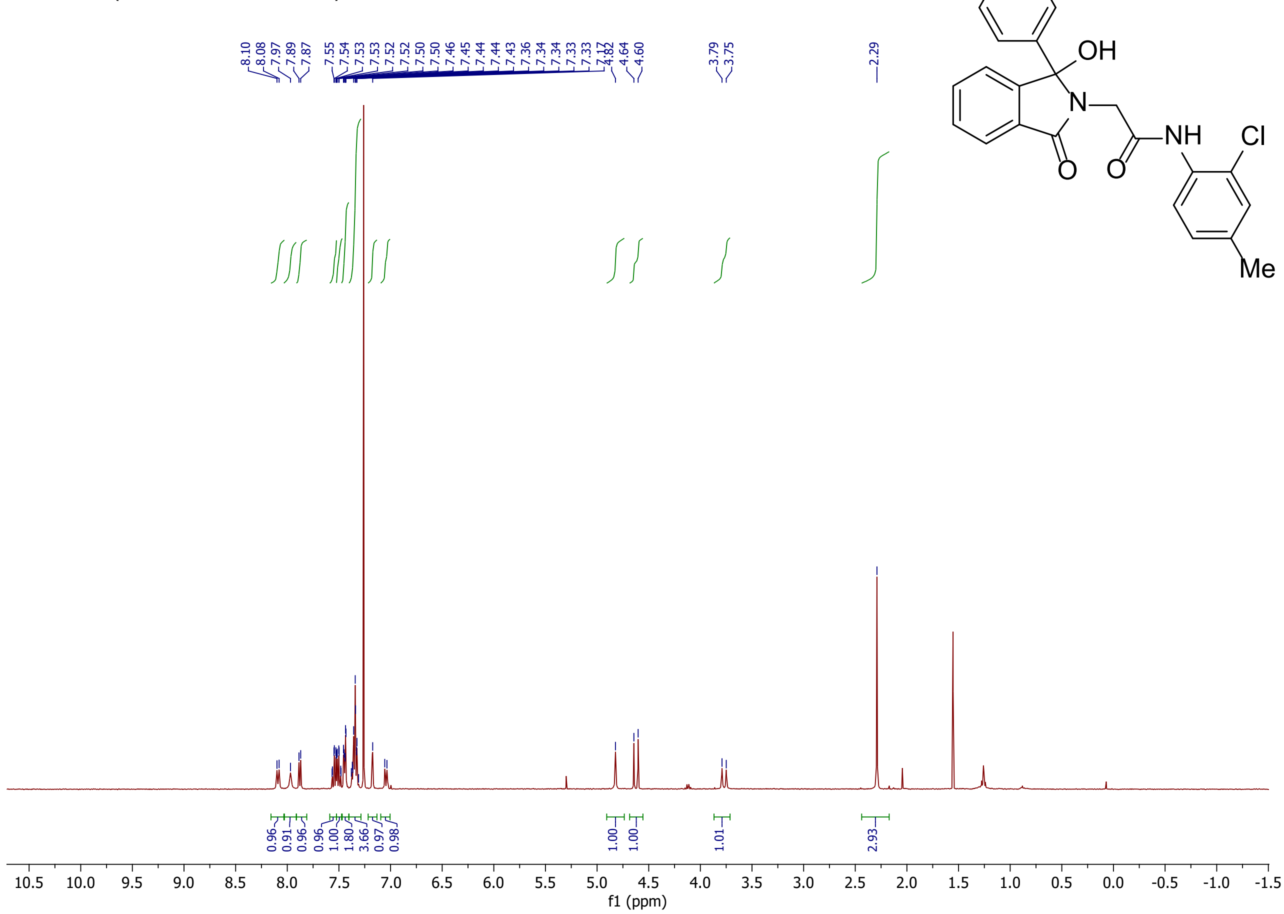

^{13}C NMR (101 MHz, $CDCl_3$)

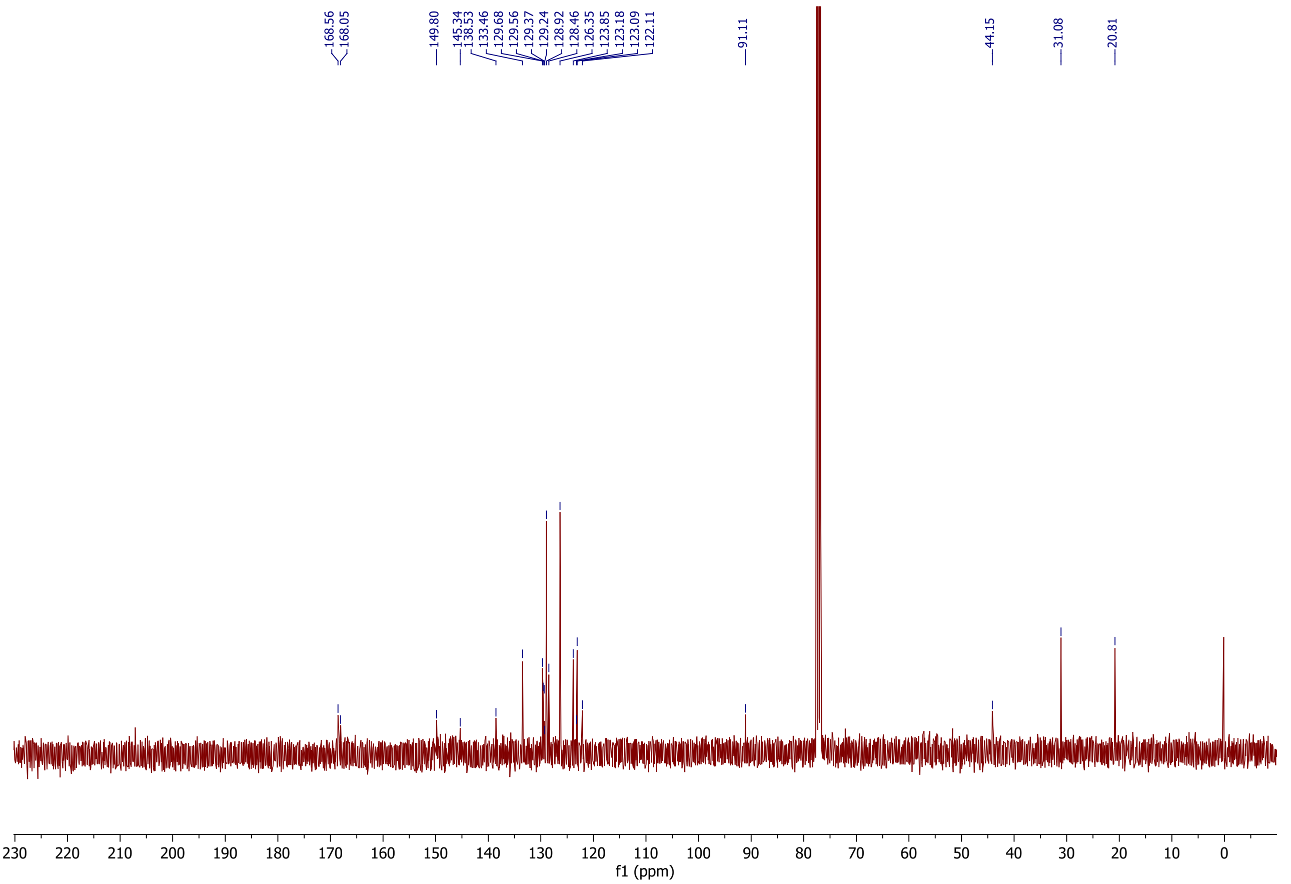

Figure S. 55. N-cyclopropyl-2-(1-hydroxy-3-oxo-1-phenyl-isoindolin-2-yl)acetamide (5j)
^{1}H NMR (400 MHz, $CDCl_3$)

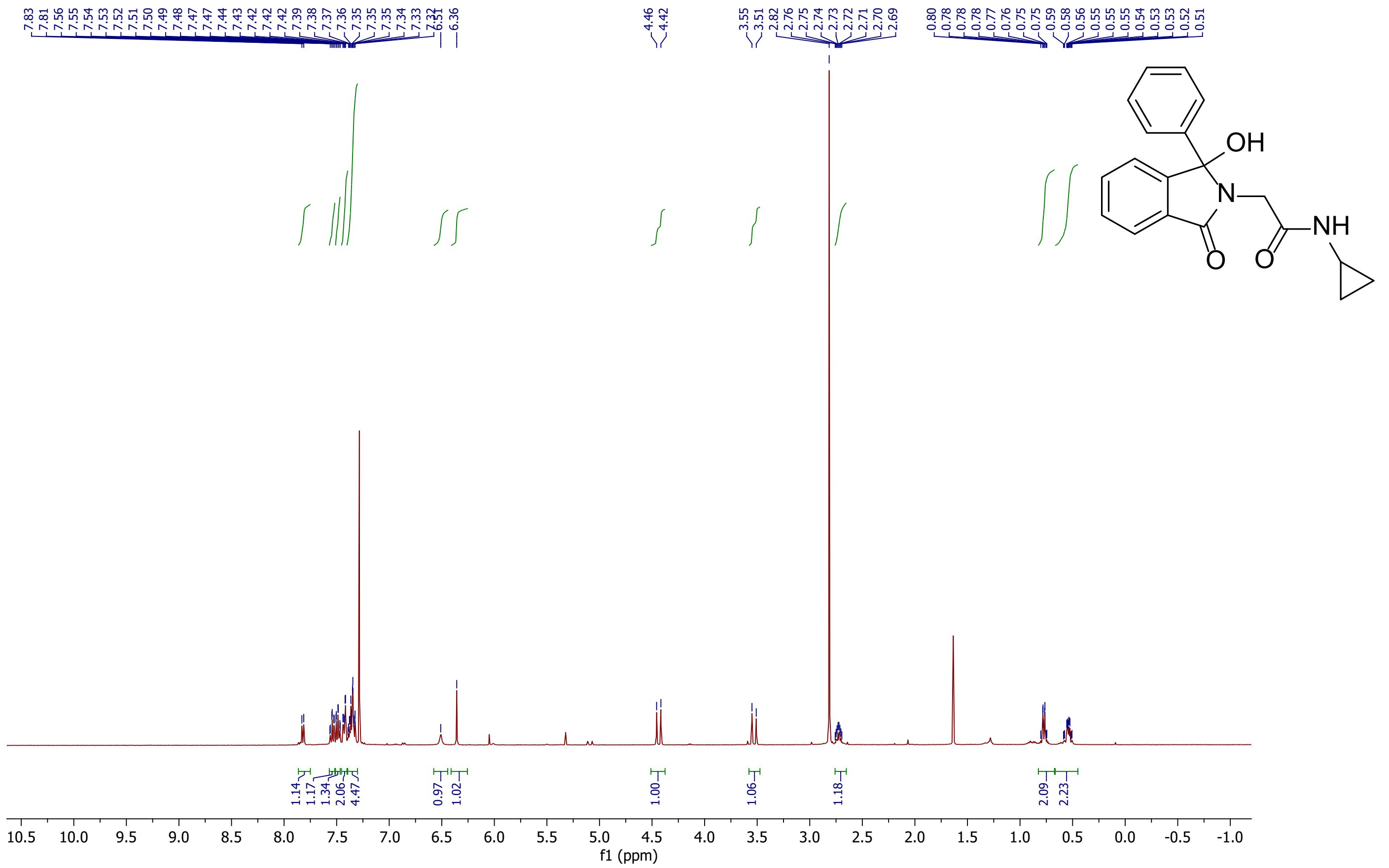

^{13}C NMR (101 MHz, $CDCl_3$)

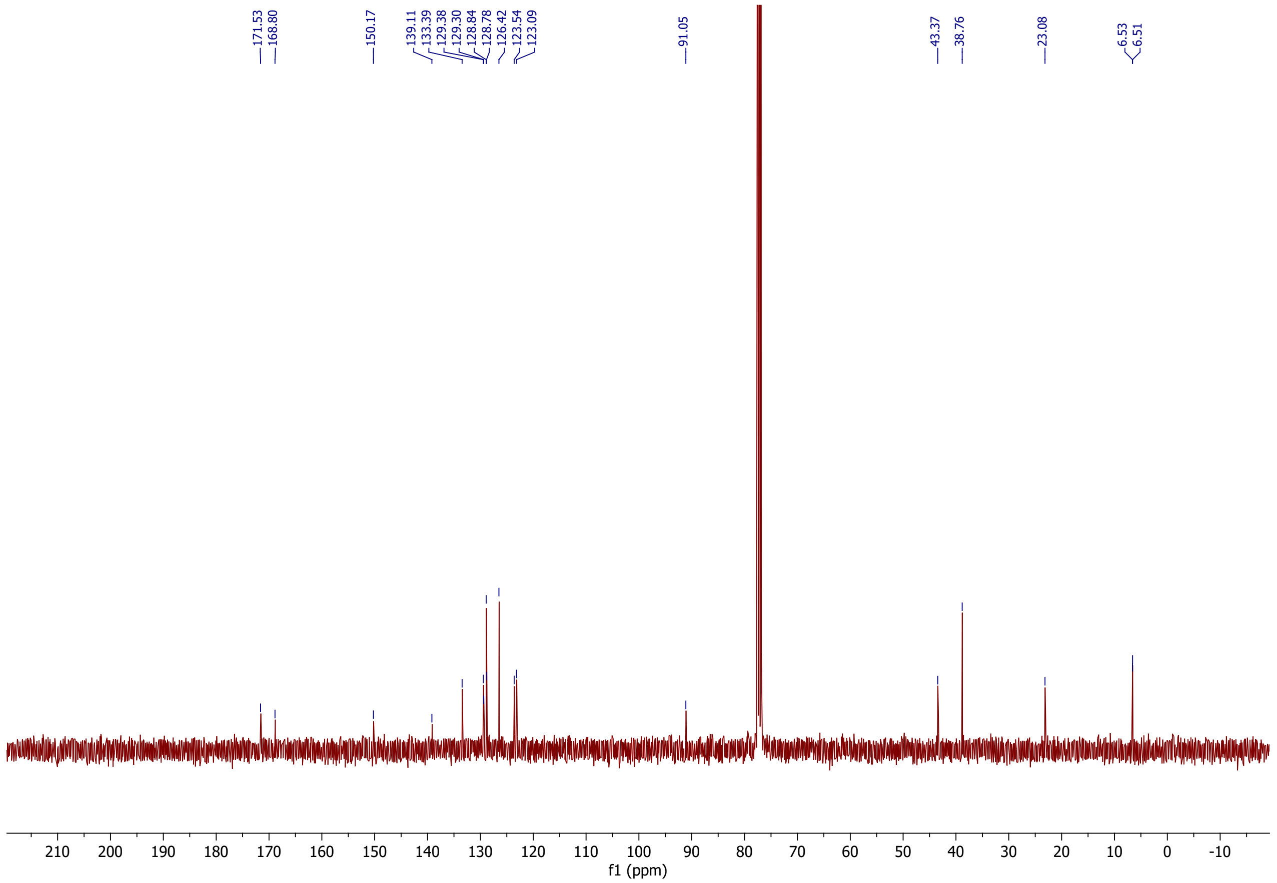

Figure S. 56. 2-(1-hydroxy-3-oxo-1-phenyl-isoindolin-2-yl)-N-isobutyl-acetamide (5k)

^{1}H NMR (400 MHz, $CDCl_3$)

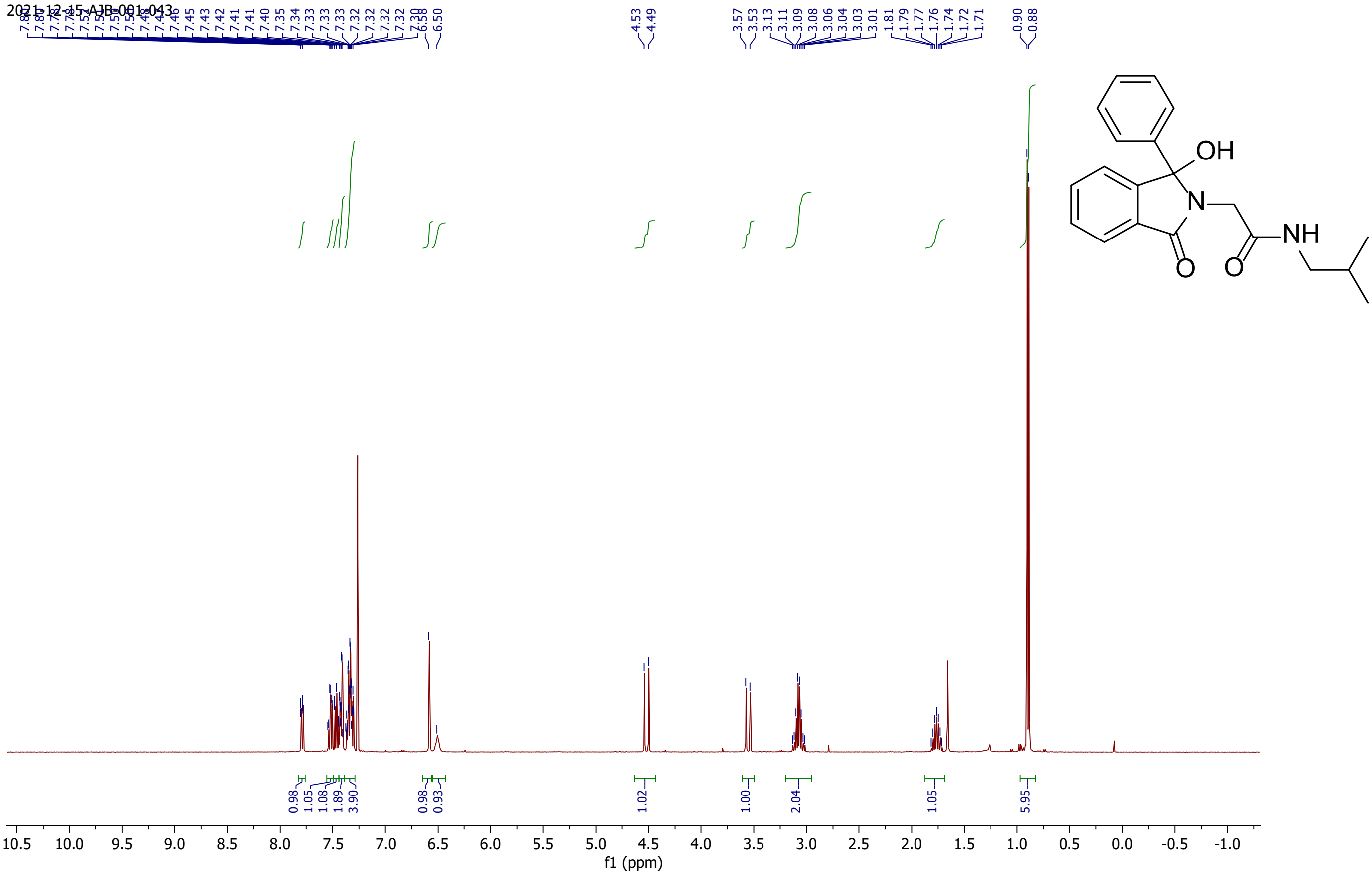

^{13}C NMR (101 MHz, $CDCl_3$)

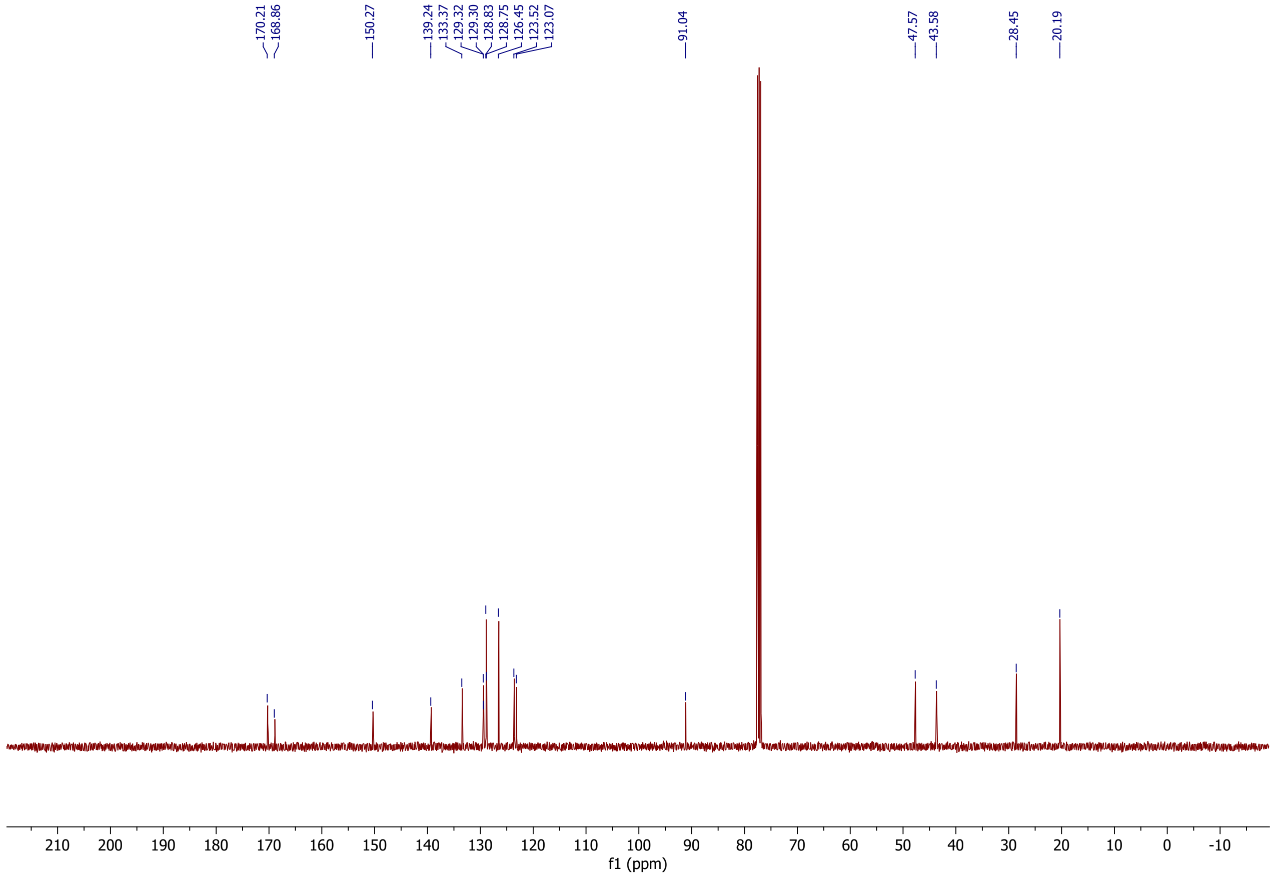

Figure S. 57. 1-benzyl-9b-phenyl-3H-imidazo[2,1-a]isoindole-2,5-dione (6a)

^{1}H NMR (400 MHz, $CDCl_3$)

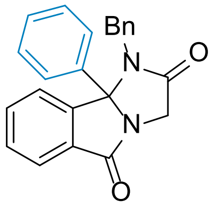

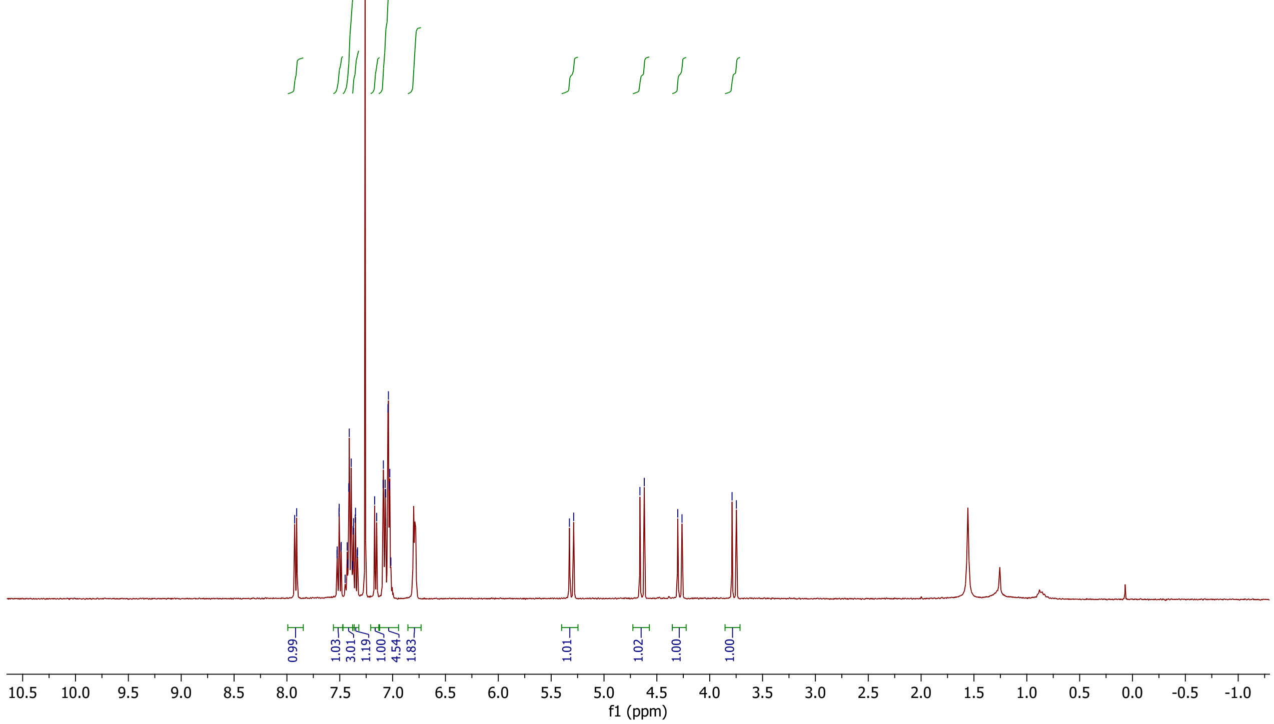

^{13}C NMR (101 MHz, $CDCl_3$)

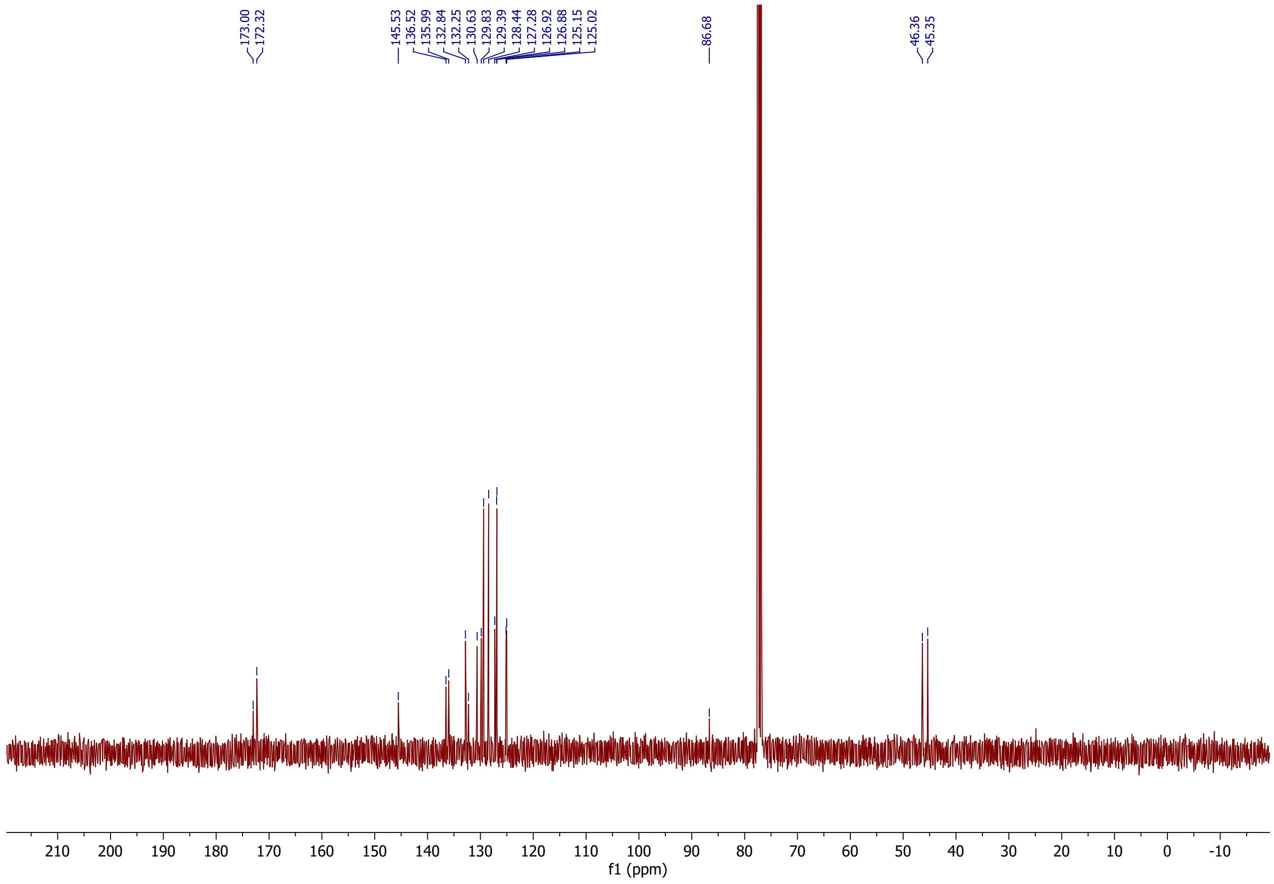

Figure S. 58. 1-benzyl-9b-(4-methoxyphenyl)-3H-imidazo[2,1-a]isoindole-2,5-dione (6b)

^{1}H NMR (400 MHz, CDCl$_3$)

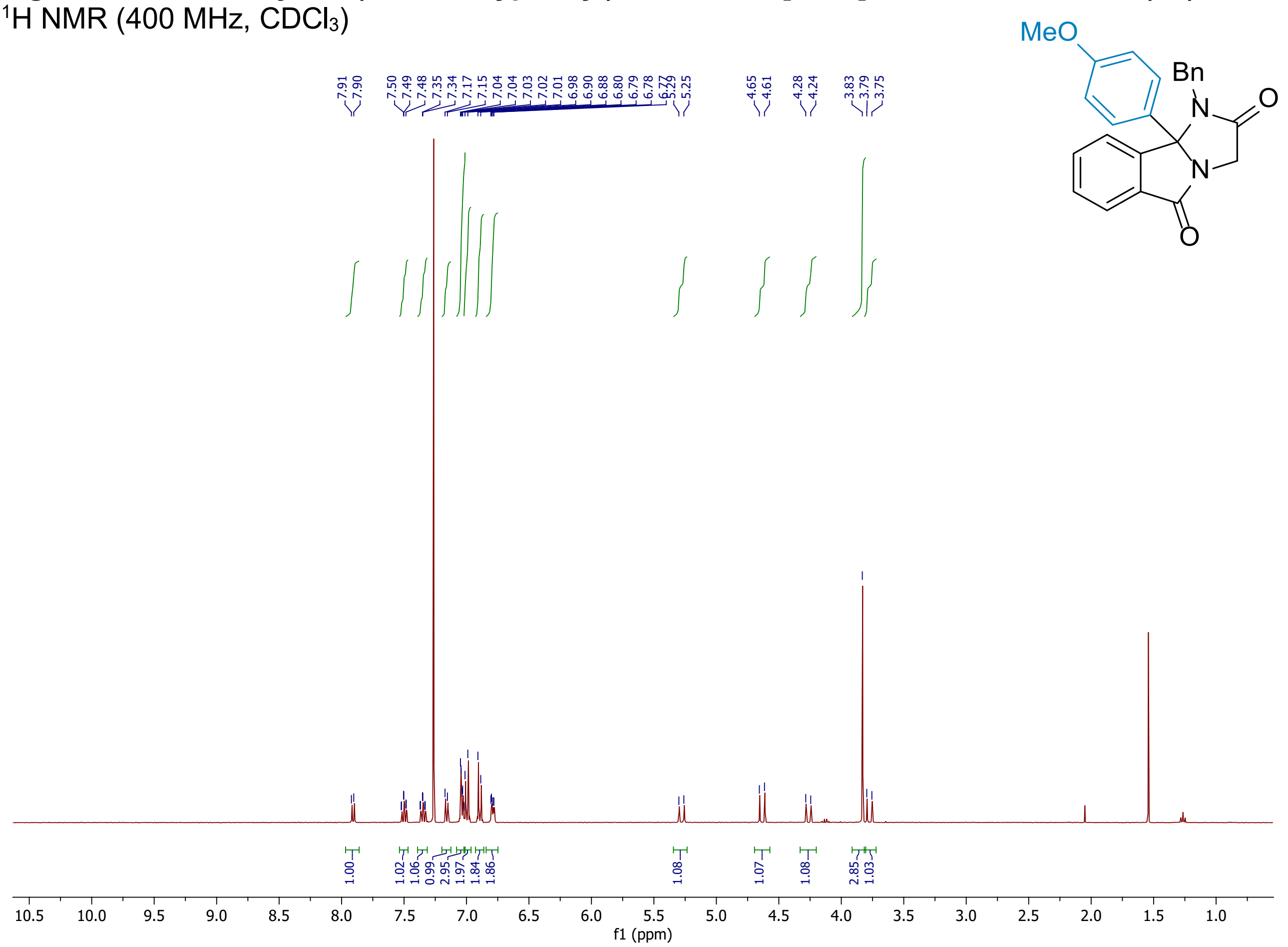

^{13}C NMR (101 MHz, CDCl$_3$)

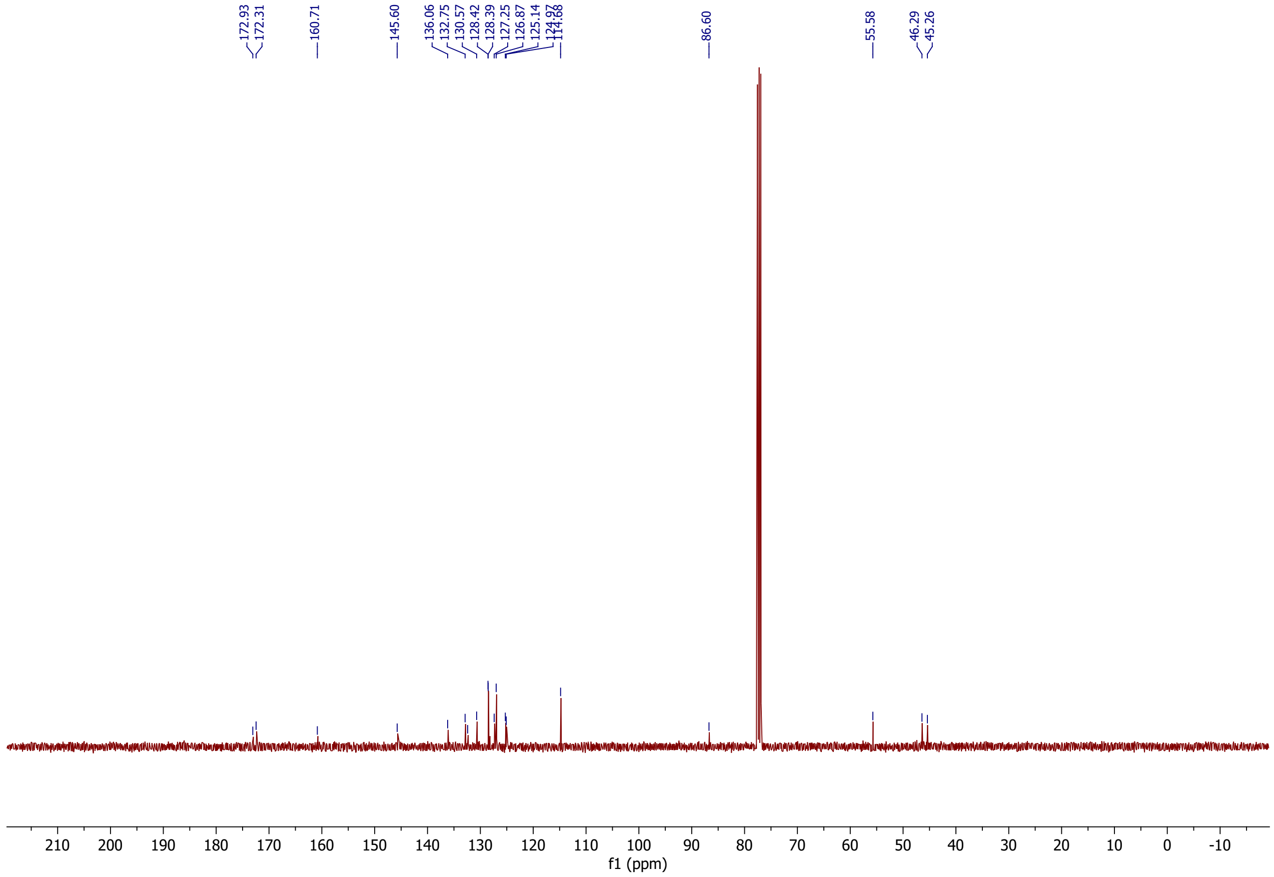

Figure S. 59. 1-benzyl-9b-[4-(trifluoromethyl)phenyl]-3H-imidazo[2,1-a]isoindole-2,5-dione (6c)

^{1}H NMR (400 MHz, $CDCl_3$)

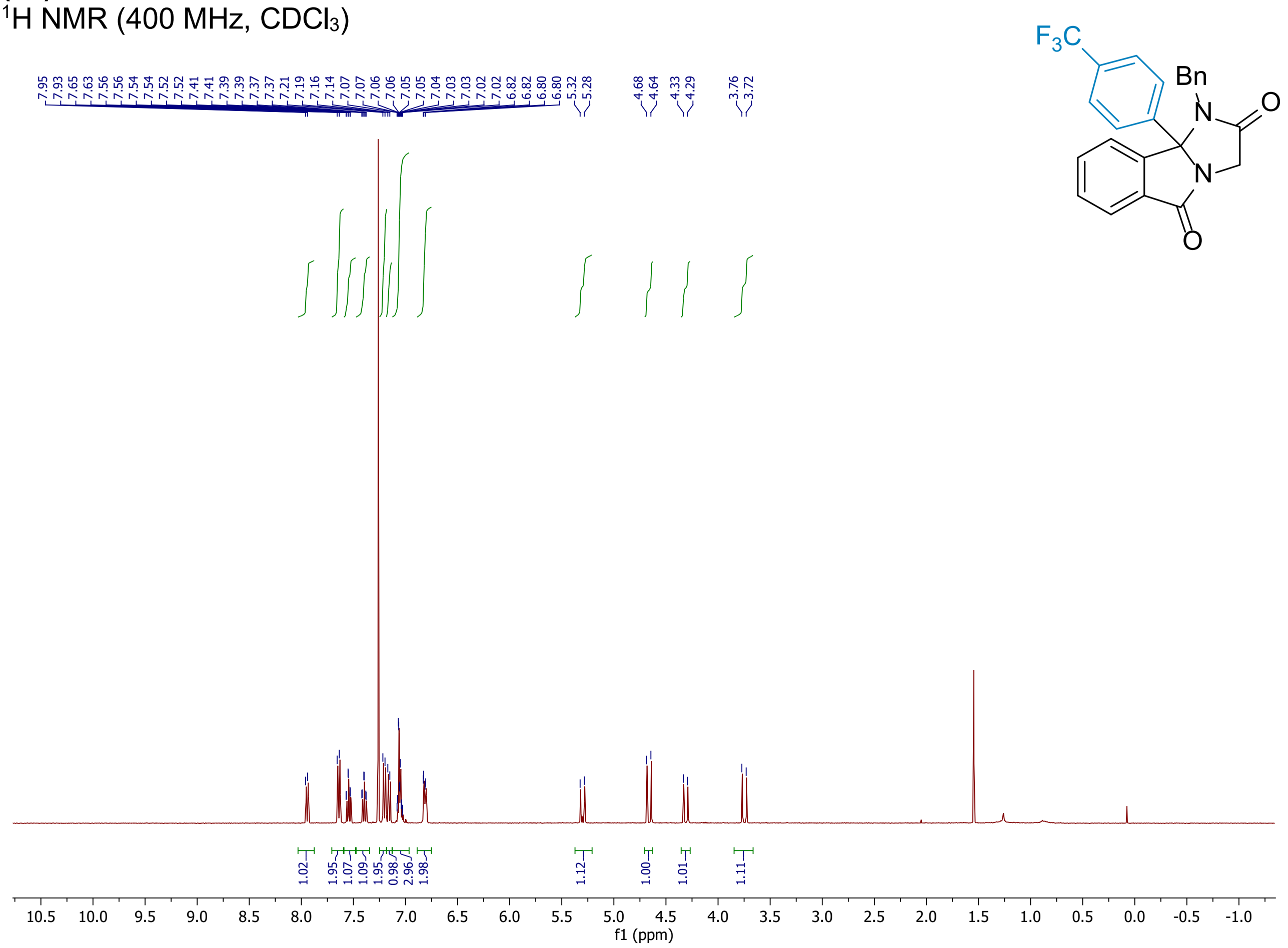

^{13}C NMR (101 MHz, $CDCl_3$)

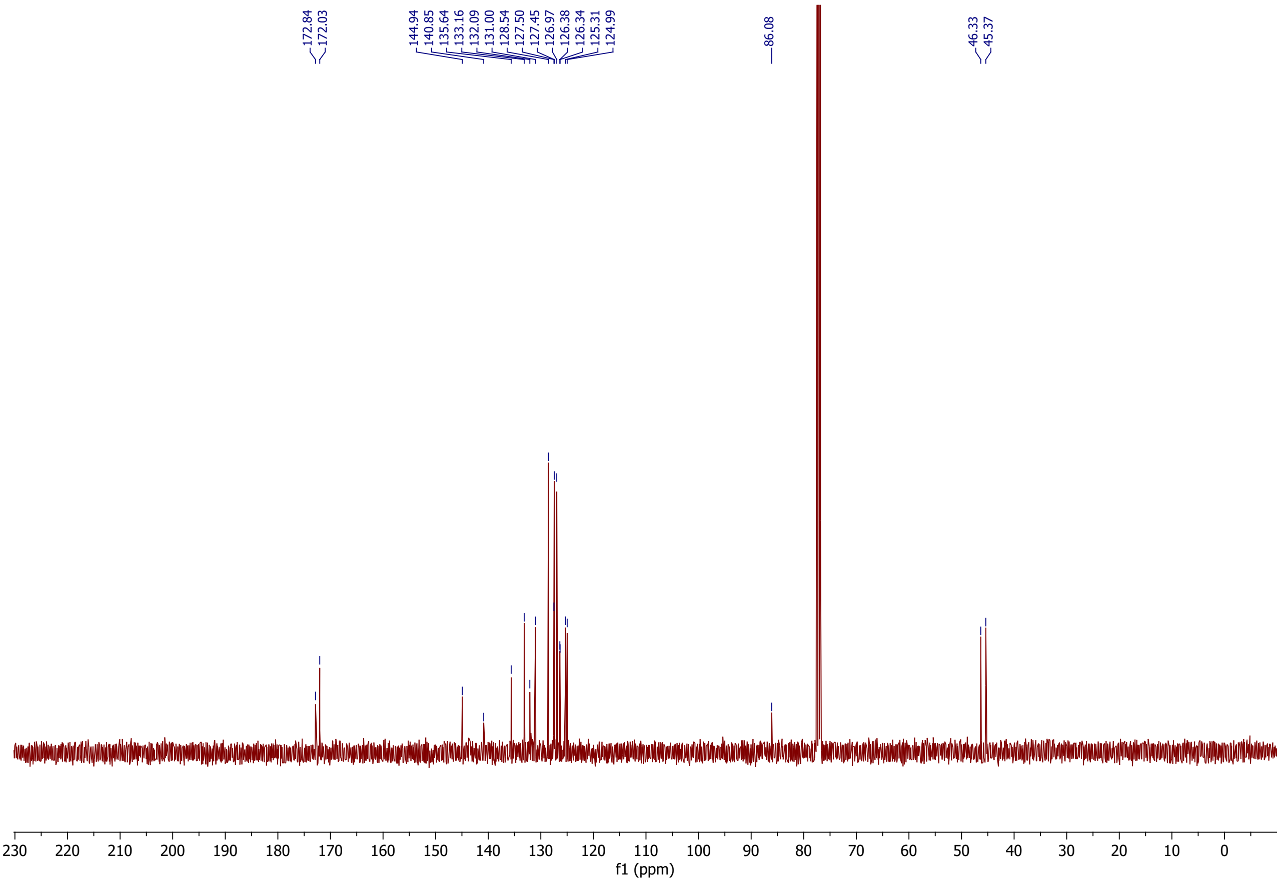

Figure S. 60. 1-benzyl-9b-(4-bromophenyl)-3H-imidazo[2,1-a]isoindole-2,5-dione (6d)

^1H NMR (400 MHz, $CDCl_3$)

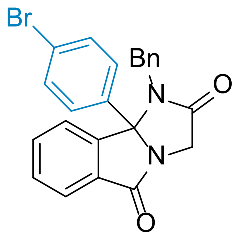

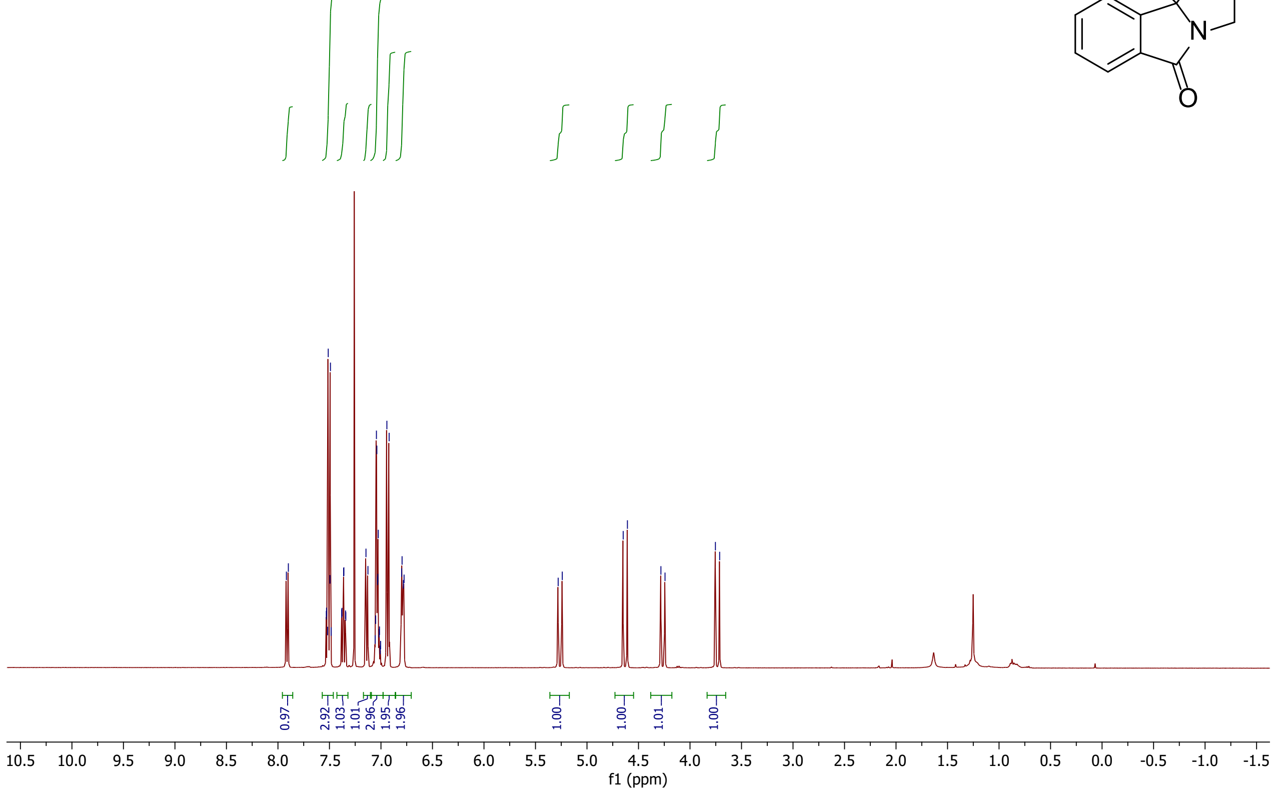

^{13}C NMR (101 MHz, $CDCl_3$)

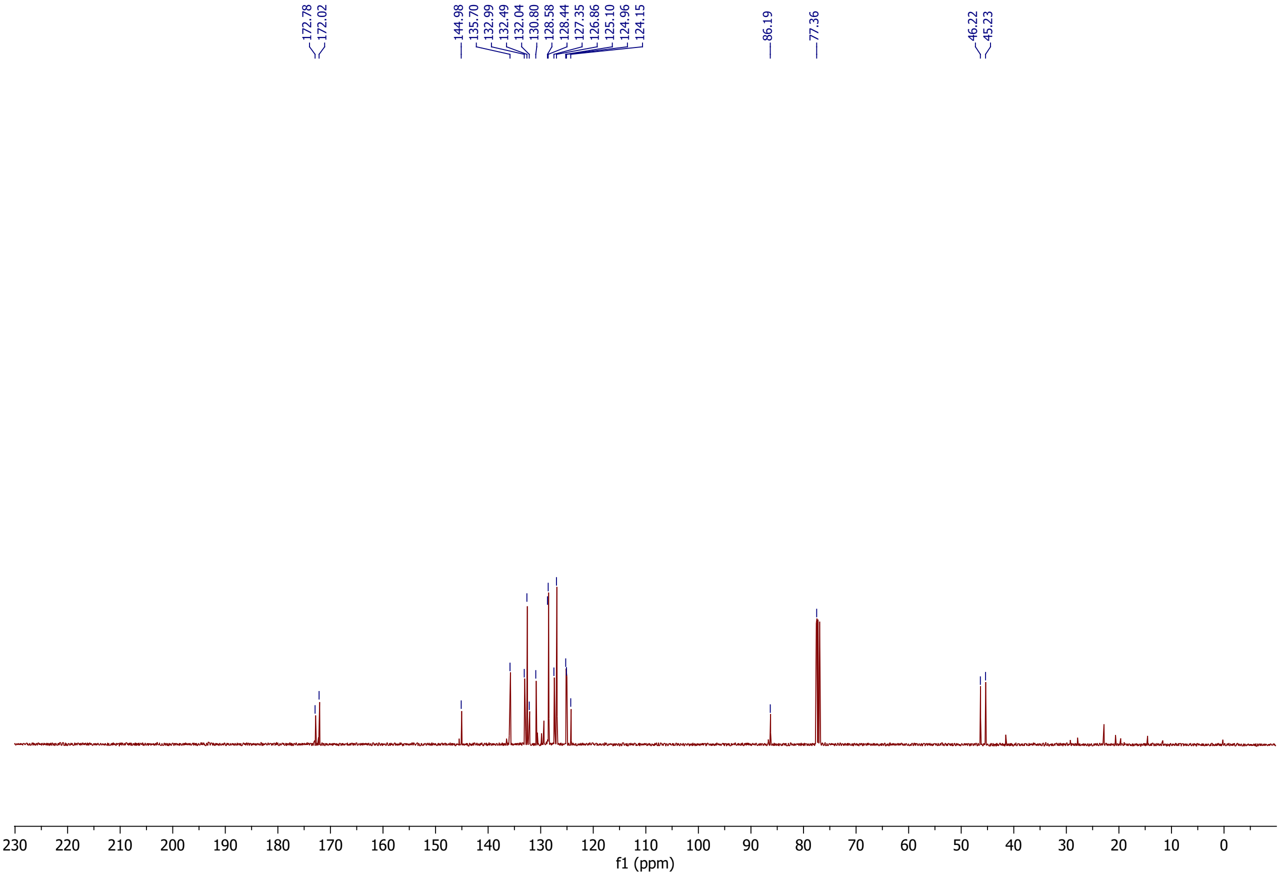

Figure S. 61. 1-benzyl-9b-(6-methoxy-2-pyridyl)-3H-imidazo[2,1-a]isoindole-2,5-dione (6e)

^{1}H NMR (400 MHz, $CDCl_3$)

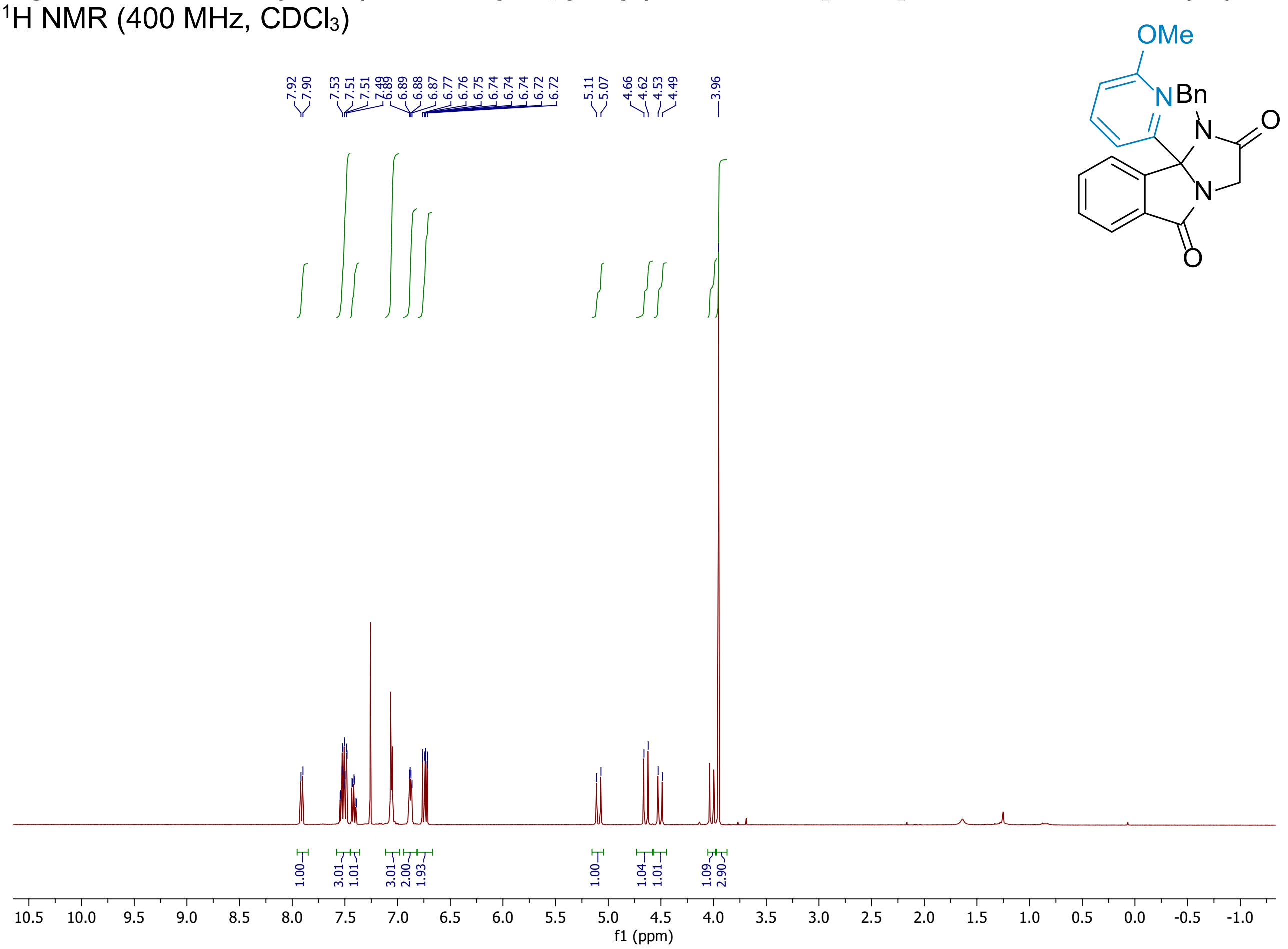

^{13}C NMR (101 MHz, $CDCl_3$)

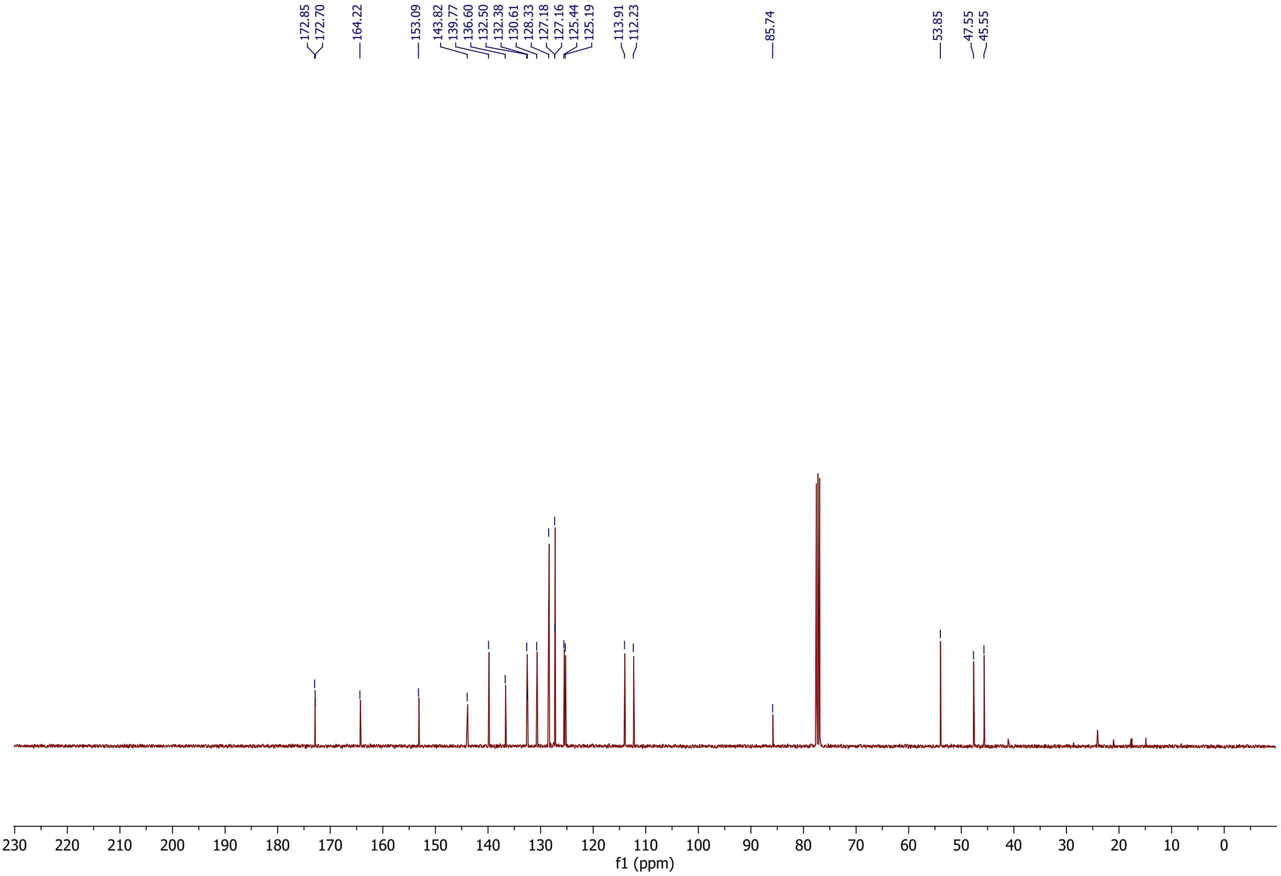

Figure S. 62. 9b-(1,3-benzodioxol-5-yl)-1-benzyl-3H-imidazo[2,1-a]isoindole-2,5-dione (6f)

^{1}H NMR (400 MHz, $CDCl_3$)

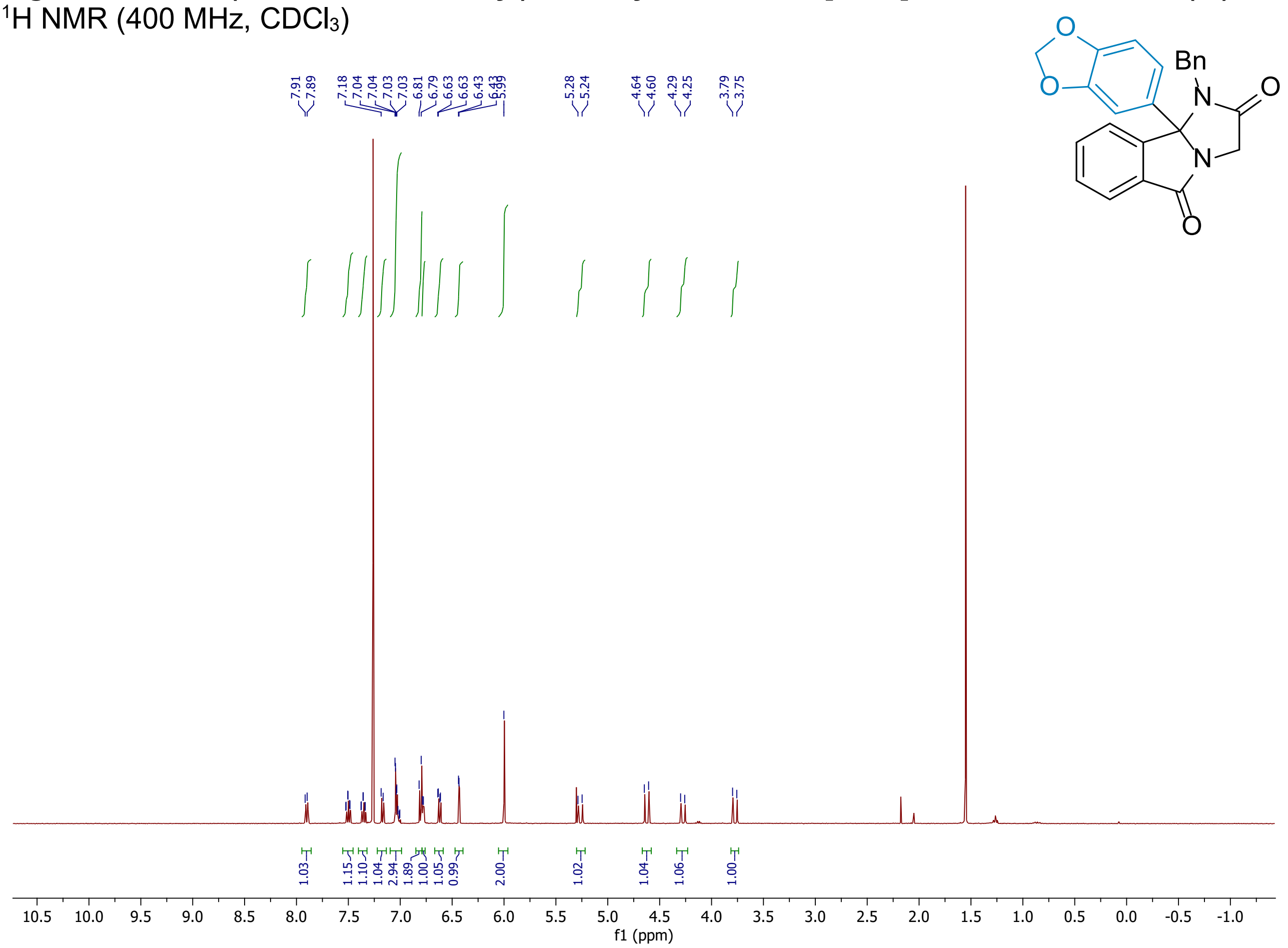

^{13}C NMR (101 MHz, $CDCl_3$)

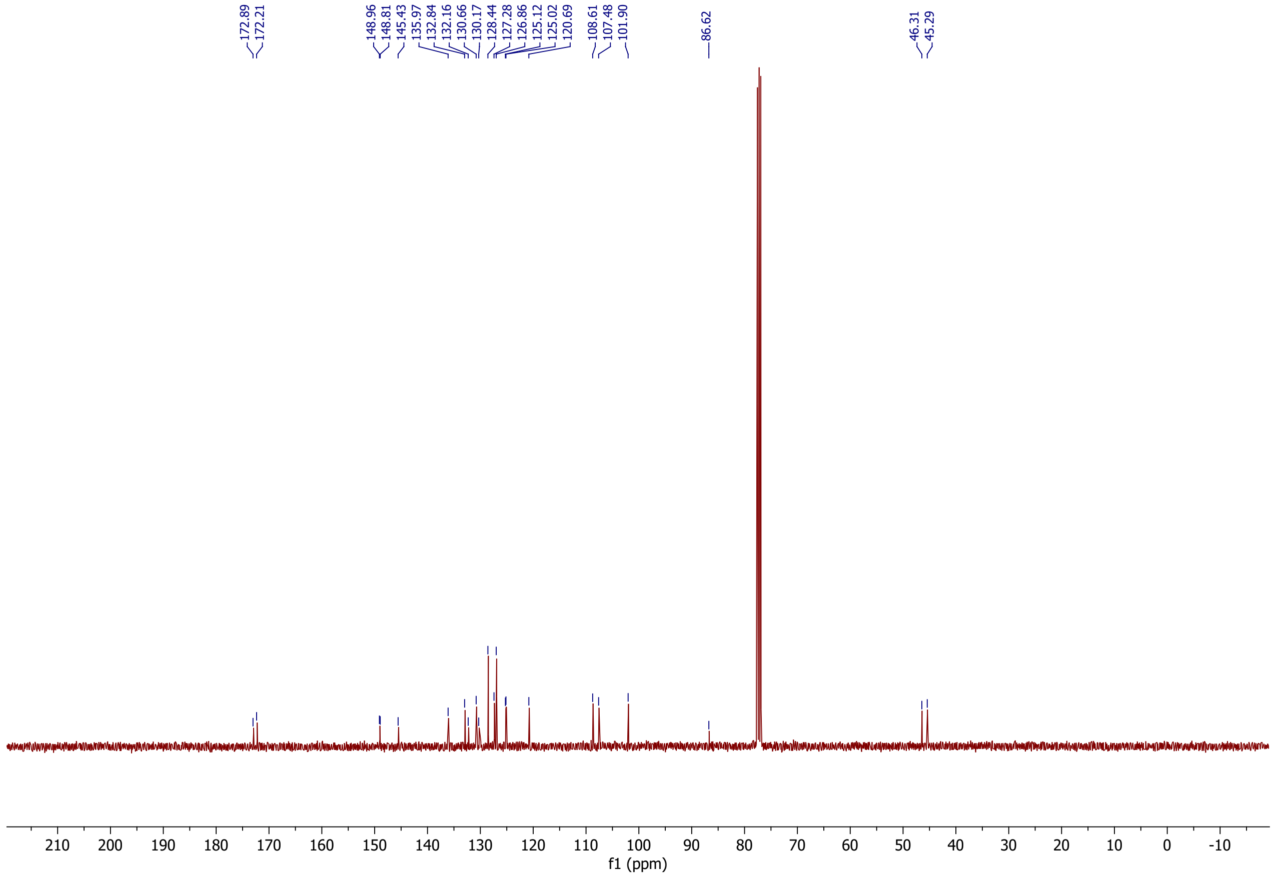

Figure S. 63. 1-[(3-chlorophenyl)methyl]-9b-phenyl-3H-imidazo[2,1-a]isoindole-2,5-dione (6g)

^{1}H NMR (400 MHz, $CDCl_3$)

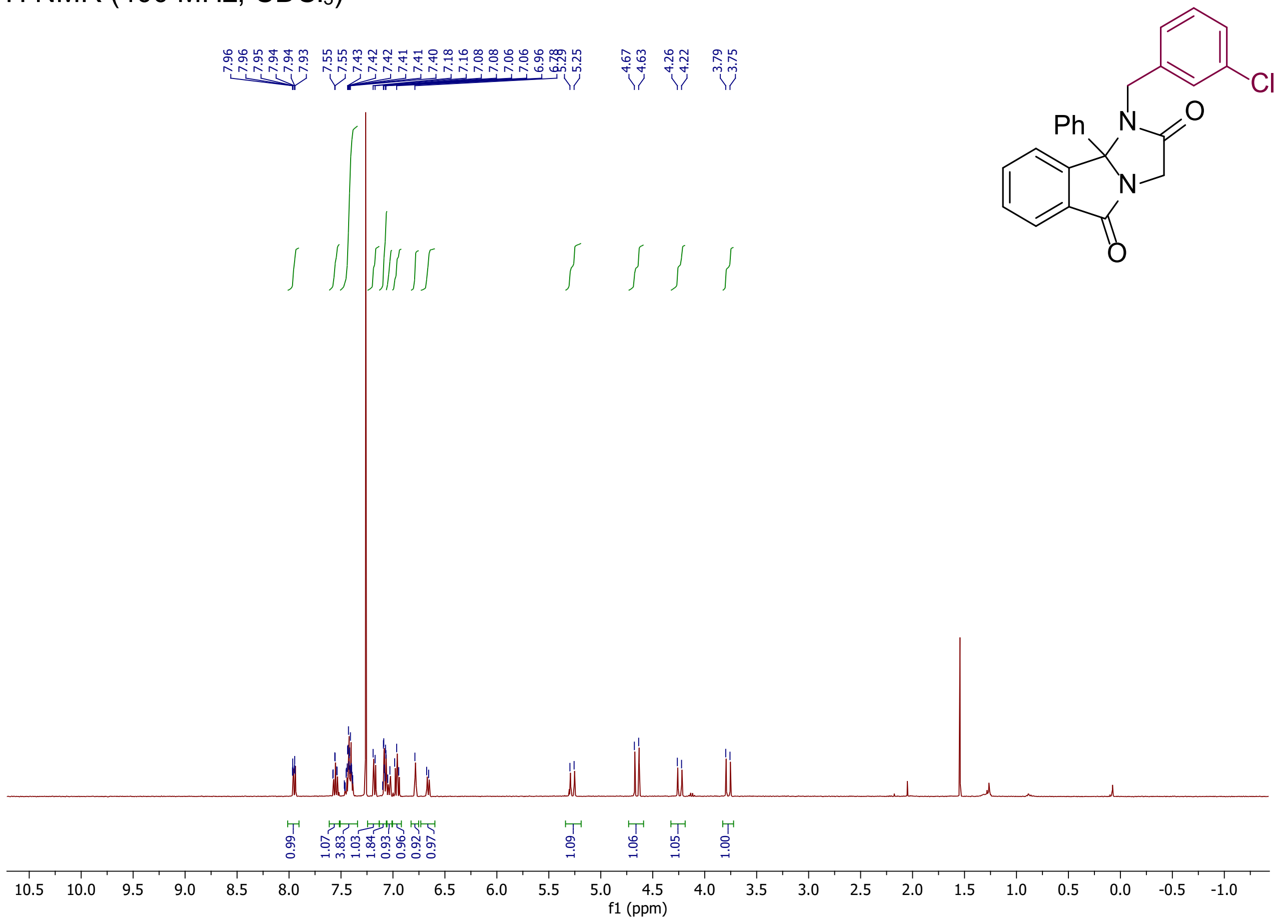

^{13}C NMR (101 MHz, $CDCl_3$)

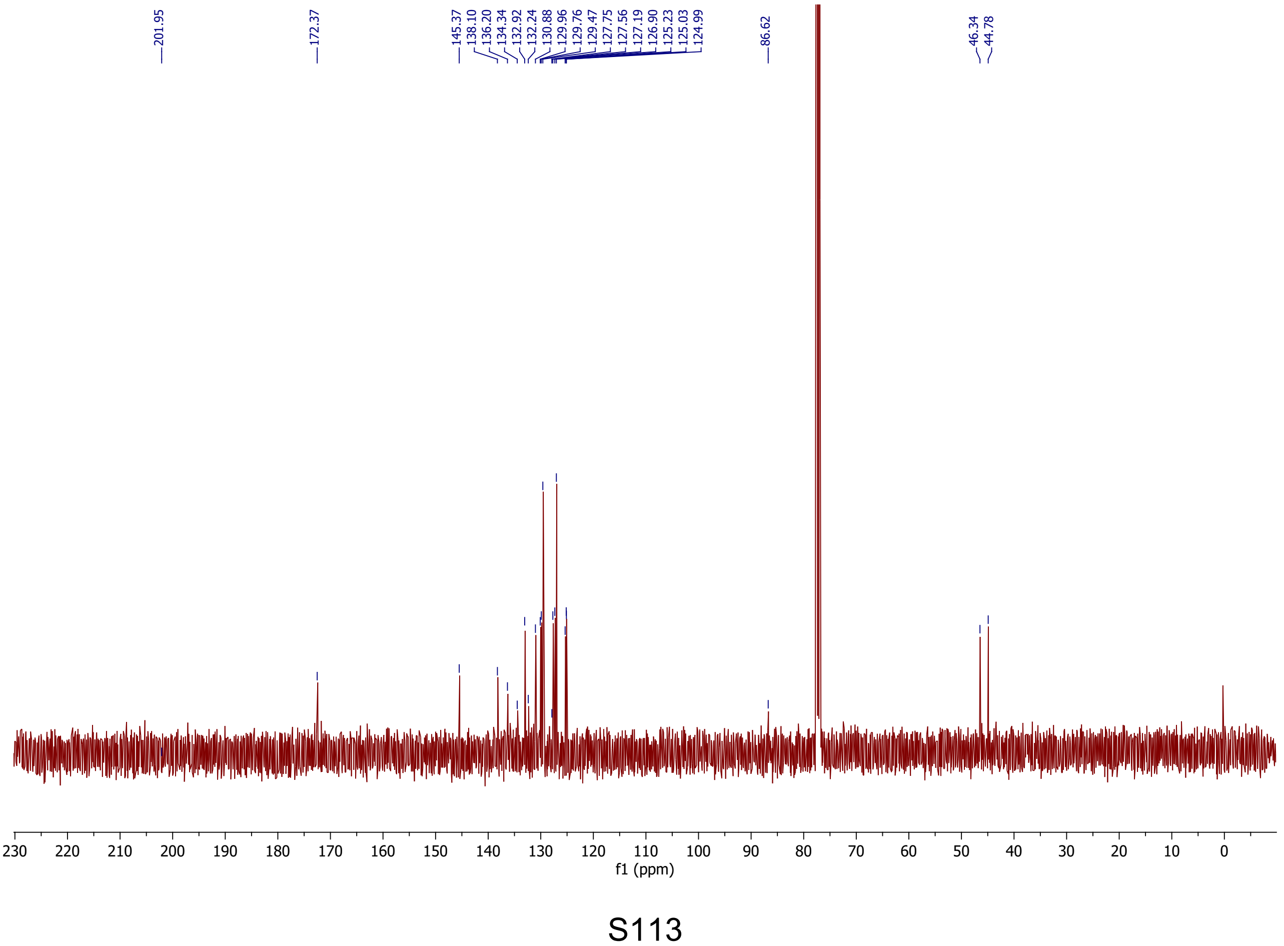

Figure S. 64. 1-(4-fluorophenyl)-9b-phenyl-3H-imidazo[2,1-a]isoindole-2,5-dione (6h)

^{1}H NMR (400 MHz, $CDCl_3$)

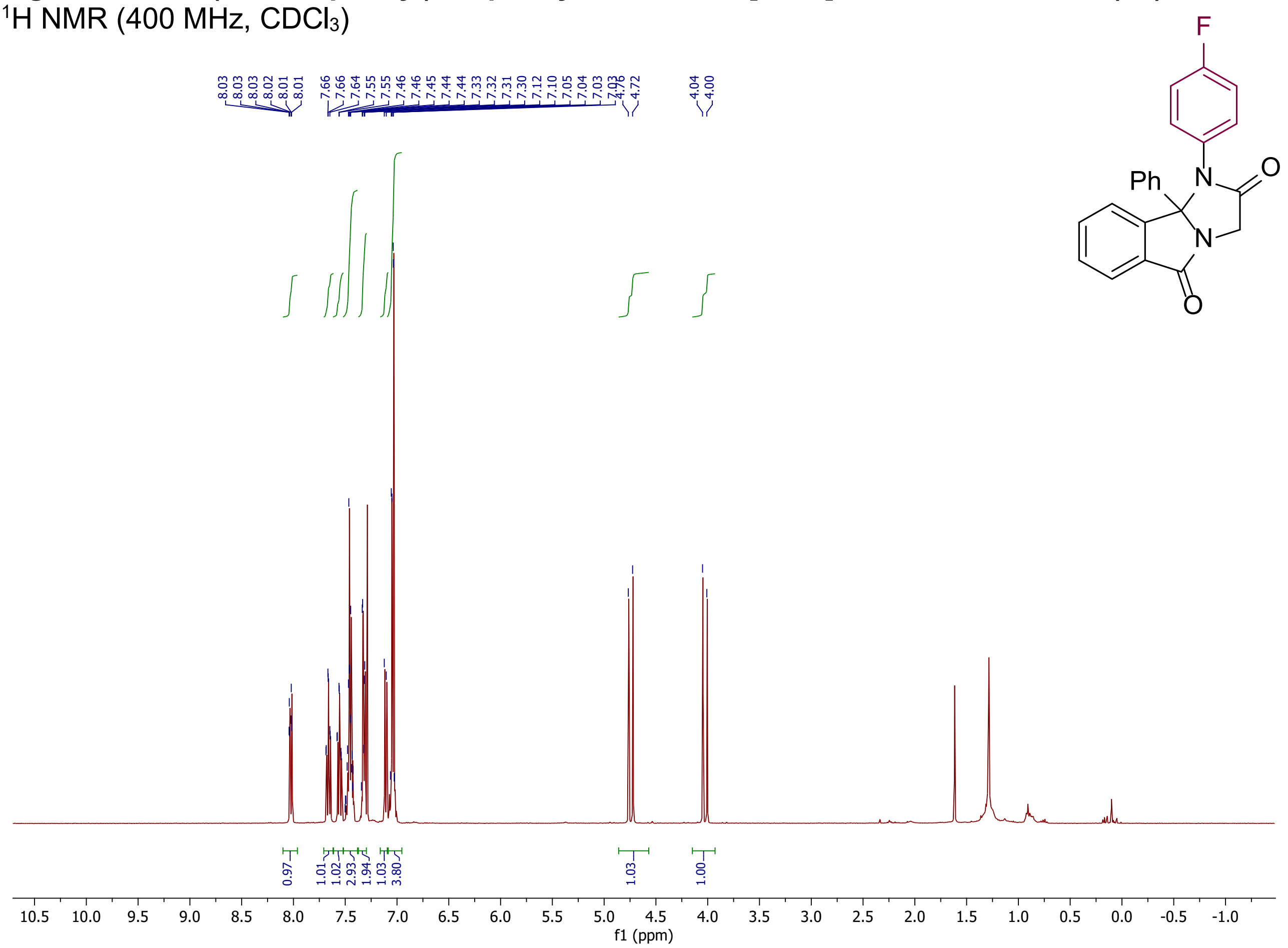

^{13}C NMR (101 MHz, $CDCl_3$)

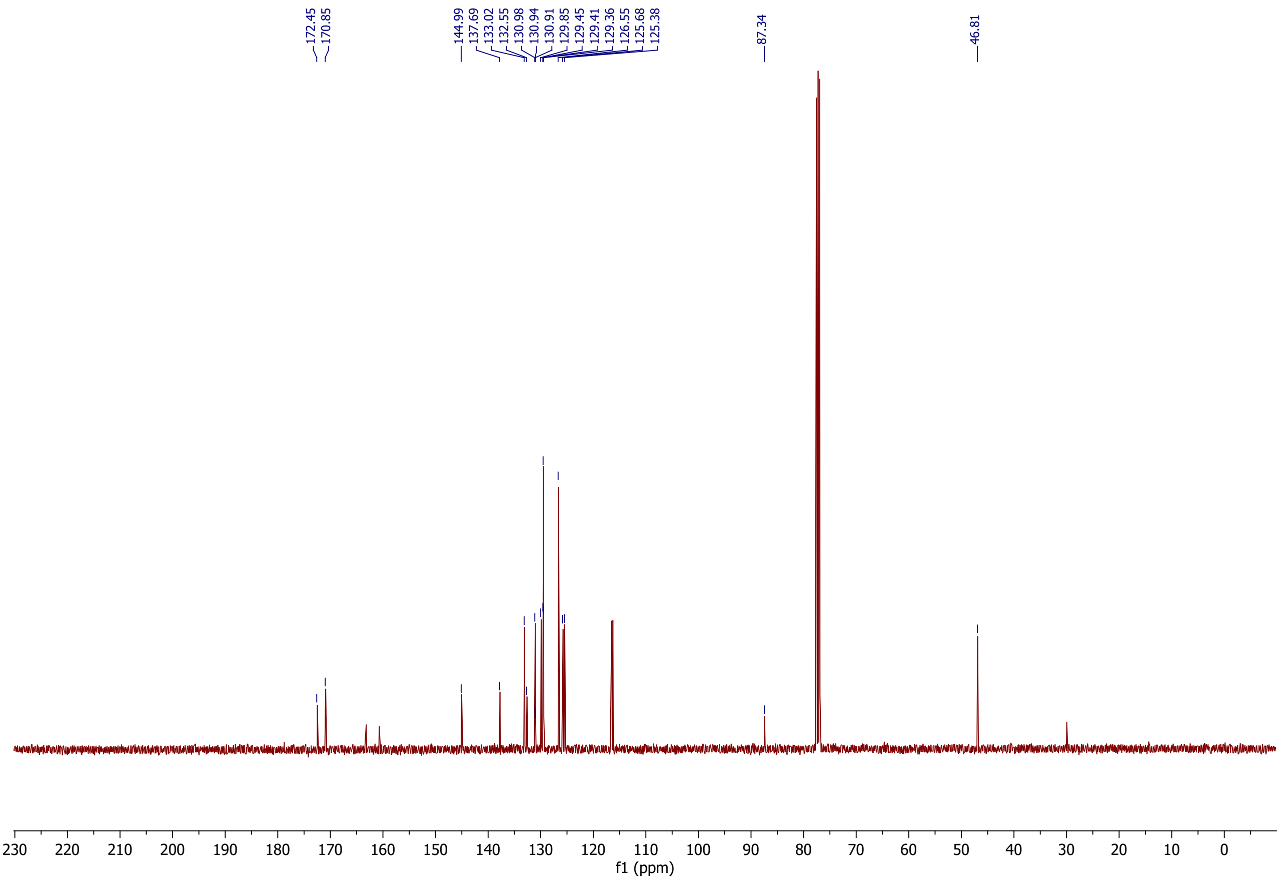

Figure S. 65. 1-(2-chloro-4-methyl-phenyl)-9b-phenyl-3H-imidazo[2,1-a]isoindole-2,5-dione (6i)

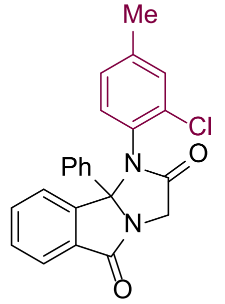

^{1}H NMR (400 MHz, $CDCl_3$)

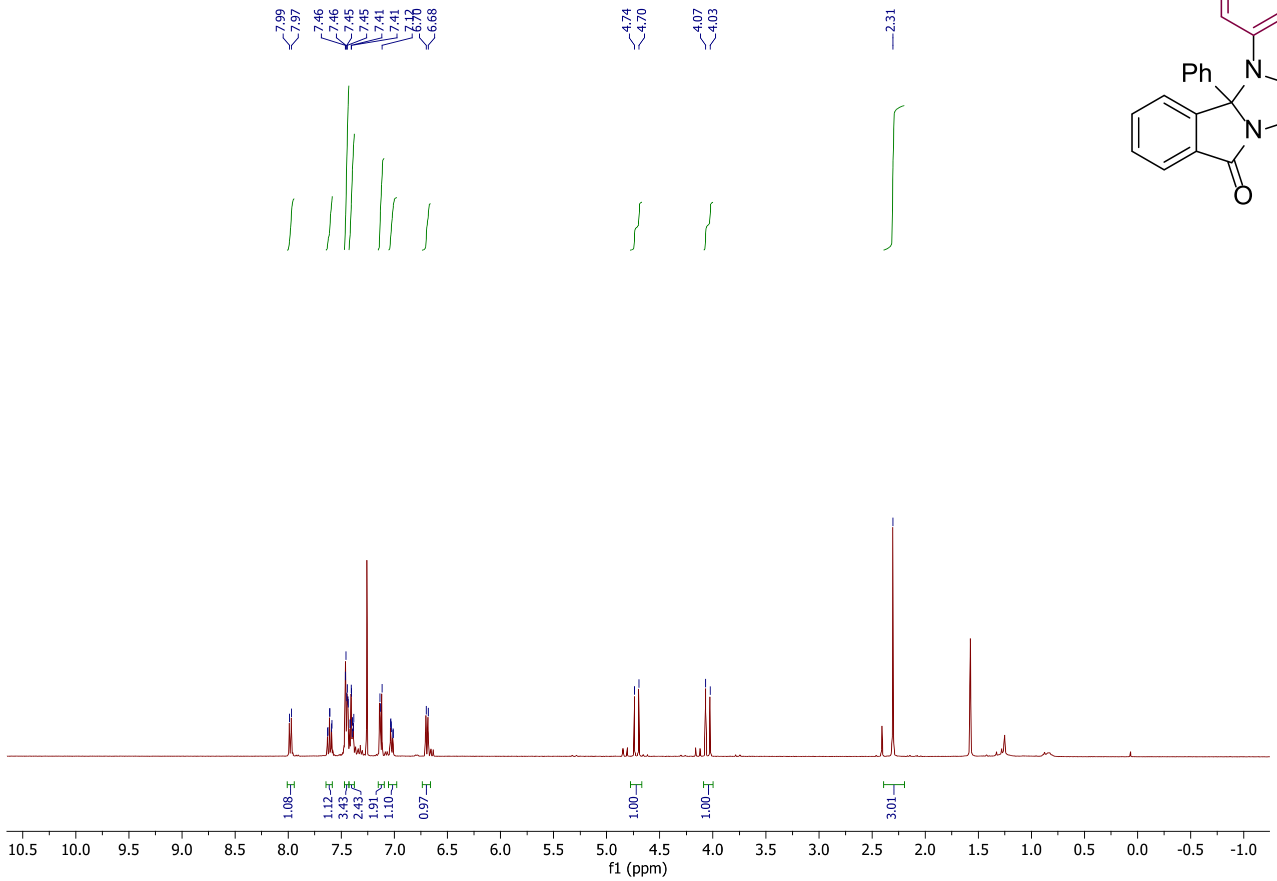

^{13}C NMR (101 MHz, $CDCl_3$)

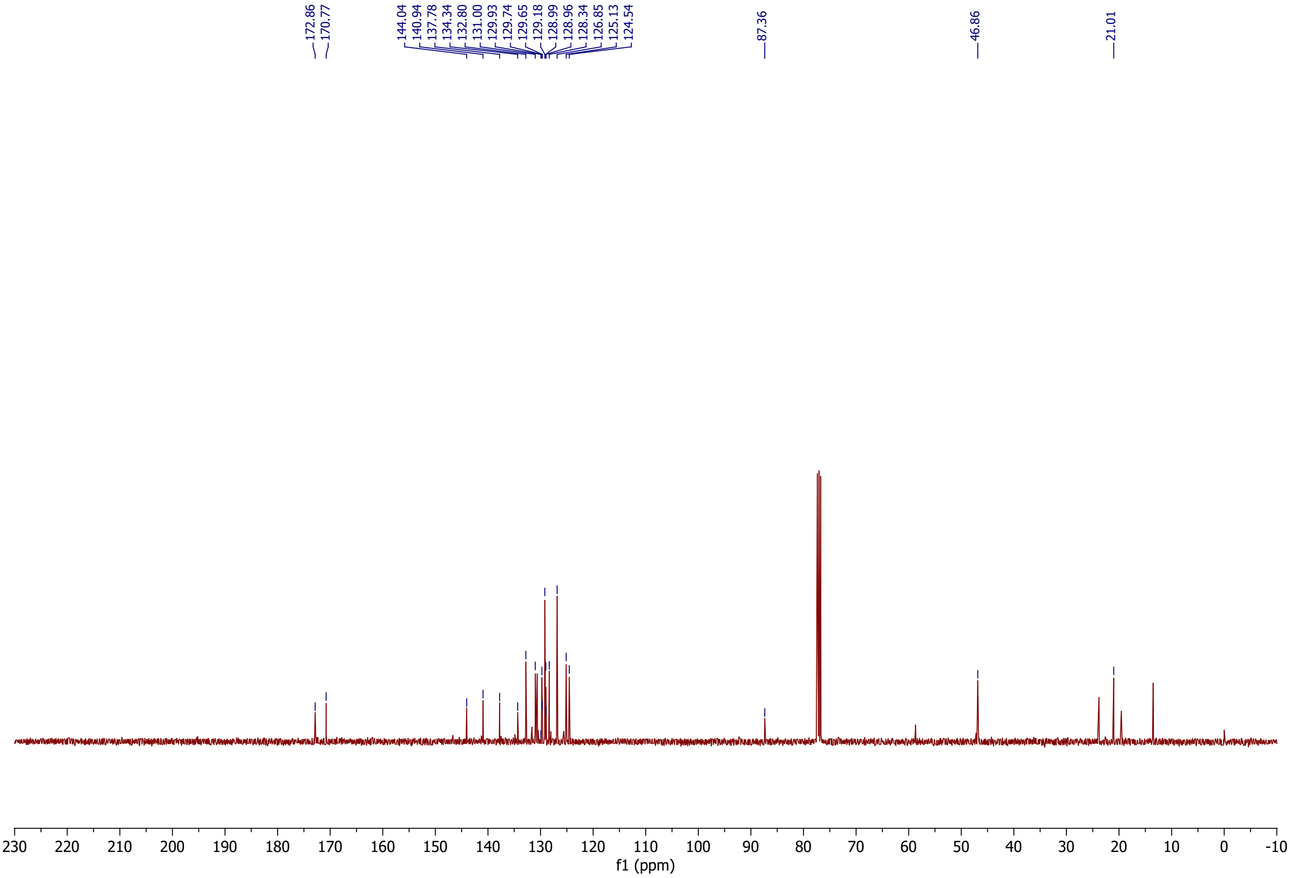

Figure S. 66. 1-cyclopropyl-9b-phenyl-3H-imidazo[2,1-a]isoindole-2,5-dione (6j)

^{1}H NMR (400 MHz, $CDCl_3$)

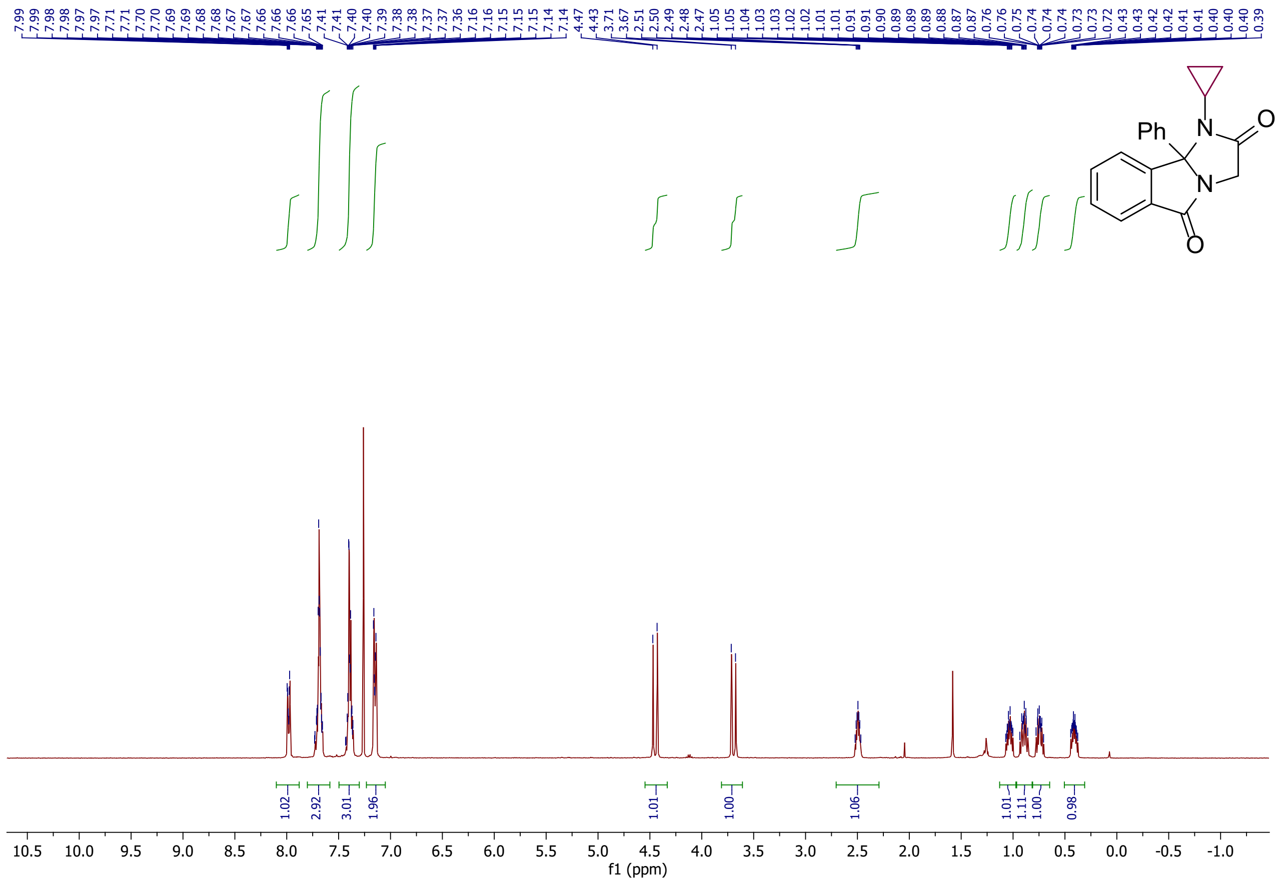

^{13}C NMR (101 MHz, $CDCl_3$)

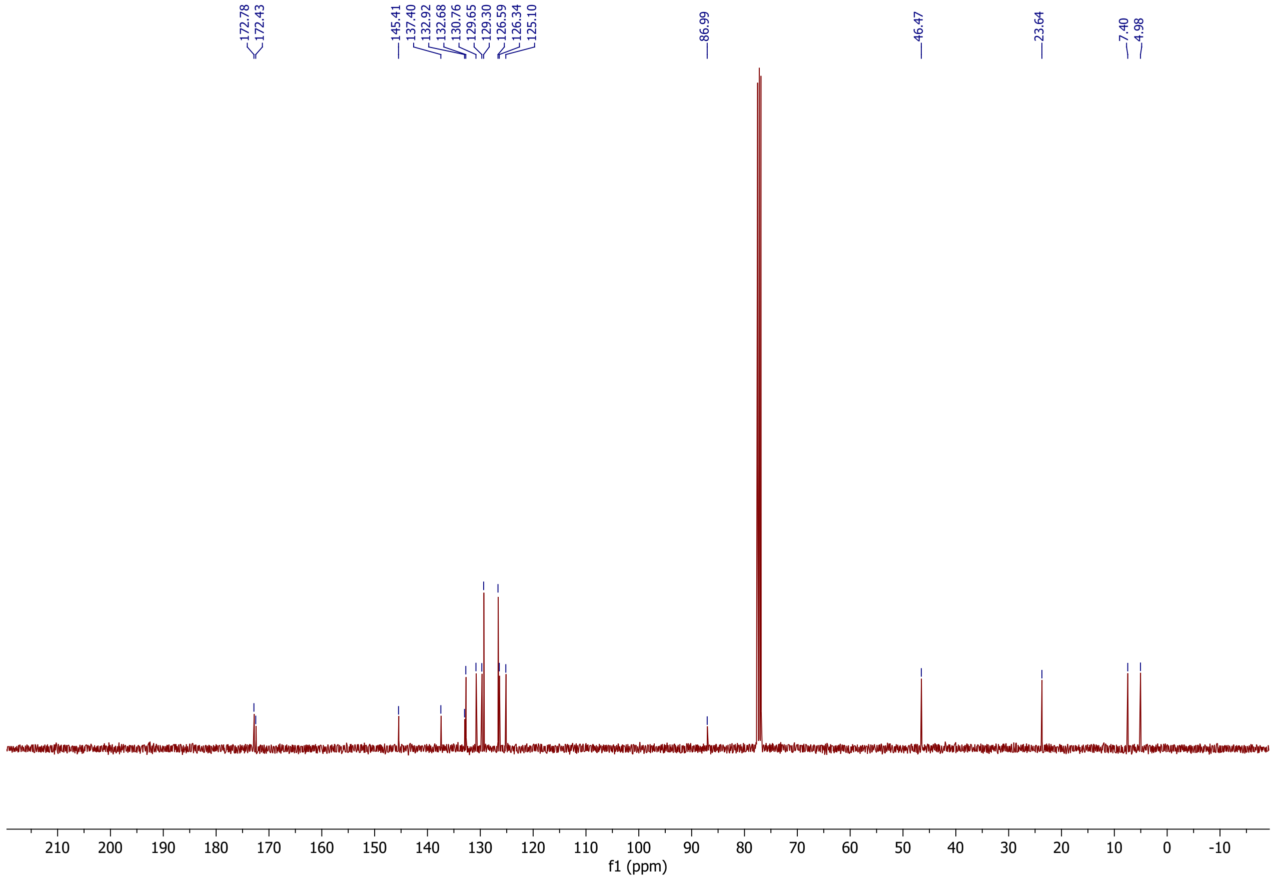

Figure S. 67. 1-isobutyl-9b-phenyl-3H-imidazo[2,1-a]isoindole-2,5-dione (6k)

^{1}H NMR (400 MHz, $CDCl_3$)

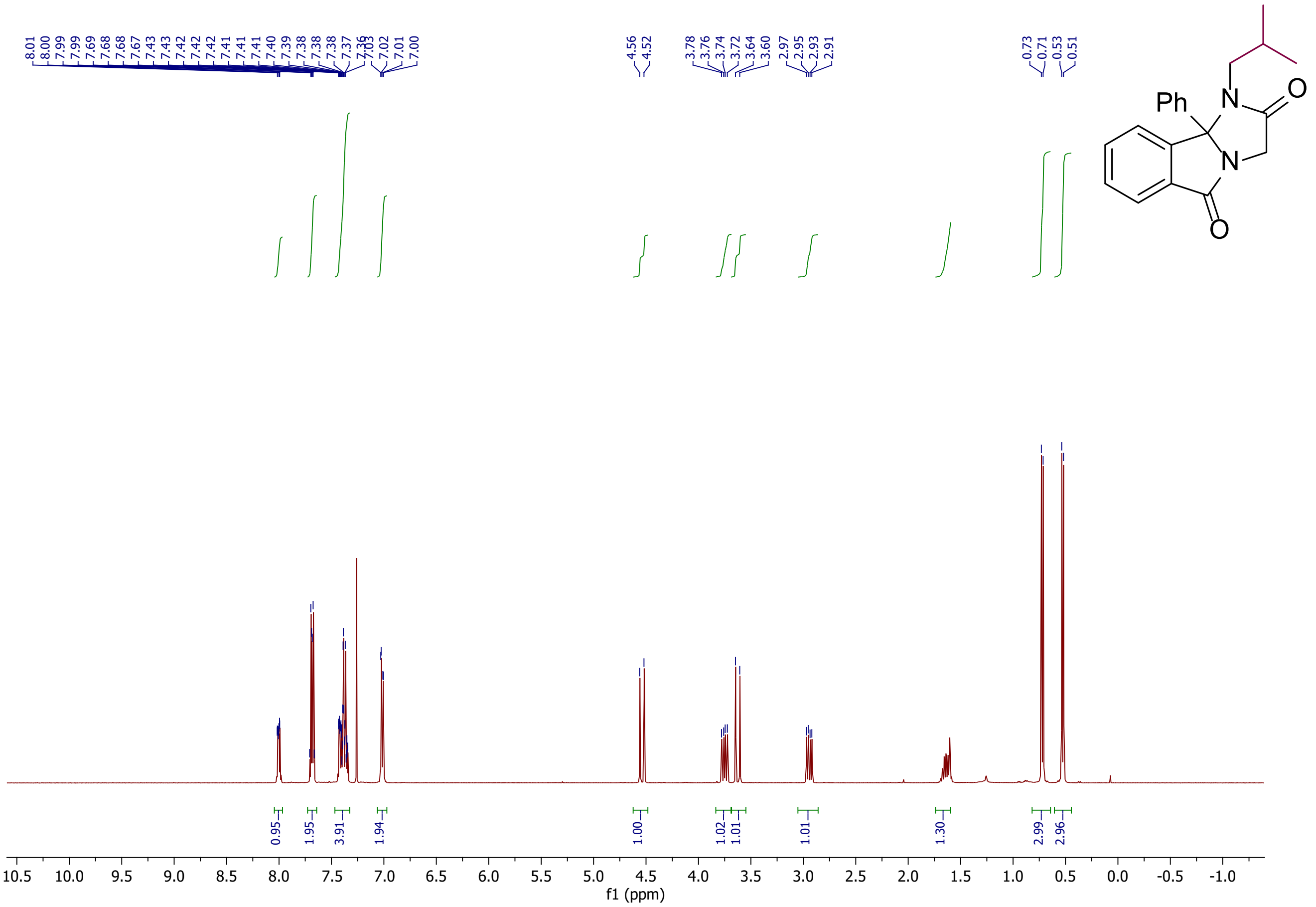

^{13}C NMR (101 MHz, $CDCl_3$)

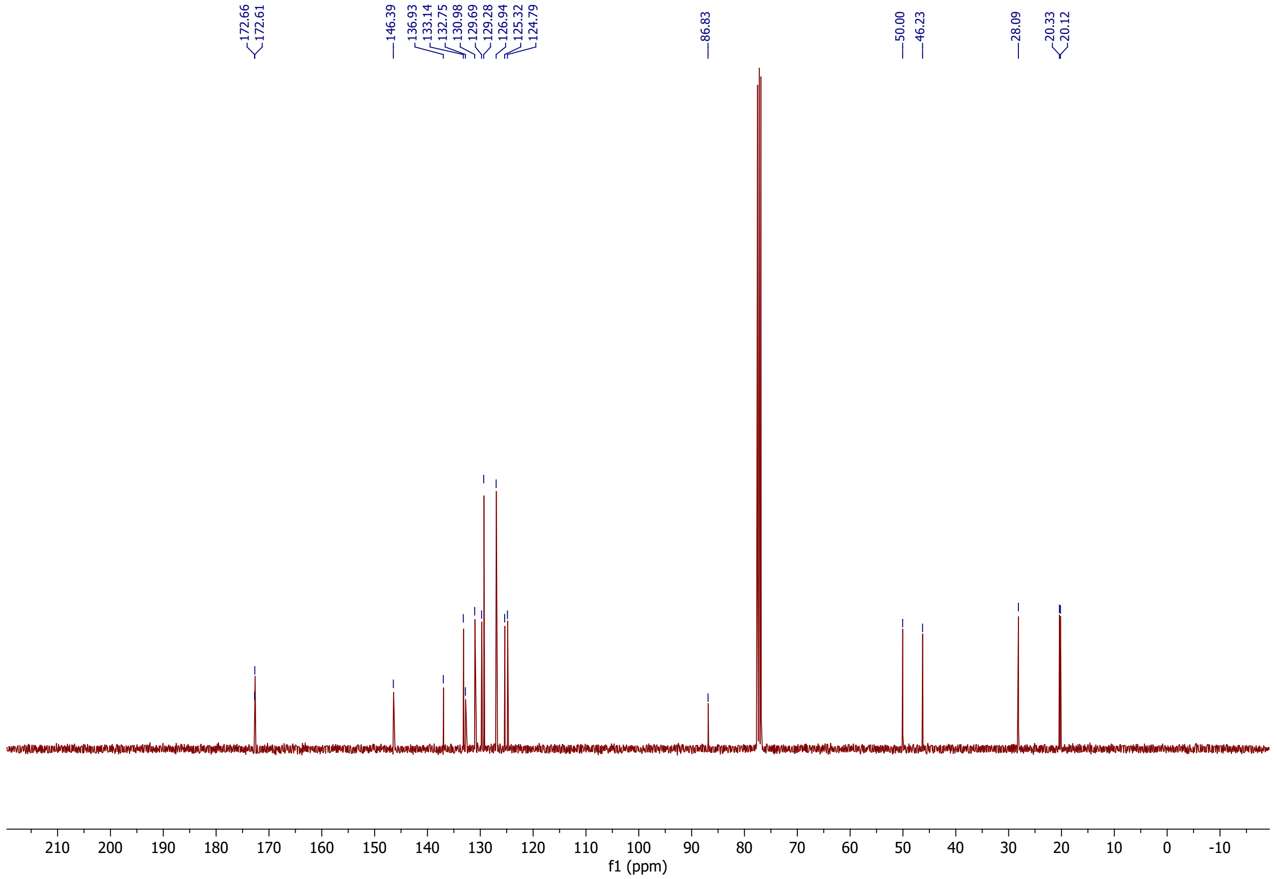

Supplement: Supplementary file 1 — Supporting Information [file CHEM-28-0-s001.pdf]
